# Expansion of *Thaumarchaeota* habitat range is correlated with horizontal transfer of ATPase operons

**DOI:** 10.1038/s41396-019-0493-x

**Published:** 2019-08-28

**Authors:** Baozhan Wang, Wei Qin, Yi Ren, Xue Zhou, Man-Young Jung, Ping Han, Emiley A. Eloe-Fadrosh, Meng Li, Yue Zheng, Lu Lu, Xin Yan, Junbin Ji, Yang Liu, Linmeng Liu, Cheryl Heiner, Richard Hall, Willm Martens-Habbena, Craig W. Herbold, Sung-keun Rhee, Douglas H. Bartlett, Li Huang, Anitra E. Ingalls, Michael Wagner, David A. Stahl, Zhongjun Jia

**Affiliations:** 1 State Key Laboratory of Soil and Sustainable Agriculture, Institute of Soil Science, Chinese Academy of Sciences, Nanjing, China; 2 Centre for Microbiology and Environmental Systems Science, Department of Microbiology and Ecosystem Science, University of Vienna, Vienna, Austria; 3 School of Oceanography, University of Washington, Seattle, WA USA; 4 Shanghai Majorbio Bio-pharm Biotechnology Co., Ltd, Shanghai, China; 5 Marine Biology Research Division, Scripps Institution of Oceanography, University of California, San Diego, La Jolla, CA USA; 6 Department of Energy Joint Genome Institute, Walnut Creek, CA USA; 7 Institute for Advanced Study, Shenzhen University, Shenzhen, China; 8 CAS Key Laboratory of Urban Pollutant Conversion, Institute of Urban Environment, Chinese Academy of Sciences, Xiamen, China; 9 College of Life Sciences, Nanjing Agricultural University, Nanjing, China; 10 Pacific Biosciences, Menlo Park, CA USA; 11 Department of Microbiology and Cell Science & Fort Lauderdale Research and Education Center, University of Florida, Gainesville, FL USA; 12 Department of Microbiology, Chungbuk National University, Cheongju, South Korea; 13 State Key Laboratory of Microbial Resources, Institute of Microbiology, Chinese Academy of Sciences, Beijing, China; 14 Center for Microbial Communities, Department of Chemistry and Bioscience, Aalborg University, Aalborg, Denmark; 15 Department of Civil and Environmental Engineering, University of Washington, Seattle, WA USA

**Keywords:** Metagenomics, Microbial ecology

## Abstract

*Thaumarchaeota* are responsible for a significant fraction of ammonia oxidation in the oceans and in soils that range from alkaline to acidic. However, the adaptive mechanisms underpinning their habitat expansion remain poorly understood. Here we show that expansion into acidic soils and the high pressures of the hadopelagic zone of the oceans is tightly linked to the acquisition of a variant of the energy-yielding ATPases via horizontal transfer. Whereas the ATPase genealogy of neutrophilic *Thaumarchaeota* is congruent with their organismal genealogy inferred from concatenated conserved proteins, a common clade of V-type ATPases unites phylogenetically distinct clades of acidophilic/acid-tolerant and piezophilic/piezotolerant species. A presumptive function of pumping cytoplasmic protons at low pH is consistent with the experimentally observed increased expression of the V-ATPase in an acid-tolerant thaumarchaeote at low pH. Consistently, heterologous expression of the thaumarchaeotal V-ATPase significantly increased the growth rate of *E. coli* at low pH. Its adaptive significance to growth in ocean trenches may relate to pressure-related changes in membrane structure in which this complex molecular machine must function. Together, our findings reveal that the habitat expansion of *Thaumarchaeota* is tightly correlated with extensive horizontal transfer of *atp* operons.

## Introduction

The ammonia-oxidizing archaea (AOA) are among the most abundant and ubiquitous microorganisms on Earth [1–3]. First identified as the dominant ammonia-oxidizing population in the upper ocean and soils [4, 5], subsequent studies also demonstrated a major presence in acidic and geothermal habitats [6–8]. Detailed molecular surveys of environmental distribution, based primarily on amplicon sequencing of the *amoA* coding for the alpha subunit of ammonia monooxygenase (AMO), further extended their habitat range to include the hadopelagic zone of the oceans [9].

Following the initial description of *Nitrosopumilus maritimus* SCM1 [10, 11], analysis of the physiology of AOA has been aided by cultivation of additional ecotypes, including neutrophilic *Nitrososphaera* species and obligately acidophilic *Candidatus* (*Ca.*) Nitrosotalea devanaterra Nd1 isolated from soils [12–14], and thermophilic *Nitrosocaldus* species enriched from geothermal habitats [15–17]. Among open ocean populations, *amoA*-based surveys point to a depth-dependent partitioning of distinct AOA ecotypes, including shallow (water column A) and deep marine groups (water column B) in the upper ocean [4, 18, 19] as well as a clade encompassing *Nitrosopumilus* species in the hadal zone below 6000 m [9, 20]. Although the deep ocean AOA ecotypes are presumably obligate or facultative piezophiles, adaptation to extreme hydrostatic pressure has not yet been explicitly considered as a major factor driving allopatric diversification.

Despite a habitat range that encompasses an extraordinarily wide range of physical/chemical conditions, all cultured AOA appear to rely solely on the energy generated from oxidation of ammonia that some AOA can also produce from urea or cyanate [11–14, 21]. AOA derive energy for anabolic processes by coupling ammonia oxidation to a copper-centric electron transfer system for generating a proton motive force (PMF) for ATP synthesis via an integral membrane ATPase [22]. The evolutionarily diverse archaeal ATPases (designated A-type ATPases) are functionally similar to the eukaryotic and bacterial F-type ATPases that catalyze ATP synthesis using a PMF [23]. However, they are structurally more similar to the vacuolar-type (V-type) ATPases of eukaryotes and some bacteria that function as proton pumps driven by ATP hydrolysis [23, 24]. Significant variation in subunit composition, structure, and mechanism of the archaeal variants is thought to confer adaptive advantage in the variety of extreme environments that archaea inhabit [25]. Since AOA are united by a single autotrophic metabolism dependent on ATP generation via a PMF, we examined the possible role that variation in ATPase composition and structure might play in their adaptive radiation into environments of low pH and high pressure.

Stable isotope probing (SIP) with ^13^C-labeled CO_2_ was used to selectively recover DNA from metabolically active AOA in acidic soils, significantly expanding the genomic representation of *Thaumarchaeota* active in acidic environments through the assembly of two novel genomes. Comparative analysis of these and available archaeal genomes and genomic fragments was then used to explore the evolutionary diversification of ATPases in relationship to archaeal habitats of varying pH and hydrostatic pressure. This revealed that the radiation of *Thaumarchaeota* into low-pH and high-pressure environments was clearly correlated with horizontal transfer of an operon encoding the same variant ATPase. The functional significance of this variant to adaptation to low pH is supported by our observations showing (i) increased transcription of the ATPase variant genes by an acid-tolerant AOA following transition from neutral to acidic growth conditions and (ii) higher growth rates at low pH of an *Escherichia coli* (*E. coli*) genetically modified to express the archaeal variant.

## Materials and methods

### Site description and soil sampling

Soil samples for DNA-SIP were collected at the long-term experimental field of the Tea Research Institute of the Chinese Academy of Agricultural Sciences (30°14′N, 120°09′E), Zhejiang Province, China [26, 27] in August 2011. Soil cores (20 cm long) were retrieved from a forest soil with a pH of 5.31 (site 1, FS) and tea orchard soil with a pH of 3.75 (site 1, TS). The latter was converted from the forest soil in 1974 [26, 27]. Both soils were 80 m apart and classified as quaternary red earth Ultisols. The sampling region was characterized by a subtropical wet monsoon climate with a mean annual temperature of 17 °C and a mean annual rainfall of 1533 mm. The tea orchard field plot received approximately 600 kg ha^−1^ urea-N, 280 kg ha^−1^ potassium sulfate, and 1125 kg ha^−1^ organic fertilizer (mainly rapeseed cake) annually since 1974 [26]. The 20 cm cores were collected in triplicate, homogenized, sieved through a 2.0 mm sieve, and stored at −20 °C until further analysis. Detailed soil properties are listed in Table S1.

For additional metagenomic comparisons, a second set of soil samples was collected from an acidic natural forest soil with pH 4.35 (site 2) in Yingtan City, Jiangxi Province of southern China (site 2, 24°29′N, 113°34′E) in June 2012 [28]. This soil was mainly covered with secondary evergreen broad-leaf camphor (*Cinnamomum camphora* L.) trees [28]. Three soil cores were collected, homogenized, sieved through a 2.0-mm sieve, and stored at −20 °C for further analysis.

### DNA-SIP-based metagenomic analysis of AOA

To determine the pH response of AOA in soils, 10 g of TS and FS soil samples were suspended in 50 ml 10 mM phosphate buffer adjusted to pH 4.5 and pH 7.0, amended with 200 μg ^15^N-urea (^15^N atom, 98% excess) g^−1^ dry weight soil (d.w.s.), and incubated in the dark for 7 days at 25 °C (Fig. S1). Ammonia oxidation activity was monitored by production of ^15^N-labeled nitrate plus nitrite as previously described [29]. Growth of AOA and ammonia-oxidizing bacteria (AOB) were determined by real-time PCR quantification of AOA and AOB *amoA* gene copies as described before [30] (Table S2). To recover AOA genomic DNA, DNA-SIP microcosms were constructed at native acidic pH as previously described [31]. Three microcosm-labeling treatments were performed: ^13^CO_2_-labeled, ^12^CO_2_ (control), and ^13^CO_2_+C_2_H_2_ (control). Each microcosm was amended with 100 μg urea-N g^−1^ d.w.s. weekly and incubated at 25 °C in the dark. Total DNA was extracted at day 56 using a FastDNA Spin Kit for Soil (MP Biomedicals, Cleveland, OH, USA) and subjected to isopycnic density gradient centrifugation [31]. Fourteen DNA gradient fractions were recovered from each 5.1 ml ultracentrifugation tube, and the DNA was separated from CsCl by PEG6000 precipitation [32] and dissolved in TE buffer for the following metagenomic analysis. More details can be found in Supplementary Information.

Multiple displacement amplification (MDA) of the ^13^C-labeled DNA (fractions 4–6, 1.730–1.740 g ml^−1^) from DNA-SIP microcosms were carried out using the REPLI-g Single Cell Kit (QIAGEN, Hilden, Germany) according to the manufacturer’s protocol (Fig. S2). The ^13^C-labeled DNA before and after MDA were analyzed by metagenomic sequencing on Illumina HiSeq and/or PacBio RS II platforms. HiSeq reads and PacBio reads of the ^13^C-DNA mainly fell into two bins of ~40% and 70% GC content. Phylogenetic analysis suggested that reads representing AOA genomes were found in the low GC cluster. Two AOA genomes, namely, FS and TS, were assembled by Velvet from the low GC bins [33]. Genome completeness and contamination were estimated using comparison of 145 lineage-specific marker genes of archaea by CheckM [34]. The average amino acid identity (AAI) [35] was calculated as in Herbold et al. [36]. The gene annotation and comparative genomic analysis can be found in Supplementary Information.

### Phylogenetic analyses

Inference of phylogenetic relationships among AOA and archaea was based on analysis of concatenated alignments of 36 and 122 conserved archaeal phylogenetic marker proteins [37–39] from 50 (meta)genomes of AOA and 158 selected representative genomes of *Euryarchaeota* and the archaeal superphyla TACK, Asgard, and DPANN (Table S3). Marker proteins in each genome were identified using hidden Markov models. Each protein was individually aligned using hmmalign [40]. The concatenated alignment was trimmed by TrimAL [41] with flag “-gt 0.5 -cons 50 -w 3.” Then maximum likelihood trees were built using IQ-TREE [42] with the best-fit model of “LG+I+G4” followed by extended model selection with FreeRate heterogeneity and 1000 times ultrafast bootstrapping [43].

The corresponding phylogenetic tree of ATPases was constructed based on the concatenation of seven subunits (A, B, D, E, F, I, and K) that were present in all selected archaeal genomes. ATPase genes were aligned by MAFFT [44] and the alignments were trimmed by TrimAL [41] with “-gappyout” flag. Inferences of maximum likelihood tree were achieved by IQ-TREE with “-MFP -bb 1000” flag for best-fit model selection and 1000 times ultrafast bootstrapping [43]. Phylogenies of *amoA* genes, aspartate carbamoyltransferase regulatory subunit (PyrI) genes, and thiosulfate/3-mercaptopyruvate sulfurtransferase genes flanking the *atp* operons of AOA were also constructed using IQ-TREE [42] as described above.

### Physiological experiments and transcription analyses

In order to determine relative transcription activity of V-ATPase of *Ca*. Nitrosocosmicus oleophilus strain MY3 at different pH, MY3 was incubated in artificial fresh water medium adjusted to pH 5.2 and 5.5 by HOMOPIPES buffer and 7.5 by HEPES buffer with the addition of 500 μM NH_4_Cl at 25 °C, respectively [45]. Each pH treatment had three replicates. Ammonium and nitrite concentrations were determined using a Skalar SAN Plus segmented flow autoanalyzer (Skalar, Breda, the Netherlands) as described before [31]. To determine growth of MY3, SYBR Gold-stained cells were directly counted on 0.2 µm polycarbonate GTTP membranes (Merck Milipore, Germany) by fluorescence microscopy [45]. Total RNA was extracted from 500 ml of exponentially growing cultures grown at pH 5.5 and 7.5 using the RNeasy Mini Kit according to manufacturer’s protocol (Qiagen, Germany). cDNA was synthesized from the total RNA using the SuperScript First Strand synthesis system (Invitrogen, San Diego, CA) with RNaseOUT solution (40 U μl^−1^, Invitrogen) according to the manufacturer’s instruction. The quantity of RNA and cDNA were determined by a Nanodrop ND-100 UV-vis Spectrophotometer (NanoDrop Technologies, Wilmington, DE). Transcript copy numbers of the genes of *ntpA* (encoding ATPase subunit A), 16S rRNA, *amoA*, 4-hydroxybutyrl-CoA dehydratase, and methylmalonyl-CoA mutase large subunit were quantified by real-time PCR using the iQ SYBR Green Supermix Kit on a C1000 CFX96 real-time PCR system (Bio-Rad). Finally, the cDNA transcripts of each gene were normalized to 1 ng of RNA for comparison between pH 5.5 and 7.5. More details of pressure experiments can be found in Supplementary Information.

### pH-dependent growth experiments with *E. coli* expressing the thaumarchaeotal V-type *atp* operon

The V-type *atp* operon from the genome of *Ca*. Nitrosotalea okcheonensis CS was synthesized by Sangon Biotech (Shanghai, China) after codon optimization for *E. coli* (see the sequence in Supplementary Information), inserted into the NdeI-XhoI site of plasmid pET29a(+), and transformed into *E. coli* BL21(DE3) [46]. A single positive colony from LB agar plate with 50 μg ml^−1^ kanamycin was picked and inoculated into 100 ml of LB medium. It was incubated at 37 °C with shaking at 200 rpm until an OD_600_ of ~1.8 was reached. Then 0.3 ml of the culture was transferred into 100 ml of M9 medium (Na_2_HPO_4_, 6.78 g l^−1^; KH_2_PO_4_, 3 g l^−1^; NaCl, 0.5 g l^−1^; NH_4_Cl, 1 g l^−1^; MgSO_4_, 0.12 g l^−1^; CaCl_2_, 0.01 g l^−1^; glucose, 4 g l^−1^) with 15 μg ml^−1^ yeast extract, 50 μg ml^−1^ kanamycin, and 0.25 mM isopropyl-β-d-thiogalactopyranoside (IPTG) at pH 4.2, 4.5, 5.0, and 7.0, respectively. These cultures were incubated at 37 °C with shaking at 200 rpm. The growth of *E. coli* were determined by optical density (OD) measurements at 600 nm every ~2–3 h. *E. coli* BL21(DE3) containing the empty vector pET29a(+) was used as the negative control. Each treatment was performed in three replicates.

### Accession numbers of nucleotide sequences and genomes

The AOA metagenome assembled genomes (MAGs) obtained in this study have been deposited in NCBI under accession numbers RCMB00000000, RCMC00000000, and QURE00000000. Nucleotide sequences for 16S rRNA genes and *amoA* genes have been deposited in GenBank under accession numbers MF037823-MF037825 and MF041805-MF041807.

## Results and discussion

### ^13^C-DNA-labeling of acidophilic *Thaumarchaeota*

^15^N-tracer experiments as well as archaeal *amoA* qPCR data suggested ammonia-oxidizing activity and growth of AOA in the acidic forest and tea orchard soils (Fig. S3 and Supplementary Information). Following labeling of urea-amended forest and tea orchard soils with ^13^C-labeled CO_2_ at in situ pH, DNA from two distinct *Nitrosotalea*-like AOA (FS and TS) sharing 87.0% *amoA* gene sequence identity was highly enriched in the ^13^C-DNA heavy fraction (Figs. S3c, d and S4). Addition of the inhibitor acetylene confirmed that incorporation of CO_2_ by AOA primarily depended on the energy generated from ammonia oxidation [47] (Fig. S3). Near complete MAGs of the forest type, FS (1.82 Mb, 89.66% completeness), and tea orchard type, TS (1.56 Mb, 82.85% completeness), were assembled from the ^13^C-labeled DNA metagenome (Table S4).

**Fig. 1:**
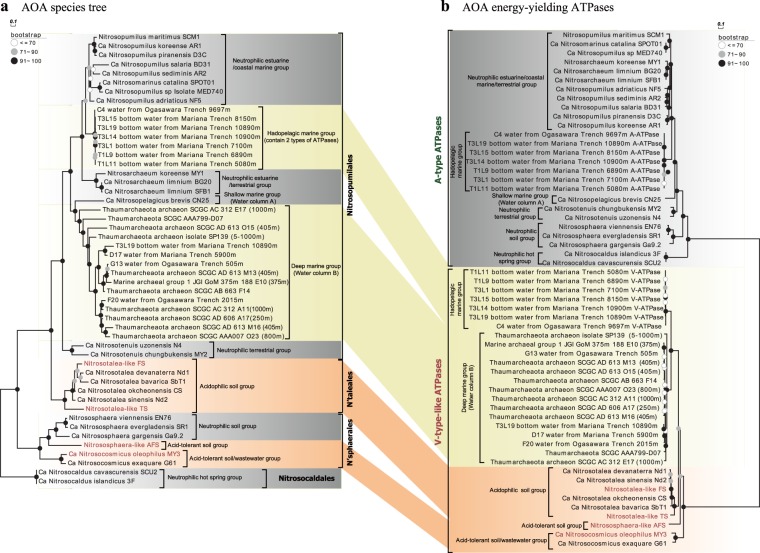
Comparative phylogeny of 36 conserved phylogenetic marker proteins and energy-yielding ATPases of ammonia-oxidizing archaea (AOA). All culture genomes, metagenome assembled genomes (MAGs), and single amplified genomes (SAGs) of AOA deposited in NCBI and JGI were collected, and only those containing the whole *atp* operons and at least 30 of the selected 36 and 100 of the selected 122 conserved phylogenetic marker proteins were used for comparative phylogenetic analysis (Figs. 3, S8, and S13). **a** Maximum likelihood species phylogenetic tree of AOA based on 36 concatenated phylogenetic marker proteins [IQ-TREE [42] with best-fit model LG+I+G4, 1000 ultrafast bootstraps]. “Nʹsphaerales” represents *Nitrososphaerales* and “Nʹtaleales” represents *Nitrosotaleales*. **b** Maximum likelihood tree of the ATPases based on the concatenation of the 7 subunits (A, B, D, E, F, I, and K) present in all AOA genomes [IQ-TREE [42] with best-fit model, 1000 ultrafast bootstraps]

**Fig. 2:**
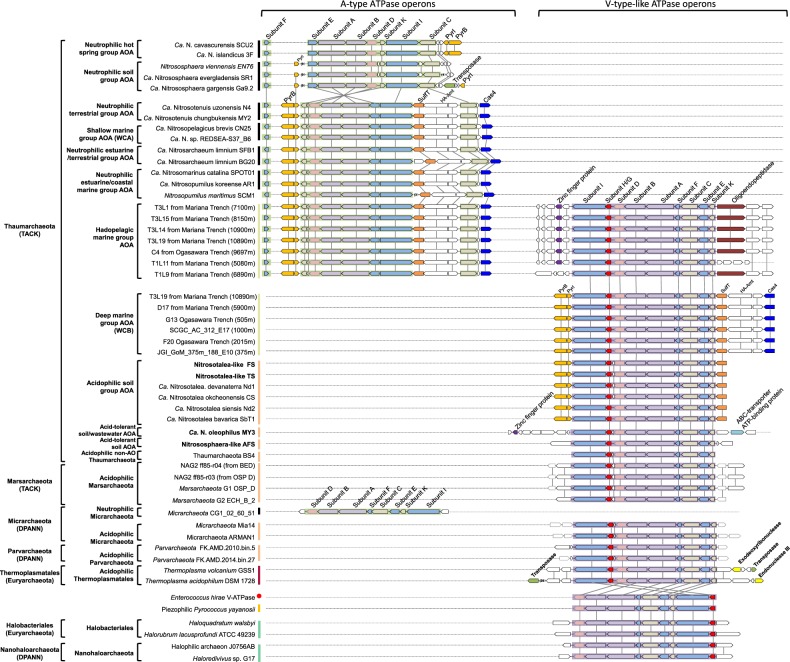
Schematic illustration of gene operons encoding for energy-yielding ATPases of *Thaumarchaeota* and other selected archaeal representatives. Homologous genes are indicated by color and connected by lines. Arrows indicate the direction of transcription. The genes flanking the ATPase operons are also shown. “SCGC_AC_312_E17” represents marine *Thaumarchaeota* SCGC_AC_312_E17 in Fig. 1. “PyrB” represents aspartate carbamoyltransferase, “PyrI” represents aspartate carbamoyltransferase regulatory subunit, “sulfT” represents thiosulfate/3-mercaptopyruvate sulfurtransferase, “HA-Amt” represents high-affinity ammonia transporter, and “Cas4” represents CRISPR-associated exonuclease Cas4. The number in the brace indicates the number of genes that are not shown

The AAIs between the FS and TS genomes and the four published *Nitrosotalea* genomes (Nd1, Nd2, CS, and SbT1) were 78.0–81.8% and 67.7–69.2%, respectively, and thus the newly recovered MAGs significantly increase the genomic representation of *Ca. Nitrosotaleales* [36] (Fig. S5). FS and TS shared 682 and 631 genes (91.7% and 84.9%), respectively, of the 743 core genome genes of AOA recently reported by Herbold et al. [36] (Fig. S6 and Tables S5–7) and possess a set of genes implicated by those authors in adaptation to low pH (Fig. S7 and Tables S8–11). These genes were partly acquired via horizontal gene transfer from acidophiles [36] and further studies are needed to explore exact function in acidophilic AOA. However, a feature not explored at depth in that study is our observation here that all *Thaumarchaeota* from acidic soils, including the new strains reported here (FS, TS, and AFS, a *Nitrososphaera*-like MAG recovered from another acidic soil, see Supplementary Information) and the four published *Nitrosotalea* strains as well as an acid-tolerant strain *Ca*. Nitrosocosmicus oleophilus MY3 regardless of their phylogenetic status [36, 45] (Figs. 1a and S8a), encode a specific energy-yielding ATPase (Figs. 1b and 2). This clade of ATPases forms a well-supported monophyletic group and is phylogenetically distinct from those encoded by neutrophilic soil *Thaumarchaeota* (Figs. 1b and S8d–i). This distribution strongly implies that variants of the energy-yielding ATPase are associated with niche specialization.

### Distribution of thaumarchaeotal ATPase variants in relationship to pH and hydrostatic pressure

**Fig. 3:**
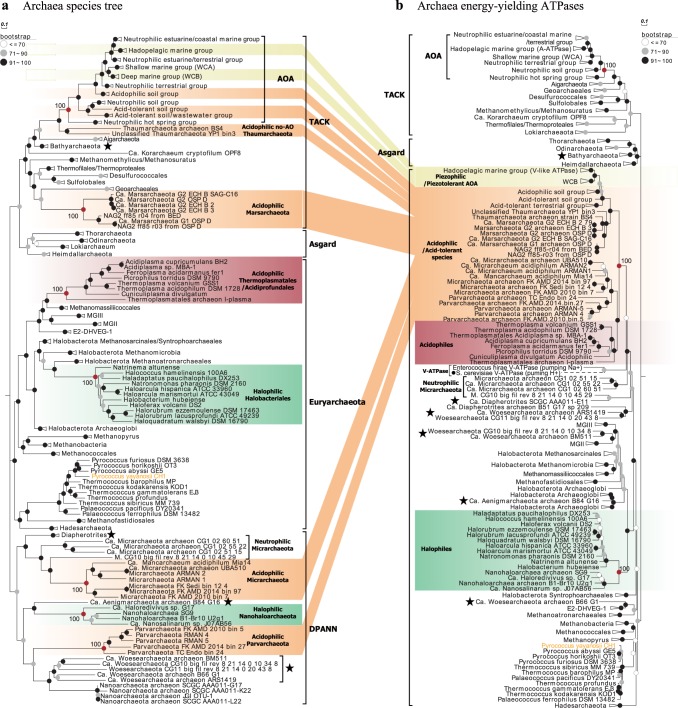
Comparative phylogeny of 36 conserved phylogenetic marker proteins and energy-yielding ATPases of Archaea domain. Only the genomes containing at least 30 of the selected 36 and 100 of the selected 122 conserved phylogenetic marker proteins as well as the whole *atp* operons were selected for comparative phylogenetic analysis (Figs. 1, S8, and S13). **a** Maximum likelihood species phylogenetic tree of archaea based on 36 conserved phylogenetic marker proteins [IQ-TREE [42] best-fit model LG+I+G4, 1000 ultrafast bootstraps]. Totally, 50 thaumarchaeotal genomes as well as 158 representative genomes of the phylum *Euryarchaeota* and superphyla TACK, Asgard, and DPANN were included. **b** Maximum likelihood tree of the ATPases based on the concatenation of the 7 major subunits (A, B, D, E, F, I, and K) present in all archaeal genomes [IQ-TREE [42] best-fit model, 1000 ultrafast bootstraps]. The V-ATPases from *S. cerevisiae* and *E. hirae* were also included in the phylogenetic analysis. The phylogenetic position of the *S. cerevisiae* V-ATPase (solid circles (●), dotted line) was inferred from the phylogeny of only subunits A and B, since its subunit compositions differed from archaeal ATPases. Some Archaeal branches whose positions in ATPase tree disagreed with their positions in the species tree are marked by stars

The membrane rotary ATPases, including the A-type in archaea, V-type in eukaryotes and a few bacteria, and F-type in eukaryotes and bacteria, normally contain ~8−11 subunits, which form a hydrophilic catalytic A_1_/V_1_/F_1_ domain and a membrane-embedded A_0_/V_0_/F_0_ domain as well as one central and 1–3 peripheral stalks [25, 48, 49] (Table S12). The ATPase of acidophilic/acid-tolerant AOA is homologous to the proton-pumping V-ATPase of eukaryotes and the ion-pumping V-ATPase of *Enterococcus hirae*, each containing nine subunits encoded by a single operon structurally and phylogenetically distinct from A-ATPases of neutrophilic AOA [48] (Figs. 2 and 3). In contrast, only eight of the nine corresponding subunits of the A-ATPase of neutrophilic *Thaumarchaeota* have been identified by homology searches (Fig. 2). No clear homolog of subunit H (corresponding to subunit G in some studies of V-ATPases [49], thus termed as subunit H/G in this study) in neutrophilic AOA has yet been reported. For simplicity, we here refer to the ATPase variant present in acidophilic/acid-tolerant AOA (e.g., *Nitrosotalea* spp. and *Nitrososphaera*-like spp., such as AFS obtained from acidic soil) as V-type-like, and the variant present in cultured neutrophiles (e.g., cultured *Nitrososphaera* spp. [12, 14]) as A-type. Our comparative analyses clearly show that neutrophilic strains contain the A-type, whereas those retrieved from acidic environments share in common the V-type-like ATPase of acidophilic *Nitrosotalea* spp. (Figs. 1b, 2, and 3).

Our comparative analyses also revealed an unusual bifurcation of archaeal A-ATPase and V-ATPase variants with depth in the ocean. Prior ocean surveys revealed that marine AOA fell into three distinct phylogenetic clusters, termed *Nitrosopumilus*/*Nitrosomarinus*-like AOA, water column A (WCA), and water column B (WCB), across diverse oceanographic regions [4, 50] (Fig. 1a). *Nitrosopumilus*/*Nitrosomarinus*-like AOA are typically most abundant in coastal areas, while WCA and WCB represent two vertically segregated major phylotypes in the open ocean [18, 50] (Fig. 1a). WCA predominate in the epipelagic water and are less abundant below the upper mesopelagic zone [51, 52]. In contrast, WCB are found primarily below the euphotic zone, becoming dominant in the deeper mesopelagic and bathypelagic zones [51, 52]. Consequently, the WCA- and WCB-AOA have been thought to represent shallow and deep-adapted pelagic ecotypes, respectively. Notably, we found a very clear separation of these two ecotypes by distinct ATPase gene clusters (Figs. 1b and 2). The gene clusters encoding A-type ATPase occur in *Nitrosopelagicus*-like WCA-AOA (Figs. 1b and 2). In contrast, V-type-like ATPase gene clusters occur in WCB-AOA populations from the deeper ocean, sharing the conserved *atp* operon composition and organization with those of the acidophilic and acid-tolerant soil AOA (Figs. 1b and 2).

Marine AOA comprise nearly 40% of bacteria and archaea below 1000 m and are abundant in the deepest hadal zones of the oceans [1, 9]. A notable observation in the recent characterizations of the Challenger Deep was the greater representation of AOA ecotypes closely related to estuarine/coastal *Nitrosopumilus* species (genus *Nitrosopumilus*; [10]) between 6000 and 10,000 m [9, 20]. Similar observations were made in the Puerto Rico Trench at 8200 m, identifying *Thaumarchaeota* and SAR11 species as presumptive piezophilic populations at this depth [53]. These hadopelagic AOA share >98% 16S rRNA gene sequence identity and >80% whole-genome identity with *N. maritimus* SCM1 [20], a cultivated coastal marine AOA representative [10, 11]. The conserved A-type *atp* operons were found in both *Nitrosopumilus*-like hadopelagic and coastal marine AOA genomes (sharing >82.2% identity; Table S13). However, surprisingly, these deep sea variants also contain a complete gene cluster encoding the V-type-like ATPase present in acidophilic AOA (Figs. 1b and 2). The presence of a second ATPase thus appears to be related to the growth of *Nitrosopumilus*-like AOA in the hadopelagic environment.

### Evidence and implication of functional significance

**Fig. 4:**
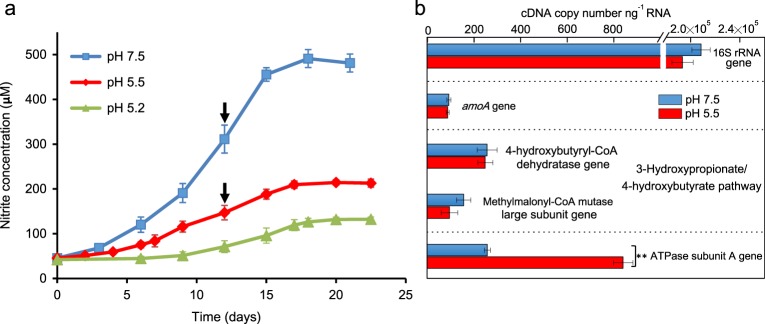
Growth and transcription of V-ATPase genes in strain *Nitrosocosmicus oleophilus* MY3 at different pH. **a** Strain MY3 was grown in artificial fresh water medium (AFM) containing 500 μM NH_4_Cl at pH 5.2, 5.5, and 7.5, respectively. Growth was monitored via nitrite accumulation. The initial cell density after inoculation was ~1.0 × 10^5^ cells ml^−1^. **b** Transcript abundance of 16S rRNA, *amoA*, 4-hydroxybutyryl-CoA dehydratase, methylmalonyl-CoA mutase large subunit, and ATPase subunit A were determined by quantitative PCR (qPCR) after reverse transcription of total RNA extracted from MY3 at mid-exponential phase (marked with arrows) at pH 5.5 and 7.5, respectively. All qPCR results were normalized to 1 ng of RNA for comparison between pH 5.5 and 7.5. Error bars represent the standard errors of triplicate incubations. For each gene, significance difference in measured cDNA copy numbers between pH 5.5 and 7.5 was detected using a *t* test (**P < 0.01)

We experimentally evaluated the expression of the V-type-like ATPase in an AOA at different pH using *Ca*. Nitrosocosmicus oleophilus MY3 isolated from a coal-tar contaminated sediment [45]. This organism grows from pH 5.2 to 8.5 [45] (Figs. 4a and S9), enabling a comparative transcript analysis of the V-type-like ATPase relative to other genes of central metabolism at both circum-neutral and acidic pH. The relative transcript levels of genes coding for the 16S rRNA, the alpha subunit of the AMO, and enzymes in the pathway for CO_2_ fixation (4-hydroxybutyryl-CoA dehydratase and methylmalonyl-CoA mutase) remained relatively constant between pH 5.5 and 7.5 (Fig. 4b). In contrast, the relative transcript abundance for the gene encoding subunit A of the V-ATPase increased 3.5-fold at pH 5.5 relative to pH 7.5 (*P* < 0.01) (Fig. 4b). Since cultivated AOA species encode either an A-type or V-type ATPase, but not both, for generating ATP, the V-type of the acidophilic *Thaumarchaeota* apparently functions both in ATP synthesis and ATP-dependent proton pumping under low-pH conditions. A function in proton pumping is also consistent with a close phylogenetic association of the ATPase of acidophilic *Thaumarcheaota* and the proton/ion-pumping V-type ATPases of *Saccharomyces cerevisiae* and *E. hirae* [48] (Fig. 3b). A closer inspection of the amino acid sequences of the V-ATPases of the *Thaumarchaeota* suggests preferential selectivity for protons over sodium cations as the critical hydrophobic residues were detected around the ion-binding site in the primary structure of the vc/K rings (Supplementary Information, Fig. S10). Although a dual function of the thaumarchaeotal variant (ATP synthesis and ATP-dependent proton pumping) has yet to be experimentally verified, comparative structural analysis points to significant functional differences between the two types of ATPases found in *Thaumarchaeota* (Supplementary Information, Fig. S11).

**Fig. 5:**
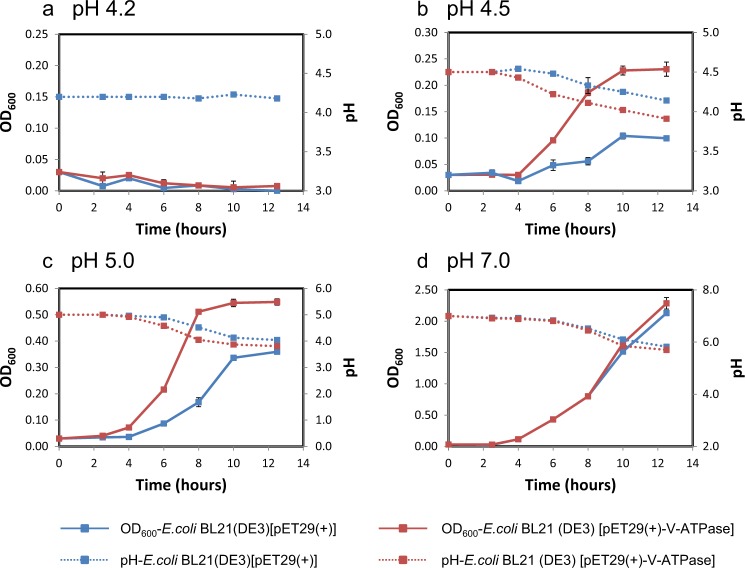
Growth of *E. coli* BL21(DE3) [pET29a(+)+V-*atp*] and *E. coli* BL21(DE3) [pET29a(+)] in M9 media at different pH. In all, 0.3 ml of the mother culture with an OD_600_ of ~1.8 was inoculated into 100 ml of M9 medium with 50 μg ml^−1^ kanamycin and 0.25 mM IPTG at pH 4.2 (**a**), 4.5 (**b**), 5.0 (**c**), and 7.0 (**d**), respectively, and incubated at 37 °C with shaking at 200 rpm. Growth of *E. coli* and media pH were determined at ~2-h intervals. Error bars represent the standard errors of triplicate incubations. Error bars smaller than symbols are not shown.

In order to further experimentally investigate the role of thaumarchaeotal V-ATPase in adaptation to acidic environments, the V-ATPase encoding operon of *Ca*. Nitrosotalea okcheonensis CS was (after codon optimization) expressed in *E. coli*. Interestingly, *E. coli* BL21(DE3) expressing the heterologous V*-*ATPase from an acidophilic AOA grew significantly faster than the negative control *E. coli* BL21(DE3) strain carrying the empty vector [pET29a(+)] in M9 medium at an initial pH of 4.5 and 5.0 (Fig. 5b, c). After ~12 h of incubation of *E. coli* BL21(DE3) [pET29a(+)+V-*atp*] in M9 media, the OD_600_ increased to 0.23 and 0.56 at initial pH values of 4.5 and 5.0, respectively (Fig. 5b, c). This was significantly higher (*P* < 0.01) than the negative control, reaching OD_600_ values of only 0.09 and 0.35 (Fig. 5b, c) at pH 4.5 and 5.0, respectively. Similar growth rates of both *E. coli* strains were observed at an initial pH of 7.0 for the first ~10 h of incubation (Fig. 5d). However, as the pH dropped to below ~6.0 at longer incubation times, the growth of *E. coli* harboring V-*atp* was slightly faster than that of the negative control (*P* > 0.05; Fig. 5d). This pH drop reflects the release of organic acids by *E. coli* during its growth on glucose in M9 media [54]. Decreasing pH values were also observed in the growth experiments with initial pH values of 4.5 and 5.0 (Fig. 5b, c). No growth was observed at an initial pH of 4.2 (Fig. 5a). These results strongly suggest an important role of V-ATPases in the adaptation of acidophilic/acid-tolerant AOA to low-pH environments, although more work is clearly needed to biochemically characterize the thaumarchaeotal V-ATPases.

Since pH variability is low in the well-buffered deep ocean, the V-ATPase in deep marine AOA more likely functions in adaptation to other characteristics specific to deep waters. A central feature of deep marine environments is elevated hydrostatic pressure [55, 56]. Many microorganisms that populate the deepest depths of the ocean are obligate piezophiles, requiring high hydrostatic pressures for optimal growth [55]. Notably, it has been shown that high pressure will decrease the p*K*a of many weak acids in biological systems, including carbonic acids, carboxylic acids, and dihydrogen phosphoric acid, which would release more free protons inside cells and decrease the intracellular pH [57, 58]. Thus the presence of V-ATPase with the function of proton pumping may offer fitness advantages for deep marine AOA under high-pressure conditions.

In the absence of a hadopelagic isolate, we could only examine the sensitivity of an A-ATPase-type coastal organism, *N. maritimus* SCM1, to high hydrostatic pressure (Fig. S12). Under the tested experimental conditions, cells were partially inhibited at 10 MPa (100 atmospheres) and completely inhibited at a 20 MPa (200 atmospheres) equivalent to the pressure at approximately 2000 m (Fig. S12), suggesting the inability of the coastal isolate to grow at greater depths. Given their environmental distribution, the A-type ATPase containing WCA-AOA are likely also piezosensitive.

Additional evidence for the possible function of the V-ATPase at elevated pressures come from studies of yeast, showing that high hydrostatic pressure promotes the dissociation of protons from carbonic acid and glycolytic intermediates [59]. The fungal V-ATPase was found to be associated with maintenance of pH homeostasis by pumping out of protons from the acidified cytoplasm into the vacuole [59]. In addition, a recent transcriptional study of the obligate deep sea piezophilic archaeon *Pyrococcus yayanosii* reported that the V-ATPase genes were among the most tightly regulated genes in response to changes in hydrostatic pressure, suggesting that they play a key role in piezophilic adaptation [60]. However, the isolation and characterization of additional marine AOA species, representing a broader range of ecotypes, will be essential to confirming a functional role of the V-type-like ATPase in adaptation to high-pressure environments.

### Horizontal transfer of the *atp* operon among *Thaumarchaeota*

The evolutionary radiation of *Thaumarchaeota* into low-pH and high-pressure habitats is correlated with a common deviation between ATPase and speciation phylogenies (Figs. 1 and 3). Maximum likelihood speciation phylogenies for ammonia-oxidizing *Thaumarchaeota* and the whole Archaea domain were constructed using concatenated alignments of 36 [38] and 122 [39] conserved marker genes (Figs. 1a, 3a, and S13) from 50 genomes of AOA and 158 genomes of *Euryarchaeota* as well as three archaeal superphyla, respectively (Table S3). The species trees based on the two different marker gene sets are highly consistent with each other (Figs. 1a, 3a, and S13). A complementary ATPase phylogeny was constructed based on a concatenated alignment of shared homologous subunits among archaeal ATPases (Figs. 1b and 3b). There is good congruence between the A-ATPase and the concatenated marker gene phylogenies of AOA members affiliated to the neutrophilic soil/hot spring groups and estuarine/coastal/shallow marine groups (Fig. 3). In contrast, phylogenetic relationships inferred from V-type-like ATPase and concatenated 36/122 conserved proteins for acidophilic/acid-tolerant *Thaumarchaeota* and presumptive piezophilic/piezotolerant *Thaumarchaeota* are discordant (Fig. 3). The ATPases of the latter group were found to be well separated from the lineage of soil neutrophilic *Thaumarchaeota* (Fig. 3b) and exclusively recovered as a well-supported monophyletic sister group of acidophilic *Euryarchaeota* (Fig. 3b). The ATPases of the four reported acidophilic *Nitrosotalea* species and those identified in thaumarchaeotal metagenomes recovered from acidic environments also affiliate with this lineage [36, 61] (Fig. 3b). The same observation was made for the acidophilic *Micrarchaeota*, *Parvarchaeota*, and recently identified acidophilic *Marsarchaeota* [62–64], and their ATPase-based phylogeny is incongruent with the corresponding species tree, being affiliated within the acidophilic/acid-tolerant lineage ATPases of *Thaumarchaeota* as well as *Thermoplasmatales* (Fig. 3).

Horizontal transfer of the entire *atp* operons (~8.9 kb in length) is the most parsimonious explanation for the presence of closely related V-type-like ATPases among distantly related archaeal taxa [65], including two distinctly related halophilic archaeal taxa, *Halobacteriales* and *Nanohaloarchaeota* (Fig. 3). Thus the phylogeny of acidophile V-type-like ATPase of these archaeal lineages tracks habitat, not organismal phylogeny. In contrast, the A-type ATPases of *Thaumarchaeota* were predominantly vertically inherited, following the general evolutionary history of speciation due to the congruence between A-ATPase and species tree (Fig. 3). *Thermoplasmatales* may have been the donor of V-ATPase in one or more of the other four archaeal phyla, because it has been reported that *Thermoplasmatales* exchanged dozens of other genes with archaeal partners in acidophilic environments [36, 66] (Fig. S14a), and their *atp* operons are flanked by genes encoding transposases and nucleases, key enzymes in mobilizing DNA fragments (Fig. 2). Our study provides evidence that horizontal transfer of V-type-like *atp* operon occurs widely in the Archaea domain, spanning the TACK and DPANN superphyla as well as *Euryarchaeota* phylum (Fig. 3), and the acquisition of V-ATPase via interphylum horizontal operon transfer (HOT) appears to play a significant role in the adaptive radiation of these archaeal species into a wide variety of habitats.

The AOA species tree was rooted with the A-type ATPase of the thermophilic *Nitrosocaldales*, suggesting that the last common ancestor of AOA harbored an A-type ATPase, and the V-type-like ATPase was later introduced into AOA by HOT (Fig. 1). An earlier analysis by Gubry-Rangin et al. [67] based on inferred changes in evolutionary rates also concluded that the split of the *Nitrosotaleales*/*Nitrosopumilales* lineage from the *Nitrososphaerales* lineage was coupled with expansion in low-pH environments. We postulate that, after the early divergence of the *Nitrosotaleales*/*Nitrosopumilales* lineage from the *Nitrososphaerales*, A- and V-ATPases have been frequently replaced in AOA via HOT (Fig. S14b). Notably, despite the frequent displacement of two distinct types of ATPases in *Nitrosotaleales* and *Nitrosopumilales* lineages (Fig. 1), their *atp* operons are always located between the genes encoding aspartate carbamoyltransferase and sulfurtransferase (Fig. 2). Both of these enzymes possess ATP-binding sites, and their enzyme activities are regulated in response to cellular ATP levels [68, 69], suggesting that they are possibly under the same transcriptional regulation as *atp* operons. Intriguingly, unlike the *atp* operons, the evolutionary histories of these neighboring genes are largely congruent with the AOA speciation history (Fig. S8b, c). Thus it appears that, in relatively recent HOT events, the thaumarchaeotal *atp* operons have undergone extensive in situ displacement with high precision between the conserved resident genes (Fig. 2). In this context, it is interesting to note that stand-alone Cas4 genes are located in the downstream flanking regions of *atp* operons, and these genes have been shown to be frequently recruited by archaeal viruses [70] (Fig. 2). Thus it is tempting to speculate that the diagnostic replacement of *atp* operons is associated with virus-mediated homologous recombination.

Our finding that single hadopelagic AOA species contain both types of ATPases (Fig. 2) is remarkable too, as it is strongly suggesting that extensive HOT of *atp* operons is a highly active and ongoing process within *Thaumarchaeota*. Consistent with the close phylogenetic relationship between *Nitrosopumilus*-like hadopelagic AOA and estuarine/coastal marine AOA, their A-type *atp* operons and flanking genes are highly conserved (Fig. S8b, c, Table S13). Thus A-ATPase is most likely the original ATPase type of hadopelagic AOA. In contrast, their V-type-like *atp* operons have unique neighboring genes (Fig. 2), suggesting that V-ATPase was acquired through relatively recent HOTs. The acquisition of V-ATPase is tightly correlated with the adaptive expansion of closely related *Nitrosopumilus*-like AOA populations from shallow waters to the high pressures of oceanic trenches. Unlike hadopelagic AOA, other AOA lineages only contain one type of ATPase (Fig. 2), thus in these AOA the acquisition of a variant ATPase via HOT was accompanied by the loss of their original type.

Overall, we show the occurrence of extensive interphylum HOT of V-ATPase across the Archaea domain, spanning the TACK and DPANN superphyla as well as *Euryarchaeota* phylum (Fig. 3). The acquisition of V-ATPase in ammonia-oxidizing *Thaumarchaeota* is closely associated with their adaptive radiation into acidic and higher-pressure habitats, which sheds new light on the major role of horizontal gene transfer in the evolutionary transitions of globally abundant AOA. Besides the HOT of V-ATPase, additional evolutionary processes associated with the energy-yielding ATPases were observed in multiple archaeal taxa. For instance, the position of *Bathyarchaeota* in the ATPase tree disagrees with their position in the species tree (Figs. 3 and S13). The ATPases of *Desulfurococcales* and *Sulfolobales* species lack subunit C [71], and *Desulfurococcales* members only contain seven subunits in their ATPase complex [71]. Furthermore, no canonical *atp* operon has been identified in most *Nanoarchaeota* in this study with the exception of *Nanoarcheaum equitans* that contains only five subunits [71, 72] (Fig. 3), and nearly all ATPases of other members in the DPANN superphylum were exclusively clustered with those of *Euryarchaeota* (Fig. 3), which might result from their severe genome reductions and symbiotic lifestyles with *Euryarchaeota* [64, 73]. The relationships between the high evolutionary dynamics of ATPases and the environmental adaptations of these archaea species remains a topic for future study. Finally, since our study has only emphasized the comparative phylogenetic analysis of a single gene cluster, we anticipate that the ongoing extensive molecular surveys and comprehensive comparative genomic studies of *Thaumarchaeota* will ultimately provide a much more complete mechanistic understanding of the evolution of some of the most successful organisms on Earth.

## Supplementary information

Supplementary information

Supplementary Figures

Supplementary Tables and DNA sequences
