# Supplementary material for: Expansion of Thaumarchaeota habitat range is correlated with horizontal transfer of ATPase operons
Source: ISME J. 2019 Aug 28;13(12):3067–79. doi: 10.1038/s41396-019-0493-x (PMC6863869; doi:10.1038/s41396-019-0493-x)
Supplement: Supplementary file 1 — Supplementary information [file 41396_2019_493_MOESM1_ESM.docx]

Supplementary Information for

**Expansion of *Thaumarchaeota* habitat range is correlated with horizontal transfer of ATPase operons**

Baozhan Wang^1,2^, Wei Qin^3^, Yi Ren^4^, Xue Zhou^1^, Man-Young Jung^2^, Ping Han^2^, Emiley A. Eloe-Fadrosh^5,6^, Meng Li^7^, Yue Zheng^8^, Lu Lu^1^, Xin Yan^9^, Junbin Ji^9^, Yang Liu^7^, Linmeng Liu^4^, Cheryl Heiner^10^, Richard Hall^10^, Willm Martens-Habbena^11^, Craig W. Herbold^2^, Sung-keun Rhee^12^, Douglas H. Bartlett^5^, Li Huang^13^, Anitra E. Ingalls^3^, Michael Wagner^2, 14^, David A. Stahl^15^, Zhongjun Jia^1^

Zhongjun Jia

Email: Jia@issas.ac.cn

**This file includes:**

Supplementary materials and methods

Supplementary results and discussion

References

Figures S1 to S14

Tables S1 to S13 and the synthesized DNA sequence of the V-type *atp* operon (separate file).

**Supplementary material and methods**

**Growth pH determination by ^15^N isotope tracing**

To determine the pH response of *Thaumarchaeota* in soil, 10 g of TS and FS soil were suspended in 50 ml 10 mM phosphate buffer adjusted to pH 4.5 and pH 7.0, amended with 200 μg ^15^N-urea (^15^N atom, 98% excess) g^-1^ dry weight soil (d.w.s) and incubated in the dark for 7 days at 25°C (Fig. S1). Ammonia oxidation activity was monitored by production of ^15^N-labeled nitrate plus nitrite as previously described ([1](#_ENREF_1)). Growth of thaumarchaeotal and bacterial ammonia oxidizers was determined by real-time PCR quantification of thaumarchaeotal and bacterial *amoA* gene copy numbers as described before ([2](#_ENREF_2)) using the primer pairs Arch-amoAF/Arch-amoAR ([3](#_ENREF_3)) and amoA1F/amoA2R ([4](#_ENREF_4)), respectively (Table S2). Real-time PCR was carried out on a CFX96 optical real-time detection system (Bio-Rad Laboratories, Inc., Hercules, CA). Each reaction contained 2 μl DNA template, 0.5 μM each primer and 10 μl SYBR Premix Ex Taq (TaKaRa, Dalian, China) in a 20 μl reaction volume. Thermal cycling was performed as follows: 95°C for 3min, followed by 38 cycles of 30 s at 95°C, 30 s at 55°C, 45 s at 72°C and plate read at 83°C. Plasmid DNA from one representative clone containing a thaumarchaeotal or bacterial *amoA* gene was used to generate a 10-fold serial dilution of the standard. Real-time PCR of each sample was conducted in biological triplicates, and each contained three technical replicates. Amplification efficiencies were 95.2%-108.5% with *r^2^* values of 0.990-0.996.

**^13^CO_2_- DNA-SIP microcosms**

In order to identify active *Thaumarchaeota* in acidic soils, DNA-SIP microcosms were constructed as previously described ([5](#_ENREF_5)). Briefly, three sets of treatments were performed, including ^13^CO_2_-labeled microcosms, ^12^CO_2_ control microcosms and ^13^CO_2_ + C_2_H_2_ control microcosms. For each treatment, 10.0 g of fresh soil was placed in a 120-ml serum bottles tightly sealed with a rubber stopper, and the headspace was adjusted to 5% (v/v) of either ^13^CO_2_ or ^12^CO_2_. The microcosms were amended with 100 μg urea-N g-1 *d.w.s.* weekly and incubated with 60% soil maximum water-holding capacity at 25°C in the dark for eight weeks. Total DNA of each treatment was extracted at day-56 by using a FastDNA Spin Kit for Soil (MP Biomedicals, Cleveland, OH, USA) and then subjected to isopycnic density gradient centrifugation ([5](#_ENREF_5)). In total, fourteen DNA gradient fractions (~360 µl each) were recovered from each 5.1 ml ultracentrifugation tube, and then the DNA was separated from CsCl by PEG6000 precipitation ([6](#_ENREF_6)), dissolved in 30 µl of TE buffer and stored at -20°C for further analysis.

To verify the labeling efficiency of thaumarchaeotal genomic DNA by ^13^CO_2_, a real-time PCR of thaumarchaeotal *amoA* genes across the entire buoyant density gradient of the DNA-SIP fractions for different treatments of the forest soil and the tea orchard soil after a 56-day incubation was conducted as described above (Table S2). Amplification efficiencies were 96.5%-106.2% with *r^2^* values of 0.992-0.998. Furthermore, the thaumarchaeotal *amoA* genes in the ^13^C-labeled DNA (fractions 4-6, 1.730-1.740 g ml^-1^) from ^13^CO_2_-labeled microcosms of the forest and tea orchard soils were also amplified for clone library construction using the primer pair of Arch-amoAF/Arch-amoAR as described before ([3](#_ENREF_3), [5](#_ENREF_5)) (Table S2). Triplicate PCR products were pooled, purified and inserted into pEASY-T3 vector (TransGen Biotech, Beijing, China), and then transformed into *Escherichia coli* JM109-competent cells. Sequencing of clones was performed by the Invitrogen Sequencing Department (Invitrogen, Shanghai, China).

**MDA of ^13^C-labeled genomic DNA**

MDA of ^13^C-labeled DNA (fractions 4-6, 1.730-1.740 g ml^-1^) from DNA-SIP microcosms was carried out using the REPLI-g Single Cell Kit (QIAGEN, Hilden, Germany) according to the manufacturer’s protocol with minor modifications. The 50μl reactions were carried out at 30°C. Between 1-3 ng of ^13^C-labeled DNA template was used to minimize amplification bias ([7](#_ENREF_7)). Following an initial 2-hour MDA reaction, amplification efficiency and MDA yield were determined by analyzing 1-µl MDA reaction aliquots every 20 minutes by gel electrophoresis on 1% w/v agarose gels. After a total of ~180-240 min run time and a MDA-generated DNA concentration of ~80-100 ng μl^-1^, the reactions were inactivated at 65°C for 3 min, stored at -20°C until further analysis by metagenomic sequencing. Potential amplification bias of the MDA was evaluated by pyrosequencing analysis of 16S rRNA genes of the ^13^C-labeled DNA before and after MDA as described before ([2](#_ENREF_2)). Briefly, DNA samples were amplified by using 16S rRNA gene universal primers 515F and 907R ([8](#_ENREF_8)) (Table S2), which were fused with A or B adapters, key sequence and sample-specific barcode for each DNA sample ([2](#_ENREF_2)). PCR reaction was performed in a 50 μl mixture containing 2 μl template DNA, 0.5 μM each primer and 45 μl Platinum PCR SuperMix (Invitrogen, Shanghai, China). Amplification conditions were: 94°C for 5 min, followed by 28 cycles of 45 s at 94°C, 30 s at 55°C, and 45 s at 72°C, followed by extension at 72°C for 5 min. The triplicate amplicons were pooled and size-selected by electrophoresis on 1.8% agarose gels and purified by AxyPrep DNA gel extraction kit (Axygen, USA). The concentration of purified PCR products was quantified using Picogreen (Invitrogen, Shanghai, China). PCR amplicons were combined in equimolar ratios into a single tube for subsequent pyrosequencing analysis on a Roche 454 GS FLX Titanium sequencer (Roche Diagnostics Corporation, Branford, CT, USA). The pyrosequencing data were processed using the MOTHUR software package version 1.22 ([9](#_ENREF_9)). Briefly, the sequences were sorted by sample-specific barcodes, and only those longer than 300 bp with an average quality score higher than 30 and without ambiguous base call were collected. Sequence chimeras were then detected and removed using chimera.uchime and remove.seqs commands within MOTHUR. The taxonomy of the high-quality sequences of each sample was classified using Silva release 119 as reference database with a bootstrap cut-off value of 80 ([10](#_ENREF_10)).

**Metagenomic sequencing of ^13^C-labeled DNA**

Samples from DNA-SIP experiments (site 1) and bulk soil DNA (site 2) were analyzed by metagenomic sequencing. The ^13^C-labeled DNA (fractions 4-6, 1.730-1.740 g ml^-1^) from DNA-SIP experiments of site 1 forest soil before and after MDA were both analyzed by metagenomic sequencing on the Illumina HiSeq 2000 platform using 150-bp paired-end library at the Qingdao Institute of BioEnergy and Bioprocess Technology, Chinese Academy of Sciences. Briefly, a DNA library with ~300bp insert size was prepared by using TruSeq DNA Sample Prep Kit (Illumina) according to the manufacturer’s recommendations. The HiSeq Rapid Duo cBot Sample Loading Kit and HiSeq Rapid SBS Kit v2 (Illumina) were used for sequencing according to standard protocols of the manufacturer. Totally, ~3.78 and ~3.86 Gbp 150bp paired-end Illumina data were obtained for original ^13^C-labeled DNA and MDA-product of ^13^C-labeled DNA from DNA-SIP experiments of the site 1 forest soil, respectively. The adaptors of reads were stripped by SeqPrep (<https://github.com/jstjohn/SeqPrep>). All raw reads were firstly trimmed to 120 bp based on the quality scores across all bases, then were quality trimmed and filtered using Sickle (<https://github.com/najoshi/sickle>) with default parameters. A total of ~3.01 and ~3.09 Gbp of high-quality Illumina sequence data were obtained from the original ^13^C-labeled DNA and MDA-generated DNA, respectively, and subsequently used for metagenomic binning and assembly.

For Site 1 tea orchard soil, the ^13^C-labeled DNA MDA products were sequenced on both the Illumina MiSeq platform at Majorbio Bio-pharm Technology Co., Ltd (Shanghai, China) and the PacBio RS II platform at Pacific Biosciences (California, USA). A paired-end library with an insert size of ~350bp was built using the TruSeq DNA Sample Prep Kit (Illumina), and then was sequenced using MiSeq Reagent Kit v2 and MiSeq Reagent Kit v3 following the MiSeq standard protocols of the manufacturer. Approximately 3.49 Gbp of 250bp paired-end and ~8.91 Gbp 300bp paired-end raw Illumina MiSeq data were obtained. Adaptors were stripped and reads were quality trimmed as described above. A total of ~10.6 Gbp of high quality Illumina MiSeq data were used for the following metagenomic analysis. Since the ^13^C-labeled DNA MDA product size ranged from ~10-20 kb, no shearing was required for thee PacBio SMRTbell library preparation of the DNA samples. Thus, a primer-annealed SMRTbell library of the DNA sample was directly created using the SMRTbell Template Prep Kit 1.0 according to protocol of the manufacturer. The SMRTbell templates with length of ~10-20 kb were selected, and fragments with size <5kb were removed using BluePippin system (Sage Science, Inc., Beverly, MA, USA). The library was sequenced on PacBio RS II platform at Pacific Biosciences (California, USA). About 8.0 Gbp PacBio data were obtained from PacBio RS II sequencing for subsequent metagenomic assembly.

DNA for metagenomic sequencing of acidic forest bulk soil from site 2 was extracted by using a FastDNA Spin Kit for Soil (MP Biomedicals, Cleveland, OH, USA). A paired-end sequencing library was built with an insert size of 300 bp using TruSeq DNA Sample Prep Kit (Illumina), then was sequenced by cBot TruSeq PE Cluster Kit v3-cBot-HS and HiSeq SBS Kit 4 (Illumina) according to standard protocols of manufacturer on Illumina HiSeq 2500 platform at the Majorbio Bio-pharm Technology Co., Ltd (Shanghai, China). About 11.47 Gbp of 125bp paired-end raw Illumina data was obtained. After adaptors striping with SeqPrep (<https://github.com/jstjohn/SeqPrep>) and quality trimming and filtering with Sickle (<https://github.com/najoshi/sickle>) using default parameters, ~10.76 Gbp clean Illumina data was left for subsequent metagenomic binning.

**Genome binning and annotation**

A thaumarchaeotal genome was reconstructed directly from site 1 forest soil ^13^C-DNA-SIP metagenome. Owing to the high abundance of enriched *Thaumarchaeota* in the SIP sample after 56 days incubation and its low population heterogeneity, the filtered high quality Illumina reads could be directly assembled using Velvet ([11](#_ENREF_11)), with a k-mer size 31, -exp_cov 60, -cov_cutoff 15. A putative *Thaumarchaeota* genome (FS) containing 312 contigs was reconstructed (Table S4). The contigs of the assemble mainly fell into ~40% GC content bin, which is in concordance with the G+C content of the marker genes of *Thaumarchaeota* in our sample. For the ^13^C-labeled DNA sequencing of site 1 tea orchard soils, the filtered Illumina reads were used to correct the high raw read errors of the PacBio by a correction pipeline, ECTools ([12](#_ENREF_12)). After error correction, PacBio long reads mainly fell into two bins of ~40% and 70% GC content. Phylogenetic analysis suggested that the reads of *Thaumarchaeota* were found in the low GC cluster. Assembly of the PacBio long reads of ~ 40% GC was conducted with the HGAP2 protocol by Pacific Biosciences (California, USA). A *Thaumarchaeota* genome (TS) containing 24 contigs were reconstructed from ^13^C-labeled DNA metagenomics of site 1 tea orchard soil (Table S4).

For the total DNA-metagenomics of site 2 acidic forest soils from Yingtan City of China ([13](#_ENREF_13)), the filtered Illumina reads were assembled with MetaVelvet assembler ([14](#_ENREF_14)). The contigs were binned based on both coverage and GC content distributions. Finally, a *Thaumarchaeota* genome (AFS) was reconstructed from a bin of ~40% GC content and ~120×coverage of diagnostic maker genes (Table S4).

**Genome completeness estimation and AAI determination.**

Genome completeness and contamination of three *Thaumarchaeota* genomes from this study were estimated using comparison of 145 lineage-specific marker genes (103 lineage-specific collocated marker gene sets) of archaea by the CheckM software ([15](#_ENREF_15)). The AAI ([16](#_ENREF_16)) was calculated as in Herbold et al. ([17](#_ENREF_17)). Briefly, bidirectional best hits were evaluated on gene pairs that aligned over 70% of length with at least 30% amino acid identity and gene length was used to calculate a weighted average % identity.

**Gene annotation and comparative genomics**

The coding sequences were predicted using Glimmer 3.0 ([18](#_ENREF_18)). Functional annotation was performed by BLASTP (BLAST 2.2.28+) searches against NCBI Non-redundant database, STRING ([19](#_ENREF_19)), KEGG databases ([20](#_ENREF_20)). Putative transporters were identified using Transporter Classification Database (TCDB) ([21](#_ENREF_21)).

Known AOA genomes from the orders *Nitrosophaerales*, *Nitrosopumilales* and *Ca.* Nitrosotaleales share a total of 743 core genome gene families ([17](#_ENREF_17)). The presence of these core genes families in the FS and TS metagenome-assembled genomes (MAGs) was determined by BLASTP with >45% amino acid identity and an e-value cutoff of 10e^-5^. Specific genes that were present in the FS and TS AOA MAGs but absent in neutrophilic *Thaumarchaeota* of the orders *Nitrosophaerales* and *Nitrosopumilales* were also determined. Each putative gene in TS and FS AOA MAGs was blasted against the AOA genomes within the order *Nitrosophaerales* and *Nitrosopumilales* (Table S3). Putative genes without any hit of at least 25% amino acid identity and an e-value cutoff of 10e^-5^ were collected and then manually identified as specific gene for TS and/or FS AOA MAGs.

**Transcriptional activity of acid-tolerant strain MY3 at different pH.**

*Ca.* Nitrosocosmicus oleophilus strain MY3 was incubated aerobically in an artificial fresh water medium (AFM) (pH 7.0) containing 500 μM ammonium chloride at 25°C in the dark without shaking ([22](#_ENREF_22)). Mid-exponential phase cells were then transferred into fresh AFM medium by 100-fold dilution, and the pH of the fresh AFM medium was adjusted to 5.2 and 5.5 by HOMOPIPES buffer and 7.5 by HEPES buffer, respectively ([23](#_ENREF_23)). Each pH treatment had three replicates. Ammonium and nitrite concentrations were determined using a Skalar SAN Plus segmented flow autoanalyzer (Skalar, Breda, the Netherlands) as described before ([5](#_ENREF_5)). To determine growth of MY3, SYBR Gold-stained cells were directly counted on 0.2 µm polycarbonate GTTP membranes (Merck Milipore, Germany) by fluorescence microscopy ([23](#_ENREF_23)). Specific growth rates were calculated by determining the slope according to the equation μ = (ln*N*_1_ – ln*N*_0_)/(t_1_ – t_0_), where (ln*N*_1_ – ln*N*_0_) is the change in the natural log of oxidation of ammonia concentration and (t_1_ – t_0_) is the change in time. At least four points in time were used for each growth rate calculation.

For analysis of transcription of the ATPase operon and other selected genes of strain MY3 at different pH conditions, total RNA was extracted from 500 ml of an exponentially growing culture of each replicate at pH 5.5 and 7.5, respectively, by using the RNeasy Mini Kit according to manufacturer’s protocol (Qiagen, Germany). cDNA was synthesized from the total RNA using the SuperScript First Strand synthesis system (Invitrogen, San Diego, CA) with RNaseOUT solution (40 U μL^-1^; Invitrogen) according to manufacturer’s instruction. The quantity of RNA and cDNA were determined by a Nanodrop ND-100 UV-vis Spectrophotometer (NanoDrop Technologies, Wilmington, DE). Transcript copy numbers of the *ntpA* gene (encoding ATPase subunit A, NMY3_02738), the 16S rRNA gene (NMY3_00918), the *amoA* gene (NMY3_03280), the 4-hydroxybutyrl-CoA dehydratase gene (NMY3_03315) and the methylmalonyl-CoA mutase large subunit gene (NMY3_02531) were quantified by real-time PCR in triplicates using the iQ SYBR Green Supermix kit on a C1000 CFX96 real-time PCR system (Bio-Rad). Each reaction contained 1 μl of template cDNA solution, 0.5 μM of each primer and 10 μl of iQ SYBR Green Supermix in a 20 μl reaction volume. A negative control was run with sterile water instead of template cDNA. The primers and thermal protocol are described in Table S2. The end point PCR product of the *ntpA* gene, 16S rRNA gene, *amoA* gene, 4-hydroxybutyrl-CoA dehydratase gene and methylmalonyl-CoA mutase large subunit gene were used as standard for qPCR (copy number in the standard was calculated from DNA concentration). 10-fold serial dilutions of the standards were prepared in triplicates to generate an external standard curves. The amplification efficiencies of qPCR were 87-95% with *r^2^* values > 0.99 for all assays. Finally, the cDNA transcripts of each gene were quantified per 1 ng of RNA for the normalization and comparison of each gene between pH 5.5 and 7.5.

**Pressure experiment of coastal marine strain SCM1**

Cultures of *N. maritimus* SCM1 were maintained aerobically at 28°C in glass Balch tubes in HEPES-buffered Synthetic Crenarchaeota Media (pH 7.5) containing ~0.6-0.8 mM ammonium chloride ([24](#_ENREF_24), [25](#_ENREF_25)). Mid-exponential phase cells were used for inoculating 100-fold dilution into fresh media for pressure experiments. Pressure experiments were performed using 4.5-ml polyethylene transfer pipette bulbs (Samco) without air space, heat sealed with a handheld heat-sealing clamp (Nalgene), and incubated at atmospheric (ATM, 0.1 MPa) and high-hydrostatic pressure in stainless-steel pressure vessels as described previously ([26](#_ENREF_26)). No differences in growth, cell densities, or ammonia uptake/nitrite production were observed between control cultures maintained in glass Balch tubes with air space and cultures maintained in polyethylene transfer bulbs without air space (data not shown). Thus, four sets of pressure treatments were performed in the polyethylene transfer bulbs without air space, that is, atmospheric pressure (ATM), 10 MPa (corresponding to ~1000m depth water pressure), 20 MPa and ‘ATM + 20 MPa’ (transferred to 20 MPa condition after incubation at atmospheric pressure for 10 days). Concentrations of ammonium and nitrate were determined using a Skalar SAN Plus segmented flow analyzer (Skalar, Breda, the Netherlands) as described previously ([5](#_ENREF_5)). The DAPI-stained cells of SCM1 were directly counted on filter by fluorescence microscopy as described previously ([27](#_ENREF_27)). Growth rates were calculated by determining the slope according to the equation μ = (ln*N*_1_ – ln*N*_0_)/(t_1_ – t_0_), where (ln*N*_1_ – ln*N*_0_) is the change in the natural log of cell density and (t_1_ – t_0_) is the change in time. Eight time points were used for each growth rate calculation.

**Statistical analysis**

SPSS for Windows version 24.0 was applied for data analysis. An independent-sample *t* test was performed to assess the possibility of significance between two groups, and *P* values less than 0.05 were considered to be statistically significant. All the experiments were conducted in triplicate.

**Supplementary results and discussion**

**The acidophilic growth of *Thaumarchaeota* in the two acidic soils**

Growth of ammonia oxidizers at pH 4.5 in acidic forest and tea orchard soils was demonstrated by concurrence increases in cell numbers and ^15^N-nitrate after stimulation with ^15^N-ammonia (Fig. S3a and b). Following seven days incubation at pH 4.5, AOA abundance as inferred from *amoA* gene counts increased from 5.1 × 10^7^ to 7.8 × 10^7^ copies g^-1^ *d.w.s* (*P*<0.05) and from 1.0 × 10^7^ to 2.4 × 10^7^ copies g^-1^ *d.w.s* (*P*<0.05) in the forest and tea orchard soils, respectively (Fig. S3b). During this period soil ^15^N-NO_3_^-^ increased from 0.09 to 2.23 (forest) and to 1.18 μg (tea orchard) ^15^N-NO_3_^-^ g^-1^ *d.w.s* (*P*<0.05). The putative cell-specific rate of ammonia oxidation was about ~0.011-0.018 and ~0.019-0.046 fmol N per cell h^-1^ for the AOA in the forest soil and tea orchard soil, respectively. These rates were lower than those previously reported for the pure culture of *Ca.* Nitrosotalea devanaterra Nd1, 0.072 fmol N per cell h^-1^ ([28](#_ENREF_28), [29](#_ENREF_29)), and this difference might reflect a slight inhibition of the nitrification activity in the soils by the added phosphate buffer. However, no production of ^15^N-NO_3_^-^ or increase in AOA *amoA* gene abundance was observed when soils were incubated at pH 7.0 (Fig. S3a and b). Bacterial *amoA* genes were not detected in the two soils, as reported in our previous study ([30](#_ENREF_30)). Thus, ammonia oxidation appeared to be primarily drove by acidophilic *Thaumarchaeota*.

**MDA of ^13^C-labeled genomic DNA**

MDA of ^13^C-labeled DNA (fractions 4-6, 1.730-1.740 g ml^-1^) from DNA-SIP microcosms of the forest and tea orchard soils generated ~10 μg of DNA with sizes of ~5.0-15 kb (Fig. S2a and S3c and d). Pyrosequencing of 16S rRNA genes demonstrated that no major bias in community structure was caused by the MDA (Fig. S2b). AOA-related 16S rRNA genes accounted for 58.2% and 52.1% of the total 16S rRNA genes in template ^13^C-labeled DNA from DNA-SIP microcosms of the forest and tea orchard soils, respectively, which accounted for 56.3% and 55.6% of the total 16S rRNA genes in MDA-generated DNA, suggesting negligible amplification bias for MDA (Fig. S2b). Therefore, besides the ^13^C-labeled DNA of the forest soil, the MDA-generated DNA from the template ^13^C-labeled DNA of the forest soil and tea orchard soil were also collected for following metagenomic analyses.

**Ammonia oxidation and electron transfer**

The two genomes of acidophilic *Thaumarchaeota* (FS and TS) contain core genes that are involved in ammonia oxidation (Table S5). As expected genes annotated as *amoA*, *amoB* and *amoC* coding for the ammonia monooxygenase are present in FS and TS genomes, and a fourth potential membrane-associated subunit, *amoX*, is also linked to *amoA*. Interestingly, *amoA*, *B*, *C* and *X* genes in these two genomes are arranged in a similar gene order with members of the *Nitrosotalea* and the *Nitrosopumilus* clusters, but different from the *Nitrososphaera* cluster ([17](#_ENREF_17), [31](#_ENREF_31), [32](#_ENREF_32)) (Table S5). Like in all published thaumarchaeotal genomes, homologues for the canonical AOB-like hydroxylamine oxidoreductase (HAO) were not identified in the two acidophilic thaumarchaeotal genomes ([33](#_ENREF_33), [34](#_ENREF_34)) (Table S5). However, published physiological and stable isotope tracer analyses provided compelling evidence that NH_2_OH is indeed as an intermediated in ammonia oxidation by *Thaumarchaeota*, and the oxidation of NH_2_OH (i.e., the activity of HAO) is independent of AMO activity, suggesting the presence of noncanonical HAO genes in thaumarchaeotal genomes ([35](#_ENREF_35)). Thus, it was speculated that hydroxylamine oxidation may be catalyzed via one of the soluble periplasmic multicopper oxidase (MCO) proteins (CuHAO) ([33](#_ENREF_33)). Like most other AOA, FS and TS encode an NO-forming nitrite reductase protein with two cupredoxin domians (Table S5), which was found to be strongly expressed during ammonia oxidation ([22](#_ENREF_22)) and might play a key role in *Thaumarchaeota* nitrification. Interestingly, up to now no NO reductase could be detected in AOA, and thus the production of the greenhouse gas nitrous oxide (N_2_O) by *Thaumarchaeota* cultures representing different clades ([36](#_ENREF_36), [37](#_ENREF_37)) is attributed to chemical formation from the intermediate hydroxylamine. Furthermore, the genes encoding the subunits of urease were detected in the genome of FS (Table S5), which was consistent with the growth of FS on urea during the incubation of DNA-SIP microcosms ([38](#_ENREF_38)). However, urease genes in the TS genome and cyanase genes in the FS and TS genomes were not detected ([38](#_ENREF_38))., but as these genomes are not closed their absence cannot be proven (Table S4).

Furthermore, FS and TS genomes also encode a complete respiratory chain complexes consisting of complexes I-V, which is used for generation of a proton motive force (PMF) and reverse electron transport (Fig. S7a and Table S11). Similar to all published *Thaumarchaeota*, FS and TS lack homologs of cytochrome *c554* and *c_M_552* proteins, suggesting *Thaumarchaeota* may use alternative mechanisms for transferring electrons derived from hydroxylamine oxidation into the electron transport system ([33](#_ENREF_33)). Consistently, genes encoding small blue type (I) copper binding domains (similar to the plastocyanin/azurin family), that might substitute for cytochromes in archaeal energy metabolism ([39](#_ENREF_39)), were identified in the FS and TS genomes.

**Central carbon metabolism**

In agreement with all published *Thaumarchaeota* genomes, FS and TS contain all key genes of a modified 3-hydroxypropionate/4-hydroxybutyrate (3-HP/4HB) autotrophic carbon fixation pathway ([40](#_ENREF_40), [41](#_ENREF_41)) (Fig. S6 and Table S7). The 3-HP/4HB pathway has two parts: the first half is the two carboxylation reactions transforming acetyl-CoA to succinyl-CoA, the second half is a multistep sequence converting succinyl-CoA into two molecules of acetyl-CoA (Fig. S6). These two genomes encode key enzymes of the 3-HP/4HB pathway including acetyl/propionyl-CoA carboxylase (EC 6.2.1.2/3), methylmalonyl-CoA epimerase (EC 5.4.99.1), methylmalonyl-CoA mutase (EC 5.4.99.2) and 4-hydroxybutyrate dehydratase (EC 4.2.1.1.20), and all of them show highest similarity to the genes of *Nitrosopumilus* cluster (Fig. S6 and Table S7). However, the FS and TS genomes lack some homologs of canonical genes of 3-HP/4HB pathway in *Metallosphaera sedula* ([42](#_ENREF_42)). For instance, there are five enzymes involved in the stepwise transformation of malonyl-CoA to propionyl-CoA for *M. sedula*. The two genomes lack any homologs of these genes, but contains alternative acyl-CoA synthetases (EC 6.2.1.36), enoyl-CoA hydratases (EC 4.2.1.1.16), aldehyde dehydrogenases (EC 1.2.1.16) and alcohol dehydrogenases (EC 1.1.1.1) possibly fulfilling the same functions ([33](#_ENREF_33)) (Fig. S6 and Table S7).

In addition to 3-HP/4HB pathway, FS and TS genomes harbor enzymes for all steps of an oxidative tricarboxylic acid (TCA) cycle, but have no homologs of the genes encoding ATP citrate lyase, which is required for the reductive TCA cycle ([43](#_ENREF_43)). Therefore, it excludes the reductive TCA cycle as a pathway for carbon fixation ([34](#_ENREF_34)). Moreover, genes participating in gluconeogenesis and nonoxidative pentose phosphate pathways are also detected in the two genomes (Fig. S6 and Table S7). Notably, succinyl-CoA and acetyl-CoA generated by 3-HP/4HB carbon fixation pathway can directly enter the TCA cycle ([44](#_ENREF_44)). During autotrophic growth, the synthesis of fructose-6-phosphate via gluconeogenesis pathway can also start from succinyl-CoA and acetyl-CoA. Succinyl-CoA can be oxidized to malate and/or oxaloacetate, and then oxaloacetate is converted into phosphoenolpyruvate (PEP) by oxidative TCA cycle (Fig. S6).

**General pH homeostasis mechanisms of acidophilic AOA**

FS and TS genomes also possess a set of genes, which are proposed to be involved in pH homeostasis (Fig. S7 and Table S8-10). That includes specific genes potentially for highly impermeable cell membrane, which can protect acidophiles from proton invasion ([45](#_ENREF_45), [46](#_ENREF_46)) (Fig. S7 and Table S8-9). They also harbor genes coding for a Na^+^/H^+^ antiporter and a variety of cation transporters, which facilitate the extrusion of cytosolic H^+^ and uptake of extracellular cations that generate an inside positive potential of Δψ to inhibit proton influx ([47](#_ENREF_47), [48](#_ENREF_48)) (Fig. S7 and Table S8-10). The gene encoding arginine decarboxylase, which can consume intracellular protons through amino acid decarboxylation, was also detected in the genomes of FS and TS ([49](#_ENREF_49)) (Fig. S7b). Moreover, acidophilic *Thaumarchaeota* might rely on transmembrane PMF for uptake of small organic acids that stimulate the growth of *Ca.* Nitrosotalea spp., since the genes of the H^+^/solute symporter were consistently detected among *Nitrosotalea* strains ([17](#_ENREF_17), [50](#_ENREF_50)) (Fig. S7 and Table S10). However, with the exception of the genes involved in synthesis of complex cell membrane and of the H^+^/solute symporter, most of the genes mentioned above are also present in at least some neutrophilic *Thaumarchaeota* (Fig. S7b), indicating that they might not be crucial for pH homeostasis of *Ca*. *Nitrosotalea* spp..

**V-ATPases of the putative acid-tolerant *Nitrososphaera*-like AFS**

For additional metagenomic comparison, another metagenome-assembled *Nitrososphaera*-like genome was recovered from an acidic forest soil of pH 4.35 in site 2, that is, AFS (2.34Mb) with 93.5% genomic completeness and 2.18% contamination estimated by CheckM ([15](#_ENREF_15)) (Fig. S4 and Table S4). The ANI between the genomes of AFS and the genomes of *Nitrososphaera* species, including *N. viennensis*, *Ca.* N. gargensis, and *Ca.* N. evergladensis ([51-53](#_ENREF_51)), were only ~56.1-57.3%, which was below the genus delineation range of 60-80% ([54](#_ENREF_54)) (Fig. S5). It indicated that the AFS might represent a novel genus of the Nitrososphaeraceae. Intriguingly, the ATPase of AFS also clustered within the monophyletic group of the V-ATPases of acidophilic lineage ([55](#_ENREF_55)) (Fig. 1, and Fig. S8). These results suggest that only AFS-like members of the Nitrososphaeraceae encoding a V-ATPase might thrive in acidic environments and it is tempting to speculate that the Nitrosophaeracea-like *amoA* genes previously detected in acidic soils ([56-60](#_ENREF_56)) also originate from members possessing a V-ATPase. Therefore, it further enhances the conclusion that V-ATPases of acidophilic/acid-tolerant AOA track niche preference, not organismal phylogeny (Fig. 1, and Fig. S8), and might play an important role in adaptation to acidic environments.

**Comparative structural analysis of thaumarchaeotal V-ATPases**

Subunit H/G is one of the essential components of the peripheral stalks (i.e. 2 to 3 EG heterodimers) for prokaryotic and eukaryotic V-ATPases ([55](#_ENREF_55), [61](#_ENREF_61)) (Fig. S11a and Table S12). Recent crystal structural studies showed that EG heterodimers were folded in a unusual right-handed coiled-coil architecture containing a partially disordered ‘bulge’ region in subunit H/G of the V-ATPases ([62-64](#_ENREF_62)), which provided flexibility for conformational changes in peripheral stalks and play an important role in regulating assembly and disassembly of V-ATPases ([62](#_ENREF_62), [63](#_ENREF_63), [65](#_ENREF_65), [66](#_ENREF_66)) (Fig. S11a). Moreover, the regulation mechanism of V-ATPase could be stimulated by different cellular pH ([67](#_ENREF_67)) and ATP/ADP levels ([68](#_ENREF_68)). Therefore, we presume that the common subunit composition shared by ATPases of acidophilic *Thaumarchaeota* and proton/ion pumping V-type ATPases of *S. cerevisiae* and *E. hirae*, particularly, the presence of subunit H/G, could facilitate a conformational change and stimulate the ATP-driven proton pumping of V-ATPases of AOA in response to changes in cellular pH and ATP/ADP levels in acidic environments.

**Diagnostic catalytic sites of thaumarchaeotal V-ATPases**

The hydrophilic catalytic A_1_/V_1_/F_1_ domain of A/V/F-ATPases comprises three catalytic nucleotide binding A/β subunits and three non-catalytic nucleotide binding B/α subunits, which alternatively arranged and form a hexagonal complex ([55](#_ENREF_55), [69](#_ENREF_69), [70](#_ENREF_70)) (Fig. S11a and Table S12). In this study, we found that V-ATPases of acidophilic/deep marine *Thaumarchaeota* and A-ATPases of neutrophilic/shallow marine *Thaumarchaeota* contained highly conserved ATP-binding sites of V-type ATP hydrolase in *E. hirae* ([71](#_ENREF_71)) and *S. cerevisiae* ([72](#_ENREF_72)), including a ‘P-loop’ (G^235^XXXXGKTV^240^) and N-terminal portion of the arm (E^261^ and R^262^) in subunit A, and the ‘Arg-finger’ (R^350^) in subunit B (Fig. S11d). However, they differ over the nucleotide binding Q^503^NAY^506^ motif and F^425^ in subunit A ([71](#_ENREF_71)), and the site^155^ of subunit B that is structurally adjacent to ATP-binding sties in subunit B and crucial for ATP-driven proton pumping activity of V-ATPases ([73](#_ENREF_73)). Recent crystallographic and single-molecule studies suggested that nucleotide-binding sites and related residues were involved in the transformations of ATP-hydrolysis-driven rotatory steps, and could significantly influence the affinities and binding rate of V-ATPases for ATP or ADP and phosphate ([74](#_ENREF_74), [75](#_ENREF_75)). The replacement of ATP/ADP-binding site from a hydrophobic amino acid (A/G^505^) in subunit A of V-ATPases to a hydrophilic residue (S^505^) of A-ATPases would be expected to affect the kinetic parameters of V-ATPase for hydrolysis reaction (Fig. S11b-d), but which still need further site-directed mutagenesis investigation. However, it had been shown that proton pumping activity driven by ATP hydrolysis decreased by ~50%, when L^155^ in subunit B was replaced by C^155^ (structural similar to Methionine (M) in A-ATPases of neutrophilic soil AOA) in the V-ATPase of *S. cerevisiae* ([73](#_ENREF_73)) (Fig. S11d). Therefore, compared to A-ATPases in neutrophilic AOA, ATP/ADP-binding domain of V-ATPases in the acidophilic AOA are more likely to induce ATP hydrolysis and proton pumping activity as V-type ATP hydrolase in *E. hirae* and *S. cerevisiae*.

**Proton selectivity of thaumarchaeotal V-ATPases**

It is generally accepted that the ATPases of *Thaumarchaeota* translocate H^+^ ions rather than Na^+^ ions ([33](#_ENREF_33), [34](#_ENREF_34), [69](#_ENREF_69)). We found that this most likely held true for the V-ATPases of acidophilic/acid-tolerant AOA as well (Fig. S10). The c/K ring consisting of 11-15 c/K-subunits within the A_0_/V_0_/F_0_ domain is responsible for ion transport of A/V/F-ATPases, and the H^+^ and Na^+^ selectivity of ATPases is determined by the primary structure of c/K rings ([76-78](#_ENREF_76)). The ion binding is consistently mediated by a conserved carboxylic side chain of Glu (marked by a star in Fig. S10) in subunit c/K regardless the types of ATPases, and inherent selectivity of this H^+^/Na^+^-binding site is largely set by the balance of flanked polar and hydrophobic side chains (highlighted in color in Fig. S10) ([76-78](#_ENREF_76)). Hydrophobic side chains in this site enhance H^+^ binding over Na^+^, and polar side-chains favor Na^+^ binding ([76-78](#_ENREF_76)). For instance, the replacement of two polar side chains T^67^ and Y^70^ in *I. tartaricus* to two hydrophobic side chains I^68^ and F^71^ in *M. acetivorans* increased the H^+^ over Na^+^ selectivity from 3.5-fold to ~10^11^-10^12^-fold ([78](#_ENREF_78), [79](#_ENREF_79)) (Fig. S10). It resulted in the concurrent translocation of H^+^ and Na^+^ of the ATPases in *M. acetivorans* from the obligate Na^+^ translocation in *I. tartaricus* under physiological conditions ([76-78](#_ENREF_76)) (Fig. S10). Compared to *M. acetivorans*, the conserved carboxylate E^97^ (site number referred to subunit K of *Nitrosotalea*-like FS ATPase) in the c/K rings of V-ATPases in acidophilic *Thaumarchaeota* are flanked by three hydrophobic and one polar side chains, that is, I^95^, L^99^, I^102^ and T^98^, corresponding to I^64^, I^68^, F^71^ and T^67^ in *M. acetivorans* (Fig. S10). However, the c/K ring of ATPase in acidophilic AOA uses a polar neutral side chain Q^68^ instead of an electrically charged side chain E^37^ in that of *M. acetivorans*, which could significantly increase hydrophobicity around the conserved E68 in c/K ring of V-ATPases in acidophilic AOA (Fig. S10). Therefore, the selectivity of H^+^ over Na^+^ of the V-ATPases in acidophilic AOA should be much higher than that of *M. acetivorans*, and might be similar to that of *S. platensis*, which also has three hydrophobic and two polar neutral side chains flanking the conserved carboxylate E^62^ and exclusively translocate H^+^ under physiological condition ([76](#_ENREF_76)) (Fig. S10). The same patterns are also found in the c/K subunits of the V-ATPases of deep marine AOA, acidophilic Micrarchaeota, Parvarchaeota and Thermoplasmatales, indicating translocation of H^+^ rather than Na^+^ for these V-ATPases (Fig. S10).

**The directionality of horizontal transfer of the V-ATPase operons.**

The directionality of horizontal gene transfer of the *atp* operon is difficult to constrain. However, the members of acidophilic *Parvarchaeota*, *Micrarchaeota*, and *Marsarchaeota* coexist with *Thermoplasmatales* species in the acid mine drainage and geothermal environments ([80](#_ENREF_80), [81](#_ENREF_81)). Specifically, it has been shown that the acidophilic *Parvarchaeota* and *Micrarchaeota* have physical interaction with *Thermoplasmatales* cells via pili-like structures ([80](#_ENREF_80), [82](#_ENREF_82)), might through which dozens of genes were transferred from *Thermoplasmatales* to *Micrarchaeota* ([83](#_ENREF_83)). In addition, a recent phylogenetic study reported that acidophilic *Nitrosotalea* ancestors acquired genes encoding a proton/solute symporter and two metal transporters from members of *Thermoplasmatales*, which presumably play a role in their adaptation to low pH environments ([17](#_ENREF_17)) (Fig. S7 and Table S10). Notably, we found that the *atp* operons of *Thermoplasma* representatives were flanked by genes encoding transposases and nucleases, the key enzymes that function in mobilizing DNA fragments between genomes, further suggesting the high potential of lateral transfer of *atp* operon from the lineage of *Thermoplasmatales* to other taxa (Fig. S14a).

**Supplementary Acknowledgments**

We are grateful to Prof. David Myrold at Oregon State University for bringing the DNA sample from China on dry ice to USA and shipping it on dry ice overnight from Oregon to Pacific Biosciences in California, USA. We thank Dr. George Yuan, Dr. Sian Loony Au, Dr. Xin Cai, Dr. Ram Laxman and Dr. Caroline Chan at Pacific Biosciences (California, USA), Dr. Fangqiao Lv for valuable discussion, Dr. Hong Wu at University of Vienna for bringing the DNA sample from Austria to China, Prof. Jian Xu and Dr. Xiaowei Zeng at the Qingdao Institute of BioEnergy and Bioprocess Technology, CAS for their help on metagenomic sequencing. We thank the assistance of Longxing Cao on ATPase 3D structure construction. We thank Dr. Junbin Ji and all students from Prof. Xin Yan’s team for the help on *E.coli* experiment.

**Supplementary References**

1. Zhang JB, Zhu TB, Cai ZC, Müller C. Nitrogen cycling in forest soils across climate gradients in Eastern China. *Plant Soil* 2011; **342**(1-2): 419-432.

2. Wang BZ, Zhao J, Guo ZY, Ma J, Xu H, Jia ZJ. Differential contributions of ammonia oxidizers and nitrite oxidizers to nitrification in four paddy soils. *ISME J* 2015; **9**(5): 1062-1075.

3. Francis CA, Roberts KJ, Beman JM, Santoro AE, Oakley BB. Ubiquity and diversity of ammonia-oxidizing archaea in water columns and sediments of the ocean. *Proc Natl Acad Sci USA* 2005; **102**(41): 14683-14688.

4. Rotthauwe J, Witzel K, Liesack W. The ammonia monooxygenase structural gene *amoA* as a functional marker: molecular fine-scale analysis of natural ammonia-oxidizing populations. *Appl Environ Microbiol* 1997; **63**(12): 4704-4712.

5. Lu L, Jia ZJ. Urease gene-containing Archaea dominate autotrophic ammonia oxidation in two acid soils. *Environ Microbiol* 2013; **15**(6): 1795-1809.

6. Freitag TE, Chang L, Prosser JI. Changes in the community structure and activity of betaproteobacterial ammonia-oxidizing sediment bacteria along a freshwater-marine gradient. *Environ Microbiol* 2006; **8**(4): 684-696.

7. Chen Y, Dumont MG, Neufeld JD, Bodrossy L, Stralis-Pavese N, McNamara NP *et al*. Revealing the uncultivated majority: combining DNA stable-isotope probing, multiple displacement amplification and metagenomic analyses of uncultivated Methylocystis in acidic peatlands. *Environ Microbiol* 2008; **10**(10): 2609-2622.

8. Stubner S. Enumeration of 16S rDNA of *Desulfotomaculum* lineage 1 in rice field soil by real-time PCR with SybrGreen^TM^ detection. *J Microbiol Meth* 2002; **50**(2): 155-164.

9. Schloss PD, Westcott SL, Ryabin T, Hall JR, Hartmann M, Hollister EB. Introducing mothur: open-source, platform-independent, community-supported software for describing and comparing microbial communities. *Appl Environ Microbiol* 2009; **75**: 7537-7541.

10. Quast C, Pruesse E, Yilmaz P, Gerken J, Schweer T, Yarza P *et al*. The SILVA ribosomal RNA gene database project: improved data processing and web-based tools. *Nucleic Acids Res* 2013; **41**(Database issue): D590-596.

11. Zerbino DR, Birney E. Velvet: Algorithms for de novo short read assembly using de Bruijn graphs. *Genome Res* 2008; **18**(5): 821-829.

12. Lee H, Gurtowski J, Yoo S, Marcus S, McCombie WR, Schatz M. Error correction and assembly complexity of single molecule sequencing reads. *bioRxiv* 2014.

13. Huang R, Wu YC, Zhang JB, Zhong WH, Jia ZJ, Cai ZC. Nitrification activity and putative ammonia-oxidizing archaea in acidic red soils. *J Soil Sediment* 2012; **12**(3): 420-428.

14. Namiki T, Hachiya T, Tanaka H, Sakakibara Y. MetaVelvet: an extension of Velvet assembler to de novo metagenome assembly from short sequence reads. *Nucleic Acids Res* 2012; **40**(20): e155.

15. Parks DH, Imelfort M, Skennerton CT, Hugenholtz P, Tyson GW. CheckM: assessing the quality of microbial genomes recovered from isolates, single cells, and metagenomes. *Genome Res* 2015; **25**(7): 1043-1055.

16. Konstantinidis KT, Tiedje JM. Towards a genome-based taxonomy for prokaryotes. *J Bacteriol* 2005; **187**(18): 6258-6264.

17. Herbold CW, Lehtovirta-Morley LE, Jung MY, Jehmlich N, Hausmann B, Han P et al. Ammonia-oxidising archaea living at low pH: Insights from comparative genomics. Environ Microbiol 2017; **19**(12): 4939-4952.

18. Delcher AL, Bratke KA, Powers EC, Salzberg SL. Identifying bacterial genes and endosymbiont DNA with Glimmer. *Bioinformatics* 2007; **23**(6): 673-679.

19. Szklarczyk D, Franceschini A, Wyder S, Forslund K, Heller D, Huerta-Cepas J *et al*. STRING v10: protein-protein interaction networks, integrated over the tree of life. *Nucleic Acids Res* 2015; **43**: D447-452.

20. Kanehisa M, Goto S. KEGG: kyoto encyclopedia of genes and genomes. *Nucleic Acids Res* 2000; **28**(1): 27-30.

21. Saier MH, Reddy VS, Tamang DG, Västermark A. The transporter classification database. *Nucleic Acids Res* 2014; **42**(D1): D251-D258.

22. Jung MY, Park SJ, Min D, Kim JS, Rijpstra WIC, Sinninghe Damsté JS *et al*. Enrichment and characterization of an autotrophic ammonia-oxidizing archaeon of mesophilic crenarchaeal group 1.1a from an agricultural soil. *Appl Environ Microbiol* 2011; **77**(24): 8635-8647.

23. Jung MY, Kim JG, Damste JSS, Rijpstra WIC, Madsen EL, Kim SJ *et al*. A hydrophobic ammonia-oxidizing archaeon of the Nitrosocosmicus clade isolated from coal tar-contaminated sediment. *Environ Microbiol Rep* 2016; **8**(6): 983-992.

24. Martens-Habbena W, Berube PM, Urakawa H, de la Torre JR, Stahl DA. Ammonia oxidation kinetics determine niche separation of nitrifying archaea and bacteria. *Nature* 2009; **461**(7266): 976-979.

25. Qin W, Amin SA, Martens-Habbena W, Walker CB, Urakawa H, Devol AH *et al*. Marine ammonia-oxidizing archaeal isolates display obligate mixotrophy and wide ecotypic variation. *Proc Natl Acad Sci USA* 2014; **111**(34): 12504-12509.

26. Eloe EA, Lauro FM, Vogel RF, Bartlett DH. The deep-sea bacterium *Photobacterium profundum* SS9 utilizes separate flagellar systems for swimming and swarming under high-pressure conditions. *Appl Environ Microb* 2008; **74**(20): 6298-6305.

27. Konneke M, Bernhard AE, de la Torre J, Walker CB, Waterbury JB, Stahl DA. Isolation of an autotrophic ammonia-oxidizing marine archaeon. *Nature* 2005; **437**: 543 - 546.

28. Lehtovirta-Morley LE, Ross J, Hink L, Weber EB, Gubry-Rangin C, Thion C *et al*. Isolation of ‘*Candidatus* Nitrosocosmicus franklandus’, a novel ureolytic soil archaeal ammonia oxidiser with tolerance to high ammonia concentration. *FEMS Microbiol Ecol* 2016; **92**: fiw057.

29. Lehtovirta-Morley LE, Stoecker K, Vilcinskas A, Prosser JI, Nicol GW. Cultivation of an obligate acidophilic ammonia oxidizer from a nitrifying acid soil. *Proc Natl Acad Sci USA* 2011; **108**(38): 15892-15897.

30. Lu L, Han WY, Zhang JB, Wu YC, Wang BZ, Lin XG *et al*. Nitrification of archaeal ammonia oxidizers in acid soils is supported by hydrolysis of urea. *ISME J* 2012; **6**(10): 1978-1984.

31. Jung MY, Park SJ, Kim SJ, Kim JG, Sinninghe Damsté JS, Jeon CO *et al*. A mesophilic autotrophic ammonia-oxidizing archaeon of the thaumarchaeal group I.1a cultivated from a deep oligotrophic soil horizon. *Appl Environ Microbiol* 2014; **80**(12): 3645-3655.

32. Spang A, Poehlein A, Offre P, Zumbrägel S, Haider S, Rychlik N *et al*. The genome of the ammonia-oxidizing *Candidatus* Nitrososphaera gargensis: insights into metabolic versatility and environmental adaptations. *Environ Microbiol* 2012; **14**(12): 3122-3145.

33. Walker CB, la Torre JRd, Klotz MG, Urakawa H, Pinel N, Arp DJ *et al*. *Nitrosopumilus maritimus* genome reveals unique mechanisms for nitrification and autotrophy in globally distributed marine crenarchaea. *Proc Natl Acad Sci USA* 2010; **107**(19): 8818-8823.

34. Hallam SJ, Mincer TJ, Schleper C, Preston CM, Roberts K, Richardson PM *et al*. Pathways of carbon assimilation and ammonia oxidation suggested by environmental genomic analyses of marine Crenarchaeota. *Plos Biol* 2006; **4**(4): 520-536.

35. Vajrala N, Martens-Habbena W, Sayavedra-Soto LA, Schauer A, Bottomley PJ, Stahl DA *et al*. Hydroxylamine as an intermediate in ammonia oxidation by globally abundant marine archaea. *Proc Natl Acad Sci USA* 2013; **110**(3): 1006-1011.

36. Kozlowski JA, Stieglmeier M, Schleper C, Klotz MG, Stein LY. Pathways and key intermediates required for obligate aerobic ammonia-dependent chemolithotrophy in bacteria and Thaumarchaeota. *ISME J* 2016; **10**(8):1836-1845.

37. Stieglmeier M, Mooshammer M, Kitzler B, Wanek W, Zechmeister-Boltenstern S, Richter A *et al.* Aerobic nitrous oxide production through N-nitrosating hybrid formation in ammonia-oxidizing archaea. *ISME J* 2014; **8**(5):1135-1146.

38. Palatinszky M, Herbold C, Jehmlich N, Pogoda M, Han P, von Bergen M *et al*. Cyanate as an energy source for nitrifiers. *Nature* 2015; **524**(7563): 105-108.

39. Cabello P, Roldan MD, Moreno-Vivian C. Nitrate reduction and the nitrogen cycle in archaea. *Microbiology* 2004; **150**: 3527-3546.

40. Kerou M, Offre P, Valledor L, Abby SS, Melcher M, Nagler M *et al*. Proteomics and comparative genomics of *Nitrososphaera viennensis* reveal the core genome and adaptations of archaeal ammonia oxidizers. *Proc Natl Acad Sci USA* 2016; **113**(49): E7937-E7946.

41. Stahl DA, de la Torre JR. Physiology and diversity of ammonia-oxidizing archaea. *Annu Rev Microbiol* 2012; **66**(1): 83-101.

42. Berg IA, Kockelkorn D, Buckel W, Fuchs G. A 3-hydroxypropionate/4-hydroxybutyrate autotrophic carbon dioxide assimilation pathway in Archaea. *Science* 2007; **318**(5857): 1782-1786.

43. Sintsov NV, Ivanovskii RN, Kondrat'eva EN. ATP-dependent citrate lyase in the green phototrophic bacterium, *Chlorobium limicola*. *Mikrobiologiia* 1980; **49**(4): 514-516.

44. Estelmann S, Hugler M, Eisenreich W, Werner K, Berg IA, Ramos-Vera WH *et al*. Labeling and enzyme studies of the central carbon metabolism in *Metallosphaera sedula*. *J Bacteriol* 2011; **193**(5): 1191-1200.

45. Wang XM, Lv BE, Cai GX, Fu L, Wu YZ, Wang X *et al*. A proton shelter inspired by the sugar coating of acidophilic archaea. *Sci rep* 2012; **2**: 892.

46. Shimada H, Nemoto N, Shida Y, Oshima T, Yamagishi A. Effects of pH and temperature on the composition of polar lipids in *Thermoplasma acidophilum* HO-62. *J Bacteriol* 2008; **190**(15): 5404-5411.

47. Aronson PS, Nee J, Suhm MA. Modifier role of internal H^+^ in activating the Na^+^-H^+^ exchanger in renal microvillus membrane vesicles. *Nature* 1982; **299**(5879): 161-163.

48. Baker-Austin C, Dopson M. Life in acid: pH homeostasis in acidophiles. *Trends Microbiol* 2007; **15**(4): 165-171.

49. Richard H, Foster JW. *Escherichia coli* glutamate- and arginine-dependent acid resistance systems increase internal pH and reverse transmembrane potential. *J Bacteriol* 2004; **186**(18): 6032-6041.

50. Lehtovirta-Morley LE, Sayavedra-Soto LA, Gallois N, Schouten S, Stein LY, Prosser JI *et al*. Identifying potential mechanisms enabling acidophily in the ammonia-oxidising archaeon ‘*Candidatus* Nitrosotalea devanaterra’. *Appl Environ Microbiol* 2016; **82**(9): 2608-2619.

51. Hatzenpichler R, Lebedeva EV, Spieck E, Stoecker K, Richter A, Daims H *et al*. A moderately thermophilic ammonia-oxidizing crenarchaeote from a hot spring. *Proc Natl Acad Sci USA* 2008; **105**(6): 2134-2139.

52. Zhalnina KV, Dias R, Leonard MT, Dorr de Quadros P, Camargo FAO, Drew JC *et al*. Genome sequence of *Candidatus* Nitrososphaera evergladensis from group I.1b enriched from everglades soil reveals novel genomic features of the ammonia-oxidizing archaea. *PLoS ONE* 2014; **9**(7): e101648.

53. Tourna M, Stieglmeier M, Spang A, Konneke M, Schintlmeister A, Urich T *et al*. *Nitrososphaera viennensis*, an ammonia oxidizing archaeon from soil. *Proc Natl Acad Sci USA* 2011; **108**(20): 8420-8425.

54. Luo CW, Rodriguez-R LM, Konstantinidis KT. MyTaxa: an advanced taxonomic classifier for genomic and metageomic sequences. *Nucleic Acids Res* 2014; **42**(8): e73-e73.

55. Mulkidjanian AY, Makarova KS, Galperin MY, Koonin EV. Inventing the dynamo machine: the evolution of the F-type and V-type ATPases. *Nat Rev Microbiol* 2007; **5**(11): 892-899.

56. Gubry-Rangin C, Hai B, Quince C, Engel M, Thomson BC, James P *et al*. Niche specialization of terrestrial archaeal ammonia oxidizers. *Proc Natl Acad Sci USA* 2011; **108**(52): 21206-21211.

57. He JZ, Shen JP, Zhang LM, Zhu YG, Zheng YM, Xu MG *et al*. Quantitative analyses of the abundance and composition of ammonia-oxidizing bacteria and ammonia-oxidizing archaea of a Chinese upland red soil under long-term fertilization practices. *Environ Microbiol* 2007; **9**(9): 2364-2374.

58. Wang BZ, Zheng Y, Huang R, Zhou X, Wang DM, He YQ *et al*. Active ammonia oxidizers in an acidic soil are phylogenetically closely related to neutrophilic Archaeon. *Appl Environ Microbiol* 2014; **80**(5): 1684-1691.

59. Pester M, Rattei T, Flechl S, Gröngröft A, Richter A, Overmann J *et al*. *amoA*-based consensus phylogeny of ammonia-oxidizing archaea and deep sequencing of *amoA* genes from soils of four different geographic regions. *Environ Microbiol* 2012; **14**(2): 525-539.

60. Gubry-Rangin C, Kratsch C, Williams TA, McHardy AC, Embley TM, Prosser JI *et al*. Coupling of diversification and pH adaptation during the evolution of terrestrial Thaumarchaeota. *Proc Natl Acad Sci USA* 2015; **112**(30): 9370-9375.

61. Forgac M. Vacuolar ATPases: rotary proton pumps in physiology and pathophysiology. *Nat Rev Mol Cell Biol* 2007; **8**(11): 917-929.

62. Oot RA, Huang LS, Berry EA, Wilkens S. Crystal structure of the yeast vacuolar ATPase heterotrimeric EGC_head_ peripheral stalk complex. *Structure* 2012; **20**(11): 1881-1892.

63. Oot RA, Kane PM, Berry EA, Wilkens S. Crystal structure of yeast V_1_-ATPase in the autoinhibited state. *Embo J* 2016; **35**(15): 1694-1706.

64. Stewart AG, Lee LK, Donohoe M, Chaston JJ, Stock D. The dynamic stator stalk of rotary ATPases. *Nat Commun* 2012; **3**: 687.

65. Zhao JH, Benlekbir S, Rubinstein JL. Electron cryomicroscopy observation of rotational states in a eukaryotic V-ATPase. *Nature* 2015; **521**(7551): 241-245.

66. Rahman S, Yamato I, Murata T. Function and Regulation of Mammalian V-ATPase Isoforms. In: Chakraborti S, Dhalla NS(eds). *Regulation of Ca^2+^-ATPases,V-ATPases and F-ATPases.* Springer International Publishing Switzerland: Springer, Cham, 2016, pp 283-299.

67. Parra KJ, Chan CY, Chen J. *Saccharomyces cerevisiae* vacuolar H^+^-ATPase regulation by disassembly and reassembly: one structure and multiple signals. *Eukaryot Cell* 2014; **13**(6): 706-714.

68. Zhou M, Politis A, Davies R, Liko I, Wu KJ, Stewart AG *et al*. Ion mobility-mass spectrometry of a rotary ATPase reveals ATP-induced reduction in conformational flexibility. *Nat chem* 2014; **6**(3): 208-215.

69. Gruber G, Manimekalai MS, Mayer F, Muller V. ATP synthases from archaea: the beauty of a molecular motor. *Biochim Biophys Acta* 2014; **1837**(6): 940-952.

70. Abrahams JP, Leslie AGW, Lutter R, Walker JE. Structure at 2.8 Â resolution of F_1_-Atpase from bovine heart-mitochondria. *Nature* 1994; **370**(6491): 621-628.

71. Arai S, Saijo S, Suzuki K, Mizutani K, Kakinuma Y, Ishizuka-Katsura Y *et al*. Rotation mechanism of *Enterococcus hirae* V_1_-ATPase based on asymmetric crystal structures. *Nature* 2013; **493**(7434): 703-707.

72. Nishi T, Forgac M. The vacuolar (H^+^)-ATPases-nature’s most versatile proton pumps. *Nat Rev Mol Cell Bio* 2002; **3**: 94.

73. Vasilyeva E, Liu Q, MacLeod KJ, Baleja JD, Forgac M. Cysteine scanning mutagenesis of the noncatalytic nucleotide binding site of the yeast V-ATPase. *J Biol Chem* 2000; **275**(1): 255-260.

74. Singharoy A, Chipot C, Moradi M, Schulten K. Chemomechanical coupling in hexameric protein-protein interfaces harnesses energy within V-Type ATPases. *J Am Chem Soc* 2017; **139**(1): 293-310.

75. Nakano M, Imamura H, Toei M, Tamakoshi M, Yoshida M, Yokoyama K. ATP hydrolysis and synthesis of a rotary motor V-ATPase from Thermus thermophilus. *J Biol Chem* 2008; **283**(30): 20789-20796.

76. Leone V, Pogoryelov D, Meier T, Faraldo-Gomez JD. On the principle of ion selectivity in Na^+^/H^+^-coupled membrane proteins: Experimental and theoretical studies of an ATP synthase rotor. *Proc Natl Acad Sci USA* 2015; **112**(10): E1057-E1066.

77. Murata T, Yamato I, Kakinuma Y, Shirouzu M, Walker JE, Yokoyama S *et al*. Ion binding and selectivity of the rotor ring of the Na^+^-transporting V-ATPase. *Proc Natl Acad Sci USA* 2008; **105**(25): 8607-8612.

78. Schlegel K, Leone V, Faraldo-Gomez JD, Muller V. Promiscuous archaeal ATP synthase concurrently coupled to Na^+^ and H^+^ translocation. *Proc Natl Acad Sci USA* 2012; **109**(3): 947-952.

79. Krah A, Pogoryelov D, Langer JD, Bond PJ, Meier T, Faraldo-Gomez JD. Structural and energetic basis for H^+^ versus Na^+^ binding selectivity in ATP synthase F_o_ rotors. *Biochim Biophys Acta* 2010; **1797**(6-7): 763-772.

80. Baker BJ, Comolli LR, Dick GJ, Hauser LJ, Hyatt D, Dill BD *et al*. Enigmatic, ultrasmall, uncultivated Archaea. *Proc Natl Acad Sci USA* 2010; **107**(19): 8806-8811.

81. Jay ZJ, Beam JP, Dlakic M, Rusch DB, Kozubal MA, Inskeep WP. Marsarchaeota are an aerobic archaeal lineage abundant in geothermal iron oxide microbial mats. *Nat microbiol* 2018; **3**(6): 732-740.

82. Comolli LR, Banfield JF. Inter-species interconnections in acid mine drainage microbial communities. *Front Microbiol* 2014; **5**: 367.

83. Golyshina OV, Toshchakov SV, Makarova KS, Gavrilov SN, Korzhenkov AA, La Cono V *et al*. ‘ARMAN’ archaea depend on association with euryarchaeal host in culture and in situ. *Nat commun* 2017; **8**(1): 60.

84. Jung MY et al. Acidification boosted nitrite incorporation into N_2_O in ammonia-oxidizing archaea and bacteria. Unpublished.

85. Saier MH, Reddy VS, Tamang DG, Västermark A. The transporter classification database. *Nucleic Acids Res* 2014; **42**: D251-D258.

86. Punta M, Coggill PC, Eberhardt RY, Mistry J, Tate J, Boursnell C *et al.* The Pfam protein families database. *Nucleic Acids Res* 2012; **40**:D290-D301.

87. Wilson D, Pethica R, Zhou YD, Talbot C, Vogel C, Madera M, *et al.* SUPERFAMILY-sophisticated comparative genomics, data mining, visualization and phylogeny. *Nucleic Acids Res* 2009; **37**:D380- D386.

88. Lebedeva EV, Hatzenpichler R, Pelletier E, Schuster N, Hauzmayer S, Bulaev A *et al.* Enrichment and genome sequence of the group I.1a ammonia-oxidizing archaeon ‘*Ca.* Nitrosotenuis uzonensis’ representing a clade globally distributed in thermal habitats. *PLoS ONE* 2013; **8**(11): e80835.

**Supplementary Figure Legends**

**Fig. S1. Flow diagram of the key experimental procedures in this study.** Two acidic soils, a forest soil with pH 5.31, and a tea orchard soil with pH 3.75 were collected. The two soils were both adjusted to acidic condition (pH 4.5) and neutral (pH 7.0) conditions by using 10 mM phosphate buffers, respectively. Nitrification activities of the two soils at different pH, as well as the growth of *Thaumarchaeota* were determined by using a ^15^N-tracing experiment and qPCR of thaumarchaeotal *amoA* genes. ^13^C-DNA-SIP microcosm experiments were further performed to capture the ^13^C-labeled genomic DNA of the active acidophilic *Thaumarchaeota* at native acidic pH in the two acidic soils. The ^13^C-labeled DNA was subsequently subjected to MDA amplification and to metagenomic sequencing. *indicated the ^13^C-labeled DNA of forest soil was also sequenced by using the Illumina HiSeq platform. Evolution of acidophilic *Thaumarchaeota* was then assessed with genome-centric analyses.

**Fig. S2** **Evaluation of MDA amplification of** **^13^C-labeled genomic DNA from DNA-SIP microcosms by agarose gel electrophoresis and community analysis based on 16S rRNA genes before and after MDA. (a)** Agarose gel electrophoresis of MDA products from the ^13^C-labeled DNA template from DNA-SIP microcosms of site 1 forest and tea orchard soils. Lane 1 and 2 are the MDA-generated DNA from the ^13^C-labeled genomic DNA of site 1 forest soil, and lanes 3 and 4 are the MDA-generated DNA from the ^13^C-labeled genomic DNA of site 1 tea orchard soil. ‘M’ represents DNA marker. **(b)** Relative abundances of 16S rRNA phylotypes in the ^13^C-labeled DNA from DNA-SIP microcosms of the forest soil and tea orchard soil before and after MDA experiment determined by using pyrosequencing analysis of the 16S rRNA genes.

**Fig. S3. ^13^C-DNA-labling of acidophilic *Thaumarchaeota* in two acidic soils.** **(a, b)** Soil pH manipulation experiment. The two acidic soils were fed with 200 μg·g^-1 15^N-urea-N dry weight soil (*d.w.s*), adjusted to pH 4.5 or pH 7.0, and incubated for a period of 7 days. The production of ^15^NO_3_^-^-N **(a)** and the number of thaumarchaeotal *amoA* genes **(b)** were determined at day-0, day-3, and day-7. **(c, d)** ^13^C-DNA-labeling experiment. The two acidic soils were fed weekly with 5% (v/v) ^13^CO_2_ and with 100 μg·g^-1 15^N-urea-N *d.w.s*, and incubated for a period of eight weeks. Quantitative distribution of thaumarchaeotal *amoA* genes was determined by qPCR after a 56-day incubation across the entire buoyant density gradient of the DNA fractions for the forest soil **(c)** and tea orchard soil **(d)**. Shown are the normalized ratios of the gene copy number in each DNA fraction to the sum of thaumarchaeotal *amoA* genes across the entire gradient of DNA fractions for each treatment. The error bars represent the standard errors of the triplicate microcosm experiments. * indicates *P* < 0.05 and ** indicates *P* < 0.01.

**Fig. S4**. **Phylogeny of ^13^C-labeled thaumarchaeotal *amoA* genes.** Representative sequences of the top three most abundant OTU (99.5% sequence identity) of thaumarchaeotal *amoA* genes are shown in the tree. The relative abundance of each OTU in the total ^13^C-labeled thaumarchaeotal *amoA* pool is indicated in brackets. The two assembled thaumarchaeotal genomes ( FS and TS) from the ^13^C-labeled DNA of the forest soil and tea orchard soils in site 1, and another metagenome-assembled *Thaumarchaeota* genome (*Ca.* Nitrososphaera sp. AFS) recovered from an acidic forest soil in site 2 are also shown.

**Fig. S5**. **Heat map showing AAI values inferred from the FS, TS and AFS genomes and published genomes of *Nitrososphaera*, *Nitrosocosmicus* and *Nitrosotalea*.**

**Fig. S6. Predicted carbon metabolism of FS and TS.** The EC numbers marked in blue indicate no putative candidate genes were found in both FS and TS based on homology searches to known enzymes involved in the respective steps. Annotations of all corresponding genes are listed in **Table S7**.

**Fig. S7. Putative cytoplasmic pH homeostasis of acidophilic/acid-tolerant *Thaumarchaeota*. (a)** Putative cytoplasmic pH homeostasis in acidophilic/acid tolerant *Thaumarchaeota*. **(i)** V-ATPase. All acidophilic/acid-tolerant *Thaumarchaeota* encode V-type ATPases. **(ii)** Increased impermeability of cell membrane. **(iii)** H^+^/solute symporter. **(iv)** Na^+^/H^+^ antiporter **(v)** Cation transporters. **(vi)** Arginine decarboxylase. There are several putative pathways for the nitrite generation during the ammonia oxidation in AOA, we only show one of them here (33). **(b)** Occurrence of the cytoplasmic pH homeostasis mechanisms in different phylotypes of *Thaumarchaeota*. Nd1, Nd2, CS and SbT1 represent the genomes of *Ca.* Nitrosotalea devanaterra Nd1, *Ca.* Nitrosotalea sinensis Nd2, *Ca.* Nitrosotalea okcheonensis CS and *Ca.* Nitrosotalea bavarica SbT1. ‘+’ means the presence of the genes, ‘-’ means the absence of the genes. Mechanisms of pH homeostasis found in acidophilic/acid-tolerant and absent in the neutrophilic *Thaumarchaeota* are highlighted in red.

**Fig. S8**. **Phylogenies of 122 conserved phylogenetic marker proteins, proteins flanking the *atp* operons, and ATPase subunits of AOA. (a)** Phylogenies of 122 concatenated conserved phylogenetic marker proteins of AOA. Phylogenies of thiosulfate/3-mercaptopyruvate sulfurtransferase proteins **(b)** and aspartate carbamoyltransferase regulatory proteins **(c)** flanking the *atp* operons of AOA. **(d-i)** Phylogenies of the subunit A, B, C, D, E and F of the ATPase of AOA.

**Fig. S9. Growth of *N. oleophilus* MY3 at different pH. (a)** The growth of MY3 incubated in artificial fresh water medium (AFM) containing 500 μM NH_4_Cl at pH 5.2, 5.5 and 7.5. The initial cell density after inoculation was ~1.0 × 10^5^ cell ml^-1^. **(b)** Growth rates of MY3 at different pH. Error bars represent the standard errors of triplicate incubations.

**Fig. S10**. **Amino acid sequence alignment of thaumarchaeotal ATPase K subunits with ATPase K subunit sequences with known atomic structures.** Residues structurally proven to be involved in the H^+^/Na^+^ selectivity and coordination are shown in colors. The conserved H^+^/Na^+^-binding site (E) is marked by a ★. Slightly polar residues (orange) and hydrophobic residues (blue) flanking this conserved site (red) are indicated. The theoretical selectivity for H^+^/Na^+^ of the subunit K, and the measured H^+^/Na^+^ selectivity due to different concentration of Na^+^ vs. H^+^ in physiological settings are shown on the right side of the figure ([76](#_ENREF_76), [78](#_ENREF_78)). ‘TM’ stands for the transmembrane helices. ● indicates the ion selectivity has been experimentally proven. Neutrophilic, acidophilic/acid-tolerant, piezophilic and halophilic ecotypes are indicated in the same colors as in Fig. 2.

**Fig. S11**. **Architecture of V-ATPases and ATP-binding catalytic sites of V- and A-ATPases. (a)** The overall architecture of V-ATPase from *Enterococcus hirae*. The three domains referred to in the main text are highlighted, A subunit in cyan, H/G subunit in red, and E subunit in blue. **(b)** Structure of A subunit in complex with an ATP analog. The ATP analog is shown as sticks and the 505 site alanine in close proximity to the analog is also shown as sticks in orange. **(c)** Magnified nucleotide-binding site as shown in (*B*). **(d)** Comparison of ATP-binding catalytic sites of A- or V-ATPases according to the crystal structure ([71](#_ENREF_71)) and site-directed mutagenesis of the A_3_B_3_ hexamer studies of V-ATPases ([73](#_ENREF_73)). Neutrophilic, acidophilic/acid-tolerant, piezophilic and halophilic ecotypes are indicated in the same colors as in Fig. 1.

**Fig. S12. Growth and activity of *N. maritimus* SCM1 under different pressure regimes. (*a*, b)** Growth and activity of *N. maritimus* SCM1 in HEPES-buffered Synthetic Crenarchaeota Media (pH 7.5) containing ~0.6-0.8 mM NH_4_Cl and bicarbonate under different pressures. ATM represents one standard atmospheric pressure, 10 MPa corresponds to the water pressure at ~1000m depth, and 20 MPa corresponds to ~2000m depth. ATM-20 MPa indicates that the SCM1 was shifted to 20 MPa after incubation at one standard ATM for 10 days (indicated by arrow). **(c)** Growth rates of SCM1 at different hydrostatic pressures.

**Fig. S13**. **Maximum likelihood species phylogenetic tree of archaea based on 122 concatenated conserved phylogenetic marker proteins.** 50 thaumarchaeotal genomes as well as 158 representative genomes of the phylum *Euryarchaeota* and the superphyla TACK, Asgard, and DPANN were included in the analysis.

**Fig. S14. Horizontal transfer of the *atp* operon among *Thaumarchaeota* and other archaeal phyla. (a)** Schematic representation of a phylogenetic tree of the four (super)phyla within the domain Archaea. Putative HGTs of the *atp* operon are represented using dashed lines between braches of the tree. ‘Thermoplasma’ represents *Thermoplasmatales*, ‘Mars’ represents *Marsarchaeota*, ‘A-Micr’ represents acidophilic *Micrarchaeota*, ‘Parv’ represents *Parvarchaeota*, ‘Halo’ represents *Halobacteriales*, and ‘Nanohalo’ represents *Nanohaloarchaeota*. ‘Npu/Nta’ represents *Nitrosopumilales* and *Nitrosotaleales*, ‘Nph’ represents *Nitrososphaerales*, and ‘Ncd’ represents *Nitrosocaldales*. Red squares represent V-ATPases among acidophilic Thermoplasma/Mars/AOA/A-Micr/Parv, and orange squares represent V-ATPases among Halo/Nanohalo. **(b)** HGT of the *atp* operon coupled with adaptive radiation of *Thaumarchaeota*. The dashed branch of acidophilic *Nitrosotenuis* was added based on the retrieval of *amoA* genes from acidophilic geothermal sites but no culture or metagenomic information is available for these ammonia-oxidizers (88). The red bars and squares represent V-ATPases acquired by HOT, the blue bars and squares indicate A-type ATPases inherited from the common neutrophilic ancestor of *Thaumarchaeota* or acquired from other neutrophilic *Thaumarchaeota* by HOT. The star symbol indicates that hadopelagic AOA contain both A- and V-ATPases. Npe, Cen, Nar represents *Nitrosopelagicus*, *Cenarchaeum* and *Nitrosarchaeum*, respectively.
