# Supplementary material for: Expansion of Thaumarchaeota habitat range is correlated with horizontal transfer of ATPase operons
Source: ISME J. 2019 Aug 28;13(12):3067–79. doi: 10.1038/s41396-019-0493-x (PMC6863869; doi:10.1038/s41396-019-0493-x)
Supplement: Supplementary file 2 — Supplementary Figures [file 41396_2019_493_MOESM2_ESM.pdf]

Fig. S1

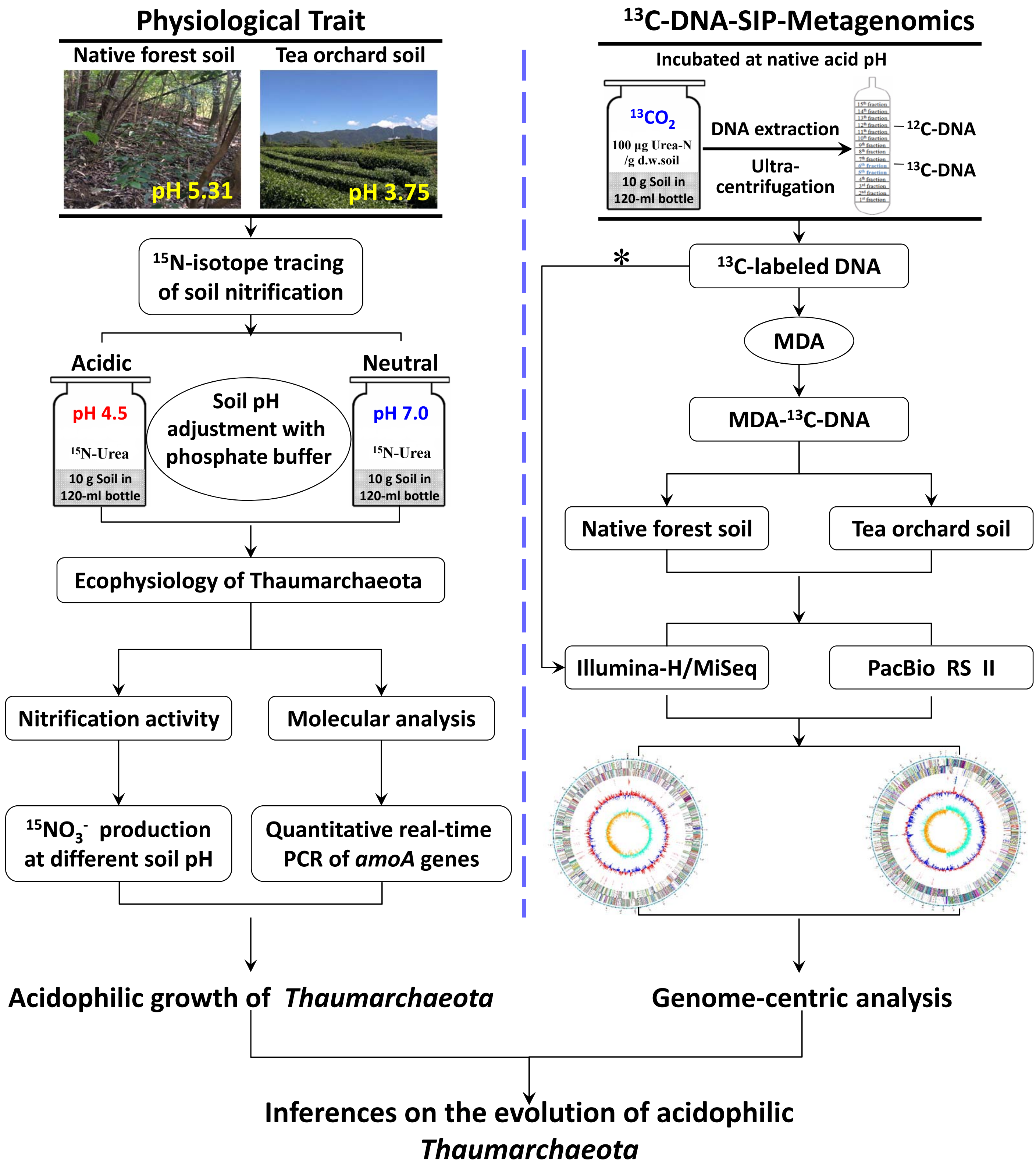
Physiological Trait
Native forest soil
Tea orchard soil
pH 5.31
pH 3.75
15N-isotope tracing of soil nitrification
Acidic
Neutral
pH 4.5
15N-Urea
10 g Soil in 120-ml bottle
Soil pH adjustment with phosphate buffer
pH 7.0
15N-Urea
10 g Soil in 120-ml bottle
Ecophysiology of Thaumarchaeota
Nitrification activity
Molecular analysis
15NO3- production at different soil pH
Quantitative real-time PCR of amoA genes
Acidophilic growth of Thaumarchaeota
13C-DNA-SIP-Metagenomics
Incubated at native acid pH
13CO2
100 μg Urea-N /g d.w.soil
10 g Soil in 120-ml bottle
DNA extraction
Ultra-centrifugation
12C-DNA
13C-DNA
*
13C-labeled DNA
MDA
MDA-13C-DNA
Native forest soil
Tea orchard soil
Illumina-H/MiSeq
PacBio RS II
Genome-centric analysis
Inferences on the evolution of acidophilic Thaumarchaeota

Fig. S2

a

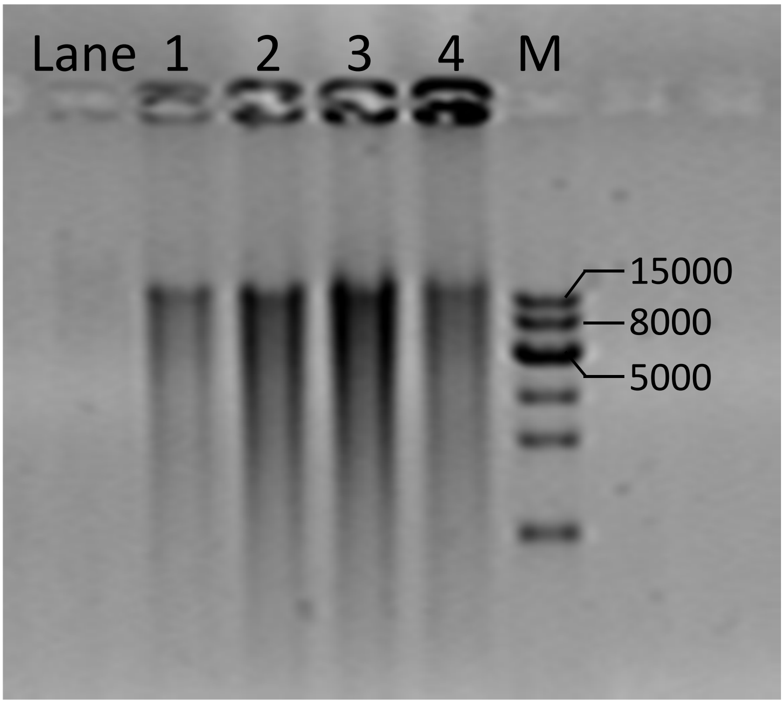
Lane 1 2 3 4 M
15000
8000
5000

b

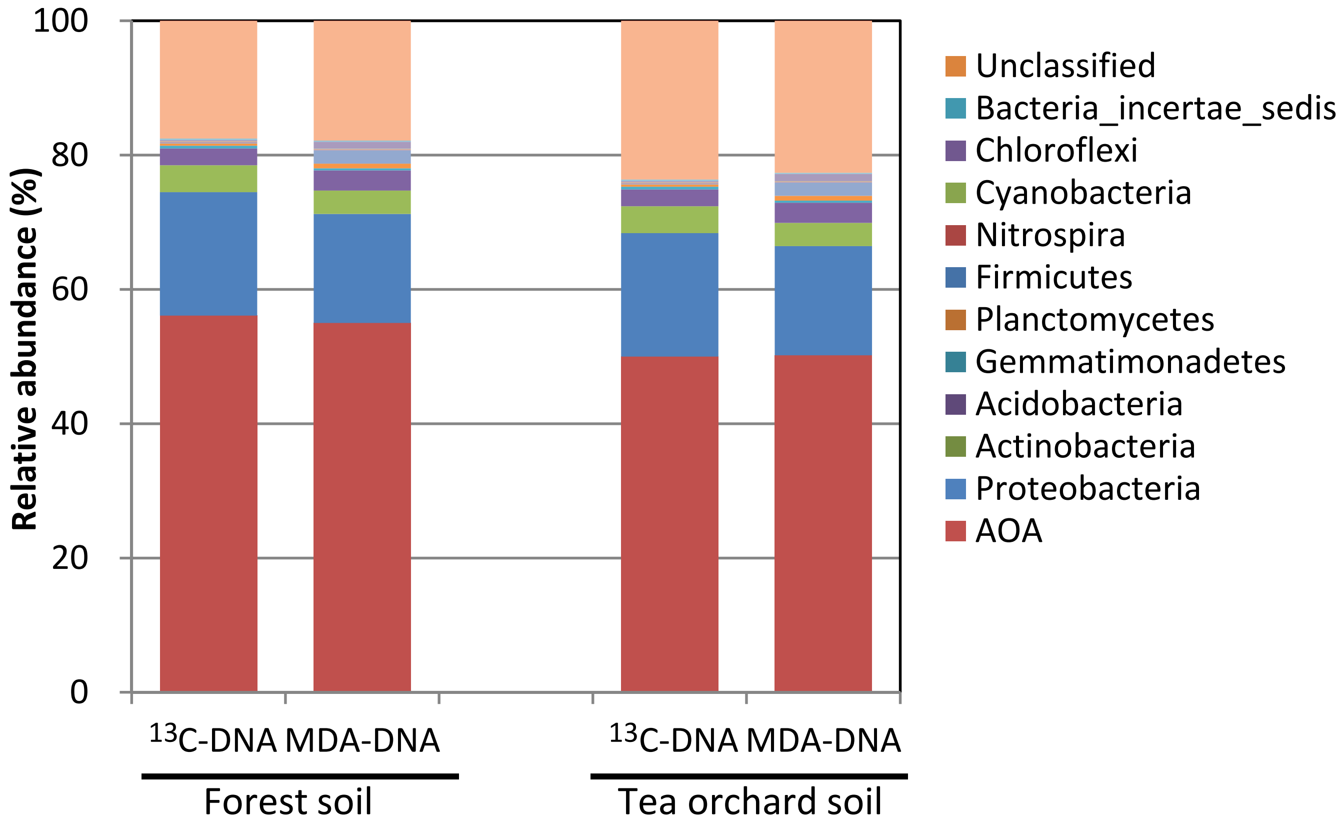
100
80
60
40
20
0
Relative abundance (%)
13C-DNA MDA-DNA
Forest soil
13C-DNA MDA-DNA
Tea orchard soil
Unclassified
Bacteria_incertae_sedis
Chloroflexi
Cyanobacteria
Nitrospira
Firmicutes
Planctomycetes
Gemmatimonadetes
Acidobacteria
Actinobacteria
Proteobacteria
AOA

Fig. S3

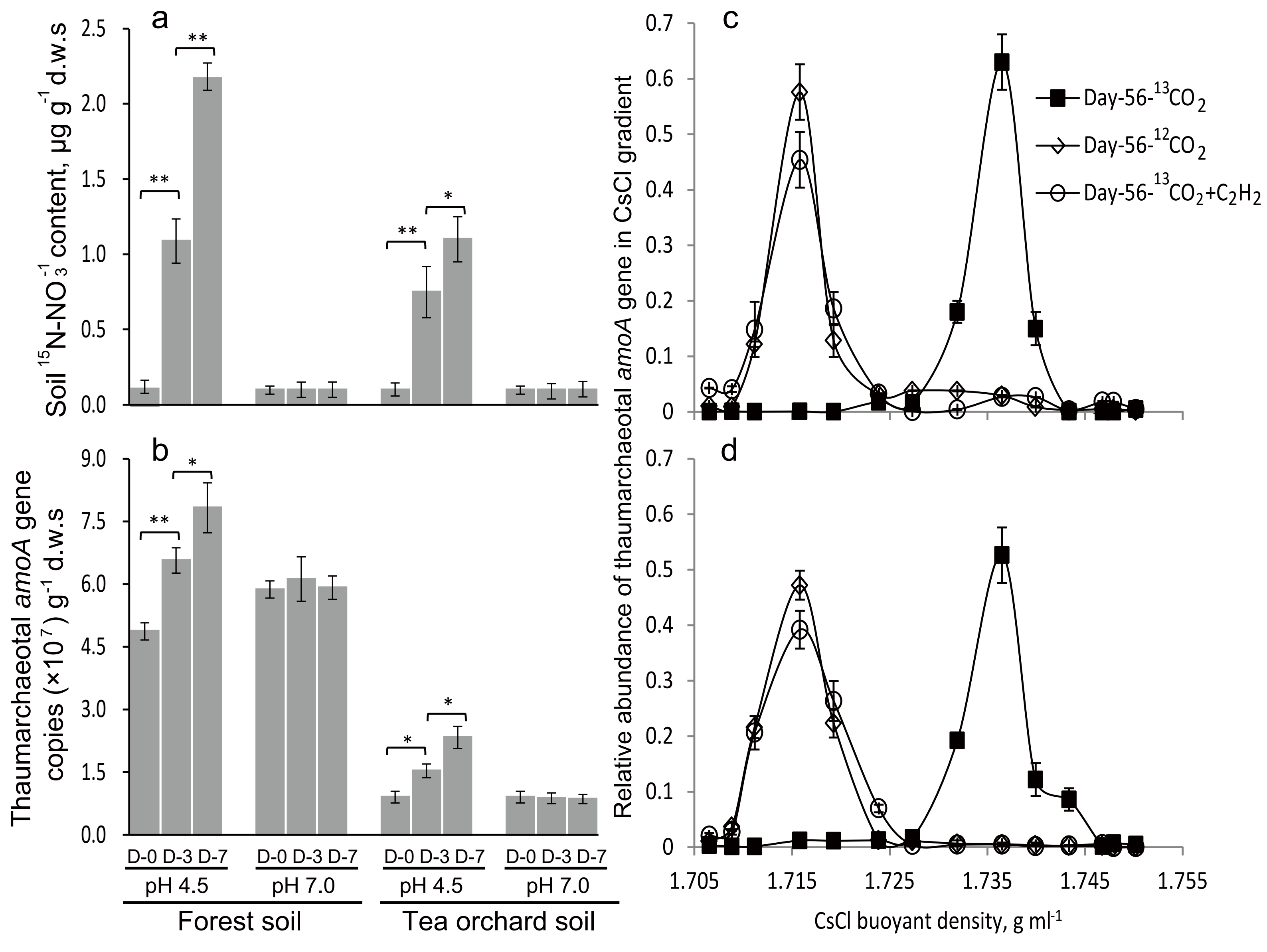

a
b
c
d
Soil ^{15}N-NO$_3^{-1}$ content, µg g^{-1} d.w.s
Thaumarchaeotal amoA gene copies (×10^7) g^{-1} d.w.s
D-0 D-3 D-7
pH 4.5
pH 7.0
Forest soil
Tea orchard soil
Relative abundance of thaumarchaeotal amoA gene in CsCl gradient
CsCl buoyant density, g ml^{-1}
Day-56-^{13}CO$_2$
Day-56-^{12}CO$_2$
Day-56-^{13}CO$_2$+C$_2$H$_2$

Fig. S4

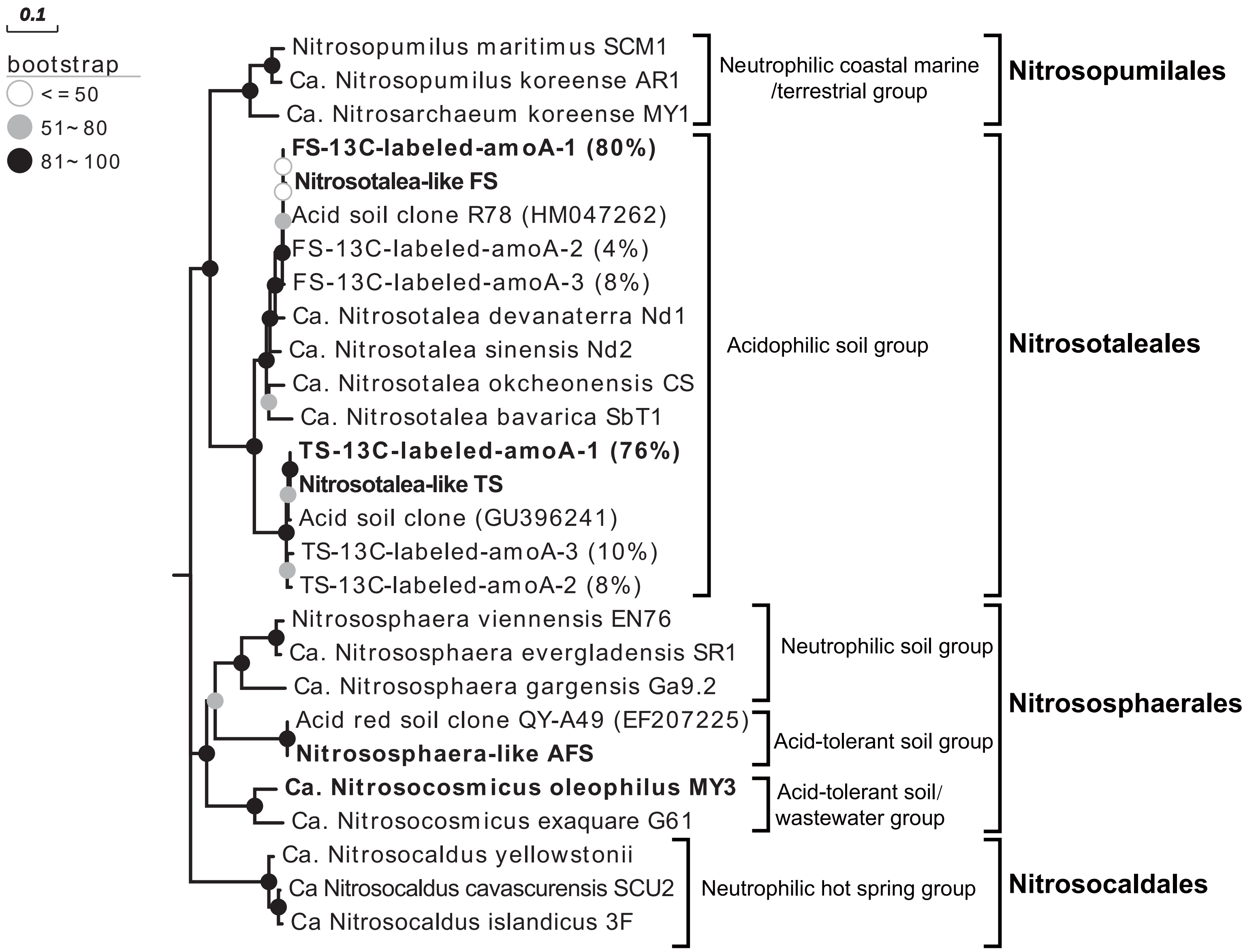
0.1
bootstrap
< = 50
51~ 80
81~ 100
Nitrosopumilus maritimus SCM1
Ca. Nitrosopumilus koreense AR1
Ca. Nitrosarchaeum koreense MY1
FS-13C-labeled-amoA-1 (80%)
Nitrosotalea-like FS
Acid soil clone R78 (HM047262)
FS-13C-labeled-amoA-2 (4%)
FS-13C-labeled-amoA-3 (8%)
Ca. Nitrosotalea devanaterra Nd1
Ca. Nitrosotalea sinensis Nd2
Ca. Nitrosotalea okcheonensis CS
Ca. Nitrosotalea bavarica SbT1
TS-13C-labeled-amoA-1 (76%)
Nitrosotalea-like TS
Acid soil clone (GU396241)
TS-13C-labeled-amoA-3 (10%)
TS-13C-labeled-amoA-2 (8%)
Nitrososphaera viennensis EN76
Ca. Nitrososphaera evergladensis SR1
Ca. Nitrososphaera gargensis Ga9.2
Acid red soil clone QY-A49 (EF207225)
Nitrososphaera-like AFS
Ca. Nitrosocosmicus oleophilus MY3
Ca. Nitrosocosmicus exaquare G61
Ca. Nitrosocaldus yellowstonii
Ca Nitrosocaldus cavascurensis SCU2
Ca Nitrosocaldus islandicus 3F
Neutrophilic coastal marine /terrestrial group
Acidophilic soil group
Neutrophilic soil group
Acid-tolerant soil group
Acid-tolerant soil/ wastewater group
Neutrophilic hot spring group
Nitrosopumilales
Nitrosotaleales
Nitrososphaerales
Nitrosocaldales

Fig. S5

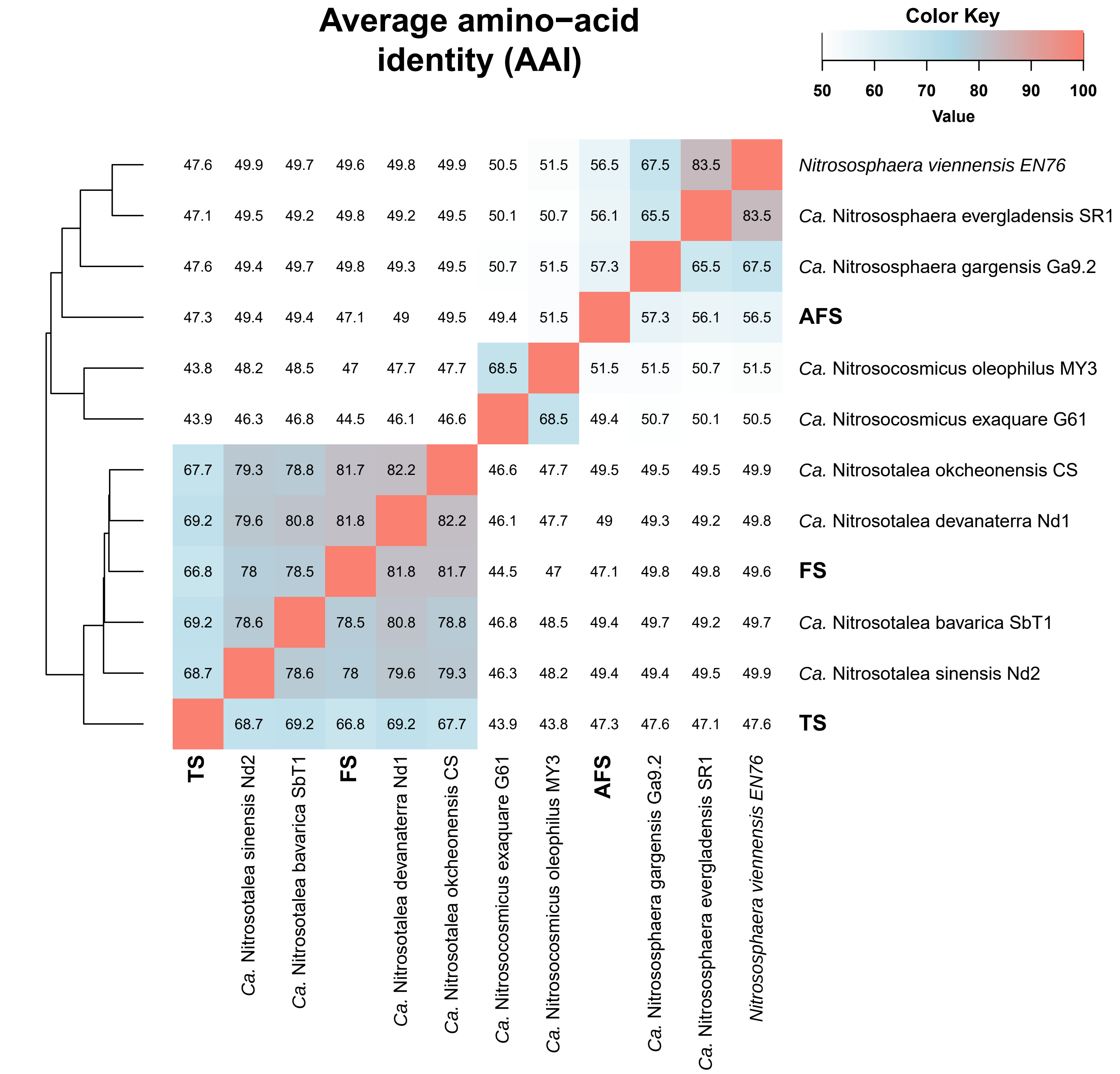
Average amino−acid identity (AAI)
Color Key
50 60 70 80 90 100
Value
47.6 49.9 49.7 49.6 49.8 49.9 50.5 51.5 56.5 67.5 83.5
47.1 49.5 49.2 49.8 49.2 49.5 50.1 50.7 56.1 65.5 83.5
47.6 49.4 49.7 49.8 49.3 49.5 50.7 51.5 57.3 65.5 67.5
47.3 49.4 49.4 47.1 49 49.5 49.4 51.5 57.3 56.1 56.5
43.8 48.2 48.5 47 47.7 47.7 68.5 51.5 51.5 50.7 51.5
43.9 46.3 46.8 44.5 46.1 46.6 68.5 49.4 50.7 50.1 50.5
67.7 79.3 78.8 81.7 82.2 46.6 47.7 49.5 49.5 49.5 49.9
69.2 79.6 80.8 81.8 82.2 46.1 47.7 49 49.3 49.2 49.8
66.8 78 78.5 81.8 81.7 44.5 47 47.1 49.8 49.8 49.6
69.2 78.6 78.5 80.8 78.8 46.8 48.5 49.4 49.7 49.2 49.7
68.7 78.6 78 79.6 79.3 46.3 48.2 49.4 49.4 49.5 49.9
68.7 69.2 66.8 69.2 67.7 43.9 43.8 47.3 47.6 47.1 47.6
Nitrososphaera viennensis EN76
Ca. Nitrososphaera evergladensis SR1
Ca. Nitrososphaera gargensis Ga9.2
AFS
Ca. Nitrosocosmicus oleophilus MY3
Ca. Nitrosocosmicus exaquare G61
Ca. Nitrosotalea okcheonensis CS
Ca. Nitrosotalea devanaterra Nd1
FS
Ca. Nitrosotalea bavarica SbT1
Ca. Nitrosotalea sinensis Nd2
TS
TS
Ca. Nitrosotalea sinensis Nd2
Ca. Nitrosotalea bavarica SbT1
FS
Ca. Nitrosotalea devanaterra Nd1
Ca. Nitrosotalea okcheonensis CS
Ca. Nitrosocosmicus exaquare G61
Ca. Nitrosocosmicus oleophilus MY3
AFS
Ca. Nitrososphaera gargensis Ga9.2
Ca. Nitrososphaera evergladensis SR1
Nitrososphaera viennensis EN76

Fig. S6

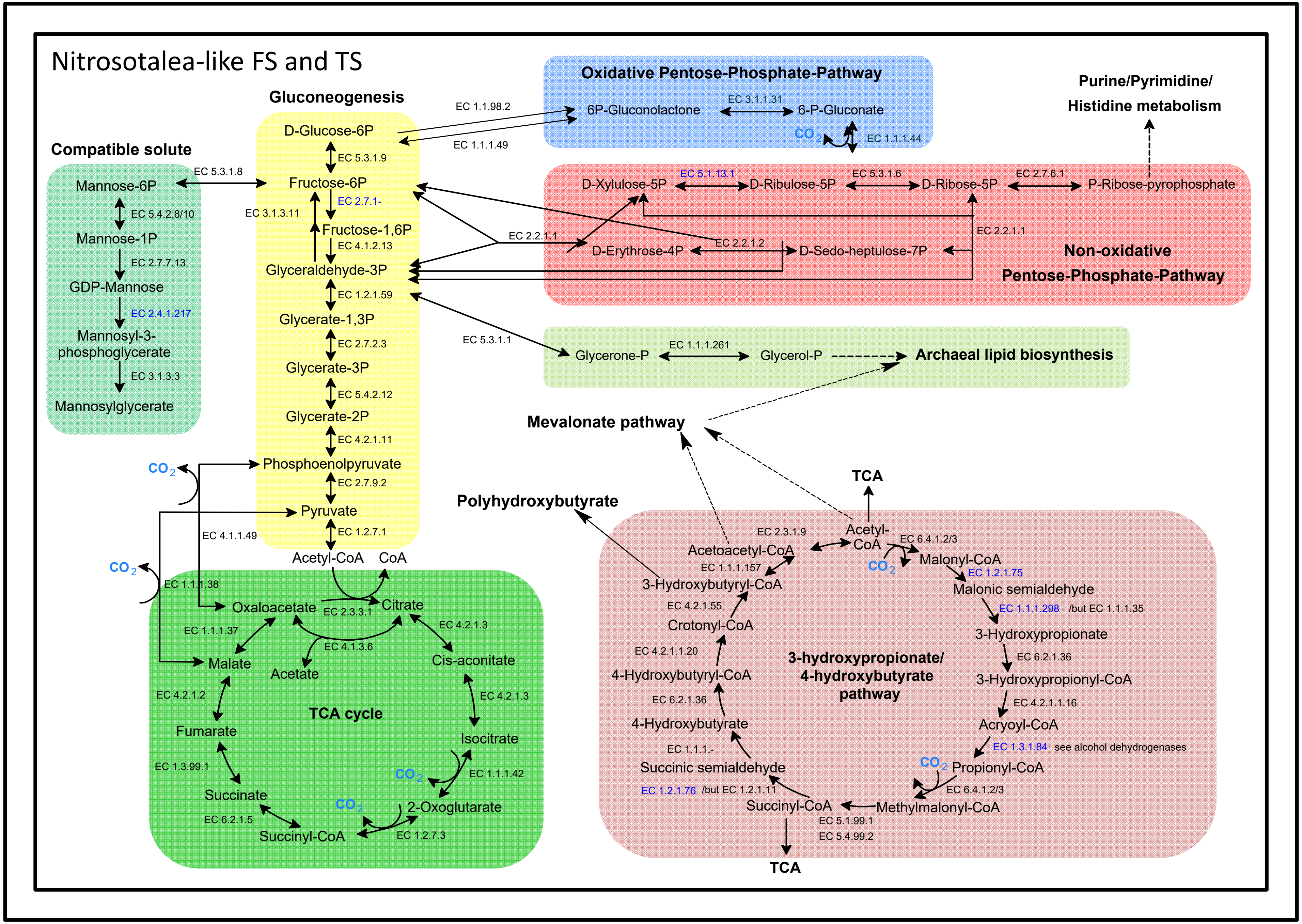

Nitrosotalea-like FS and TS
Oxidative Pentose-Phosphate-Pathway
6P-Gluconolactone
EC 3.1.1.31
6-P-Gluconate
CO_2
EC 1.1.1.44
EC 1.1.98.2
EC 1.1.1.49
Purine/Pyrimidine/
Histidine metabolism
Gluconeogenesis
D-Glucose-6P
EC 5.3.1.9
Fructose-6P
EC 2.7.1-
EC 3.1.3.11
Fructose-1,6P
EC 4.1.2.13
Glyceraldehyde-3P
EC 1.2.1.59
Glycerate-1,3P
EC 2.7.2.3
Glycerate-3P
EC 5.4.2.12
Glycerate-2P
EC 4.2.1.11
Phosphoenolpyruvate
EC 2.7.9.2
Pyruvate
EC 1.2.7.1
Acetyl-CoA
CoA
Compatible solute
Mannose-6P
EC 5.3.1.8
EC 5.4.2.8/10
Mannose-1P
EC 2.7.7.13
GDP-Mannose
EC 2.4.1.217
Mannosyl-3-
phosphoglycerate
EC 3.1.3.3
Mannosylglycerate
D-Xylulose-5P
EC 5.1.13.1
D-Ribulose-5P
EC 5.3.1.6
D-Ribose-5P
EC 2.7.6.1
P-Ribose-pyrophosphate
EC 2.2.1.1
D-Erythrose-4P
EC 2.2.1.2
D-Sedo-heptulose-7P
EC 2.2.1.1
Non-oxidative
Pentose-Phosphate-Pathway
EC 5.3.1.1
Glycerone-P
EC 1.1.1.261
Glycerol-P
Archaeal lipid biosynthesis
Mevalonate pathway
TCA
Polyhydroxybutyrate
CO_2
CO_2
EC 4.1.1.49
EC 1.1.1.38
Oxaloacetate
EC 2.3.3.1
Citrate
EC 4.2.1.3
EC 1.1.1.37
EC 4.1.3.6
Malate
Acetate
Cis-aconitate
EC 4.2.1.2
EC 4.2.1.3
TCA cycle
Fumarate
Isocitrate
CO_2
EC 1.1.1.42
EC 1.3.99.1
Succinate
CO_2
2-Oxoglutarate
EC 6.2.1.5
Succinyl-CoA
EC 1.2.7.3
EC 2.3.1.9
Acetyl-
CoA
EC 6.4.1.2/3
Acetoacetyl-CoA
CO_2
Malonyl-CoA
EC 1.1.1.157
EC 1.2.1.75
3-Hydroxybutyryl-CoA
Malonic semialdehyde
EC 4.2.1.55
EC 1.1.1.298 /but EC 1.1.1.35
Crotonyl-CoA
3-Hydroxypropionate
EC 4.2.1.1.20
EC 6.2.1.36
4-Hydroxybutyryl-CoA
3-hydroxypropionate/
4-hydroxybutyrate
pathway
3-Hydroxypropionyl-CoA
EC 6.2.1.36
EC 4.2.1.1.16
4-Hydroxybutyrate
Acryoyl-CoA
EC 1.1.1.-
EC 1.3.1.84 see alcohol dehydrogenases
Succinic semialdehyde
CO_2
Propionyl-CoA
EC 1.2.1.76 /but EC 1.2.1.11
EC 6.4.1.2/3
Succinyl-CoA
Methylmalonyl-CoA
EC 5.1.99.1
EC 5.4.99.2
TCA

Fig. S7

a

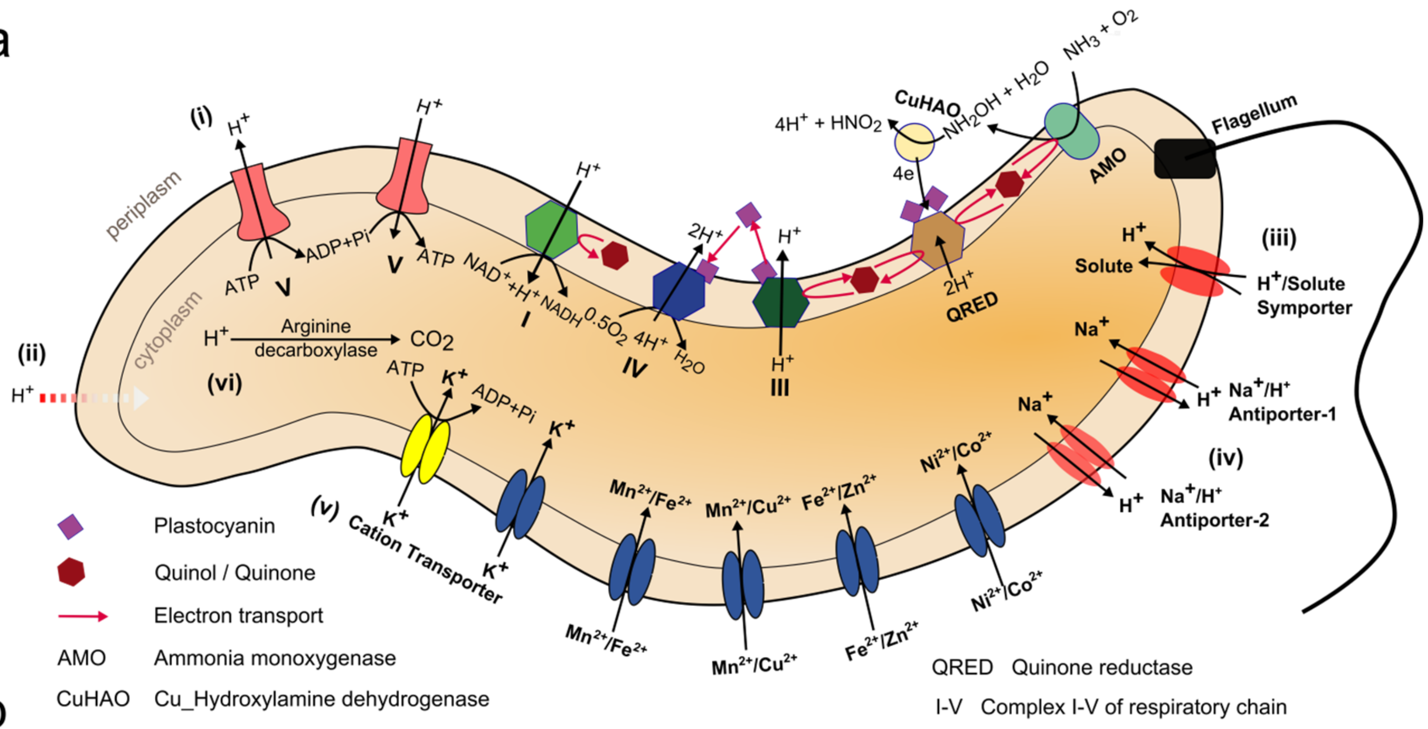

b

Genome \ Mechanism	(i) V-type ATPase	(ii) Specific membrane	(iii) H^+/Solute symporter	(iv) Na^+/H^+ antiporter-1	(iv) Na^+/H^+ antiporter-2	(v) K^+-ATPase	(v) K^+ channel	(v) Cation transporter	(vi) Arginine/Lysine decarboxylase
**Nitrosotalea-like FS**	+	+	+	+	-	+	+	+	+
**Nitrosotalea-like TS**	+	+	+	+	-	-	+	+	+
**Nd1, Nd2, CS and SbT1**[†]	+	+	+	+	-	+/-	+	+	+
**Nitrososphaera-like AFS**	+	-	-	-	+	+	+	+	+
***Ca.* N. oleophilus MY3**	+	-	-	+	+	+	+	+	+
***N. viennensis* EN76**	-	-	-	-	+	+	+	+	+
***N. maritimus* SCM1**	-	-	-	+	+	-	+	+	+

Fig. S8

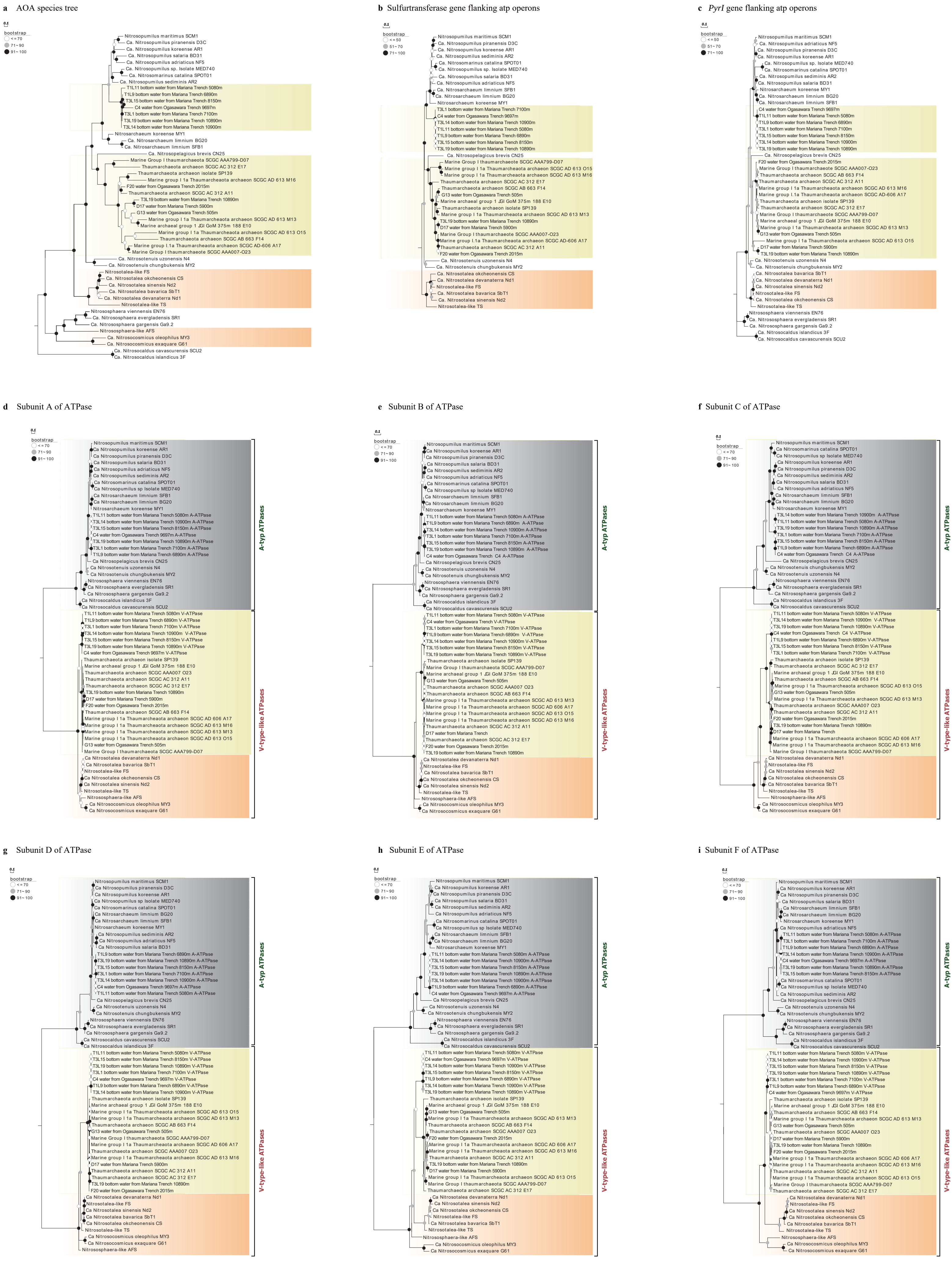
a AOA species tree
b Sulfurtransferase gene flanking atp operons
c PyrI gene flanking atp operons
d Subunit A of ATPase
e Subunit B of ATPase
f Subunit C of ATPase
g Subunit D of ATPase
h Subunit E of ATPase
i Subunit F of ATPase
0.1
bootstrap
<=70
71~90
91~100
<=50
51~70
71~100
A-typ ATPases
V-type-like ATPases

Fig. S9

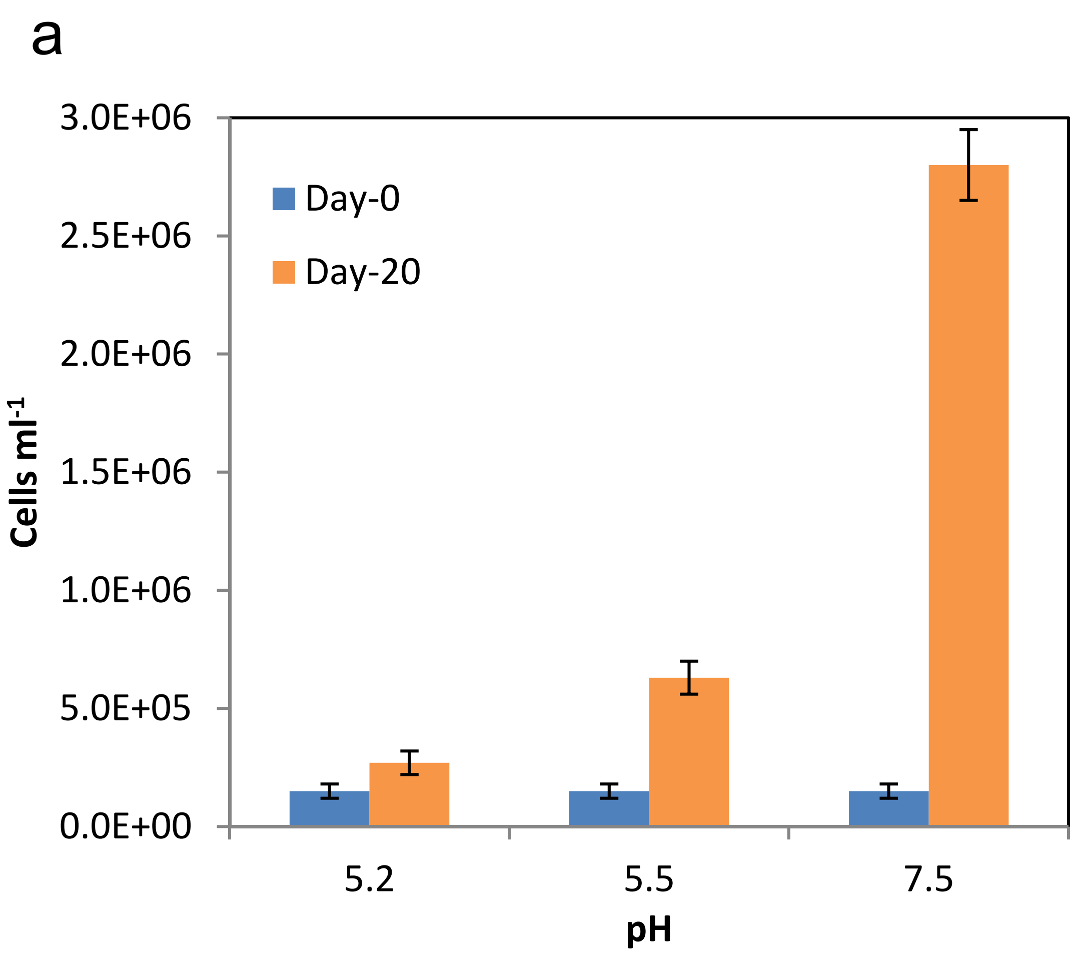
a
Cells ml-1
3.0E+06
2.5E+06
2.0E+06
1.5E+06
1.0E+06
5.0E+05
0.0E+00
Day-0
Day-20
5.2
5.5
7.5
pH

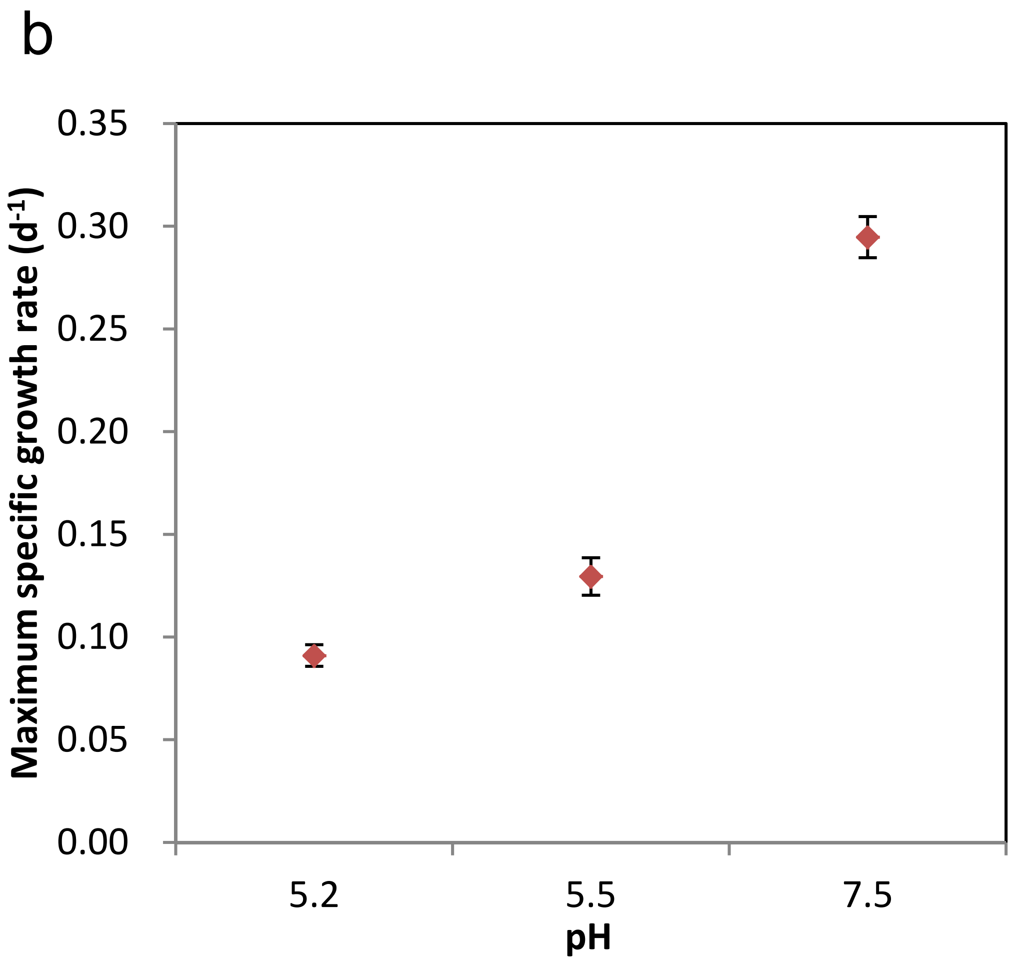
b
Maximum specific growth rate (d-1)
0.35
0.30
0.25
0.20
0.15
0.10
0.05
0.00
5.2
5.5
7.5
pH

Fig. S10

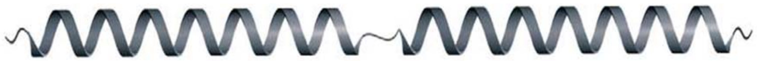

Organism	Start	Sequence	End	H^+/Na^+ selectivity	Physiological conditions
Enterococcus hirae V-ATPase	78	ILQLLPGTQGLYGFVIAFLIFINLG-------SDMSVVQGLNFLGASLPIA---FTGLFSGIAQGKVAAAGIQILAKKPEHAT----KGIIFAAMVETYAILGFVISFLLVLNA-------	156	3.5	Na^+
Ilyobacter tartaricus	1	--------------------------------MDMLFAKTVVLAASAVGAGTAMIAGIGPGVGQGYAAGKAVESVARQPEAKGDIISTMVLGQAVAESTGIYSLVIALILLYANPFVGLLG	89	~10^3	Na^+
Haloquadratum walsbyi DSM 16790	1	--------------MIGLLQQA-----------QETAPAIEPSAAAAIALG---LAALGAGYAERGIGAAAMGAIAEDDSLFV----NGLILTVLPETILILALVGFFLI-----------	78		
Halorubrum lacusprofundi AT49239	1	--------------MLEATNELGNV------ALQAGGTLTDPSAAAALAVG---LAALGAGYAERGIGSAAVGAMAEDDDLFV----NGLILTVLPETIVILALVVVFIV-----------	83		Na^++(H^+?)
Haloredivivus sp. G17	1	-----------------------------------MALELGAALGAAIAIA---VSALATAWAQAKIGTAGIGALAEDDSLFG----NVLILVAIPETIVIFGLVVALVIAGFI-------	72		
Halophilic archaeon J0756AB	1	-----------------------------------MAIEGIAGIGAAIAVG---ISAIATAYAQAKLGAAGIGALAEDDSLFG----KIFVLIAIPETMIIFGFVVALVLTGFI-------	72		
Methanosarcina acetivorans	5	--------------MVGEELISG----------PFLDADGMKALGAALAIT---VTGLASAWAEKEIGTAAIGAMAENEGLFG----KGLILTVIPETIVIFGLVVALLINSA--------	82	~10^{11}-10^{12}	H^++Na^+
Nitrososphaera viennensis EN76	12	LLTILAAVVFAGVAFASPAVYAQEAEP-----APAASSNAFKFLAAGIAFG---LAALGAGFGLGFVGAAGLAAISENPKMQS----SVFIIVGMVESIAIYGIVMMFIILGQ--------	112		
Nitrosopumilus maritimus SCM1	7	LLMAAAVISISG---STSIAYAAEGD------AAASS-DSLKILGAGLAFG---LAAFGAGIGLGQVGAAGLAVISENPALQS----KVFIFVGMVESIAIYGIVMMFIILGQ--------	102		
Ca. Nitrosopelagicus brevis CN25	16	FLLAVIAVSFTG---LTGVAHAAEED------ASSGSGDGSKLIGAGLAFG---LAAMGAGIGLGYVGSAGLAVITENPALQS----KVFIFIGMVESIAIYGIVMMFIILGQ--------	112		
Nitrosotalea-like FS	23	LLVIAAG----LTIALSTFVSTTPAFAAETSTTSSGLTKPLLAIAAAIAIA---GGLIGTGNAQQGIGAAGMGIIAEKPEKFG----QVLFFFVIPETLWIIGFVLGIILLLGIL------	115		
Nitrosotalea-like TS	23	LIIIMTG----LTIGLSTFFTTHSAFAADTTTTSSGLTKPLLAIAAAIAIA---GGLIGTGNAQQGIGAAGMGIIAEKPEKFG----QVLFFFVIPETLWIIGFVLGIILLLGIL------	115		
Ca. Nitrosotalea devanaterra Nd1	23	LLVIVAG----LTIALSTFVSTTPAFAADASTTSSGLTKPLLAIAAAIAIA---GGLIGTGNAQQGIGAAGMGIIAEKPEKFG----QVLFFFVIPETLWIIGFVLGIILLLGIL------	115		
Ca. Nitrosotalea okcheonensis CS	23	ILVIAAG----LTIALSTFVSTTPAFAAADSSTTSGLTKPLLAIAAAIAIA---GGLIGTGNAQQGIGAAGMGIIAEKPEKFG----QVLFFFVIPETLWIIGFVLGIILLLGIL------	115		
Ca. Nitrosotalea sinensis Nd2	23	LLVIAAG----LTIALSTFVSTTPAFAAESSATSSGLTKPLLAIAAAIAIA---GGLIGTGNAQQGIGAAGMGIIAEKPEKFG----QVLFFFVIPETLWIIGFVLGIILLLGIL------	115		
Ca. Nitrosotalea bavarica sbT1	24	LLVIAAG----LTIALSTFVSTTPAFAADTSTTSSGLTKPLLAIAAAIAIA---GGLIGTGNAQQGIGAAGMGIIAEKPEKFG----QVLFFFVIPETLWIIGFVLGIILLLGIL------	116		
Nitrososphaera-like AFS	1	------------------------------------MTKPLLAIAAAIAIA---GGLIGTGNAQQGIGAAGMGIIAEKPEKFG----QVLFFFVIPETLWIIGFVLGIILLLGIL------	72		
Ca. Nitrocosmicus oleophilus MY3	22	MIPVIAGAALLAFTLLTPQAFAQEN-----TTGDAGLNKSLVAIAAAIAIA---GGLIGTGNAQQGIGAAGMGIIAEKPEKFG----QVLFFFVIPETLWIIGFVLGIILLLGIL------	113		
Thaumarchaeota SCGC AAA799_D07	21	YLAIVAGAVMLVMTFVTPPAFAAEG-------GDSGLSKPILALAAAIAIA---GGLIGTGSAQQGIGAAGMGIIAEKPEKFG----QVLFFFVIPETLWIIGFVLGIILLLDIL------	110		
Thaumarchaeota SCGC AD-606_A17	21	YLAISAGAVMLLMTFVTPPAFAAEG-------GDSGLSKPILALAAAIAIA---GGLIGTGSAQQGIGAAGMGIIAEKPEKFG----QVLFFFVIPETLWIIGFVLGIILLLDIL------	110	~10^{11}-10^{15}	H^+
Thaumarchaeota SCGC AD-613_O15	21	YLAIVAGAVMLVMTFATPPAFAAEG-------GDSGLSKPILALAAAIAIA---GGLIGTGSAQQGIGAAGMGIIAEKPEKFG----QVLFFFVIPETLWIIGFVLGIILLLDIL------	110		
Thaumarchaeota SCGC AAA007_O23	21	YLAITAGAIMLVMTFVTPPAFAATG-------DDSGLSKPILALAAAIAIA---GGLIGTGSAQQGIGAAGMGIIAEKPEKFG----QVLFFFVIPETLWIIGFVLGIILLLDIL------	110		
Thaumarchaeota SCGC AB-663_F14	58	YLAIVAGAVMLSMTFISPPAFAAEG-------GDSGLSKPILALAAAIAIA---GGLIGTGSAQQGIGAAGMGIIAEKPEKFG----QVLFFFVIPETLWIIGFVLGIILLLDIL------	147		
Thaumarchaeota SCGC AC-312_E17	21	FLAVIAGAVMLVMTFATPSAFAAGG-------DDSGLSKPILALAAAIAIA---GGLIGTGSAQQGIGAAGMGIIAEKPEKFG----QVLFFFVIPETLWIIGFVLGIILLLDIL------	110		
Micrarchaeota Mia14	1	-------------------MVAS----------------ALVEIAASIAFA---GGLIGTGMAQQGIGASGMGIIAEKPEKFG----QVLFFFVIPETLWIIGFVLGLILLLQIL------	73		
Micrarchaeota FK.AMD.2014.bin.97	1	------------------MVVAD----------------PLYAIAASIAIA---GGLVGTGIVQQGIGAVGMGVIAEKPEKFG----QVLFFFVIPETLWIIGFVLGLILLLQIL------	74		
Parvarchaeota FK.AMD.2014.bin.27	1	------------------MVVAAS-------------SSGLYALAAAIAIA---GGLIGTGMAQQGIGAAGMGVIAEKPEKFG----QVLFFFVIPETLWIIGFVLGLLLLLQIL------	77		
Parvarchaeota FK.AMD.2010.bin.5	1	------------------MVVATD-------------AGALYAIAAAIAIA---GGLIGTGMAQQGIGAAGMGIIAEKPEKFG----QVLFFFVIPETLWIIGFVLGLILLLQIL------	77		
Marsarchaeota NAG2 ff85-r03	1	-----------------------------------VVTDPTLSLAAAIVMA---GGLIGTGIAQQGIGAAGMGIIAEKPEKFG----QVLFFFVIPETLWIIGFVLGVILLLNIL------	73		
Marsarchaeota NAG2 ff85-r04	1	-----------------------------------VVTDPTLSLAAAIVMA---GGLIGTGIAQQGIGAAGMGIIAEKPEKFG----QVLFFFVIPETLWIIGFVLGVILLLNIL------	73		
Thermoplasma volcanium GSS1	1	-------------------MVTT--------------VDALLAIAASISIA---GGLIGTGMAQQGIGAAGMGIIAEKPEKFG----QVLFFFVIPETLWVIGLALGIILLLHII------	75		
Picrophilus torridus DSM 9790	1	------------------MVITS--------------ADALLAVAASIAIA---GGLIGTGMAQQGIGAAGMGIIAEKPEKFG----QVLFFFVIPETLWIIGFILGIILLLHVI------	76		
Ferroplasma acidarmanus Fer1	1	-------------------MITT--------------PDAILALAASIAIA---GGLIGTGMAQQGIGAAGMGIIAEKPEKFG----QVLFFFVIPETLWIIGFVLGIILLLHVI------	75		
Spirulina platensis	1	--------------------------------MESNLTTAASVIAAALAVG---IGSIGPGLGQGQAAGQAVEGIARQPEAEGKIRGTLLLSLAFMEALTIYGLVVALVLLFANPFV----	82	~10^{14}-10^{15}	H^+

★

Fig. S11

a b c

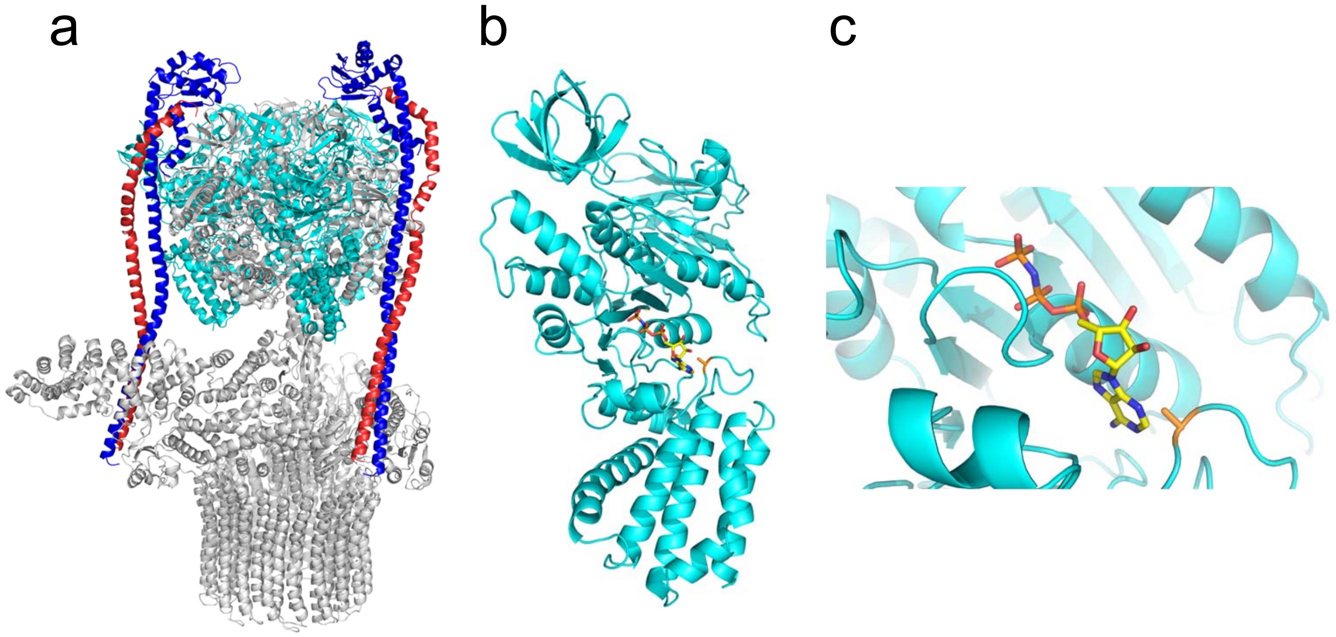

d

	Subunit A (235, 239, 261, 425, 505)	Subunit B (155, 350)
Neutrophilic soil Thaumarchaeota	-G-GKTV--ER--Y-QNSF-	--M--R--
Shallow marine Thaumarchaeota	-G-GKTV--ER--Y-QNSF-	--M--R--
Nitrosotalea-like FS	-G-GKTV--ER--F-QSAY-	--L--R--
Nitrosotalea-like TS	-G-GKTV--ER--F-QSAY-	--L--R--
Ca. Nitrosotalea devanaterra Nd1	-G-GKTV--ER--F-QSAY-	--L--R--
Ca. Nitrosotalea okcheonensis CS	-G-GKTV--ER--F-QSAY-	--L--R--
Ca. Nitrosotalea siensis Nd2	-G-GKCV--ER--F-QSAY-	--L--R--
Ca. Nitrosotalea bavarica SbT1	-G-GKTV--ER--F-QSAY-	--L--R--
Nitrosotalea-like AFS	-G-GKTV--ER--F-QSAY-	--L--R--
Ca. Nitrosocosmicus oleophilus MY3	-G-GKTV--ER--F-QSAY-	--L--R--
Thaumarchaeota BS4	-G-GKTV--ER--F-QSAF-	--L--R–
Acidophilic Micrarchaeota	-G-GKTV--ER--F-QSAF-	--L--R--
Acidophilic Parvarchaeota	-G-GKTV--ER--F-QNAF-	--L--R--
Acidophilic Marsarchaeota	-G-GKTV--ER--F-QSAF-	--L--R–
Deep marine Thaumarchaeota	-G-GKTV--ER--F-QSAF-	--L--R--
Hadalpelagic Nitrosopumilus	-G-GKTV--ER--F-QSAY-	--L--R–
Acidophilic Thermoplasmatales	-G-GKCV--ER--F-QNAF-	--L--R--
Piezophilic Pyrococcus yayanossi	-G-GKTV--ER--F-QDAF-	--L--R–
Halobacteriales	-G-GKTV--ER--F-QNAF-	--L--R--
Nanohaloarchaeota	-G-GKTV--ER--F-QNAF-	--L--R–
***Enterococcus hirae* V-type ATPase**	-G-GKTV--ER--F-QSAF-	--L--R–
***S. cerevisiae* V-type ATPase**	-G-GKTV--ER--F-QSGF-	--L--R--

Fig. S12

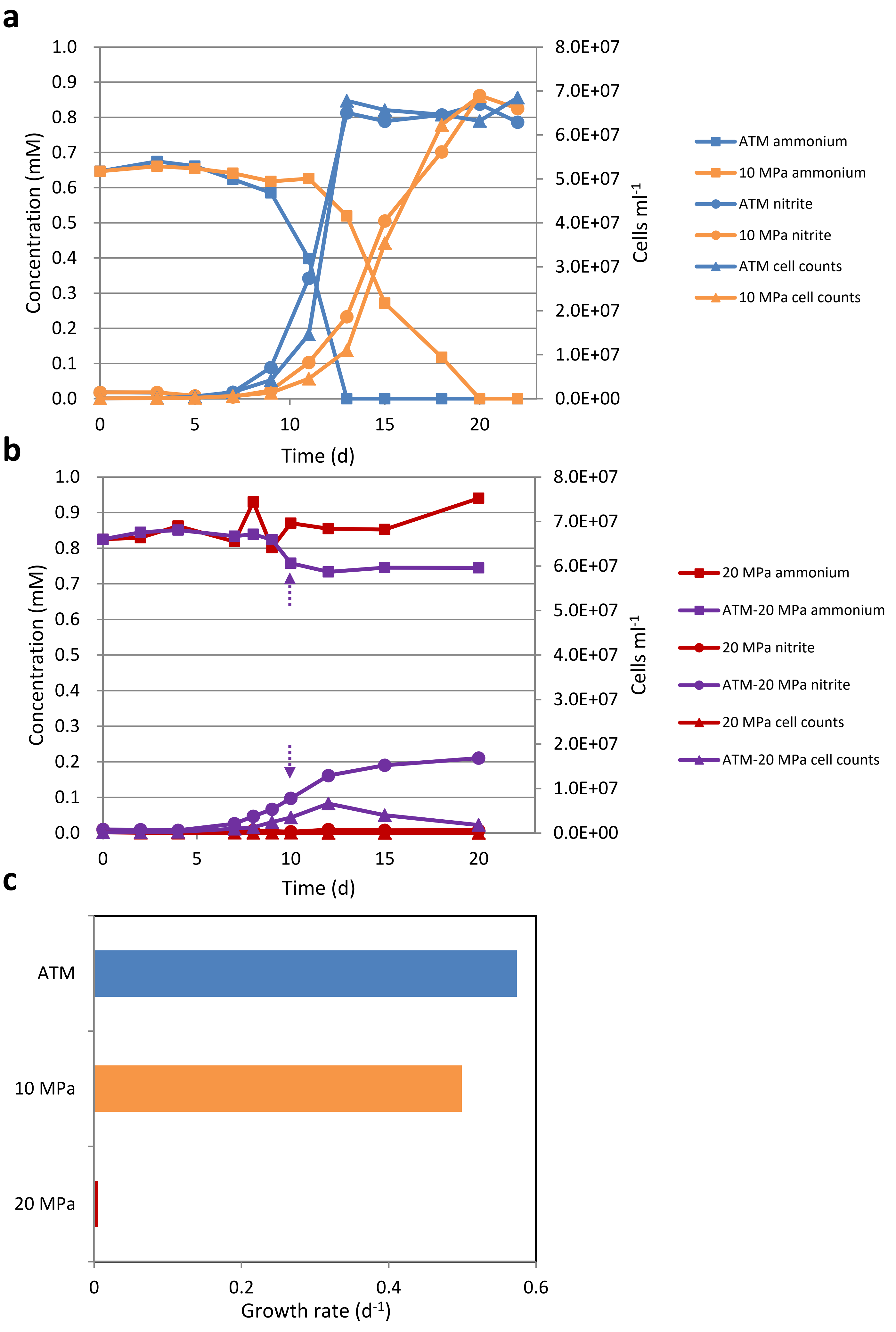

a
Concentration (mM)
Cells ml-1
Time (d)
ATM ammonium
10 MPa ammonium
ATM nitrite
10 MPa nitrite
ATM cell counts
10 MPa cell counts
b
Concentration (mM)
Cells ml-1
Time (d)
20 MPa ammonium
ATM-20 MPa ammonium
20 MPa nitrite
ATM-20 MPa nitrite
20 MPa cell counts
ATM-20 MPa cell counts
c
ATM
10 MPa
20 MPa
Growth rate (d-1)

Fig. S13

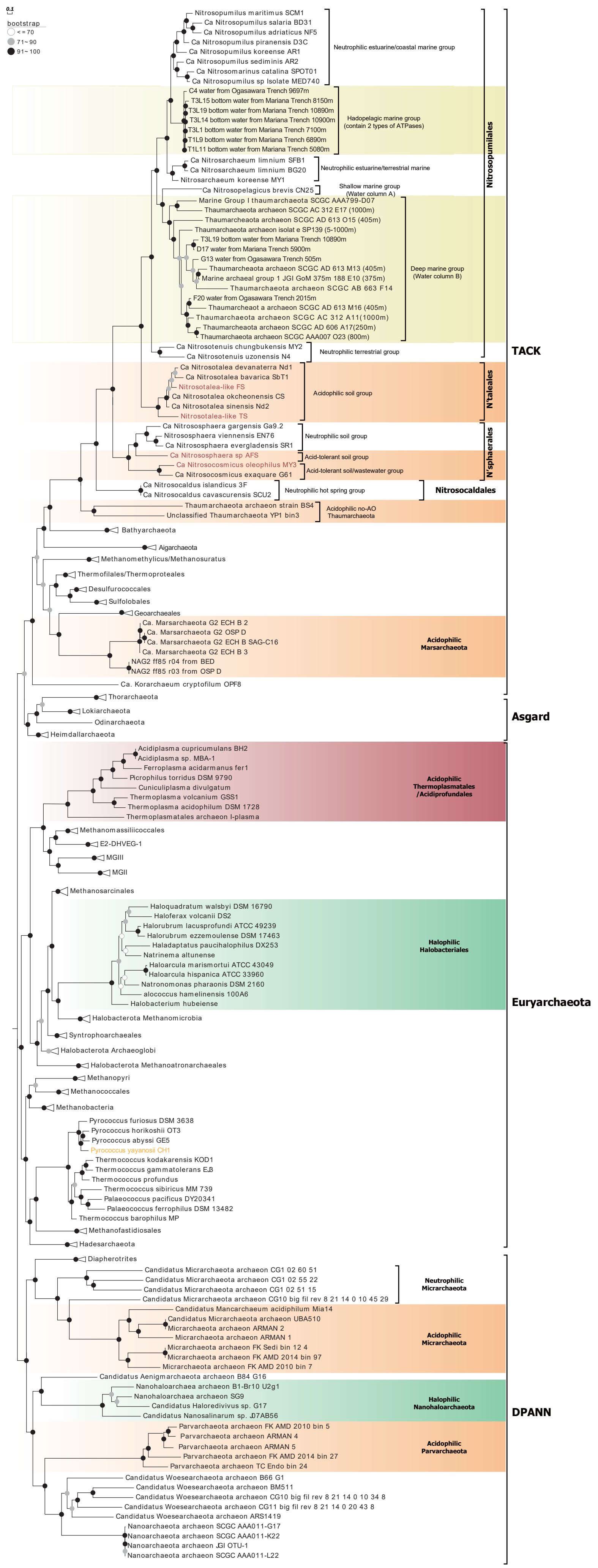
bootstrap
<= 70
71~90
91~100
Nitrosopumilus maritimus SCM1
Ca Nitrosopumilus salaria BD31
Ca Nitrosopumilus adriaticus NF5
Ca Nitrosopumilus piranensis D3C
Ca Nitrosopumilus koreense AR1
Ca Nitrosopumilus sediminis AR2
Ca Nitrosomarinus catalina SPOT01
Ca Nitrosopumilus sp Isolate MED740
Neutrophilic estuarine/coastal marine group
C4 water from Ogasawara Trench 9697m
T3L15 bottom water from Mariana Trench 8150m
T3L19 bottom water from Mariana Trench 10890m
T3L14 bottom water from Mariana Trench 10900m
T3L1 bottom water from Mariana Trench 7100m
T1L9 bottom water from Mariana Trench 6890m
T1L11 bottom water from Mariana Trench 5080m
Hadopelagic marine group (contain 2 types of ATPases)
Ca Nitrosarchaeum limnium SFB1
Ca Nitrosarchaeum limnium BG20
Nitrosarchaeum koreense MY1
Neutrophilic estuarine/terrestrial marine
Ca Nitrosopelagicus brevis CN25
Shallow marine group (Water column A)
Marine Group I thaumarchaeota SCGC AAA799-D07
Thaumarchaeota archaeon SCGC AC 312 E17 (1000m)
Thaumarchaeota archaeon SCGC AD 613 O15 (405m)
Thaumarchaeota archaeon isolat e SP139 (5-1000m)
T3L19 bottom water from Mariana Trench 10890m
D17 water from Mariana Trench 5900m
G13 water from Ogasawara Trench 505m
Thaumarcheaota archaeon SCGC AD 613 M13 (405m)
Marine archaeal group 1 JGI GoM 375m 188 E10 (375m)
Thaumarchaeota archaeon SCGC AB 663 F14
F20 water from Ogasawara Trench 2015m
Thaumarcheaot a archaeon SCGC AD 613 M16 (405m)
Thaumarchaeota archaeon SCGC AC 312 A11(1000m)
Thaumarcheaota archaeon SCGC AD 606 A17(250m)
Thaumarchaeota archaeon SCGC AAA007 O23 (800m)
Deep marine group (Water column B)
Ca Nitrosotenuis chungbukensis MY2
Ca Nitrosotenuis uzonensis N4
Neutrophilic terrestrial group
Nitrosopumilales
TACK
Ca Nitrosotalea devanaterra Nd1
Ca Nitrosotalea bavarica SbT1
Nitrosotalea-like FS
Ca Nitrosotalea okcheonensis CS
Ca Nitrosotalea sinensis Nd2
Nitrosotalea-like TS
Acidophilic soil group
N'taleales
Ca Nitrososphaera gargensis Ga9.2
Nitrososphaera viennensis EN76
Ca Nitrososphaera evergladensis SR1
Neutrophilic soil group
Ca Nitrososphaera sp AFS
Acid-tolerant soil group
Ca Nitrosocosmicus oleophilus MY3
Ca Nitrosocosmicus exaquare G61
Acid-tolerant soil/wastewater group
N'sphaerales
Ca Nitrosocaldus islandicus 3F
Ca Nitrosocaldus cavascurensis SCU2
Neutrophilic hot spring group
Nitrosocaldales
Thaumarchaeota archaeon strain BS4
Unclassified Thaumarchaeota YP1 bin3
Acidophilic no-AO Thaumarchaeota
Bathyarchaeota
Aigarchaeota
Methanomethylicus/Methanosuratus
Thermofilales/Thermoproteales
Desulfurococcales
Sulfolobales
Geoarchaeales
Ca. Marsarchaeota G2 ECH B 2
Ca. Marsarchaeota G2 OSP D
Ca. Marsarchaeota G2 ECH B SAG-C16
Ca. Marsarchaeota G2 ECH B 3
NAG2 ff85 r04 from BED
NAG2 ff85 r03 from OSP D
Acidophilic Marsarchaeota
Ca. Korarchaeum cryptofilum OPF8
Thorarchaeota
Lokiarchaeota
Odinarchaeota
Heimdallarchaeota
Asgard
Acidiplasma cupricumulans BH2
Acidiplasma sp. MBA-1
Ferroplasma acidarmanus fer1
Picrophilus torridus DSM 9790
Cuniculiplasma divulgatum
Thermoplasma volcanium GSS1
Thermoplasma acidophilum DSM 1728
Thermoplasmatales archaeon I-plasma
Acidophilic Thermoplasmatales /Acidiprofundales
Methanomassiliicoccales
E2-DHVEG-1
MGIII
MGII
Methanosarcinales
Haloquadratum walsbyi DSM 16790
Haloferax volcanii DS2
Halorubrum lacusprofundi ATCC 49239
Halorubrum ezzemoulense DSM 17463
Haladaptatus paucihalophilus DX253
Natrinema altunense
Haloarcula marismortui ATCC 43049
Haloarcula hispanica ATCC 33960
Natronomonas pharaonis DSM 2160
alococcus hamelinensis 100A6
Halobacterium hubeiense
Halophilic Halobacteriales
Halobacterota Methanomicrobia
Syntrophoarchaeales
Halobacterota Archaeoglobi
Halobacterota Methanoatronarchaeales
Methanopyri
Methanococcales
Methanobacteria
Pyrococcus furiosus DSM 3638
Pyrococcus horikoshii OT3
Pyrococcus abyssi GE5
Pyrococcus yayanosii CH1
Thermococcus kodakarensis KOD1
Thermococcus gammatolerans EJ3
Thermococcus profundus
Thermococcus sibiricus MM 739
Palaeococcus pacificus DY20341
Palaeococcus ferrophilus DSM 13482
Thermococcus barophilus MP
Methanofastidiosales
Hadesarchaeota
Euryarchaeota
Diapherotrites
Candidatus Micrarchaeota archaeon CG1 02 60 51
Candidatus Micrarchaeota archaeon CG1 02 55 22
Candidatus Micrarchaeota archaeon CG1 02 51 15
Candidatus Micrarchaeota archaeon CG10 big fil rev 8 21 14 0 10 45 29
Neutrophilic Micrarchaeota
Candidatus Mancarchaeum acidiphilum Mia14
Candidatus Micrarchaeota archaeon UBA510
Micrarchaeota archaeon ARMAN 2
Micrarchaeota archaeon ARMAN 1
Micrarchaeota archaeon FK Sedi bin 12 4
Micrarchaeota archaeon FK AMD 2014 bin 97
Micrarchaeota archaeon FK AMD 2010 bin 7
Acidophilic Micrarchaeota
Candidatus Aenigmarchaeota archaeon B84 G16
Nanohaloarchaea archaeon B1-Br10 U2g1
Nanohaloarchaea archaeon SG9
Candidatus Haloredivivus sp. G17
Candidatus Nanosalinarum sp. J07AB56
Halophilic Nanohaloarchaeota
Parvarchaeota archaeon FK AMD 2010 bin 5
Parvarchaeota archaeon ARMAN 4
Parvarchaeota archaeon ARMAN 5
Parvarchaeota archaeon FK AMD 2014 bin 27
Parvarchaeota archaeon TC Endo bin 24
Acidophilic Parvarchaeota
Candidatus Woesearchaeota archaeon B66 G1
Candidatus Woesearchaeota archaeon BM511
Candidatus Woesearchaeota archaeon CG10 big fil rev 8 21 14 0 10 34 8
Candidatus Woesearchaeota archaeon CG11 big fil rev 8 21 14 0 20 43 8
Candidatus Woesearchaeota archaeon AR51419
Nanoarchaeota archaeon SCGC AAA011-G17
Nanoarchaeota archaeon SCGC AAA011-K22
Nanoarchaeota archaeon JGI OTU-1
Nanoarchaeota archaeon SCGC AAA011-L22
DPANN

Fig. S14

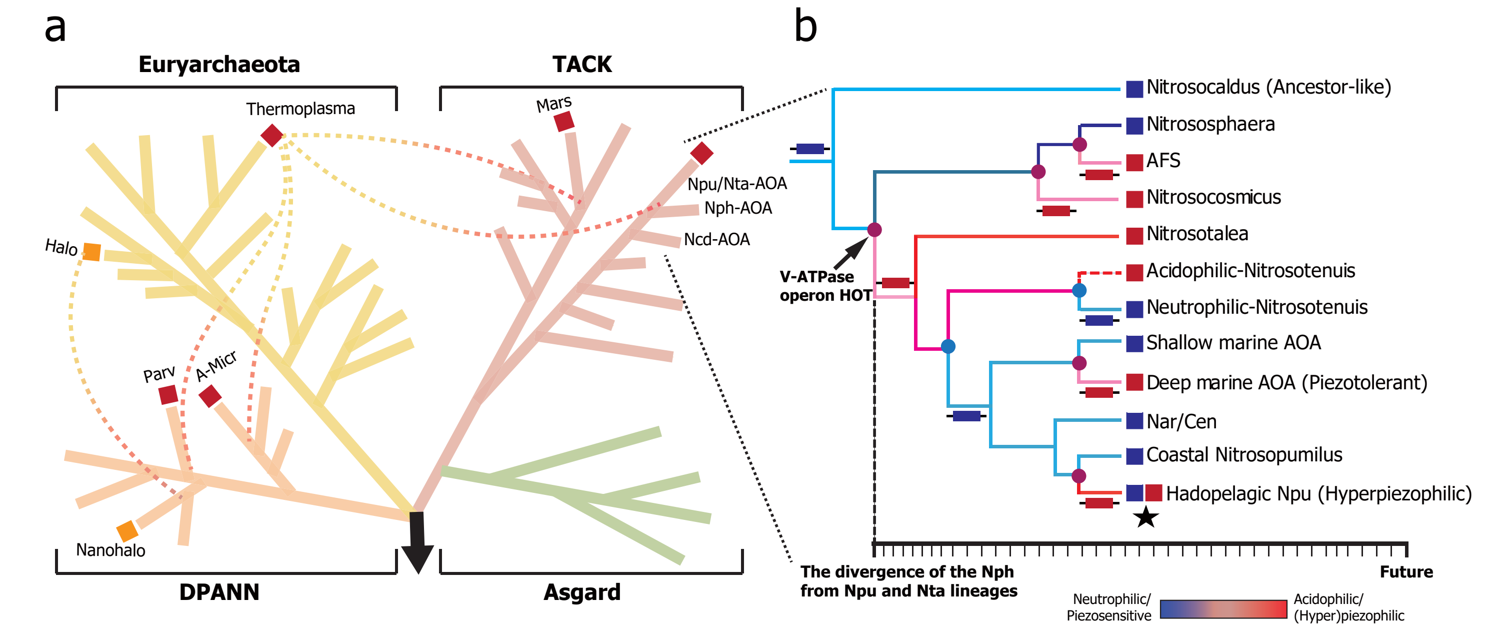
a
Euryarchaeota
TACK
Thermoplasma
Mars
Halo
Npu/Nta-AOA
Nph-AOA
Ncd-AOA
Parv
A-Micr
Nanohalo
DPANN
Asgard
b
Nitrosocaldus (Ancestor-like)
Nitrososphaera
AFS
Nitrosocosmicus
Nitrosotalea
V-ATPase
operon HOT
Acidophilic-Nitrosotenuis
Neutrophilic-Nitrosotenuis
Shallow marine AOA
Deep marine AOA (Piezotolerant)
Nar/Cen
Coastal Nitrosopumilus
Hadopelagic Npu (Hyperpiezophilic)
The divergence of the Nph
from Npu and Nta lineages
Future
Neutrophilic/
Piezosensitive
Acidophilic/
(Hyper)piezophilic
