# Supplementary material for: Expansion of Thaumarchaeota habitat range is correlated with horizontal transfer of ATPase operons
Source: ISME J. 2019 Aug 28;13(12):3067–79. doi: 10.1038/s41396-019-0493-x (PMC6863869; doi:10.1038/s41396-019-0493-x)
Supplement: Supplementary file 3 — Supplementary Tables and DNA sequences [file 41396_2019_493_MOESM3_ESM.docx]

**SI Tables and DNA sequences**

**Table S1.** Soil characteristics. P2.

**Table S2.** Primers and PCR conditions used in this study. P3-4.

**Table S3.** Accession numbers of archaeal genomes used for phylogenetic analysis of the 36/122 conserved marker genes and the energy-yielding ATPases. P5-12.

**Table S4.** General genomic features of the FS, TS, AFS and 4 selected AOA. P13.

**Table S5.** Genes encoding ammonia monooxgenase (AMO), nitrite reductase K (NirK) and urease in the FS and TS genomes. P14.

**Table S6.** Presence of *Thaumarchaeota* core genome genes in the FS and TS genomes. P15-140.

**Table S7.** Genes implicated in carbon metabolisms in the FS and TS genomes. P141-150.

**Table S8.** Presence of horizontally acquried genes in the ‘*Nitrosotalea*-specific core’ gene set identified by Herbold et al. (17) in the FS and TS genomes. P151.

**Table S9.** Specific gene sets in the FS and TS genomes but absent in the non-acidophilic thaumarchaeotal genomes. P152-165.

**Table S10.** Transporters in the FS and TS genomes. P166-178.

**Table S11.** Genes encoding proteins of complex I, II, III, IV and V in the respiration chain of the FS and TS genomes. P179-182.

**Table S12.** Homologous and non-homologous components of the A, V- and F-type ATPases. P183-184.

**Table S13.** The sequence identities of A-type atp operons and flanking genes among hadopelagic marine, neutrophilic estuarine/coastal marine, WCA and neutrophilic terrestiral group of AOA. P185.

**The synthesized DNA sequence of the V-type *atp* operon. P186-188**

**Table S1. Soil characteristics.**

| **Soil Characteristics** | **Forest soil** | **Tea orchard soil** |
| --- | --- | --- |
| Location | 30°14′N, 120°09′E | 30°14′N, 120°09′E |
| MAT (°C) | 17°C | 17°C |
| MAR (mm) | 1533 | 1533 |
| pH* | 5.31 | 3.75 |
| SOM**^†^** (g kg^-1^) | 24.8 | 50.7 |
| Total N (g kg^-1^) | 1.15 | 2.97 |
| Soil water content (%) | 22.2 | 23.6 |
| NO_3_^-^-N (mg kg^-1^) | 8.56 | 93.6 |
| NH_4_^+^-N (mg kg^-1^) | 4.85 | 7.06 |
| Available P (mg kg^-1^) | 1.61 | 128 |
| K^+^ (mg kg^-1^) | 7.26 | 27.3 |
| Na^+^ (mg kg^-1^) | 2.98 | 2.67 |
| Mg^2+^ (mg kg^-1^) | 3.19 | 2.76 |
| Ca^2+^ (mg kg^-1^) | 0.017 | 0.013 |
| Al^3+^ (mg kg^-1^) | 9.03 | 9.88 |
| As (mg kg^-1^) | 10.1 | 10.3 |
| Co (mg kg^-1^) | 7.29 | 7.27 |
| Cr (mg kg^-1^) | 78.4 | 58.9 |
| Cu (mg kg^-1^) | 20.8 | 20.5 |
| Fe^3+^ (mg kg^-1^) | 3.66 | 3.96 |
| Mn (mg kg^-1^) | 269 | 246 |
| Mo (mg kg^-1^) | 1.19 | 0.94 |
| Ni (mg kg^-1^) | 3.55 | 5.87 |
| Pb (mg kg^-1^) | 41.3 | 30.3 |
| Sb (mg kg^-1^) | 1.39 | 1.11 |
| Zn (mg kg^-1^) | 65.9 | 60.5 |

*pH was measured with water : soil ratio of 2.5.

**^†^**SOM denotes soil organic matter.

| **Table S2. Primers and PCR conditions used in this study.** | | | | | |
| --- | --- | --- | --- | --- | --- |
| **Primer Name** | **Primer sequence (5′-3′)** | **Target gene** | **Thermal Profile** | **Molecular analysis** | **Reference** |
| Arch-*amo*AF | STA ATG GTC TGG CTT AGA CG | Thaumarchaeotal *amoA* gene | 95°C, 3min; 38×(95°C, 30s; 55°C, 30s; 72°C, 45s with plate read at 83°C); Melt curve 65.0°C to 95.0°C, increment 0.5°C, 0:05+ plate read | Real-Time PCR in Fig. 1; Clone library in Fig. S3. | (3) |
| Arch-*amo*AR | GCG GCC ATC CAT CTG TAT GT |  |  |  |  |
| *amo*A-1F | GGG GTT TCT ACT GGT GGT | Bacterial *amoA* gene |  |  | (4) |
| *amo*A-2R | CCC CTC KGS AAA GCC TTC TTC |  |  |  |  |
| 515F | GTG CCA GCM GCC GCG G | Universal 16S rRNA genes | 95°C, 3min; 30×(95°C, 30s; 55°C, 30s; 72°C, 30s); 72°C, 10min | Pyrosequencing in Fig. S2. | (8) |
| 907R | CCG TCA ATT CMT TTR AGT TT |  |  |  |  |
| 519F | CAG CMG CCG CGG TAA | 16S rRNA gene of MY3 | 95°C, 3min; 38×(95°C, 30s; 55°C, 30s; 72°C, 45s with plate read at 83°C); Melt curve 65.0°C to 95.0°C, increment 0.5°C, 0:05+ plate read | Real-Time PCR in Fig. 5 | (84) |
| 727R | GCT TTC RTC CCT CAC CGT |  |  |  |  |
| amoA23F-modi | ATG GTC TGG YTW AGA CG | *amoA* gene of MY3 |  |  |  |
| amoA616R-modi | GCR GCC ATC CAT CTG TAW GT |  |  |  |  |
| 683-03315-F | CTA TGG AAG ACA ATC GTG CGA | 4-hydroxybutyrl-  CoA dehydratase gene (NMY3_03315) of MY3 | 95°C, 3min; 38×(95°C, 30s; 55°C, 30s; 72°C, 45s with plate read at 83°C); Melt curve 65.0°C to 95.0°C, increment 0.5°C, 0:05+ plate read | Real-Time PCR in Fig. 5 | (84) |
| 892-03315-R | GAT CAA TAC ATC TCC TAA TCC GG |  |  |  |  |
| 320-02370-F | CGC TTC GAC CTC GCT ACC CA | Methylmalonyl-CoA mutase large subunit gene (NMY3_02531) of MY3 |  |  |  |
| 540-02370-R | GTA CCG GAT AAC TTC TCC GG |  |  |  |  |
| MY3_atp_subA_F | TAG TTG CTG CTG GGT GAC TG | ATPase subunit A gene (NMY3_02738) of MY3 |  |  | This study |
| MY3_atp_subA_R | TGC TAA GGG TGG AAC TGC TG |  |  |  |  |

**Table S3. Accession numbers of archaeal genomes used for phylogenetic analysis of the 36/122 marker genes and the energy-yielding ATPases.**

| **Genome name** | **NCBI accession no.** | **JGI id** | **BIGD id** | **This study** |
| --- | --- | --- | --- | --- |
| *Nitrosopumilus maritimus* SCM1 | GCA_000018465.1 |  |  |  |
| *Ca.* Nitrosopumilus koreense AR1 | GCA_000299365.1 |  |  |  |
| *Ca.* Nitrosopumilus piranensis D3C | GCA_000875775.1 |  |  |  |
| *Ca.* Nitrosopumilus salaria BD31 | GCA_000242875.3 |  |  |  |
| *Ca.* Nitrosopumilus adriaticus NF5 | GCA_000956175.1 |  |  |  |
| *Ca.* Nitrosopumilus sediminis AR2 | GCA_000299395.1 |  |  |  |
| *Ca.* Nitrosomarinus catalina SPOT01 | GCA_002156965.1 |  |  |  |
| *Ca.* Nitrosopumilus sp. Isolate MED740 | GCA_002690535.1 |  |  |  |
| T1L11 bottom water from Mariana Trench 5080m |  |  | SAMC021086 |  |
| T1L9 bottom water from Mariana Trench 6890m |  |  | SAMC021091 |  |
| T3L1 bottom water from Mariana Trench 7100m |  |  | SAMC021092 |  |
| T3L15 bottom water from Mariana Trench 8150m |  |  | SAMC021093 |  |
| T3L14 bottom water from Mariana Trench 10900m |  |  | SAMC021095 |  |
| T3L19 bottom water from Mariana Trench 10890m |  |  | SAMC021096 |  |
| C4 water from Ogasawara Trench 9697m |  |  | SAMC026836 |  |
| *Ca.* Nitrosarchaeum limnium SFB1 | GCA_000204585.1 |  |  |  |
| *Ca.* Nitrosarchaeum limnium BG20 | GCA_000241145.2 |  |  |  |
| *Nitrosarchaeum koreense* MY1 | GCA_000220175.2 |  |  |  |
| *Ca.* Nitrosopelagicus brevis CN25 | GCA_000812185.1 |  |  |  |
| Thaumarchaeota archaeon isolate SP139 | GCA_002709285.1 |  |  |  |
| Marine_archaeal_group_1_JGI_GoM_375m_188_E10 |  | 2706794823 |  |  |
| Thaumarchaeota_archaeon_SCGC_AAA007_O23 |  | 2527291500 |  |  |
| Marine_group_I_1a_Thaumarcheaota_archaeon_SCGC_AD_606_A17 |  | 2740891886 |  |  |
| Thaumarchaeota_archaeon_SCGC_AC_312_A11 |  | 2657245293 |  |  |
| Marine_group_I_1a_Thaumarcheaota_archaeon_SCGC_AD_613_M16 |  | 2740891974 |  |  |
| Thaumarchaeota_archaeon_SCGC_AB_663_F14 |  | 2648501537 |  |  |
| Marine_group_I_1a_Thaumarcheaota_archaeon_SCGC_AD_613_M13 |  | 2740891970 |  |  |
| Marine_group_I_1a_Thaumarcheaota_archaeon_SCGC_AD_613_O15 |  | 2740892563 |  |  |
| Marine_Group_I_thaumarchaeote_SCGC_AAA799-D07 | GCA_000746695.1 |  |  |  |
| Thaumarchaeota_archaeon_SCGC_AC_312_E17 |  | 2657245306 |  |  |
| T3L19 bottom water from Mariana Trench 10890m |  |  | SAMC021097 |  |
| G13 water from Ogasawara Trench 505m |  |  | SAMC026833 |  |
| F20 water from Ogasawara Trench 2015m |  |  | SAMC026834 |  |
| D17 water from Mariana Trench 5900m |  |  | SAMC021089 |  |
| *Ca.* Nitrosotenuis uzonensis N4 |  |  |  |  |
| *Ca.* Nitrosotenuis chungbukensis MY2 |  |  |  |  |
| *Ca.* Nitrosotalea devanaterra Nd1 | GCA_900065925.1 |  |  |  |
| *Ca.* Nitrosotalea bavarica SbT1 | GCA_900167955.1 |  |  |  |
| *Ca.* Nitrosotalea okcheonensis CS | GCA_900177045.1 |  |  |  |
| *Ca.* Nitrosotalea sinensis Nd2 | GCA_900143675.1 |  |  |  |
| **Nitrosotalea-like FS** |  |  |  | **This study** |
| **Nitrosotalea-like TS** |  |  |  | **This study** |
| *Nitrososphaera viennensis* EN76 | GCA_000698785.1 |  |  |  |
| *Ca.* Nitrososphaera evergladensis SR1 | GCA_000730285.1 |  |  |  |
| *Ca.* Nitrososphaera gargensis Ga9.2 | GCA_000303155.1 |  |  |  |
| **Nitrososphaera-like AFS** |  |  |  | **This study** |
| *Ca.* Nitrosocosmicus oleophilus MY3 | GCA_000802205.2 |  |  |  |
| *Ca.* Nitrosocosmicus exaquare G61 | GCA_001870125.1 |  |  |  |
| *Ca.* Nitrosocaldus islandicus 3F | GCA_002906215.1 |  |  |  |
| *Ca.* Nitrosocaldus cavascurensis SCU2 | GCA_900248165.1 |  |  |  |
| Thaumarchaeota_archaeon_strain_BS4 |  | 2519899514 |  |  |
| Unclassified_Thaumarchaeota_YP1_bin3 |  | 2718217684 |  |  |
| *Ca.* Caldiarchaeum subterraneum | GCA_000270325 |  |  |  |
| Archaeon 13 2 20CM 2 53 6 | GCA_001915065.1 |  |  |  |
| Candidatus Bathyarchaeota archaeon RBG 13 38 9 | GCA_001775955 |  |  |  |
| *Ca*. Bathyarchaeota_archaeon_B76_G16 | GCA_003661965 |  |  |  |
| *Ca.* Bathyarchaeota_archaeon_B27_G16 | GCA_003662445 |  |  |  |
| *Candidatus* Korarchaeum cryptofilum OPF8 | GCF_000019605.1 |  |  |  |
| *Candidatus* Methanomethylicus mesodigestum | GCA_001717035.1 |  |  |  |
| *Candidatus* Methanomethylicus oleusabulum | GCA_001717025.1 |  |  |  |
| *Candidatus* Methanosuratus petracarbonis | GCA_001717015.1 |  |  |  |
| Crenarchaeota archaeon SCGC AAA471-B05 | GCA_000380705.1 |  |  |  |
| Thermofilum pendens Hrk 5 | GCF_000015225.1 |  |  |  |
| Thermofilum sp. ex4484 15 | GCA_002254595.1 |  |  |  |
| Caldivirga maquilingensis IC-167 | GCF_000018305.1 |  |  |  |
| Vulcanisaeta distributa DSM 14429 | GCF_000148385.1 |  |  |  |
| Pyrobaculum islandicum DSM 4184 | GCF_000015205.1 |  |  |  |
| Acidilobus saccharovorans 345-15 | GCF_000144915.1 |  |  |  |
| Desulfurococcus amylolyticus DSM 16532 | GCF_000231015.2 |  |  |  |
| Sulfolobus acidocaldarius DSM 639 | GCF_000012285.1 |  |  |  |
| Metallosphaera sedula DSM 5348 | GCF_000016605.1 |  |  |  |
| Marsarchaeota_G2_archaeon_ECH_B_2 | GCA_003019515.1 |  |  |  |
| Marsarchaeota_G2_archaeon_ECH_B_3 | GCA_003019475.1 |  |  |  |
| Marsarchaeota_G2_archaeon_OSP_D | GCA_003019545.1 |  |  |  |
| Marsarchaeota_G2_archaeon_ECH_B_SAG_C16 | GCA_003019485.1 |  |  |  |
| Marsarchaeota_G1_archaeon_OSP_D | GCA_003019565.1 |  |  |  |
| NAG2_ff85_r04_from_BED |  | 2524023239 |  |  |
| NAG2_ff85_r03_from_OSP_D |  | 2524023238 |  |  |
| *Candidatus* Heimdallarchaeota archaeon | GCA_002728275.1 |  |  |  |
| *Candidatus* Heimdallarchaeota archaeon LC 2 | GCA_001940725.1 |  |  |  |
| *Candidatus* Heimdallarchaeota archaeon LC 3 | GCA_001940645.1 |  |  |  |
| *Candidatus* Odinarchaeota archaeon LCB 4 | GCA_001940665.1 |  |  |  |
| *Candidatus* Lokiarchaeota archaeon B53 G9 | GCA 003662865.1 |  |  |  |
| Lokiarchaeaota_Lokiarchaeum_sp._GC14_75 | GCA_000986845.1 |  |  |  |
| *Candidatus* Thorarchaeota archaeon AB 25 | GCA_001940705.1 |  |  |  |
| *Candidatus* Thorarchaeota archaeon MP8T 1 | GCA_002825465.1 |  |  |  |
| *Candidatus* Thorarchaeota archaeon MP11T 1 | GCA_002825515.1 |  |  |  |
| *Candidatus* Thorarchaeota archaeon SMTZ1-83 | GCA_001563325.1 |  |  |  |
| Hadesarchaea archaeon DG-33 | GCA_001515185.1 |  |  |  |
| Hadesarchaea archaeon YNP 45 | GCA_001515205.2 |  |  |  |
| Arc I group archaeon ADurb1013 Bin02101 | GCA_001587595.1 |  |  |  |
| Arc I group archaeon BMIXfssc0709 Meth Bin006 | GCA_001587575.1 |  |  |  |
| Arc I group archaeon U1lsi0528 Bin089 | GCA_001587675.1 |  |  |  |
| Palaeococcus ferrophilus DSM 13482 | GCF_000966265.1 |  |  |  |
| Palaeococcus pacificus DY20341 | GCF_000725425.1 |  |  |  |
| Pyrococcus furiosus DSM 3638 | GCF_000007305.1 |  |  |  |
| Pyrococcus horikoshii OT3 | GCF_000011105.1 |  |  |  |
| Thermococcus kodakarensis KOD1 | GCF_000009965.1 |  |  |  |
| Thermococcus profundus | GCF_002214585.1 |  |  |  |
| Thermococcus sibiricus MM 739 | GCF_000022545.1 |  |  |  |
| Thermococcus barophilus MP | GCF_000151105.2 |  |  |  |
| Pyrococcus_abyssi_GE5 | GCA_000195935.2 |  |  |  |
| Pyrococcus_yayanosii_CH1 | GCA_000215995.1 |  |  |  |
| Thermococcus_gammatolerans_EJ3 | GCA_000022365.1 |  |  |  |
| Methanocaldococcus vulcanius M7 | GCF_000024625.1 |  |  |  |
| Methanocaldococcus villosus KIN24-T80 | GCF_000371805.1 |  |  |  |
| Methanococcus maripaludis S2 | GCF_000011585.1 |  |  |  |
| Methanobacterium formicicum | GCF_000762265.1 |  |  |  |
| Methanobrevibacter olleyae | GCF_900114585.1 |  |  |  |
| Methanosphaera cuniculi | GCF_002287195.1 |  |  |  |
| Methanosphaera stadtmanae DSM 3091 | GCF_000012545.1 |  |  |  |
| Methanothermus fervidus DSM 2088 | GCF_000166095.1 |  |  |  |
| Methanothermobacter thermautotrophicus str. Delta H | GCF_000008645.1 |  |  |  |
| Methanopyrus kandleri AV19 | GCA_000007185.1 |  |  |  |
| Methanopyrus sp. KOL6 | GCF_002201915.1 |  |  |  |
| Euryarchaeota archaeon JdFR-21 | GCA_002011165.1 |  |  |  |
| Archaeoglobus fulgidus DSM 4304 | GCF_000008665.1 |  |  |  |
| Archaeoglobus sulfaticallidus PM70-1 | GCF_000385565.1 |  |  |  |
| Ferroglobus placidus DSM 10642 | GCF_000025505.1 |  |  |  |
| Geoglobus acetivorans | GCF_000789255.1 |  |  |  |
| Candidatus Methanohalarchaeum thermophilum | GCA_001914405.1 |  |  |  |
| Methanonatronarchaeum thermophilum | GCF_002153915.1 |  |  |  |
| Haladaptatus paucihalophilus DX253 | GCF_000187225.1 |  |  |  |
| Haloarcula hispanica ATCC 33960 | GCF_000223905.1 |  |  |  |
| Halobacterium hubeiense | GCF_001488575.1 |  |  |  |
| Halococcus hamelinensis 100A6 | GCF_000336675.1 |  |  |  |
| Halorubrum ezzemoulense DSM 17463 | GCF_000421805.1 |  |  |  |
| Natrinema altunense | GCF_000731985.1 |  |  |  |
| Haloquadratum_walsbyi_DSM_16790 | GCA_000009185.1 |  |  |  |
| Haloferax_volcanii_DS2 | GCA_000025685.1 |  |  |  |
| Halorubrum_lacusprofundi_ATCC_49239 | GCA_000022205.1 |  |  |  |
| Haloarcula_marismortui_ATCC_43049 | GCA_000011085.1 |  |  |  |
| Natronomonas_pharaonis_DSM_2160 | GCA_000026045.1 |  |  |  |
| Methanoculleus marisnigri | GCA_002503885.1 |  |  |  |
| Methanoculleus bourgensis MS2 | GCF_000304355.2 |  |  |  |
| Methanocorpusculum labreanum Z | GCF_000015765.1 |  |  |  |
| Methanofollis ethanolicus | GCF_001571385.1 |  |  |  |
| Methanofollis liminatans DSM 4140 | GCF_000275865.1 |  |  |  |
| Methanolacinia paynteri | GCF_000784355.1 |  |  |  |
| Methanoregula boonei 6A8 | GCF_000017625.1 |  |  |  |
| Methanolinea tarda NOBI-1 | GCF_000235685.2 |  |  |  |
| Methanospirillum hungatei JF-1 | GCF_000013445.1 |  |  |  |
| UBA7935 | SRX993395 |  |  |  |
| UBA7939 | SRX993395 |  |  |  |
| Methanosarcinales archaeon UBA203 | GCA_002503595.1 |  |  |  |
| Methanosarcinales archaeon UBA261 | GCA_002506015.1 |  |  |  |
| Candidatus Methanoperedens nitroreducens | GCF_000685155.1 |  |  |  |
| Candidatus Methanoperedens sp. BLZ2 | GCF_002487355.1 |  |  |  |
| Methanococcoides methylutens | GCF_000765475.1 |  |  |  |
| Methanohalophilus euhalobius | GCF_900215215.1 |  |  |  |
| Methanolobus profundi | GCF_900114835.1 |  |  |  |
| Methanomethylovorans hollandica DSM 15978 | GCF_000328665.1 |  |  |  |
| Methanosarcina mazei S-6 | GCF_000970205.1 |  |  |  |
| Methanosarcina barkeri MS | GCF_000970025.1 |  |  |  |
| Methanosarcina acetivorans C2A | GCF_000007345.1 |  |  |  |
| Methanothrix thermoacetophila PT | GCA_000014945.1 |  |  |  |
| Methanothrix soehngenii GP6 | GCF_000204415.1 |  |  |  |
| ANME-1 cluster archaeon ex4572 4 | GCA_002254785.1 |  |  |  |
| Candidatus Syntrophoarchaeum caldarius | GCA_001766815.1 |  |  |  |
| Candidatus Syntrophoarchaeum butanivorans | GCA_001766825.1 |  |  |  |
| Thermoplasmatales archaeon SG8-52-3 | GCA_001595915.1 |  |  |  |
| Thermoplasmatales archaeon ex4572 165 | GCA_002254885.1 |  |  |  |
| Euryarchaeota archaeon UBA442 | GCA_002499205.1 |  |  |  |
| Euryarchaeota archaeon UBA173 | GCA_002498925.1 |  |  |  |
| Euryarchaeota archaeon UBA529 | GCA_002507305.1 |  |  |  |
| Euryarchaeota archaeon | GCA_002722735.1 |  |  |  |
| Euryarchaeota archaeon UBA136 | GCA_002506485.1 |  |  |  |
| Marine Group III euryarchaeote CG-Bathy1 | GCA_001875425.1 |  |  |  |
| Euryarchaeota archaeon UBA102 | GCA_002509225.1 |  |  |  |
| Thermoplasmatales archaeon I-plasma | GCA_001856825.1 |  |  |  |
| Acidiplasma sp. MBA-1 | GCF_000949015.1 |  |  |  |
| Acidiplasma_cupricumulans_BH2 |  | 2654587522 |  |  |
| Cuniculiplasma divulgatum | GCA_900083515.1 |  |  |  |
| Ferroplasma acidarmanus fer1 | GCF_000152265.2 |  |  |  |
| Thermoplasma acidophilum DSM 1728 | GCF_000195915.1 |  |  |  |
| Thermoplasma volcanium GSS1 | GCF_000011185.1 |  |  |  |
| Picrophilus_torridus_DSM_9790 | GCA_000008265.1 |  |  |  |
| Methanomassiliicoccus luminyensis B10 | GCF_000308215.1 |  |  |  |
| *Candidatus* Methanomethylophilus alvus Mx1201 | GCF_000300255.2 |  |  |  |
| *Candidatus* Methanoplasma termitum | GCF_000800805.1 |  |  |  |
| *Ca.* Aenigmarchaeota_archaeon_B84_G16 | GCA_003663235 |  |  |  |
| Nanohaloarchaea archaeon SG9 | GCA_001761425.1 |  |  |  |
| Nanohaloarchaea archaeon B1-Br10 U2g1 | GCA_001563875.1 |  |  |  |
| Haloredivivus_sp._G17 | GCA_000236195.2 |  |  |  |
| Candidatus Nanosalinarum sp. J07AB56 | GCA_000220355.1 |  |  |  |
| *Ca.* Woesearchaeota archaeon CG11 big fil rev 8 21 14 0 20 43 8 | GCA_002762705.1 |  |  |  |
| *Ca.* Woesearchaeota archaeon B66 G1 | GCA_003650575 |  |  |  |
| *Ca.* Woesearchaeota_archaeon_BM511 | GCA_002867475 |  |  |  |
| *Ca.* Woesearchaeota_archaeon_CG10_big_fil_rev_8_21_14_0_10_34_8 | GCA_002762855 |  |  |  |
| *Ca.* Woesearchaeota_archaeon_ARS1419 | GCA_002687795 |  |  |  |
| Parvarchaeota_archaeon_FK_AMD_2010_bin_5 |  | 2698536778 |  |  |
| Parvarchaeota_archaeon_ARMAN_4 |  | 2698536790 |  |  |
| Parvarchaeota_archaeon_ARMAN_5 |  | 2698536791 |  |  |
| Parvarchaeota_archaeon_FK_AMD_2014_bin_27 |  | 2698536782 |  |  |
| Parvarchaeota_archaeon_TC_Endo_bin_24 |  | 2698536786 |  |  |
| Candidate division DUSEL3 archaeon SCGC AAA011-E11 | GCA 000402355.1 |  |  |  |
| Ca._Diapherotrites_archaeon_B51_G17 | GCA_003661685.1 |  |  |  |
| Micrarchaeota_archaeon_CG10_big_fil_rev_8_21_14_0_10_45_29 | GCA_002778455.1 |  |  |  |
| Micrarchaeota_archaeon_CG1_02_51_15 | GCA_001871495.1 |  |  |  |
| Micrarchaeota_archaeon_CG1_02_55_22 | GCA_001871595.1 |  |  |  |
| Micrarchaeota_archaeon_CG1_02_60_51 | GCA_001871655.1 |  |  |  |
| Candidatus Micrarchaeota archaeon UBA510 | GCA_002505655.1 |  |  |  |
| Micrarchaeota_archaeon_ARMAN_2 |  | 2698536792 |  |  |
| Micrarchaeota_archaeon_Mia14 |  | 2757320680 |  |  |
| Micrarchaeota_archaeon_ARMAN_1 |  | 2698536777 |  |  |
| Micrarchaeota_archaeon_FK_AMD_2014_bin_97 |  | 2698536767 |  |  |
| Micrarchaeota_archaeon_FK_Sedi_bin_12_4 |  | 2698536770 |  |  |
| Micrarchaeota_archaeon_FK_AMD_2010_bin_7 |  | 2698536753 |  |  |

**Table S4. General genomic features of the FS and TS and 4 selected AOA.**

| **Parameter** | **Value** | | | | | | |
| --- | --- | --- | --- | --- | --- | --- | --- |
|  | ***Nitrosotalea***  **-like FS** | ***Nitrosotalea* -like TS** | ***Ca*. Nitrosotalea *devanaterra* Nd1** | ***Nitrososphaera***  **-like AFS** | ***Ca*. Nitrosocosmicus**  **oleophilu*s* MY3** | ***Nitrososphaera* *viennensis* EN76** | ***Nitrosopumilus* *maritimus* SCM1** |
| Environment | Acidic soil (5.31) | Acidic soil (3.75) | Acidic soil (4.50) | Acidic soil (4.35) | Acidic soil (5.50) | Neutral soil (8.0) | Marine aquarium |
| Cluster | *Nitrosotalea* | *Nitrosotalea* | *Nitrosotalea* | *Nitrososphaera* | *Nitrosocosmicus* | *Nitrososphaera* | *Nitrosopumilus* |
| Ph adaptation | Acidophilic | | | Acidic tolerant | | Neutrophilic | |
| Sequencing | ^13^C-DNA-SIP-metagenomics | | Pure culture | Metagenomics | Pure culture | Pure culture | Pure culture |
| Genome | This study | This study | (50) | This study | (23) | (40) | (33) |
| Size (Mb) | 1.82 | 1.54 | 1.81 | 2.34 | 3.43 | 2.53 | 1.65 |
| CheckM (%)* | 89.66 (0) | 82.85 (0) | 98.54 (0) | 93.5 (2.18) | 99.17 (0.97) | 100 (0.97) | 100 (0.97) |
| Contigs | 312 | 24 | 1 | 154 | 1 | 1 | 1 |
| N_50_ of contigs (Kb) | 22.6 | 138.1 | - | 16.6 | - | - | - |
| G+C% | 38.8% | 40.1% | 37.1% | 38.9% | 34.1% | 52.7% | 34.2% |
| ORFs | 2,768 | 2,355 | 2,205 | 3,398 | 3,725 | 2,882 | 1,997 |
| 16S-23S rRNA | 1 | 1 | 1 | 1 | 1 | 1 | 1 |
| 5S rRNA | 1 | 1 | 1 | 1 | 1 | 1 | 1 |
| tRNAs | 36 | 34 | 40 | 30 | 26 | 41 | 38 |

*CheckM predicts the genomic completeness and the level of contamination (in brackets) as a proportion of the multiple copies of conserved single-copy genes in archaea (15).

**Table S5. Genes encoding ammonia monooxgenase (AMO), nitrite reductase K (NirK) and urease in the FS and TS genomes.**

| **Gene** | **Product** | **FS** | **TS** |
| --- | --- | --- | --- |
| *amoB* | ammonia monooxygenase subunit B | FS_0047 | TS_1667 |
| *amoC* | ammonia monooxygenase subunit C | FS_0048 | TS_1666 |
| *amoX* | hypothetical protein | FS_0049 | TS_1665 |
| *amoA* | ammonia monooxygenase subunit A | FS_0050 | TS_1664 |
| *NirK-MCO* | NirK, nitrite reductase multicopper oxidase | FS_0529 | TS_1332 |
| *ureB* | urease subunit beta | FS_0619 | - |
| *ureC* | urease subunit alpha | FS_0620 | - |

**Table S6. Presence of *Thaumarchaeota* core genome genes in the FS and TS genomes.**

| OrthoGroup* | COGs in OG  (COGSOFT-EGGNOG 4.0) | Categories | Annotations | Core Genes identified in the genomes | |
| --- | --- | --- | --- | --- | --- |
|  |  |  |  | **FS** | **TS** |
| OG0001 | COG0001,COG0464, NOG00011,NOG00016 | H,O,R,T | Collagen, type,Aaa atpase,Glutamate-1-semialdehyde aminotransferase,Histidine kinase | FS_1201,FS_2092,FS_2436, FS_2727 | TS_0105,TS_1842,TS_1934 |
| OG0002 | COG0002,COG0459, NOG00028 | E,O,T | N-acetylglutamate semialdehyde dehydrogenase,Prevents misfolding and promotes the refolding and proper assembly of unfolded polypeptides generated under stress conditions, calcium calmodulin-dependent serine protein kinase | FS_0614,FS_0876,FS_2569, FS_2623 | TS_1052,TS_1860 |
| OG0004 | COG0004,COG0604, NOG00045 | C,I,P | Acyl-CoA dehydrogenase,alcohol dehydrogenase,ammonium Transporter | FS_1348,FS_2654 | TS_0294,TS_1085 |
| OG0006 | COG0006,COG1405 | E,K | Stabilizes TBP binding to an archaeal box-A promoter. Also responsible for recruiting RNA polymerase II to the pre- initiation complex (DNA-TBP-TFIIB) (By similarity),peptidase M24 | FS_0405,FS_0636,FS_1455, FS_2415,FS_2433,FS_2445 | TS_0160,TS_0823,TS_0963,  TS_1185 |
| OG0008 | COG0008,COG0075,NOG00088,NOG01218, NOG54142,NOG66928 | E,J,K,R,S,V | ABC transporter (Permease,Catalyzes the attachment of glutamate to Trna(Glu) in a two-step reaction glutamate is first activated by ATP to form Glu-AMP and then transferred to the acceptor end of Trna(Glu) (By similarity),TPR Repeat-Containing,aminotransferase, small nuclear ribonucleoprotein | FS_0331,FS_0521,FS_1245 | TS_1213 |
| OG0014 | COG1136 | V | (ABC) transporter | FS_1436,FS_2709 | TS_0306,TS_0418 |
| OG0015 | COG0015,COG3794,NOG00159 | C,F,U | Blue (Type 1) copper domain protein,Cadherins are calcium dependent cell adhesion proteins (By similarity),adenylosuccinate lyase | FS_0309,FS_1314 | TS_2100,TS_2179 |
| OG0018 | COG0018,COG0499,NOG00186 | H,J,U | Component of the adaptor protein complex 2 (AP-2). Adaptor protein complexes function in protein transport via transport vesicles in different membrane traffic pathways. Adaptor protein complexes are vesicle coat components and appear to be involved in cargo selection and vesicle formation. AP-2 is involved in clathrin-dependent endocytosis in which cargo proteins are incorporated into vesicles surrounded by clathrin (clathrin- coated vesicles, CCVs) which are destined for fusion with the early endosome. | FS_2592 | TS_0501 |
| OG0019 | COG0019,NOG00192,NOG00197,NOG03983 | E,G,M,V | Specifically catalyzes the decarboxylation of meso- diaminopimelate (meso-DAP) to L-lysine (By similarity), Transporter,acriflavin resistance protein,dolichyl-phosphate beta-D-mannosyltransferase (EC 2.4.1.83) | - | - |
| OG0020 | COG0020,NOG70190 | C,I | Ammonia monooxygenase methane monooxygenase, subunit C,Catalyzes the condensation of isopentenyl diphosphate (IPP) with allylic pyrophosphates generating different type of terpenoids (By similarity) | FS_0048 | TS_1666 |
| OG0022 | COG0696,COG1626 | G | Catalyzes the interconversion of 2-phosphoglycerate and 3-phosphoglycerate, trehalase | - | TS_1658 |
| OG0024 | COG0024,COG1146 | C,J | 4Fe-4S ferredoxin iroN-sulfur binding,Removes the N-terminal methionine from nascent proteins (By similarity) | FS_0458,FS_0975,FS_1513 | TS_0816,TS_1972,TS_2014, TS_2083 |
| OG0027 | COG0027,NOG102625,NOG65107 | F,S | Catalyzes two reactions the first one is the production of beta-formyl glycinamide ribonucleotide (GAR) from formate, ATP and beta GAR,Integral membrane protein DUF95 | - | - |
| OG0029 | NOG06733 | C | Alcohol dehydrogenase | FS_0325 | TS_2196,TS_2216 |
| OG0031 | COG0031 | E | Cysteine synthase | FS_0625,FS_1074,FS_1253 | TS_0427,TS_0495 |
| OG0032 | COG0449 | M | Catalyzes the first step in hexosamine metabolism, converting fructose-6P into glucosamine-6P using glutamine as a nitrogen source (By similarity) | FS_0343,FS_2150 | TS_2299 |
| OG0035 | NOG00358,NOG77174 | K,O | Ring finger and WD repeat domain 2, E3 ubiquitin protein ligase,asnC family transcriptional regulator. | FS_0081,FS_0429,FS_1176, FS_2313 | TS_0834,TS_1630,TS_1837 |
| OG0037 | COG0037,COG1064 | C,D | Ligates lysine onto the cytidine present at position 34 of the AUA codon-specific Trna(Ile) that contains the anticodon CAU, in an ATP-dependent manner. Cytidine is converted to lysidine, thus changing the amino acid specificity of the Trna from methionine to isoleucine (By similarity),alcohol dehydrogenase | - | - |
| OG0039 | COG0039,NOG39671,NOG59627 | C,R,S | Catalyzes the reversible oxidation of malate to oxaloacetate, pyridoxamine 5’-phosphate | - | TS_2165 |
| OG0041 | COG0041,COG0265 | F,O | Catalyzes the conversion of N5-carboxyaminoimidazole ribonucleotide (N5-CAIR) to 4-carboxy-5-aminoimidazole ribonucleotide (CAIR) (By similarity),serine protease | FS_0445,FS_1240 | TS_0827 |
| OG0042 | COG0042,COG0600 | J,P | Binding-protein-dependent transport systems inner membrane component,Catalyzes the synthesis of dihydrouridine a modified base found in the D-loop of most tRNAs (By similarity) | FS_0544 | TS_1314 |
| OG0043 | COG0043,COG0458 | F,H | Catalyzes the decarboxylation of 3-octaprenyl-4-hydroxy benzoate to 2-octaprenylphenol (By similarity),carbamoyl-phosphate synthetase ammonia chain | FS_2229,FS_2475 | TS_0557 |
| OG0044 | COG0044,COG1422,  NOG00443 | F,S,V | Dihydroorotase,Efflux transporter RND family MFP subunit,integral membrane protein | FS_2492 | TS_0866,TS_1928 |
| OG0045 | COG0045,COG0803,NOG00453 | C,G,P | Succinyl-CoA synthetase subunit beta,Synaptic vesicle glycoprotein 2A,periplasmic solute binding protein | FS_0502,FS_2498 | TS_0538 |
| OG0046 | COG0046,COG1782,NOG00461 | F,R | Beta-lactamase domain protein, phosphoribosylformylglycinamidine synthase | - | TS_1199 |
| OG0047 | COG0047,COG0361,NOG00476,NOG00479 | F,J,P,U | It seems to stimulate more or less all the activities of the other two initiation factors, IF-2 and IF-3 (By similarity),(ABC) transporter,gtp binding protein,phosphoribosylformylglycinamidine synthase | FS_0147,FS_0877,FS_2470 | TS_0570,TS_0650,TS_1566 |
| OG0050 | COG1695,COG5009 | K,M | Transcriptional regulator,penicillin-binding protein 1A | - | - |
| OG0051 | COG0051,COG0225 | J,O | Has an important function as a repair enzyme for proteins that have been inactivated by oxidation. Catalyzes the reversible oxidation-reduction of methionine sulfoxide in proteins to methionine (By similarity),Involved in the binding of Trna to the ribosomes (By similarity) | FS_1840 | TS_0194,TS_0327 |
| OG0052 | COG0052,COG1024 | I,J | 30S ribosomal protein S2,Enoyl-CoA hydratase | FS_0337,FS_0570,FS_1478 | TS_1281,TS_1512,TS_2305 |
| OG0053 | COG0053 | P | Cation diffusion facilitator family transporter | FS_0249 | TS_1872 |
| OG0054 | COG0054,COG0329,COG1412,NOG00543 | E,H,P,R | Catalyzes the formation of 6,7-dimethyl-8- ribityllumazine by condensation of 5-amino-6-(D- ribitylamino)uracil with 3,4-dihydroxy-2-butanone 4-phosphate. This is the penultimate step in the biosynthesis of riboflavin (By similarity),small subunit (SSU) processome component, homolog,Catalyzes the condensation of (S)-aspartate-beta- semialdehyde (S)-ASA and pyruvate to 4-hydroxy- tetrahydrodipicolinate (HTPA) (By similarity),tonB-dependent Receptor | FS_2650 | TS_1080 |
| OG0055 | COG0057,NOG00556 | G,S | Heme-binding protein,glyceraldehyde3phosphate dehydrogenase | FS_2169 | - |
| OG0058 | COG0058,COG0671 | G,I | Phosphorylase is an important allosteric enzyme in carbohydrate metabolism. Enzymes from different sources differ in their regulatory mechanisms and in their natural substrates. However, all known phosphorylases share catalytic and structural properties (By similarity),PHOsphatase | - | - |
| OG0059 | COG0059,COG2073,NOG00595 | E,G,H | Alpha-keto-beta-hydroxylacyl reductoisomerase,Major Facilitator superfamily,cobalamin (vitamin B12) biosynthesis CbiG protein | FS_1278 | TS_1456 |
| OG0061 | COG0061,COG0443 | G,O | Catalyzes the phosphorylation of NAD to NADP. Utilizes ATP and other nucleoside triphosphates as well as inorganic polyphosphate as a source of phosphorus (By similarity),heat shock protein 70 | FS_1142 | - |
| OG0062 | COG0062,COG1964 | G,R | Catalyzes the epimerization of the S- and R-forms of NAD(P)HX, a damaged form of NAD(P)H that is a result of enzymatic or heat-dependent hydration. This is a prerequisite for the S- specific NAD(P)H-hydrate dehydratase to allow the repair of both epimers of NAD(P)HX (By similarity),radical SAM domain protein | FS_2513 | - |
| OG0063 | COG0063,COG1797 | G,H | Responsible for the amidation of carboxylic groups at position A and C of either cobyrinic acid or hydrogenobrynic acid. NH(2) groups are provided by glutamine, and one molecule of ATP is hydrogenolyzed for each amidation (By similarity),Together with NAD(P)HX epimerase, which catalyzes the epimerization of the S- and R-forms, the enzyme allows the repair of both epimers of NAD(P)HX, a damaged form of NAD(P)H that is a result of enzymatic or heat-dependent hydration (By similarity) | FS_1156 | TS_1460 |
| OG0064 | COG0064,COG0476,NOG00643,NOG00647 | H,J,P,S | Allows the formation of correctly charged Asn-Trna(Asn) or Gln-Trna(Gln) through the transamidation of misacylated Asp- Trna(Asn) or Glu-Trna(Gln) in organisms which lack either or both of asparaginyl-Trna or glutaminyl-Trna synthetases. The reaction takes place in the presence of glutamine and ATP through an activated phospho-Asp-Trna(Asn) or phospho-Glu-Trna(Gln) (By similarity),UBA THIF-type NAD FAD binding protein,oligo-peptide transporter,receptor | FS_1138 | - |
| OG0065 | COG0065,COG1245 | E,R | ATP-binding cassette, sub-family E,Catalyzes the isomerization between 2-isopropylmalate and 3-isopropylmalate, via the formation of 2-isopropylmaleate. | FS_0012 | TS_1707 |
| OG0066 | COG0066,COG0113 | E,H | Catalyzes the isomerization between 2-isopropylmalate and 3-isopropylmalate, via the formation of 2-isopropylmaleate (By similarity),delta-aminolevulinic acid dehydratase | FS_1418 | TS_0975 |
| OG0067 | COG3889 | R | Solute binding protein-like protein | FS_0483,FS_2745 | TS_0789 |
| OG0068 | COG0068,COG1690 | O,S | Catalytic subunit of the Trna-splicing ligase complex that acts by directly joining spliced Trna halves to mature-sized tRNAs by incorporating the precursor-derived splice junction phosphate into the mature Trna as a canonical 3’,5’- phosphodiester. May act as a RNA ligase with broad substrate specificity, and may function toward other RNAs (By similarity),hydrogenase maturation protein Hypf | FS_0104 | TS_1608 |
| OG0070 | COG3945 | S | Hemerythrin hhe cation binding domain protein | FS_0659 | TS_0926,TS_1886 |
| OG0072 | COG0072,COG4820,NOG00729,NOG05922 | E,J,O,S | Prickle homolog, alpha-L-rhamnosidase,ethanolamine utilization protein eutJ,phenylalanyl-Trna synthetase (beta subunit) | FS_0078 | TS_1634 |
| OG0073 | COG0073,COG0843 | C,J | Cytochrome C oxidase, subunit I,Is required not only for elongation of protein synthesis but also for the initiation of all Mrna translation through initiator Trna(fMet) aminoacylation (By similarity) | FS_2328 | TS_2256 |
| OG0074 | COG0074,COG1503,NOG00748,NOG00749 | C,J,S,T | Histidine kinase,Peptide Chain Release Factor,Poly (ADP-ribose) polymerase,Succinyl-CoA ligase ADP-forming subunit alpha | FS_1966 | - |
| OG0075 | COG0075,COG0513 | E,L | Aminotransferase,atp-dependent rna helicase | FS_0860 | TS_2045 |
| OG0076 | COG1467 | L | DNA primase is the polymerase that synthesizes small RNA primers for the Okazaki fragments on both template strands at replication forks during chromosomal DNA synthesis (By similarity) | FS_1083,FS_2591 | TS_1479 |
| OG0080 | COG0080,COG1389 | J,L | DNA topoisomerase VI subunit B,This protein binds directly to 23S ribosomal RNA (By similarity) | FS_0150,FS_0833 | TS_1563,TS_1902 |
| OG0081 | COG0081,COG1048 | C,J | Binds directly to 23S Rrna. The L1 stalk is quite mobile in the ribosome, and is involved in E site Trna release (By similarity),aconitate hydratase | FS_0090 | TS_1623 |
| OG0082 | COG0082,COG0518,COG0519 | E,F | 5-enolpyruvylshikimate-3-phosphate phospholyase,Catalyzes the synthesis of GMP from XMP (By similarity) | FS_1109 | TS_1798 |
| OG0083 | COG0083,NOG17147 | E,R | Catalyzes the ATP-dependent phosphorylation of L- homoserine to L-homoserine phosphate (By similarity),Methyltransferase | FS_0882 | TS_0645 |
| OG0084 | COG0084,COG0399 | E,L | DegT DnrJ EryC1 StrS aminotransferase,tatd family | FS_1634 | TS_1815 |
| OG0085 | COG0085,NOG02914,NOG71390 | K,P,R | Binding-protein-dependent transport systems inner membrane component,DNA-dependent RNA polymerase catalyzes the transcription of DNA into RNA using the four ribonucleoside triphosphates as substrates (By similarity),Regulatory protein RecX | FS_0813 | - |
| OG0086 | COG0086,COG1093,NOG00861 | J,K,U | DNA-dependent RNA polymerase catalyzes the transcription of DNA into RNA using the four ribonucleoside triphosphates as substrates (By similarity),Low density lipoprotein receptor-related protein,translation initiation factor | FS_0814,FS_1152 | TS_1464 |
| OG0087 | COG0087,COG0552 | J,U | One of the primary Rrna binding proteins, it binds directly near the 3’-end of the 23S Rr where it nucleates assembly of the 50S subunit (By similarity),Involved in targeting and insertion of nascent membrane proteins into the cytoplasmic membrane. Acts as a receptor for the complex formed by the signal recognition particle (SRP) and the ribosome-nascent chain (RNC) | FS_0849 | - |
| OG0088 | COG0088,COG1478,NOG00882 | J,R,S | Catalyzes the GTP-dependent successive addition of two or more gamma-linked L-glutamates to the L-lactyl phosphodiester of 7,8-didemethyl-8-hydroxy-5-deazariboflavin (F420-0) to form coenzyme F420-0-glutamyl-glutamate (F420-2) or polyglutamated F420 derivatives (By similarity),One of the primary Rrna binding proteins, this protein initially binds near the 5’-end of the 23S Rrna. | - | TS_1945 |
| OG0089 | COG0089,COG3794,NOG00891 | C,J,S | Blue (Type 1) copper domain protein,One of the early assembly proteins it binds 23S Rrna. One of the proteins that surrounds the polypeptide exit tunnel on the outside of the ribosome. Forms the main docking site for trigger factor binding to the ribosome (By similarity) | FS_0309,FS_1055,FS_2041, FS_2088,FS_2551,FS_2672 | TS_0089,TS_0109,TS_0297, TS_0314,TS_0406,TS_0673,  TS_2100,TS_2179 |
| OG0090 | COG0090,COG1648 | H,J | One of the primary Rrna binding proteins. Required for association of the 30S and 50S subunits to form the 70S ribosome, for Trna binding and peptide bond formation. It has been suggested to have peptidyltransferase activity,Multifunctional enzyme that catalyzes the SAM-dependent methylation of uroporphyrinogen III at position C-2 and C-7 to form precorrin-2 and then position C-12 or C-18 to form trimethylpyrrocorphin 2. It also catalyzes the conversion of precorrin-2 into siroheme. This reaction consists of the NAD- dependent oxidation of precorrin-2 into sirohydrochlorin and its subsequent ferrochelation into siroheme. | FS_1416 | TS_0978 |
| OG0091 | COG0091,COG1258 | J | The globular domain of the protein is located near the polypeptide exit tunnel on the outside of the subunit, while an extended beta-hairpin is found that lines the wall of the exit tunnel in the center of the 70S ribosome, Responsible for synthesis of pseudouridine from uracil- 54 and uracil-55 in the psi GC loop of transfer RNAs (By similarity) | FS_0650 | TS_0938 |
| OG0092 | COG0092,COG1537,NOG00924,NOG00928 | C,J,R,S | Binds the lower part of the 30S subunit head. Binds Mrna in the 70S ribosome, positioning it for translation, May function in recognizing stalled ribosomes, interact with stem-loop structures in stalled Mrna molecules, and effect endonucleolytic cleavage of the Mrna. May play a role in the release non-functional ribosomes and degradation of damaged mRNAs. Has endoribonuclease activity (By similarity),Aldo keto reductase,domain protein | FS_1052,FS_2614 | TS_0676 |
| OG0093 | COG0093,COG0488,COG4671,COG4799,NOG00938,NOG01485,NOG58429 | I,J,M,R,S | (ABC) transporter,Binds to 23S Rrna. Forms part of two intersubunit bridges in the 70S ribosome (By similarity),Cadherins are calcium dependent cell adhesion proteins (By similarity),Cholesterol esterase,Glycosyl transferase (Group 1,Glycosyltransferase 28 domain protein,carboxylase | - | - |
| OG0095 | COG0095,COG1310 | H,R | Component of the eukaryotic translation initiation factor 3 (Eif-3) complex, which is involved in protein synthesis and, together with other initiation factors, stimulates binding of Mrna and methionyl-tRNAi to the 40S ribosome (By similarity),Lipoate-protein, ligase | FS_2266 | TS_0782 |
| OG0096 | COG0096,COG0156,NOG00961 | H,J,L | BTAF1 RNA polymerase II, B-TFIID transcription factor-associated, 170kDa (Mot1 homolog, S. cerevisiae),One of the primary Rrna binding proteins, it binds directly to 16S Rrna central domain where it helps coordinate assembly of the platform of the 30S subunit (By similarity),8-Amino-7-oxononanoate synthase | FS_1028 | TS_0703 |
| OG0097 | COG0040,COG0097 | E,J | This protein binds to the 23S Rr and is important in its secondary structure. It is located near the subunit interface in the base of the L7 L12 stalk, and near the Trna binding site of the peptidyltransferase center (By similarity),Catalyzes the condensation of ATP and 5-phosphoribose 1- diphosphate to form N’-(5’-phosphoribosyl)-ATP (PR-ATP). Has a crucial role in the pathway because the rate of histidine biosynthesis seems to be controlled primarily by regulation of HisG enzymatic activity. | FS_1125 | - |
| OG0098 | COG0079,COG0098 | E,J | Imidazole acetol-phosphate transaminase,Located at the back of the 30S subunit body where it stabilizes the conformation of the head with respect to the body. | FS_1129 | - |
| OG0099 | COG0099,COG0423 | J | Located at the top of the head of the 30S subunit, it contacts several helices of the 16S Rrna. In the 70S ribosome it contacts the 23S Rrna (bridge B1a) and protein L5 of the 50S subunit (bridge B1b), connecting the 2 subunits,Catalyzes the attachment of glycine to Trna(Gly). | FS_2496 | TS_1482 |
| OG0100 | COG0100,COG1635 | H,J | Involved in biosynthesis of the thiamine precursor thiazole. Catalyzes the conversion of NAD and glycine to adenosine diphosphate 5-(2-hydroxyethyl)-4-methylthiazole-2-carboxylic acid (ADT), an adenylated thiazole intermediate. The reaction includes an iron-dependent sulfide transfer from a conserved cysteine residue of the protein to a thiazole intermediate. The enzyme can only undergo a single turnover, which suggests it is a suicide enzyme. May have additional roles in adaptation to various stress conditions and in DNA damage tolerance (By similarity),Located on the platform of the 30S subunit, it bridges several disparate RNA helices of the 16S Rrna. Forms part of the Shine-Dalgarno cleft in the 70S ribosome (By similarity) | FS_0107,FS_1256 | TS_0432,TS_1604 |
| OG0101 | COG0101,COG0652,COG1287 | J,O,R | Formation of pseudouridine at positions 38, 39 and 40 in the anticodon stem and loop of transfer RNAs (By similarity),Oligosaccharyl transferase, STT3 subunit,PPIases accelerate the folding of proteins | FS_1624 | TS_1806 |
| OG0102 | COG0102,COG0495,NOG01021 | J,O | This protein is one of the early assembly proteins of the 50S ribosomal subunit, although it is not seen to bind Rrna by itself. It is important during the early stages of 50S assembly (By similarity),Leucyl-trna synthetase,heat shock | FS_0822 | TS_1890 |
| OG0103 | COG0103,COG0252 | E,J | 30S ribosomal protein S9,L-asparaginase | FS_2248 | - |
| OG0104 | COG0104,COG2511,NOG01041 | F,J,O | Allows the formation of correctly charged Gln-Trna(Gln) through the transamidation of misacylated Glu-Trna(Gln) in organisms which lack glutaminyl-Trna synthetase. The reaction takes place in the presence of glutamine and ATP through an activated gamma-phospho-Glu-Trna(Gln). The gatDE system is specific for glutamate and does not act on aspartate (By similarity),Plays an important role in the de novo pathway of purine nucleotide biosynthesis,cylindromatosis (turban tumor syndrome) | FS_1492 | - |
| OG0105 | COG0105,COG1378 | F,K | Major role in the synthesis of nucleoside triphosphates other than ATP. The ATP gamma phosphate is transferred to the NDP beta phosphate via a ping-pong mechanism, using a phosphorylated active-site intermediate (By similarity),Transcriptional regulator | FS_2514 | - |
| OG0106 | COG0106,COG1903,NOG01065 | E,H,S | Laminin alpha 1,May catalyze the methylation of C-1 in cobalt-precorrin- 5 and the subsequent extrusion of acetic acid from the resulting intermediate to form cobalt-precorrin-6A (By similarity),phosphoribosylformimino-5-aminoimidazole carboxamide ribotide isomerase | FS_1204 | TS_1455 |
| OG0107 | COG0107,COG2109,NOG01071 | E,H,S | Cob-I-yrinic acid a,c-diamide adenosyltransferase,IGPS catalyzes the conversion of PRFAR and glutamine to IGP, AICAR and glutamate. The HisF subunit catalyzes the cyclization activity that produces IGP and AICAR from PRFAR using the ammonia provided by the HisH subunit (By similarity) | FS_1160 | TS_1463 |
| OG0108 | COG0108,COG0498 | E,H | Catalyzes the conversion of D-ribulose 5-phosphate to formate and 3,4-dihydroxy-2-butanone 4-phosphate (By similarity),threonine synthase | FS_1137 | - |
| OG0109 | COG0109,COG3958 | G,O | Converts heme B (protoheme IX) to heme O by substitution of the vinyl group on carbon 2 of heme B porphyrin ring with a hydroxyethyl farnesyl side group (By similarity),Transketolase | FS_0017 | TS_1699 |
| OG0110 | COG0110,COG0294 | H,R | acetyltransferase,dihydropteroate synthase | FS_1113 | TS_1792 |
| OG0111 | COG0037,COG0111 | D,E | Ligates lysine onto the cytidine present at position 34 of the AUA codon-specific Trna(Ile) that contains the anticodon CAU, in an ATP-dependent manner. Cytidine is converted to lysidine, thus changing the amino acid specificity of the Trna from methionine to isoleucine, Dehydrogenase | FS_2253 | - |
| OG0112 | COG0112,COG0750 | E,M | Catalyzes the reversible interconversion of serine and glycine with tetrahydrofolate (THF) serving as the one-carbon carrier. This reaction serves as the major source of one-carbon groups required for the biosynthesis of purines, thymidylate, methionine, and other important biomolecules. Also exhibits THF- independent aldolase activity toward beta-hydroxyamino acids, producing glycine and aldehydes, via a retro-aldol mechanism, Membrane-associated zinc metalloprotease | FS_0008,FS_2572,FS_2616 | TS_1859 |
| OG0113 | COG0113,COG0351, COG1992 | H | Phosphomethylpyrimidine kinase,delta-aminolevulinic acid dehydratase,phosphomethylpyrimidine kinase | FS_1418 | TS_0918,TS_0919,TS_0975 |
| OG0114 | COG2520 | R | Specifically methylates the N1 position of guanosine-37 in various cytoplasmic and mitochondrial tRNAs. Methylation is not dependent on the nature of the nucleoside 5’ of the target nucleoside. This is the first step in the biosynthesis of wybutosine (Yw), a modified base adjacent to the anticodon of tRNAs and required for accurate decoding. | FS_0011 | TS_1708 |
| OG0115 | COG0115,NOG10296 | E,R | Methyltransferase, type 11,brancheD-chain amino acid aminotransferase | FS_0792,FS_0988 | TS_1763,TS_2240 |
| OG0116 | COG0500 | Q | methyltransferase | FS_0327 | TS_1246,TS_1492 |
| OG0118 | COG0118,COG1599 | E,L | IGPS catalyzes the conversion of PRFAR and glutamine to IGP, AICAR and glutamate. The hisH subunit provides the glutamine amidotransferase activity that produces the ammonia necessary to hisF for the synthesis of IGP and AICAR (By similarity),replication | FS_1404 | TS_0987 |
| OG0119 | COG0119,COG3253 | E,S | Catalyzes the condensation of the acetyl group of acetyl-CoA with 3-methyl-2-oxobutanoate (2-oxoisovalerate) to form 3-carboxy-3-hydroxy-4-methylpentanoate (2-isopropylmalate) (By similarity),chlorite dismutase | FS_1408 | TS_0985 |
| OG0120 | COG0120,COG0585,NOG01208 | G,S,T | Phosphodiesterase,Responsible for synthesis of pseudouridine from uracil- 13 in transfer RNAs (By similarity),phosphoriboisomerase A | FS_2546 | TS_1346 |
| OG0122 | COG0122,COG0667,NOG01221 | C,L,P | Aldo keto reductase,Glycosylase,Major Facilitator superfamily | FS_1411 | TS_0983 |
| OG0123 | COG0123,COG1736 | BQ,J | Histone deacetylase,diphthamide biosynthesis protein | FS_1120 | TS_1786 |
| OG0124 | COG0124,COG0155 | C,J | Component of the sulfite reductase complex that catalyzes the 6-electron reduction of sulfite to sulfide. This is one of several activities required for the biosynthesis of L- cysteine from sulfate (By similarity),Histidyl-trna synthetase | FS_1264 | TS_0435 |
| OG0126 | COG0126,COG0367 | E,G | Asparagine synthetase,phosphoglycerate kinase | FS_0100 | TS_1615 |
| OG0128 | COG0128,COG1942 | E,R | 4-Oxalocrotonate tautomerase,5-enolpyruvylshikimate-3-phosphate synthase | FS_2667 | TS_1105 |
| OG0129 | COG0129,COG4799 | E,I | Dihydroxy-acid dehydratase,carboxylase | FS_1747 | TS_1736 |
| OG0131 | COG0131,COG1980 | E,G | EC 3.1.3.11,imidazoleglycerolphosphate dehydratase | FS_1131,FS_2181 | - |
| OG0133 | COG0133,COG4257,NOG118183,NOG77731 | E,S,V | Inactivates the type B streptogramin antibiotics by linearizing the lactone ring at the ester linkage, generating a free phenylglycine carboxylate and converting the threonyl moiety into 2-amino-butenoic acid,The beta subunit is responsible for the synthesis of L- tryptophan from indole and L-serine (By similarity) | - | - |
| OG0135 | COG1899,NOG01357 | G,O | Catalyzes the NAD-dependent oxidative cleavage of spermidine and the subsequent transfer of the butylamine moiety of spermidine to the epsilon-amino group of a specific lysine residue of the Eif-5A precursor protein to form the intermediate deoxyhypusine residue,Periplasmic binding protein LacI transcriptional regulator | FS_0701,FS_1173 | TS_0068 |
| OG0137 | COG0137,COG0649,NOG01372,NOG01375 | C,E,I,R | NDH-1 shuttles electrons from NADH, via FMN and iron- sulfur (Fe-S) centers, to quinones in the respiratory chain. The immediate electron acceptor for the enzyme in this species is believed to be ubiquinone. Couples the redox reaction to proton translocation (for every two electrons transferred, four hydrogen ions are translocated across the cytoplasmic membrane), and thus conserves the redox energy in a proton gradient (By similarity),Aldo keto reductase,Citrulline—aspartate ligase,acyl-CoA dehydrogenase | FS_1738 | TS_1729 |
| OG0139 | COG0139,COG1032 | C,E | Phosphoribosyl-amp cyclohydrolase,radical SAM domain protein | FS_1943 | TS_1535 |
| OG0140 | COG1146,COG1571 | C,R | ATP-dependent agmatine transferase that catalyzes the formation of 2-agmatinylcytidine (agm2C) at the wobble position (C34) of Trna(Ile2), converting the codon specificity from AUG to AUA (By similarity),4fe-4S ferredoxin iroN-sulfur binding | FS_1512 | TS_1972,TS_2013,TS_2083 |
| OG0143 | COG0143,COG1199 | J,L | Is required not only for elongation of protein synthesis but also for the initiation of all Mrna translation through initiator Trna(fMet) aminoacylation (By similarity),helicase | FS_0255,FS_0448,FS_1073,  FS_2594 | TS_0502,TS_0820,TS_1869 |
| OG0144 | COG0524,NOG207164 | G,S | pfkB family carbohydrate kinase,pfkb domain protein | FS_2353 | TS_0028 |
| OG0145 | COG0145,COG0171 | EQ,H | 5-Oxoprolinase,nh(3)-dependent nad synthetase | FS_1077 | TS_0497 |
| OG0146 | COG0146,NOG02536,NOG120467 | EQ,R | 5-Oxoprolinase,Finger protein,MDS1 and EVI1 complex locus | - | - |
| OG0147 | COG0147,COG1611 | E,S | Decarboxylase,synthase (Component I) | FS_2107 | - |
| OG0149 | COG0149,NOG216610,NOG226212 | G,R,S | Sulfite exporter TauE/SafE,triosephosphate isomerase | - | TS_0461 |
| OG0150 | COG0119,COG0150 | E,F | Catalyzes the condensation of the acetyl group of acetyl-CoA with 3-methyl-2-oxobutanoate (2-oxoisovalerate) to form 3-carboxy-3-hydroxy-4-methylpentanoate (2-isopropylmalate) (By similarity),phosphoribosylaminoimidazole synthetase | FS_2749,FS_2751 | TS_0049 |
| OG0151 | COG0151,COG2406 | F,P | Phosphoribosylglycinamide synthetase,ferritin dps family protein | FS_0415 | TS_0848 |
| OG0153 | COG2219 | L | DNA primase is the polymerase that synthesizes small RNA primers for the Okazaki fragments on both template strands at replication forks during chromosomal DNA synthesis. | FS_1084 | TS_1480 |
| OG0154 | COG0154,COG1164 | E,J | Allows the formation of correctly charged Gln-Trna(Gln) through the transamidation of misacylated Glu-Trna(Gln) in organisms which lack glutaminyl-Trna synthetase. The reaction takes place in the presence of glutamine and ATP through an activated gamma-phospho-Glu-Trna(Gln) (By similarity),Oligoendopeptidase f. | FS_2334,FS_2361 | TS_0035 |
| OG0155 | COG0155,COG1945 | C,E | Component of the sulfite reductase complex that catalyzes the 6-electron reduction of sulfite to sulfide. This is one of several activities required for the biosynthesis of L- cysteine from sulfate (By similarity),Pyruvoyl-dependent arginine decarboxylase | FS_0702,FS_2338 | TS_0037 |
| OG0156 | COG0156,COG3182,COG4711,NOG01567 | H,R,S | 8-Amino-7-oxononanoate synthase,PepSY-associated TM helix,integral membrane protein TIGR02587,zinc finger SWIM-type containing 8 | - | - |
| OG0157 | COG0157,NOG01573,NOG83329 | H,S | Domain of unknown function (DUF2024),lipoprotein metabolic process,nicotinate-nucleotide pyrophosphorylase | FS_2086 | TS_0111 |
| OG0158 | COG1909 | S | UPF0218 protein | FS_2079 | - |
| OG0159 | COG0159,COG0420,NOG01591 | E,L,S | Exonuclease,The alpha subunit is responsible for the aldol cleavage of indoleglycerol phosphate to indole and glyceraldehyde 3- phosphate (By similarity),endonuclease exonuclease phosphatase | FS_0168,FS_1212 | TS_0023,TS_0133 |
| OG0161 | COG0161,COG1498 | E,J | Catalyzes the transfer of the alpha-amino group from S- adenosyl-L-methionine (SAM) to 7-keto-8-aminopelargonic acid (KAPA) to form 7,8-diaminopelargonic acid (DAPA). It is the only animotransferase known to utilize SAM as an amino donor (By similarity),Nucleolar protein | FS_1947 | TS_1539 |
| OG0162 | COG0162,COG1617,COG1977 | H,J,S | Catalyzes the attachment of tyrosine to Trna(Tyr) in a two-step reaction tyrosine is first activated by ATP to form Tyr- AMP and then transferred to the acceptor end of Trna(Tyr) (By similarity),Kinase binding protein CGI-121,Molybdopterin converting factor (Subunit 1) | - | - |
| OG0163 | COG0060,COG0163,COG0405,COG0665,NOG01639 | E,H,J,U | Catalyzes the last two steps in the biosynthesis of 5- methylaminomethyl-2-thiouridine (mnm(5)s(2)U) at the wobble position (U34) in Trna. Catalyzes the FAD-dependent demodification of cmnm(5)s(2)U34 to nm(5)s(2)U34, followed by the transfer of a methyl group from S-adenosyl-L-methionine to nm(5)s(2)U34, to form mnm(5)s(2)U34 (By similarity),amino acids such as valine, to avoid such errors it has two additional distinct Trna(Ile)-dependent editing activities. | FS_0320 | TS_2189 |
| OG0164 | COG0164,COG0723 | C,L | Component of the ubiquinol-cytochrome c reductase complex (complex III or cytochrome b-c1 complex), which is a respiratory chain that generates an electrochemical potential coupled to ATP synthesis (By similarity),Endonuclease that specifically degrades the RNA of RNA- DNA hybrids (By similarity) | FS_0306,FS_0363,FS_2478 | TS_2176 |
| OG0165 | COG0139,COG0165,NOG01655 | E,U | Phosphoribosyl-amp cyclohydrolase,Protocadherin 7,argininosuccinate lyase | FS_1135 | - |
| OG0166 | COG0107,COG0166 | E,G | IGPS catalyzes the conversion of PRFAR and glutamine to IGP, AICAR and glutamate. The HisF subunit catalyzes the cyclization activity that produces IGP and AICAR from PRFAR using the ammonia provided by the HisH subunit (By similarity),phosphohexose isomerase | FS_1134 | - |
| OG0167 | COG0106,COG0167 | E,F | Catalyzes the conversion of dihydroorotate to orotate,phosphoribosylformimino-5-aminoimidazole carboxamide ribotide isomerase | FS_1133 | - |
| OG0168 | COG0118,COG0168 | E,P | IGPS catalyzes the conversion of PRFAR and glutamine to IGP, AICAR and glutamate. The hisH subunit provides the glutamine amidotransferase activity that produces the ammonia necessary to hisF for the synthesis of IGP and AICAR (By similarity),Low-affinity potassium transport system. Interacts with Trk system potassium uptake protein TrkA (By similarity) | FS_1132 | - |
| OG0169 | COG0131,COG0169 | E | Imidazoleglycerolphosphate dehydratase,shikimate dehydrogenase | FS_1131 | - |
| OG0170 | COG0546,NOG01704 | R,U | Superfamily hydrolase, subfamily ia, variant,golgin B1 | FS_1130 | - |
| OG0171 | COG0171,COG1096 | H,J | Exosome complex,nh(3)-dependent nad( ) synthetase | FS_1119 | TS_1787 |
| OG0172 | COG0172,COG1634 | J,R | Catalyzes the attachment of serine to Trna(Ser). Is also able to aminoacylate Trna(Sec) with serine, to form the misacylated Trna L-seryl-Trna(Sec), which will be further converted into selenocysteinyl-Trna(Sec) (By similarity),Catalyzes the transfer of pyrophosphate from ATP to 6- hydroxymethyl-7,8-dihydropterin (6-HMD), leading to 6- hydroxymethyl-7,8-dihydropterin diphosphate (6-HMDP) (By similarity) | FS_1112 | TS_1793 |
| OG0173 | COG0173,NOG118475 | J,S | Aspartyl-trna synthetase | - | TS_1795 |
| OG0174 | COG0174,COG2266 | E,H | Adenosylcobinamide-phosphate guanylyltransferase,glutamine synthetase | - | TS_1057 |
| OG0175 | COG0175,COG1492 | H,P | Catalyzes amidations at positions B, D, E, and G on adenosylcobyrinic A,C-diamide. NH(2) groups are provided by glutamine, and one molecule of ATP is hydrogenolyzed for each amidation (By similarity),Reduction of activated sulfate into sulfite (By similarity) | FS_2630 | TS_1060 |
| OG0176 | COG0176 | G | Transaldolase is important for the balance of metabolites in the pentose-phosphate pathway (By similarity) | FS_0018 | TS_1697 |
| OG0177 | COG0177,COG1985 | H,L | Converts 2,5-diamino-6-(ribosylamino)-4(3h)-pyrimidinone 5’-phosphate into 5-amino-6-(ribosylamino)-2,4(1h,3h)- pyrimidinedione 5’-phosphate (By similarity),endonuclease III | FS_1798,FS_2646 | TS_1076,TS_1499 |
| OG0178 | COG0044,COG0178 | F,L | The UvrABC repair system catalyzes the recognition and processing of DNA lesions. UvrA is an ATPase and a DNA-binding protein. A damage recognition complex composed of 2 UvrA and 2 UvrB subunits scans DNA for abnormalities. When the presence of a lesion has been verified by UvrB, the UvrA molecules dissociate (By similarity),Dihydroorotase | FS_2617 | TS_2034 |
| OG0179 | COG0179,COG2429 | Q,S | Catalyzes the formation of 2-amino-5-formylamino-6- ribofuranosylamino-4(3H)-pyrimidinone ribonucleotide monophosphate and inorganic phosphate from GTP. Also has an independent pyrophosphate phosphohydrolase activity (By similarity),Fumarylacetoacetate hydrolase | FS_2649 | TS_1079 |
| OG0180 | COG0180,COG2301 | G,J | Citrate lyase, tryptophanyltRNA synthetase | FS_2653 | TS_1083 |
| OG0181 | COG0181,COG1108 | H,P | ABC transporter,Tetrapolymerization of the monopyrrole PBG into the hydroxymethylbilane pre-uroporphyrinogen in several discrete steps (By similarity) | FS_0500,FS_2673 | TS_1109 |
| OG0182 | COG0182,COG0452 | H,J | Catalyzes the interconversion of methylthioribose-1- phosphate (MTR-1-P) into methylthioribulose-1-phosphate (MTRu-1-P) (By similarity),Phosphopantothenoylcysteine decarboxylase | FS_1215 | TS_1153 |
| OG0184 | COG0184,COG0462 | F,J | Forms an intersubunit bridge (bridge B4) with the 23S Rrna of the 50S subunit in the ribosome (By similarity),Phosphoribosyl pyrophosphate synthase | - | TS_1882 |
| OG0185 | COG0185,COG1971,COG2322 | J,S | Probably functions as a manganese efflux pump (By similarity),Protein S19 forms a complex with S13 that binds strongly to the 16S ribosomal RNA (By similarity),membrAne | FS_1054 | TS_0674 |
| OG0186 | COG0186,COG1933 | J,L | One of the primary Rrna binding proteins, it binds specifically to the 5’-end of 16S ribosomal,Possesses two activities a DNA synthesis (polymerase) and an exonucleolytic activity that degrades single stranded DNA in the 3’- to 5’-direction. Has a template-primer preference which is characteristic of a replicative DNA polymerase (By similarity) | FS_2573 | TS_1858 |
| OG0187 | COG0071 | O | Heat shock protein | FS_1541,FS_2728 | TS_1844 |
| OG0189 | COG0189,COG1204 | HJ,L | Responsible for the addition of glutamate residues to the C-terminus of ribosomal protein S6 (By similarity),helicase | FS_0981 | TS_1771 |
| OG0190 | COG0190,COG0673 | H,R | Catalyzes the oxidation of 5,10- methylenetetrahydrofolate to 5,10-methenyltetrahydrofolate and then the hydrolysis of 5,10-methenyltetrahydrofolate to 10- formyltetrahydrofolate (By similarity),oxidoreductase | FS_0986 | TS_1765 |
| OG0191 | COG0191,COG0491 | G,R | Aldolase,Beta-lactamase domain protein | FS_0341 | TS_2301 |
| OG0192 | COG0085,COG0192 | H,K | Catalyzes the formation of S-adenosylmethionine from methionine and ATP,DNA-dependent RNA polymerase catalyzes the transcription of DNA into RNA using the four ribonucleoside triphosphates as substrates (By similarity) | FS_0813 | - |
| OG0193 | COG0192 | H | Catalyzes the formation of S-adenosylmethionine from methionine and ATP | FS_0932,FS_1385 | TS_1452 |
| OG0194 | COG0468 | L | Can catalyze the hydrolysis of ATP in the presence of single-stranded D the ATP-dependent uptake of single-stranded DNA by duplex D and the ATP-dependent hybridization of homologous single-stranded DNAs. It interacts with LexA causing its activation and leading to its autocatalytic cleavage (By similarity) | - | - |
| OG0195 | COG0078,COG0195 | E,K | Transcription elongation factor NusA,ornithine carbamoyltransferase | FS_2484 | TS_1918 |
| OG0196 | COG2019 | F | adenylate kinase | FS_2490 | TS_1927 |
| OG0197 | COG0197,COG0579 | C,J | Binds 23S Rrna and is also seen to make contacts with the A and possibly P site tRNAs (By similarity),malate dehydrogenase (quinone) | FS_0721 | TS_2268,TS_2314 |
| OG0198 | COG0065,COG0198 | E,J | Catalyzes the isomerization between 2-isopropylmalate and 3-isopropylmalate, via the formation of 2-isopropylmaleate (By similarity),One of the proteins that surrounds the polypeptide exit tunnel on the outside of the subunit (By similarity) | FS_2012 | TS_1947 |
| OG0199 | COG0199,COG1500 | J | Binds 16S Rr required for the assembly of 30S particles and may also be responsible for determining the conformation of the 16S Rrna at the A site (By similarity),Ribosome maturation protein | FS_1998 | TS_1957 |
| OG0200 | COG0200,COG1439 | J,R | Binds to the 23S Rrna (By similarity),NIN1 RPN12 binding protein 1 homolog (S. cerevisiae) | FS_1507 | TS_1968 |
| OG0202 | COG0037,COG0202,COG0533,NOG82025 | D,K,O,S | Ligates lysine onto the cytidine present at position 34 of the AUA codon-specific Trna(Ile) that contains the anticodon CAU, in an ATP-dependent manner. Cytidine is converted to lysidine, thus changing the amino acid specificity of the Trna from methionine to isoleucine (By similarity),ANK,DNA-dependent RNA polymerase catalyzes the transcription of DNA into RNA using the four ribonucleoside triphosphates as substrates (By similarity),Required for the formation of a threonylcarbamoyl group on adenosine at position 37 (t(6)A37) in tRNAs that read codons beginning with adenine (By similarity) | FS_0319,FS_1999,FS_2253 | TS_1956,TS_2188 |
| OG0203 | COG1564,COG3303,NOG07952,NOG40852 | H,M,P,S | Plays a role in nitrite reduction (By similarity),glycosyl transferase group 1,thiamine pyrophosphokinase | - | TS_2190 |
| OG0204 | COG0541 | U | Involved in targeting and insertion of nascent membrane proteins into the cytoplasmic membrane. Binds to the hydrophobic signal sequence of the ribosome-nascent chain (RNC) as it emerges from the ribosomes. The SRP-RNC complex is then targeted to the cytoplasmic membrane where it interacts with the SRP receptor FtsY | FS_0647 | TS_0948 |
| OG0205 | COG0169,NOG02056,NOG02059 | A,E,O | PRP19 PSO4 pre-Mrna processing factor 19 homolog (S. cerevisiae),shikimate dehydrogenase,ubiquitin protein ligase E3 component (N-recognin) | FS_0672 | TS_0907 |
| OG0206 | COG0206,NOG02065,NOG08365,NOG128497 | D,R,S | Fanconi anemia, complementation group I,Essential cell division protein that forms a contractile ring structure (Z ring) at the future cell division site. The regulation of the ring assembly controls the timing and the location of cell division. One of the functions of the FtsZ ring is to recruit other cell division proteins to the septum to produce a new cell wall between the dividing cells. Binds GTP and shows GTPase activity (By similarity),Paralysed flagella protein,Putative esterase | FS_2613 | - |
| OG0207 | COG0432 | S | Secondary thiamine-phosphate synthase enzyme | FS_2612 | - |
| OG0209 | COG0209,COG1196 | D,F | Provides the precursors necessary for DNA synthesis. Catalyzes the biosynthesis of deoxyribonucleotides from the corresponding ribonucleotides (By similarity),Required for chromosome condensation and partitioning (By similarity) | FS_1171 | TS_0063 |
| OG0210 | COG0210,COG0624 | E,L | Catalyzes the hydrolysis of N-succinyl-L,L- diaminopimelic acid (SDAP), forming succinate and LL-2,6- diaminoheptanedioate (DAP), an intermediate involved in the bacterial biosynthesis of lysine and meso-diaminopimelic acid, an essential component of bacterial cell walls (By similarity),helicase | FS_0378,FS_0560,FS_0633 | TS_0455,TS_1295,TS_2156 |
| OG0211 | COG0229,NOG02111,NOG02113 | I,M,O | Glycosyl transferase, family 2,)-reductase,Lipid-transfer protein | FS_0859,FS_1516 | TS_1975 |
| OG0212 | COG0212,COG2233,NOG108006,NOG89234 | F,H,S | 5-formyltetrahydrofolate cyclo-ligase,Protein of unknown function (DUF3278),integral membrane protein,permease | FS_0192 | TS_0141 |
| OG0213 | COG0088,COG0213 | F,J | One of the primary Rrna binding proteins, this protein initially binds near the 5’-end of the 23S Rrna. It is important during the early stages of 50S assembly. It makes multiple contacts with different domains of the 23S Rrna in the assembled 50S subunit and ribosome (By similarity),The enzymes which catalyze the reversible phosphorolysis of pyrimidine nucleosides are involved in the degradation of these compounds and in their utilization as carbon and energy sources, or in the rescue of pyrimidine bases for nucleotide synthesis (By similarity) | FS_1056 | TS_0672 |
| OG0214 | COG0214,COG2106 | H,S | Involved in the production of pyridoxal phosphate, probably by incorporating ammonia into the pyridine ring (By similarity),chromosome 9 open reading frame 114 | FS_1059 | TS_0667 |
| OG0215 | COG0132,COG0215 | H,J | Catalyzes a mechanistically unusual reaction, the ATP- dependent insertion of CO2 between the N7 and N8 nitrogen atoms of 7,8-diaminopelargonic acid (DAPA) to form an ureido ring (By similarity),Cysteinyl-Trna synthetase | FS_1024 | TS_2123 |
| OG0216 | NOG02113 | I | Lipid-transfer protein | FS_2055,FS_2664 | TS_1102 |
| OG0217 | NOG101696 | R | Glyoxalase Bleomycin resistance protein (Dioxygenase | FS_1299 | - |
| OG0227 | COG0064 | J | Allows the formation of correctly charged Asn-Trna(Asn) or Gln-Trna(Gln) through the transamidation of misacylated Asp- Trna(Asn) or Glu-Trna(Gln) in organisms which lack either or both of asparaginyl-Trna or glutaminyl-Trna synthetases. The reaction takes place in the presence of glutamine and ATP through an activated phospho-Asp-Trna(Asn) or phospho-Glu-Trna(Gln) (By similarity) | FS_1980 | - |
| OG0228 | COG0154 | J | Allows the formation of correctly charged Gln-Trna(Gln) through the transamidation of misacylated Glu-Trna(Gln) in organisms which lack glutaminyl-Trna synthetase. The reaction takes place in the presence of glutamine and ATP through an activated gamma-phospho-Glu-Trna(Gln) (By similarity) | FS_1979 | - |
| OG0229 | COG0229,COG1759 | F,O | Catalyzes the ATP- and formate-dependent formylation of 5-aminoimidazole-4-carboxamide-1-beta-d-ribofuranosyl 5’- monophosphate (AICAR) to 5-formaminoimidazole-4-carboxamide-1- beta-d-ribofuranosyl 5’-monophosphate (FAICAR) in the absence of folates (By similarity) | FS_1974 | - |
| OG0231 | COG0231,COG0537 | FG,J | Involved in peptide bond synthesis. Stimulates efficient translation and peptide-bond synthesis on native or reconstituted 70S ribosomes in vitro. Probably functions indirectly by altering the affinity of the ribosome for aminoacyl-Tr thus increasing their reactivity as acceptors for peptidyl transferase (By similarity),histidine triad (Hit) protein | FS_2526 | TS_1473 |
| OG0232 | COG0129 | E | Dihydroxy-acid dehydratase | FS_2523 | TS_0014 |
| OG0233 | COG0147 | E | Synthase (Component I) | FS_2726 | TS_0018 |
| OG0234 | COG0547 | E | Anthranilate phosphoribosyltransferase | FS_2724 | TS_0021 |
| OG0235 | COG0134 | E | Indole-3-glycerol phosphate synthase | FS_2723 | TS_0022 |
| OG0236 | COG0159 | E | The alpha subunit is responsible for the aldol cleavage of indoleglycerol phosphate to indole and glyceraldehyde 3- phosphate (By similarity) | FS_1212 | TS_0023 |
| OG0237 | COG0237,COG0504 | F,H | Catalyzes the ATP-dependent amination of UTP to CTP with either L-glutamine or ammonia as the source of nitrogen (By similarity),Catalyzes the phosphorylation of the 3’-hydroxyl group of dephosphocoenzyme A to form coenzyme A (By similarity) | FS_2349 | TS_0025 |
| OG0238 | COG0061,COG0238,COG0640 | G,J,K | Binds as a heterodimer with protein S6 to the central domain of the 16S Rr where it helps stabilize the platform of the 30S subunit (By similarity),Transcriptional regulator, arsr family,Catalyzes the phosphorylation of NAD to NADP. Utilizes ATP and other nucleoside triphosphates as well as inorganic polyphosphate as a source of phosphorus (By similarity) | FS_2352 | TS_0027 |
| OG0239 | COG0239,COG4830 | D,J | Protein CrcB homolog,Ribosomal protein | FS_2355 | TS_0029 |
| OG0240 | COG0240,COG0558 | C,I | cdp-diacylglycerol-glycerol-3-phosphate 3-phosphatidyltransferase,glycerol-3-phosphate dehydrogenase | FS_2356 | TS_0030 |
| OG0241 | COG0010,COG0241,NOG02412 | E,G | D,d-heptose 1,7-bisphosphate phosphatase,agmatinase (EC 3.5.3.11),paraoxonase | FS_0392,FS_0708,FS_1326 | TS_0484 |
| OG0242 | COG0242,COG0441, NOG02426 | G,J | Maltose permease,Removes the formyl group from the N-terminal Met of newly synthesized proteins. Requires at least a dipeptide for an efficient rate of reaction. N-terminal L-methionine is a prerequisite for activity but the enzyme has broad specificity at other positions (By similarity),Threonyl-trna synthetase | FS_1323 | - |
| OG0244 | COG0244,NOG104173 | J | 30S ribosomal protein S25E,50s ribosomal protein L10 | FS_2119 | TS_0473 |
| OG0245 | COG2164 | S | Pfam:DUF369 | FS_2118 | TS_0474 |
| OG0246 | COG1758 | K | Promotes RNA polymerase assembly. Latches the N- and C- terminal regions of the beta’ subunit thereby facilitating its interaction with the beta and alpha subunits (By similarity) | FS_2117 | TS_0475 |
| OG0247 | COG2522 | K | Transcriptional regulator | FS_2115 | TS_0477 |
| OG0248 | COG0248,COG0367,NOG02485,NOG02488 | E,FP,P,S | Neutral invertase,Phospholipid-transporting atpase,asparagine synthetase,ppx gppa phosphatase | FS_2114 | TS_0479 |
| OG0250 | COG0250,COG0667 | C,K | Participates in transcription elongation, termination and antitermination (By similarity),Aldo keto reductase | FS_2110 | TS_0481 |
| OG0251 | COG0251,COG1575 | H,J | 1,4-dihydroxy-2-naphthoate octaprenyltransferase,Endoribonuclease LPSP | FS_0394,FS_0908 | TS_0482,TS_0619,TS_0737, TS_1631 |
| OG0252 | COG0252,COG0760 | E,O | L-asparaginase,peptidyl-prolyl cis-trans isomerase | FS_2309 | - |
| OG0253 | COG2131,NOG02536 | F,R | MDS1 and EVI1 complex locus,deaminase | FS_2095 | - |
| OG0254 | COG0417,COG1372 | L | DNA polymerase,Provides the precursors necessary for DNA synthesis. Catalyzes the biosynthesis of deoxyribonucleotides from the corresponding ribonucleotides (By similarity) | FS_2094 | - |
| OG0255 | COG0149,COG0255 | G,J | 50s ribosomal protein l29,triosephosphate isomerase | FS_0246,FS_0870,FS_1051 | TS_0532,TS_0677 |
| OG0256 | COG0256,COG3620 | J,K | This is one of the proteins that binds and probably mediates the attachment of the 5S RNA into the large ribosomal subunit, where it forms part of the central protuberance (By similarity),transcriptional regulator XRE family | FS_0244 | TS_0523 |
| OG0257 | COG0346 | E | Glyoxalase bleomycin resistance protein dioxygenase | FS_0240 | TS_0518 |
| OG0258 | COG0258,COG1884 | I,L | Structure-specific nuclease with 5’-flap endonuclease and 5’-3’ exonuclease activities involved in DNA replication and repair. During DNA replication, cleaves the 5’-overhanging flap structure that is generated by displacement synthesis when DNA polymerase encounters the 5’-end of a downstream Okazaki fragment. It enters the flap from the 5’-end and then tracks to cleave the flap base, leaving a nick for ligation. Also involved in the long patch base excision repair (LP-BER) pathway, by cleaving within the apurinic apyrimidinic (AP) site-terminated flap. | FS_0238,FS_0525 | TS_0517 |
| OG0259 | COG1703 | E | LAO/AO transport system ATPase | FS_0526 | TS_0516 |
| OG0260 | COG0308 | E | Aminopeptidase | FS_2465 | TS_1351 |
| OG0261 | COG2185 | I | Methylmalonyl-coA mutase | FS_0527 | TS_0515 |
| OG0262 | COG0059,COG0262 | E,H | Key enzyme in folate metabolism. Catalyzes an essential reaction for de novo glycine and purine synthesis, and for DNA precursor synthesis (By similarity),Alpha-keto-beta-hydroxylacyl reductoisomerase | FS_0850 | TS_0514 |
| OG0263 | COG3276,NOG02631 | J,O | FAD-dependent oxidoreductase domain containing 2,Selenocysteine-specific translation elongation factor | FS_1188 | TS_0084 |
| OG0264 | COG0143 | J | Is required not only for elongation of protein synthesis but also for the initiation of all Mrna translation through initiator Trna(fMet) aminoacylation (By similarity) | FS_2594 | TS_0503 |
| OG0265 | COG0265,COG2146,NOG02656 | C,O,T | Histidine kinase,nitrite reductase NADPH small subunit,serine protease | FS_1076 | TS_0500 |
| OG0266 | COG0215 | J | Cysteinyl-Trna synthetase | FS_0607 | TS_0496 |
| OG0267 | COG1602 | S | Archaeal protein of unknown function (DUF650) | FS_1185 | TS_0081 |
| OG0268 | NOG30935 | P | Periplasmic binding protein | FS_0945 | TS_1883 |
| OG0269 | COG0269,COG1832 | G,R | CoA-binding domain protein,synthase | FS_2756 | TS_0490 |
| OG0270 | COG0270,COG0340,COG1654 | H,L | Biotin- acetyl-CoA-carboxylase ligase,Cytosine-specific methyltransferase | FS_1745,FS_2764 | TS_0485 |
| OG0273 | COG0001 | H | Glutamate-1-semialdehyde aminotransferase | FS_2429 | TS_2111 |
| OG0274 | COG1917 | S | Cupin 2 Conserved Barrel Domain Protein | - | - |
| OG0275 | COG0484 | O | ATP binding to DnaK triggers the release of the substrate protein, thus completing the reaction cycle. Several rounds of ATP-dependent interactions between DnaJ, DnaK and GrpE are required for fully efficient folding. Also involved, together with DnaK and GrpE, in the DNA replication of plasmids through activation of initiation proteins (By similarity) | FS_0648,FS_1143 | TS_0152,TS_0298,TS_1454 |
| OG0276 | COG0537 | FG | Histidine triad (Hit) protein | FS_2526 | TS_1473 |
| OG0277 | COG0277,COG1250 | C,I | Dehydrogenase,FAD linked oxidase domain protein | FS_2106 | - |
| OG0278 | COG0068,NOG01041,NOG18553 | O,S | Putative amidoligase enzyme,cylindromatosis (turban tumor syndrome),hydrogenase maturation protein Hypf | FS_2175 | - |
| OG0279 | COG0279,COG1996,NOG02793,NOG02796 | G,K,R,S | Catalyzes the isomerization of sedoheptulose 7-phosphate in D-glycero-D-manno-heptose 7-phosphate (By similarity),DNA-dependent RNA polymerase catalyzes the transcription of DNA into RNA using the four ribonucleoside triphosphates as substrates,NHL repeat | FS_2177 | - |
| OG0280 | COG0051 | J | Involved in the binding of Trna to the ribosomes (By similarity) | FS_2178 | - |
| OG0281 | COG0281,COG5256 | C,J | This protein promotes the GTP-dependent binding of aminoacyl-Trna to the A-site of ribosomes during protein biosynthesis (By similarity),malic enzyme | FS_2179 | - |
| OG0282 | COG0282,COG1793 | C,L | Catalyzes the formation of acetyl phosphate from acetate and ATP. Can also catalyze the reverse reaction (By similarity),DNA ligase | FS_2123 | TS_0471 |
| OG0283 | NOG02838,NOG160148 | P,S | phosphate transport | FS_2126 | TS_0469 |
| OG0284 | COG0284,COG1676 | F,J | Sites to release the intron. The products are an intron and two Trna half-molecules bearing 2’,3’ cyclic phosphate and 5’-OH termini. Recognizes a pseudosymmetric substrate in which 2 bulged loops of 3 bases are separated by a stem of 4 bp (By similarity),Catalyzes the decarboxylation of orotidine 5’- monophosphate (OMP) to uridine 5’-monophosphate (UMP) (By similarity) | FS_2127 | TS_0467 |
| OG0285 | COG1028,COG4221,NOG02855 | IQ,O,R | Dehydrogenase,matrix metallopeptidase,short-chain dehydrogenase reductase | FS_2129 | TS_0463 |
| OG0286 | COG0286,COG0648 | L,V | Endonuclease IV plays a role in DNA repair. It cleaves phosphodiester bonds at apurinic or apyrimidinic sites (AP sites) to produce new 5’-ends that are base-free deoxyribose 5-phosphate residues. It preferentially attacks modified AP sites created by bleomycin and neocarzinostatin (By similarity),type I restriction-modification system | FS_1491 | TS_1481 |
| OG0287 | COG0287,COG1430 | E,S | Exported protein,prephenate dehydrogenase | FS_0667,FS_1228 | TS_0634,TS_0761,TS_0915 |
| OG0288 | COG1321,NOG02881 | K,S | Iron (metal) dependent repressor, dtxr family,KIAA0319-like | FS_1230 | TS_0759 |
| OG0289 | COG1855 | R | K06865 ATPase | FS_1231 | TS_0758 |
| OG0290 | COG1573,NOG02905 | L,S | Uracil-dna glycosylase | FS_1238 | TS_0751 |
| OG0291 | COG2094,NOG02914 | L,P | 3-Methyladenine DNA glycosylase,Binding-protein-dependent transport systems inner membrane component | FS_1239 | TS_0750 |
| OG0292 | COG0444 | EP | (ABC) transporter | FS_1167 | TS_0060 |
| OG0293 | COG0017,COG0293 | J | Specifically methylates the uridine in position 2552 of 23S Rrna at the 2’-O position of the ribose in the fully assembled 50S ribosomal subunit (By similarity),asparaginyl-Trna synthetase | FS_1162,FS_2744 | TS_0055 |
| OG0294 | COG0294,COG0473 | E,H | Catalyzes the oxidation of 3-carboxy-2-hydroxy-4- methylpentanoate (3-isopropylmalate) to 3-carboxy-4-methyl-2- oxopentanoate. The product decarboxylates to 4-methyl-2 oxopentanoate (By similarity),dihydropteroate synthase | FS_2747 | TS_0050 |
| OG0295 | COG0440 | E | Acetolactate synthase small subunit | - | TS_0048 |
| OG0296 | COG0028 | E | Acetolactate synthase | FS_1013 | TS_0045 |
| OG0297 | COG1830 | G | Aldolase | FS_0683,FS_2345 | TS_0042,TS_0897 |
| OG0298 | COG1063 | C | Dehydrogenase | FS_2343 | TS_0040 |
| OG0299 | COG0299,NOG16105,NOG42470 | F,O,S | Phosphatidylinositol-specific phospholipase C X domain containing,lipoprotein transmembrane,phosphoribosylglycinamide formyltransferase | FS_2215, FS_2470 | TS_0569,TS_0586,TS_0587, TS_1034,TS_1044 |
| OG0300 | COG0300,COG5431 | R,S | Zinc finger, swim domain protein,Dehydrogenase reductase | FS_2472 | TS_0567 |
| OG0301 | COG0301,COG1407 | H,R | Catalyzes the ATP-dependent transfer of a sulfur to Trna to produce 4-thiouridine in position 8 of tRNAs, which functions as a near-UV photosensor. Also catalyzes the transfer of sulfur to the sulfur carrier protein ThiS, forming ThiS-thiocarboxylate. This is a step in the synthesis of thiazole, in the thiamine biosynthesis pathway. The sulfur is donated as persulfide by IscS (By similarity),metallophosphoesterase | FS_2473 | TS_0566,TS_1047 |
| OG0302 | COG0302,COG0505 | F,H | GTP cyclohydrolase i,carbamoyl-phosphate synthetase glutamine chain | FS_1471,FS_2228,FS_2229, FS_2475,FS_2680 | TS_0553,TS_0557,TS_1517 |
| OG0303 | COG0464,NOG03035 | O,S | Aaa atpase | FS_2227 | TS_0316,TS_0320,TS_0404, TS_0550 |
| OG0304 | COG0251,COG0304 | I,J | Catalyzes the condensation reaction of fatty acid synthesis by the addition to an acyl acceptor of two carbons from malonyl-ACP (By similarity),Endoribonuclease LPSP | FS_0908 | TS_0619,TS_0737 |
| OG0305 | COG0608 | L | Single-stranded-DNA-specific exonuclease (RecJ) | FS_0909 | TS_0618,TS_0736 |
| OG0306 | COG0306,COG0372 | C,P | citrate synthase,phosphate transporter | FS_0911 | TS_0615,TS_0734 |
| OG0307 | COG0307,COG0322 | H,L | riboflavin synthase, subunit alpha,The UvrABC repair system catalyzes the recognition and processing of DNA lesions. UvrC both incises the 5’ and 3’ sides of the lesion. The N-terminal half is responsible for the 3’ incision and the C-terminal half is responsible for the 5’ incision (By similarity) | FS_2218 | TS_0591,TS_1031 |
| OG0308 | COG0178,COG0308 | E,L | The UvrABC repair system catalyzes the recognition and processing of DNA lesions. UvrA is an ATPase and a DNA-binding protein. A damage recognition complex composed of 2 UvrA and 2 UvrB subunits scans DNA for abnormalities. When the presence of a lesion has been verified by UvrB, the UvrA molecules dissociate (By similarity),aminopeptidase | FS_0926,FS_2220 | TS_0594,TS_0717 |
| OG0309 | COG0309,COG0556 | L,O | Damaged site, the DNA wraps around one UvrB monomer. DNA wrap is dependent on ATP binding by UvrB and probably causes local melting of the DNA helix, facilitating insertion of UvrB beta-hairpin between the DNA strands. Then UvrB probes one DNA strand for the presence of a lesion. If a lesion is found the UvrA subunits dissociate and the UvrB-DNA preincision complex is formed. This complex is subsequently bound by UvrC and the second UvrB is released. If no lesion is found, the DNA wraps around the other UvrB subunit that will check the other stand for damage (By similarity),Hydrogenase expression formation protein HypE | FS_0924 | TS_0598,TS_0718 |
| OG0310 | COG3467 | S | Resistance protein | FS_0923 | TS_0599,TS_0719 |
| OG0311 | COG0311,COG2141 | C,H | Involved in the hydrolysis of glutamine to glutamate and ammonia. Channels an ammonia molecule to PdxS (By similarity),Monooxygenase | FS_0920 | TS_0601,TS_0721 |
| OG0312 | COG0312,COG1454 | C,R | Alcohol dehydrogenase,peptidase U62 modulator of DNA gyrase | FS_0919 | TS_0603,TS_0723 |
| OG0313 | COG0436 | E | Aminotransferase | FS_0295 | TS_2164 |
| OG0314 | COG2379,NOG03145 | G,R | Alkaline phosphatase,hydroxypyruvate reductase | FS_0916 | TS_0611,TS_0730 |
| OG0315 | COG1236 | J | Cleavage and polyadenylation | FS_2216 | TS_0588,TS_1033 |
| OG0316 | COG0299,COG0316 | F,S | Iron—sulfur cluster,phosphoribosylglycinamide formyltransferase | FS_0571,FS_2215,FS_2470 | TS_0569,TS_0586,TS_1034, TS_1044,TS_1280 |
| OG0317 | COG0317,NOG83660 | KT,S | Cupin 2 Conserved Barrel Domain Protein,In eubacteria ppGpp (guanosine 3’-diphosphate 5-‘ diphosphate) is a mediator of the stringent response that coordinates a variety of cellular activities in response to changes in nutritional abundance (By similarity) | FS_2214 | TS_1036 |
| OG0318 | COG0190,COG0318 | H,I | Catalyzes the oxidation of 5,10- methylenetetrahydrofolate to 5,10-methenyltetrahydrofolate and then the hydrolysis of 5,10-methenyltetrahydrofolate to 10- formyltetrahydrofolate (By similarity),Amp-dependent synthetase and ligase | FS_2213 | TS_0575,TS_1038 |
| OG0319 | COG0212 | H | 5-Formyltetrahydrofolate cyclo-ligase | FS_2212 | TS_0573,TS_1039 |
| OG0320 | COG0151 | F | Phosphoribosylglycinamide synthetase | FS_2470 | TS_0570,TS_1044 |
| OG0321 | COG0060 | J | Amino acids such as valine, to avoid such errors it has two additional distinct Trna(Ile)-dependent editing activities. One activity is designated as ‘pretransfer’ editing and involves the hydrolysis of activated Val-AMP. The other activity is designated ‘posttransfer’ editing and involves deacylation of mischarged Val-Trna(Ile) (By similarity) | FS_2223,FS_2500 | TS_0542 |
| OG0322 | COG0322,NOG35811 | L,T | Histidine kinase,The UvrABC repair system catalyzes the recognition and processing of DNA lesions. UvrC both incises the 5’ and 3’ sides of the lesion. The N-terminal half is responsible for the 3’ incision and the C-terminal half is responsible for the 5’ incision (By similarity) | - | TS_0540 |
| OG0323 | COG0045,COG0323, NOG03237 | C,L,O | DnaJ (Hsp40) homolog, subfamily B, member,This protein is involved in the repair of mismatches in DNA. It is required for dam-dependent methyl-directed DNA mismatch repair. May act as a molecular matchmaker , a protein that promotes the formation of a stable complex between two or more DNA-binding proteins in an ATP-dependent manner without itself being part of a final effector complex (By similarity),Succinyl-CoA synthetase subunit beta | FS_2498 | TS_0538 |
| OG0324 | COG0074 | C | Succinyl-CoA ligase ADP-forming subunit alpha | FS_2497 | TS_0535 |
| OG0325 | COG0341,COG0597 | MU,U | Part of the Sec protein translocase complex. Interacts with the SecYEG preprotein conducting channel. SecDF uses the proton motive force (PMF) to complete protein translocation after the ATP-dependent function of SecA (By similarity),This protein specifically catalyzes the removal of signal peptides from prolipoproteins (By similarity) | - | - |
| OG0326 | COG2085 | R | NADP oxidoreductase, coenzyme f420-dependent | FS_0298 | TS_2167 |
| OG0327 |  |  |  | - | - |
| OG0328 | COG0328,COG0716,NOG12974 | C,L,S | Endonuclease that specifically degrades the RNA of RNA- DNA hybrids (By similarity),Low-potential electron donor to a number of redox enzymes (By similarity) | FS_0902 | TS_0633 |
| OG0329 | COG0329,COG1331 | E,O | Catalyzes the condensation of (S)-aspartate-beta- semialdehyde (S)-ASA and pyruvate to 4-hydroxy- tetrahydrodipicolinate (HTPA) (By similarity),spermatogenesis-associated protein | FS_0903 | TS_0624,TS_0741 |
| OG0330 | COG0330,COG0406 | G,O | Band 7 protein,Phosphoglycerate mutase | FS_0300 | TS_2168 |
| OG0331 | COG1990 | S | The natural substrate for this enzyme may be peptidyl- tRNAs which drop off the ribosome during protein synthesis (By similarity) | FS_1196 | TS_0100 |
| OG0332 | COG0644 | C | Geranylgeranyl reductase | FS_1198 | TS_0102 |
| OG0333 | COG0333,COG2092 | J | Promotes the exchange of GDP for GTP in EF-1-alpha GDP, thus allowing the regeneration of EF-1-alpha GTP that could then be used to form the ternary complex EF-1-alpha GTP AAtRNA (By similarity),50s ribosomal protein l32 | - | TS_0103 |
| OG0334 | COG0334,NOG85495 | E,S | Glutamate dehydrogenase | FS_2091 | TS_0107 |
| OG0335 | COG0109,NOG03355,NOG03359 | O,S | Converts heme B (protoheme IX) to heme O by substitution of the vinyl group on carbon 2 of heme B porphyrin ring with a hydroxyethyl farnesyl side group (By similarity),K13735 adhesin invasin,abortive phage infection | FS_2090 | TS_0108 |
| OG0336 | COG3794,NOG102528,NOG181274,NOG181892 | C,P,S | Copper binding proteins, plastocyanin/azurin family,Blue (Type 1) copper domain protein | FS_2089 | TS_0089,TS_0109, TS_0297 |
| OG0337 | COG0337,COG1310 | E,R | Component of the eukaryotic translation initiation factor 3 (Eif-3) complex, which is involved in protein synthesis and, together with other initiation factors, stimulates binding of Mrna and methionyl-tRNAi to the 40S ribosome (By similarity),3-dehydroquinate synthase | FS_2087 | TS_0110 |
| OG0338 | COG0338,COG1622,NOG03389 | C,L,V | Dna adenine methylase,Efflux transporter RND family MFP subunit,Subunits I and II form the functional core of the enzyme complex. Electrons originating in cytochrome c are transferred via heme a and Cu(A) to the binuclear center formed by heme a3 and Cu(B) (By similarity) | FS_2329 | TS_2257 |
| OG0339 | COG0489 | D | ATP-binding protein | FS_2078 | TS_0118 |
| OG0340 | COG0340,COG1095 | H,K | Biotin- acetyl-CoA-carboxylase ligase,DNA-directed RNA Polymerase | FS_2077 | TS_0119 |
| OG0341 | COG0341,COG2093 | K,U | DNA-directed RNA polymerase, subunit e’,Part of the Sec protein translocase complex. Interacts with the SecYEG preprotein conducting channel. SecDF uses the proton motive force (PMF) to complete protein translocation after the ATP-dependent function of SecA (By similarity) | FS_2076 | TS_0120 |
| OG0342 | COG0157 | H | Nicotinate-nucleotide pyrophosphorylase | FS_2074 | TS_0121 |
| OG0343 | COG0343,COG0379,NOG03438 | H,I,J | Exchanges the guanine residue with 7-aminomethyl-7- deazaguanine in tRNAs with GU(N) anticodons (Trna-Asp, -Asn, -His and —Tyr). After this exchange, a cyclopentendiol moiety is attached to the 7-aminomethyl group of 7-deazaguanine, resulting in the hypermodified nucleoside queuosine (Q) (7-(((4,5-cis- dihydroxy-2-cyclopenten-1-yl)amino)methyl)-7-deazaguanosine) (By similarity),Catalyzes the condensation of iminoaspartate with dihydroxyacetone phosphate to form quinolinate (By similarity),acyl-CoA dehydrogenase | FS_2073 | TS_0123 |
| OG0344 | COG1712,NOG03441 | H,V | Specifically catalyzes the NAD or NADP-dependent dehydrogenation of L-aspartate to iminoaspartate (By similarity),restriction endonuclease | FS_0161 | TS_0125 |
| OG0345 | COG0345,COG1612 | E,O | Catalyzes the oxidation of the C8 methyl side group on heme O porphyrin ring into a formyl group (By similarity),pyrroline-5-carboxylate reductase | FS_2325 | TS_2254 |
| OG0346 | COG0346,NOG92028 | E,O | Alkyl hydroperoxide reductase Thiol specific antioxidant Mal allergen, glyoxalase bleomycin resistance protein dioxygenase | FS_2763 | TS_0486 |
| OG0347 | COG0347,COG1632 | E,J | Nitrogen regulatory protein pii,Ribosomal protein L15 | FS_1283,FS_2234 | TS_1337 |
| OG0348 | COG5637 | S | Cyclase dehydrase | - | - |
| OG0349 | COG0111 | E | Dehydrogenase | FS_2231 | TS_1333 |
| OG0350 | COG0350,COG1027 | E,L | Aspartate ammonia-lyase,Involved in the cellular defense against the biological effects of O6-methylguanine (O6-MeG) in DNA. Repairs alkylated guanine in DNA by stoichiometrically transferring the alkyl group at the O-6 position to a cysteine residue in the enzyme. This is a suicide reaction the enzyme is irreversibly inactivated (By similarity) | FS_0530,FS_0624,FS_0840 | TS_1329,TS_1330,TS_1906 |
| OG0351 | COG0351,COG0621 | H,J | Catalyzes the methylthiolation of N6- (dimethylallyl)adenosine (i(6)A), leading to the formation of 2- methylthio-N6-(dimethylallyl)adenosine (ms(2)i(6)A) at position 37 in tRNAs that read codons beginning with uridine (By similarity),phosphomethylpyrimidine kinase | FS_0531 | TS_0917,TS_1329 |
| OG0352 | COG0206 | D | Essential cell division protein that forms a contractile ring structure (Z ring) at the future cell division site. The regulation of the ring assembly controls the timing and the location of cell division. One of the functions of the FtsZ ring is to recruit other cell division proteins to the septum to produce a new cell wall between the dividing cells. Binds GTP and shows GTPase activity (By similarity) | FS_0532 | TS_1327 |
| OG0353 | COG2097 | J | (Ribosomal) protein | FS_0533 | TS_1326 |
| OG0354 | COG0494 | L | NUDiX hydrolase | FS_0535 | TS_1323 |
| OG0355 | COG0685 | E | Methylenetetrahydrofolate reductase | FS_0539 | TS_1319 |
| OG0356 | COG1410,NOG03564 | B,E | TOX high mobility group box family member 4,methionine synthase | FS_0536 | TS_1322 |
| OG0357 | COG0646 | E | Methionine synthase | FS_0538 | TS_1320 |
| OG0358 | COG0358,COG1116 | L,P | (ABC) transporter,DNA primase is the polymerase that synthesizes small RNA primers for the Okazaki fragments on both template strands at replication forks during chromosomal DNA synthesis (By similarity) | FS_0545 | TS_1312 |
| OG0359 | COG0137 | E | Citrulline—aspartate ligase | FS_0546 | TS_1310 |
| OG0360 | COG0189,NOG03607 | HJ,R | Responsible for the addition of glutamate residues to the C-terminus of ribosomal protein S6 (By similarity),alpha-2-macroglobulin | FS_0548,FS_0559 | TS_1296,TS_1308 |
| OG0361 | COG0002,COG0361 | E,J | It seems to stimulate more or less all the activities of the other two initiation factors, IF-2 and IF-3 (By similarity), N-acetylglutamate semialdehyde dehydrogenase | FS_0550 | TS_1305 |
| OG0362 | COG0548 | E | nag kinase | FS_0553 | TS_1303 |
| OG0363 | COG4992 | E | Acetylornithine aminotransferase | FS_0554 | TS_1302 |
| OG0364 | COG1522 | K | Transcriptional regulator AsnC family | FS_0555 | TS_1300 |
| OG0365 | COG0119,COG0365 | E,I | Catalyzes the conversion of acetate into acetyl-CoA (AcCoA), an essential intermediate at the junction of anabolic and catabolic pathways. AcsA undergoes a two-step reaction. In the first half reaction, AcsA combines acetate with ATP to form acetyl-adenylate (AcAMP) intermediate. In the second half reaction, it can then transfer the acetyl group from AcAMP to the sulfhydryl group of CoA, forming the product AcCoA (By similarity),Catalyzes the condensation of the acetyl group of acetyl-CoA with 3-methyl-2-oxobutanoate (2-oxoisovalerate) to form 3-carboxy-3-hydroxy-4-methylpentanoate (2-isopropylmalate) (By similarity) | FS_0557,FS_1226,FS_1227, FS_1724,FS_2749 | TS_0049,TS_0762,TS_1299 |
| OG0366 | COG0366,NOG181278 | G,S | Alpha amylase, catalytic | FS_0547,FS_0558 | TS_1298,TS_1309 |
| OG0367 | COG0189,COG0367 | E,HJ | Responsible for the addition of glutamate residues to the C-terminus of ribosomal protein S6 (By similarity),asparagine synthetase | FS_0548,FS_0559 | TS_1296,TS_1297,TS_1308 |
| OG0368 | COG0368,COG0624 | E,H | Catalyzes the hydrolysis of N-succinyl-L,L- diaminopimelic acid (SDAP), forming succinate and LL-2,6- diaminoheptanedioate (DAP), an intermediate involved in the bacterial biosynthesis of lysine and meso-diaminopimelic acid, an essential component of bacterial cell walls (By similarity),Joins Ado-cobinamide-GDP and alpha-ribazole to generate adenosylcobalamin (Ado-cobalamin) (By similarity) | FS_0560 | TS_1295 |
| OG0369 | COG0369,COG1798,COG1849 | J,P,S | S-adenosyl-L-methionine-dependent methyltransferase that catalyzes the trimethylation of the amino group of the modified target histidine residue in translation elongation factor 2 (EF- 2), to form an intermediate called diphthine. The three successive methylation reactions represent the second step of diphthamide biosynthesis (By similarity),Component of the sulfite reductase complex that catalyzes the 6-electron reduction of sulfite to sulfide. This is one of several activities required for the biosynthesis of L- cysteine from sulfate. The flavoprotein component catalyzes the electron flow from NADPH — FAD — FMN to the hemoprotein component (By similarity),Cytidyltransferase-related domain protein | FS_0561 | TS_1293 |
| OG0370 | COG0615 | IM | Cytidylyltransferase | FS_0562 | TS_1292 |
| OG0371 | COG0371,COG1339 | C,H | Catalyzes the CTP-dependent phosphorylation of riboflavin (vitamin B2) to form flavin mononucleotide (FMN) (By similarity),Dehydrogenase | FS_0563 | TS_1291 |
| OG0372 | COG0358,COG0372 | C,L | DNA primase is the polymerase that synthesizes small RNA primers for the Okazaki fragments on both template strands at replication forks during chromosomal DNA synthesis (By similarity),citrate synthase | FS_0564,FS_0911 | TS_0615,TS_0734,TS_1289 |
| OG0373 | COG0316,COG0373 | H,S | Catalyzes the NADPH-dependent reduction of glutamyl- Trna(Glu) to glutamate 1-semialdehyde (GSA) (By similarity),Iron—sulfur cluster | FS_0571,FS_1417 | TS_0976,TS_1280 |
| OG0374 | COG0374,COG1042 | C | Hydrogenase, large subunit,CoA-binding domain protein | FS_0569 | TS_1283 |
| OG0376 | COG0334,COG0376 | E,P | Bifunctional enzyme with both catalase and broad- spectrum peroxidase activity (By similarity),glutamate dehydrogenase | FS_0574 | TS_1277 |
| OG0377 | COG0377,COG2226,NOG03778,NOG03779 | C,H,R,S | NDH-1 shuttles electrons from NADH, via FMN and iron- sulfur (Fe-S) centers, to quinones in the respiratory chain. The immediate electron acceptor for the enzyme in this species is believed to be ubiquinone. Couples the redox reaction to proton translocation, and thus conserves the redox energy in a proton gradient (By similarity),Domain of unknown function(DUF2779), Methyltransferase required for the conversion of demethylmenaquinone (DMKH2) to menaquinone (MKH2),tripartite motif containing | FS_0578 | TS_1275 |
| OG0378 | COG0115,COG0378 | E,KO | Facilitates the functional incorporation of the urease nickel metallocenter. This process requires GTP hydrolysis, probably effectuated by UreG (By similarity),brancheD-chain amino acid aminotransferase | FS_0792,FS_0988 | TS_2240 |
| OG0379 | COG0379,COG1826 | H,U | Catalyzes the condensation of iminoaspartate with dihydroxyacetone phosphate to form quinolinate (By similarity),Part of the twin-arginine translocation (Tat) system that transports large folded proteins containing a characteristic twin-arginine motif in their signal peptide across membranes | FS_0575 | TS_1276 |
| OG0380 | COG3569,NOG88336 | L | DNA topoisomerase type IB small subunit,Dna topoisomerase | FS_0581 | TS_1268 |
| OG0381 | COG0381,COG0527 | E,M | Aspartokinase,UDP-N-acetylglucosamine 2-epimerase | FS_0789,FS_2404 | TS_2237 |
| OG0382 | COG0382,COG1819 | CG,H | Synthesis of 3-octaprenyl-4-hydroxybenzoate (By similarity),glycosyltransferase | FS_2400 | - |
| OG0383 | COG2912 | S | Transcriptional regulator | FS_0585 | TS_0827 |
| OG0384 | COG0473 | E | Catalyzes the oxidation of 3-carboxy-2-hydroxy-4- methylpentanoate (3-isopropylmalate) to 3-carboxy-4-methyl-2- oxopentanoate. The product decarboxylates to 4-methyl-2 oxopentanoate (By similarity) | FS_0588,FS_2747 | TS_0050,TS_1948 |
| OG0385 | COG2890,NOG03855 | J,S | Methylates the class 1 translation termination release factors RF1 PrfA and RF2 PrfB on the glutamine residue of the universally conserved GGQ motif (By similarity),Radial spoke | FS_0591 | - |
| OG0386 | COG0030,NOG03864 | J,P | Specifically dimethylates two adjacent adenosines (A1518 and A1519) in the loop of a conserved hairpin near the 3’-end of 16S Rrna in the 30S particle. May play a critical role in biogenesis of 30S subunits (By similarity),binding-protein-dependent transport systems inner membrane Component | FS_0592 | - |
| OG0387 | COG1491 | J | RNA-binding protein | FS_0593 | - |
| OG0388 | COG0388,COG1460,NOG103501 | K,R | DNA-directed RNA polymerase subunit f,RNA Polymerase,nitrilase cyanide hydratase and apolipoprotein n-acyltransferase | FS_0594 | - |
| OG0389 | COG0389,COG0468 | L | Can catalyze the hydrolysis of ATP in the presence of single-stranded D the ATP-dependent uptake of single-stranded DNA by duplex D and the ATP-dependent hybridization of homologous single-stranded DNAs. It interacts with LexA causing its activation and leading to its autocatalytic cleavage (By similarity),Poorly processive error-prone DNA polymerase involved in untargeted mutagenesis. Copies undamaged DNA at stalled replication forks which arise in vivo from mismatched or misaligned primer ends. These misaligned primers can be extended by polIV. Exhibits no 3-5 exonuclease (proofreading) activity. May be involved in translesional synthesis in conjunction with the beta clamp from polIII (By similarity) | FS_0596,FS_1182,FS_1387 | TS_0076 |
| OG0390 | COG4919 | J | 30S Ribosomal protein | FS_1942 | TS_1534 |
| OG0391 | COG0391,COG0503 | F,S | Catalyzes a salvage reaction resulting in the formation of AMP, that is energically less costly than de novo synthesis (By similarity),Catalyzes the transfer of the 2-phospholactate moiety from lactyl (2) diphospho-(5’)guanosine (LPPG) to 7,8-didemethyl- 8-hydroxy-5-deazariboflavin (FO) with the formation of the L- lactyl phosphodiester of 7,8-didemethyl-8-hydroxy-5- deazariboflavin (F420-0) and GMP (By similarity) | FS_0158 | TS_1553 |
| OG0392 | COG0005,COG0392 | F,S | The purine nucleoside phosphorylases catalyze the phosphorolytic breakdown of the N-glycosidic bond in the beta- (deoxy)ribonucleoside molecules, with the formation of the corresponding free purine bases and pentose-1-phosphate (By similarity),Membrane | FS_0157 | TS_1554 |
| OG0393 | COG0151,COG0841,COG4943 | F,P,T | Eal domain protein,Phosphoribosylglycinamide synthetase,acriflavin resistance protein | FS_0156 | TS_1555 |
| OG0394 | COG0394 | T | PHOsphatase | FS_0155 | TS_1556 |
| OG0395 | COG0395,COG1378,NOG03950 | G,K,T | Binding-protein-dependent transport systems inner membrane component,Diguanylate cyclase,Transcriptional regulator | FS_0154 | TS_1557 |
| OG0396 | COG0396,COG1697 | L,O | DNA topoisomerase VI, subunit A,feS assembly ATPase SufC | - | - |
| OG0397 | COG1094,NOG03975 | R | Zinc finger, MYM-type,Required for 40S ribosome biogenesis. Involved in nucleolar processing of pre-18S ribosomal RNA and ribosome assembly (By similarity) | FS_0149 | TS_1564 |
| OG0398 | COG2412,NOG03983 | M,S | Protein of unknown function (DUF424),dolichyl-phosphate beta-D-mannosyltransferase (EC 2.4.1.83) | FS_0145 | TS_1567 |
| OG0399 | COG0399,COG1601,NOG03998 | E,J,S | DegT DnrJ EryC1 StrS aminotransferase,chromosome 4 open reading frame 27,translation Initiation Factor | FS_0144 | TS_1568 |
| OG0400 | COG2237 | S | Domain of unknown function (DUF373) | FS_0136 | TS_1571 |
| OG0401 | COG0020 | I | Catalyzes the condensation of isopentenyl diphosphate (IPP) with allylic pyrophosphates generating different type of terpenoids (By similarity) | FS_0133 | TS_1572 |
| OG0402 | COG0402,COG0494 | F,L | NUDiX hydrolase,deaminase | FS_0132 | - |
| OG0403 | COG0284 | F | Catalyzes the decarboxylation of orotidine 5’- monophosphate (OMP) to uridine 5’-monophosphate (UMP) (By similarity) | FS_0131 | TS_1574 |
| OG0404 | COG1100 | R | GTP-binding Protein | FS_0130 | TS_1576 |
| OG0405 | COG0104,COG0405 | E,F | Gamma-glutamyltranspeptidase (EC 2.3.2.2),Plays an important role in the de novo pathway of purine nucleotide biosynthesis | FS_0129 | TS_1577 |
| OG0406 | COG0406,COG3321,NOG00186 | G,Q,U | Component of the adaptor protein complex 2 (AP-2). Adaptor protein complexes function in protein transport via transport vesicles in different membrane traffic pathways. Adaptor protein complexes are vesicle coat components and appear to be involved in cargo selection and vesicle formation. AP-2 is involved in clathrin-dependent endocytosis in which cargo proteins are incorporated into vesicles surrounded by clathrin (clathrin- coated vesicles, CCVs) which are destined for fusion with the early endosome. The clathrin lattice serves as a mechanical scaffold but is itself unable to bind directly to membrane components. | FS_0128 | - |
| OG0407 | COG0834,COG2924 | E,P | Could be a mediator in iron transactions between iron acquisition and iron-requiring processes, such as synthesis and or repair of Fe-S clusters in biosynthetic enzymes (By similarity),(ABC) transporter | - | TS_1579 |
| OG0408 |  |  |  | FS_0125 | TS_1580 |
| OG0409 | COG0009 | J | Sua5 ycio yrdc ywlc family protein | FS_0121 | TS_1585 |
| OG0410 | COG1818,NOG04104 | R,S | Protein of unknown function (DUF3500),thump domain | - | TS_1586 |
| OG0411 | COG1383 | J | Ribosomal protein | FS_0118 | TS_1590 |
| OG0412 | COG0084,COG0412 | L,Q | Dienelactone hydrolase,tatd family | FS_0117 | TS_1591 |
| OG0413 | COG0413,COG2036,NOG04131,NOG04133 | B,H,S | Catalyzes the reversible reaction in which hydroxymethyl group from 5,10-methylenetetrahydrofolate is tranferred onto alpha-ketoisovalerate to form ketopantoate (By similarity),Core component of nucleosome. Nucleosomes wrap and compact DNA into chromatin, limiting DNA accessibility to the cellular machineries which require DNA as a template. Histones thereby play a central role in transcription regulation, DNA repair, DNA replication and chromosomal stability. DNA accessibility is regulated via a complex set of post-translational modifications of histones, also called histone code, and nucleosome remodeling,Chromosome 14 open reading frame 101,domain) protein | FS_0116 | TS_1593 |
| OG0414 | COG0414,COG3432 | H,K | Catalyzes the condensation of pantoate with beta-alanine in an ATP-dependent reaction via a pantoyl-adenylate intermediate (By similarity) | FS_0112,FS_0123,FS_0440, FS_1463 | TS_1523,TS_1583,TS_1597 |
| OG0415 | COG1109,NOG04158 | G,S | Catalyzes the conversion of glucosamine-6-phosphate to glucosamine-1-phosphate (By similarity),nhl repeat containing protein | FS_0110 | TS_1599 |
| OG0416 | COG0611 | H | Catalyzes the ATP-dependent phosphorylation of thiamine- monophosphate (TMP) to form thiamine-pyrophosphate (TPP), the active form of vitamin B1 (By similarity) | FS_0108 | TS_1601 |
| OG0417 | COG0100,COG0417 | J,L | Located on the platform of the 30S subunit, it bridges several disparate RNA helices of the 16S Rrna. Forms part of the Shine-Dalgarno cleft in the 70S ribosome (By similarity),DNA polymerase | FS_0107 | TS_1604 |
| OG0418 | NOG100510 | S |  | FS_0105 | TS_1606 |
| OG0419 | COG0015,COG0419 | F,L | Involved in DNA double-strand break repair (DSBR). The Rad50 Mre11 complex possesses single-strand endonuclease activity and ATP-dependent double-strand-specific 3’-5’ exonuclease activity. Rad50 provides an ATP-dependent control of Mre11 by unwinding and or repositioning DNA ends into the Mre11 active site (By similarity),adenylosuccinate lyase | FS_0101 | TS_1613 |
| OG0420 | COG0420,COG2154 | H,L | Exonuclease,Pterin CarBinolamine Dehydratase | FS_0099 | - |
| OG0422 | COG0422,COG0525 | H,J | Amino acids such as threonine, to avoid such errors, it has a posttransfer editing activity that hydrolyzes mischarged Thr-Trna(Val) in a Trna-dependent manner (By similarity),Catalyzes the synthesis of the hydroxymethylpyrimidine phosphate (HMP-P) moiety of thiamine from aminoimidazole ribotide (AIR) in a radical S-adenosyl-L-methionine (SAM)-dependent reaction (By similarity) | FS_0097,FS_0665 | TS_0918,TS_1618 |
| OG0423 | COG0311,COG0423 | H,J | Catalyzes the attachment of glycine to Trna(Gly) (By similarity),Involved in the hydrolysis of glutamine to glutamate and ammonia. Channels an ammonia molecule to PdxS (By similarity) | FS_0089 | TS_1624 |
| OG0424 | COG0214,NOG04248 | H,R | Involved in the production of pyridoxal phosphate, probably by incorporating ammonia into the pyridine ring (By similarity),repeat-containing protein | FS_0087 | TS_1625 |
| OG0425 | COG0425,COG1032 | C,O | Part of a sulfur-relay system required for 2-thiolation of 5-methylaminomethyl-2-thiouridine (mnm(5)s(2)U) at Trna wobble positions. Interacts with IscS and stimulates its activity. Then, accepts a sulfur from IscS and transfers it in turn to TusD (By similarity),radical SAM domain protein | FS_0806,FS_2399 | TS_2057 |
| OG0426 | COG0057,NOG12594 | G,S | Fras1 related extracellular matrix protein 2, glyceraldehyde3phosphate dehydrogenase | FS_0083 | TS_1629 |
| OG0427 | COG0072,NOG04275 | J,R | Polyamine oxidase,phenylalanyl-Trna synthetase (beta subunit) | FS_0078 | TS_1634 |
| OG0428 | COG0016,COG0428 | J,P | phenylalanyL-Trna synthetase, alpha subunit,zinc transporter | FS_0077 | TS_1635 |
| OG0429 | COG0180 | J | TryptophanyltRNA synthetase | FS_0076 | TS_1641 |
| OG0430 | COG0430 | A | Catalyzes the conversion of 3’-phosphate to a 2’,3’- cyclic phosphodiester at the end of RNA. The mechanism of action of the enzyme occurs in 3 steps (A) adenylation of the enzyme by ATP | FS_0075 | - |
| OG0431 | COG0338,COG0431 | L,R | DNA adenine methylasedph-dependent fmn reductase | FS_0051 | TS_1663 |
| OG0432 | COG0432,NOG01567,NOG04329,NOG175901 | R,S | Secondary thiamine-phosphate synthase enzyme,Gcn5-related N-acetyltransferase,zinc finger SWIM-type containing 8 | FS_0050 | TS_1664 |
| OG0433 | COG0433,NOG04337,NOG11046 | C,R,S | Monooxygenase, subunit B,Aaa atpase | FS_0047 | TS_1667 |
| OG0434 |  |  |  | FS_0032,FS_0189,FS_0578, FS_1974, FS_2177,FS_2657 | TS_0139,TS_1089,TS_1275, TS_1683 |
| OG0435 | COG0184 | J | Forms an intersubunit bridge (bridge B4) with the 23S Rrna of the 50S subunit in the ribosome (By similarity) | FS_0030 | TS_1685 |
| OG0436 | COG0436,COG0608 | E,L | Single-stranded-DNA-specific exonuclease (RecJ),aminotransferase | FS_0029 | TS_1686 |
| OG0437 | COG0328,COG0437 | C,L | 4Fe-4S Ferredoxin iron-sulfur binding domain protein,Endonuclease that specifically degrades the RNA of RNA- DNA hybrids (By similarity) | - | - |
| OG0438 | COG0172,COG0438 | J,M | Catalyzes the attachment of serine to Trna(Ser). Is also able to aminoacylate Trna(Sec) with serine, to form the misacylated Trna L-seryl-Trna(Sec), which will be further converted into selenocysteinyl-Trna(Sec) (By similarity),Glycosyl transferase (Group 1 | FS_0027 | TS_1687 |
| OG0439 | COG0439,COG1890 | I,J | Ribosomal protein,acetyl-CoA carboxylase biotin carboxylase | FS_0026 | TS_1688 |
| OG0440 | COG0440,COG1325,NOG04405,NOG04408 | E,J,S,T | Acetolactate synthase small subunit,Adenylate guanylate Cyclase,exosome subunit | - | - |
| OG0441 | COG0441,COG0644 | C,J | Threonyl-trna synthetase,geranylgeranyl reductase | FS_0024 | TS_1690 |
| OG0442 | COG0442,COG0714 | J,R | Catalyzes the attachment of proline to Trna(Pro) in a two-step reaction proline is first activated by ATP to form Pro- AMP and then transferred to the acceptor end of Trna(Pro). As ProRS can inadvertently accommodate and process non-cognate amino acids such as alanine and cysteine, to avoid such errors it has two additional distinct editing activities against alanine. One activity is designated as ‘pretransfer’ editing and involves the Trna(Pro)-independent hydrolysis of activated Ala-AMP. The other activity is designated ‘posttransfer’ editing and involves deacylation of mischarged Ala-Trna(Pro). | FS_0022 | TS_1692 |
| OG0443 | COG0443,COG0503,COG4548 | F,O,P | Catalyzes a salvage reaction resulting in the formation of AMP, that is energically less costly than de novo synthesis (By similarity),heat shock protein 70,von Willebrand factor type A | FS_0020 | TS_1694 |
| OG0444 | COG0444,COG1042 | C,EP | (ABC) transporter,CoA-binding domain protein | FS_0506,FS_2394 | TS_2050 |
| OG0445 | COG0127,COG0445 | D,F | NAD-binding protein involved in the addition of a carboxymethylaminomethyl (cmnm) group at the wobble position (U34) of certain tRNAs, forming Trna-cmnm(5)s(2)U34 (By similarity),Pyrophosphatase that hydrolyzes non-canonical purine nucleotides such as XTP and ITP Ditp to their respective monophosphate derivatives. Might exclude non-canonical purines from DNA precursor pool, thus preventing their incorporation into DNA and avoiding chromosomal lesions (By similarity) | FS_0322 | TS_2191 |
| OG0446 | COG0446,COG3642 | C,T | Required for the formation of a threonylcarbamoyl group on adenosine at position 37 (t(6)A37) in tRNAs that read codons beginning with adenine (By similarity),pyridine nucleotide-disulfide oxidoreductase | FS_0321 | TS_2190 |
| OG0447 | COG0533,NOG04478 | O,T | Required for the formation of a threonylcarbamoyl group on adenosine at position 37 (t(6)A37) in tRNAs that read codons beginning with adenine (By similarity),serine threonine-protein kinase d | FS_0319 | TS_2188 |
| OG0448 | COG0012,COG0448 | G,J | Catalyzes the synthesis of ADP-glucose, a sugar donor used in elongation reactions on alpha-glucans (By similarity),gtp-binding protein | FS_0318,FS_0608,FS_1441 | TS_0492,TS_2187 |
| OG0449 | COG0449,COG1936 | F,M | Catalyzes the first step in hexosamine metabolism, converting fructose-6P into glucosamine-6P using glutamine as a nitrogen source (By similarity),Broad-specificity nucleoside monophosphate (NMP) kinase that catalyzes the reversible transfer of the terminal phosphate group between nucleoside triphosphates and monophosphates | FS_0317 | TS_2186 |
| OG0450 | COG0343,COG0450 | J,O | Exchanges the guanine residue with 7-aminomethyl-7- deazaguanine in tRNAs with GU(N) anticodons (Trna-Asp, -Asn, -His and —Tyr). After this exchange, a cyclopentendiol moiety is attached to the 7-aminomethyl group of 7-deazaguanine, resulting in the hypermodified nucleoside queuosine (Q) (7-(((4,5-cis- dihydroxy-2-cyclopenten-1-yl)amino)methyl)-7-deazaguanosine) (By similarity),alkyl hydroperoxide reductase | FS_0316 | TS_2185 |
| OG0451 | COG0451,COG2368 | M,Q | 4-Hydroxyphenylacetated-dependent epimerase dehydratase | FS_0505 | TS_2048 |
| OG0452 | COG0452,COG1236 | H,J | Phosphopantothenoylcysteine decarboxylase,cleavage and polyadenylation | FS_0313 | TS_2182 |
| OG0453 | COG0122 | L | Glycosylase | FS_0311 | TS_2181 |
| OG0454 | COG0454,COG3980 | K,M | Acetyltransferase,pseudaminic acid biosynthesis-associated protein PseG | - | TS_2180 |
| OG0455 | COG1290,NOG04556 | C,S | Component of the ubiquinol-cytochrome c reductase complex (complex III or cytochrome b-c1 complex), which is a respiratory chain that generates an electrochemical potential coupled to ATP synthesis (By similarity),ATP-dependent endonuclease of the OLD family-like protein | FS_0308 | TS_2177 |
| OG0456 | COG0456,NOG04566,NOG199123,NOG210101 | R,T,U | Peptidase S26B, signal peptidase, Histidine kinase,Peptidase S24-like | - | TS_2047 |
| OG0457 | COG0141,COG0457 | E,R | Catalyzes the sequential NAD-dependent oxidations of L- histidinol to L-histidinaldehyde and then to L-histidine (By similarity),repeat-containing protein | FS_1128 | - |
| OG0458 | COG0458,COG1257 | F,I | Carbamoyl-phosphate synthetase ammonia chain | FS_1124 | - |
| OG0459 | COG0459,COG2133 | G,O | Dehydrogenase,Prevents misfolding and promotes the refolding and proper assembly of unfolded polypeptides generated under stress conditions (By similarity) | FS_1123 | - |
| OG0460 | COG0460,COG1571,NOG04603 | E,R | ATP-dependent agmatine transferase that catalyzes the formation of 2-agmatinylcytidine (agm2C) at the wobble position (C34) of Trna(Ile2), converting the codon specificity from AUG to AUA (By similarity),IQ and AAA domain-containing protein,homoserine dehydrogenase | FS_1122 | TS_1783 |
| OG0461 | COG0461,COG1333,COG1873,NOG119515,NOG195296,NOG196080 | F,O,S | Catalyzes the transfer of a ribosyl phosphate group from 5-phosphoribose 1-diphosphate to orotate, leading to the formation of orotidine monophosphate (OMP) (By similarity),sporulation protein, YlmC YmxH,Bacterial SH3 domain,Cytochrome c biogenesis protein,Ubiquitin carboxyl-terminal hydrolase | FS_0862 | TS_2046 |
| OG0462 | COG0077,COG0462 | E,F | Phosphoribosyl pyrophosphate synthase,Prephenate dehydratase | FS_1117 | TS_1788 |
| OG0463 | COG0463,COG4772,NOG03950,NOG04638 | M,P,R,T | Glycosyl transferase, family 2,Diguanylate cyclase,WD repeat domain 19,receptor | - | - |
| OG0464 | COG0464,COG0516 | F,O | Catalyzes the conversion of inosine 5’-phosphate (IMP) to xanthosine 5’-phosphate (XMP), the first committed and rate- limiting step in the de novo synthesis of guanine nucleotides, and therefore plays an important role in the regulation of cell growth (By similarity),Aaa atpase | FS_1115 | TS_1791 |
| OG0465 | COG0465,NOG61803 | O | Acts as a processive, ATP-dependent zinc metallopeptidase for both cytoplasmic and membrane proteins. Plays a role in the quality control of integral membrane proteins (By similarity),Thioredoxin | FS_2092,FS_2249,FS_2436,  FS_2727 | TS_0106,TS_1840,TS_1934 |
| OG0466 | COG1631,NOG04662 | J,U | Endoplasmic reticulumgolgi intermediate compartment protein,Ribosomal protein | FS_2625 | TS_1056 |
| OG0467 | COG0467,COG2051,NOG04671 | J,K,T | 40s ribosomal protein S27,Circadian clock protein KaiC,LysR family (Transcriptional regulator | FS_2626 | TS_1056 |
| OG0468 | COG0368,COG0468 | H,L | Can catalyze the hydrolysis of ATP in the presence of single-stranded D the ATP-dependent uptake of single-stranded DNA by duplex D and the ATP-dependent hybridization of homologous single-stranded DNAs. It interacts with LexA causing its activation and leading to its autocatalytic cleavage (By similarity),Joins Ado-cobinamide-GDP and alpha-ribazole to generate adenosylcobalamin (Ado-cobalamin) (By similarity) | FS_2628 | TS_1058 |
| OG0469 | COG1270 | H | Converts cobyric acid to cobinamide by the addition of aminopropanol on the F carboxylic group (By similarity) | FS_2629 | TS_1059 |
| OG0470 | COG0079,COG0470 | E,L | DNA polymerase III subunit delta’,Imidazole acetol-phosphate transaminase | FS_2631 | TS_1061 |
| OG0471 | COG0471,COG4260,COG5411,NOG75952,NOG87035 | P,S,T | TM2 domain containing protein,Transporter,inositol,virion core protein (Lumpy skin disease | FS_2735 | TS_2042 |
| OG0472 | COG0136,COG0472 | E,M | Catalyzes the NADPH-dependent formation of L-aspartate- semialdehyde (L-ASA) by the reductive dephosphorylation of L- aspartyl-4-phosphate (By similarity),First step of the lipid cycle reactions in the biosynthesis of the cell wall peptidoglycan (By similarity) | FS_2633 | TS_1062 |
| OG0473 | COG0473,COG1522 | E,K | Catalyzes the oxidation of 3-carboxy-2-hydroxy-4- methylpentanoate (3-isopropylmalate) to 3-carboxy-4-methyl-2- oxopentanoate. The product decarboxylates to 4-methyl-2 oxopentanoate (By similarity),transcriptional regulator AsnC family | FS_0081,FS_0206,FS_0429, FS_0556,FS_0588,FS_0769, FS_0893,FS_1065,FS_1121, FS_1176,FS_1298,FS_1954, FS_2048,FS_2238,FS_2313, FS_2747 | TS_0050,TS_0207,TS_0234, TS_0337,TS_0640,TS_0654, TS_0834,TS_0979,TS_1300, TS_1340,TS_1630,TS_1785, TS_1837,TS_1948,TS_2076 |
| OG0474 | COG0109,COG0474 | O,P | Converts heme B (protoheme IX) to heme O by substitution of the vinyl group on carbon 2 of heme B porphyrin ring with a hydroxyethyl farnesyl side group (By similarity),P-type atpase | FS_2635 | TS_1064 |
| OG0475 | COG4260,NOG219992,NOG95407 | S,T | TM2 domain,virion core protein (Lumpy skin disease | FS_2736 | TS_2043 |
| OG0476 | COG0043,COG0476 | H | Catalyzes the decarboxylation of 3-octaprenyl-4-hydroxy benzoate to 2-octaprenylphenol (By similarity),UBA THIF-type NAD FAD binding protein | FS_2636 | TS_1065 |
| OG0477 | COG0477,COG1882,NOG39237 | C,G,S | formate acetyltransferase,major facilitator Superfamily | FS_2637 | TS_1066 |
| OG0478 | NOG15376,NOG55147 | G,S | Glycoside hydrolase family 38 | FS_2639 | TS_1068 |
| OG0479 | COG0479,COG1308 | C,K | Nascent polypeptide-associated complex,succinate dehydrogenase | FS_2640 | TS_1069 |
| OG0480 | COG0480,COG1370,NOG04806 | J,U | Catalyzes the GTP-dependent ribosomal translocation step during translation elongation. During this step, the ribosome changes from the pre-translocational (PRE) to the post- translocational (POST) state as the newly formed A-site-bound peptidyl-Trna and P-site-bound deacylated Trna move to the P and E sites, respectively. Catalyzes the coordinated movement of the two Trna molecules, the Mrna and conformational changes in the ribosome (By similarity),Exchanges the guanine residue with 7-cyano-7- deazaguanine (preQ0) at position 15 in the dihydrouridine loop (D- loop) of archaeal tRNAs (By similarity),Polycystic kidney disease | FS_1444,FS_1550,FS_2641 | TS_0169,TS_1070 |
| OG0481 | COG0481,COG2262 | M,R | Required for accurate and efficient protein synthesis under certain stress conditions. May act as a fidelity factor of the translation reaction, by catalyzing a one-codon backward translocation of tRNAs on improperly translocated ribosomes. Back- translocation proceeds from a post-translocation (POST) complex to a pre-translocation (PRE) complex, thus giving elongation factor G a second chance to translocate the tRNAs correctly. Binds to ribosomes in a GTP-dependent manner (By similarity),GTPase that associates with the 50S ribosomal subunit and may have a role during protein synthesis or ribosome biogenesis (By similarity) | FS_2643 | TS_1072 |
| OG0482 | COG0482,COG1303 | J,S | Catalyzes the 2-thiolation of uridine at the wobble position (U34) of Tr leading to the formation of s(2)U34 (By similarity),Specifically catalyzes the AdoMet-dependent 2’-O-ribose methylation of cytidine at position 56 in tRNAs (By similarity) | FS_2644 | TS_1073 |
| OG0483 | COG0483,COG1675 | G,K | Transcription factor that plays a role in the activation of archaeal genes transcribed by RNA polymerase. Facilitates transcription initiation by enhancing TATA-box recognition by TATA-box-binding protein (Tbp), and transcription factor B (Tfb) and RNA polymerase recruitment. Not absolutely required for transcription in vitro, but particularly important in cases where Tbp or Tfb function is not optimal. It dynamically alters the nucleic acid-binding properties of RNA polymerases by stabilizing the initiation complex and destabilizing elongation complexes. | FS_2645 | TS_1075 |
| OG0484 | COG0484,COG3945 | O,S | ATP binding to DnaK triggers the release of the substrate protein, thus completing the reaction cycle. Several rounds of ATP-dependent interactions between DnaJ, DnaK and GrpE are required for fully efficient folding. Also involved, together with DnaK and GrpE, in the DNA replication of plasmids through activation of initiation proteins (By similarity),hemerythrin hhe cation binding domain protein | FS_0659 | TS_0926 |
| OG0485 | COG0402 | F | Deaminase | FS_2647 | TS_1077 |
| OG0486 | COG0054 | H | Catalyzes the formation of 6,7-dimethyl-8- ribityllumazine by condensation of 5-amino-6-(D- ribitylamino)uracil with 3,4-dihydroxy-2-butanone 4-phosphate. This is the penultimate step in the biosynthesis of riboflavin (By similarity) | FS_2650 | TS_1080 |
| OG0487 | COG0108 | H | Catalyzes the conversion of D-ribulose 5-phosphate to formate and 3,4-dihydroxy-2-butanone 4-phosphate (By similarity) | FS_2651 | TS_1081 |
| OG0488 | COG0307,COG0488 | H,R | Riboflavin synthase, subunit alpha,(ABC) transporter | FS_2652 | TS_1082 |
| OG0489 | COG0489,COG2118 | D,R | ATP-binding protein,DNA-binding protein | FS_2655 | - |
| OG0490 | COG2238 | J | Ribosomal protein | FS_2656 | TS_1088 |
| OG0491 | COG0491,COG2023,NOG04916 | J,R | Part of ribonuclease P, a protein complex that generates mature Trna molecules by cleaving their 5’-ends (By similarity),(ABC) transporter,Beta-lactamase domain protein | FS_0341,FS_2657 | TS_1089,TS_2301 |
| OG0492 | COG0492,COG1756 | J,O | Methyltransferase involved in ribosomal biogenesis. Specifically catalyzes the N1-methylation of the pseudouridine corresponding to position 914 in M.jannaschii 16S Rrna (By similarity),thioredoxin reductase | FS_2658 | TS_1090 |
| OG0493 | COG0209,COG0493 | E,F | Provides the precursors necessary for DNA synthesis. Catalyzes the biosynthesis of deoxyribonucleotides from the corresponding ribonucleotides (By similarity),glutamate synthase | FS_2660 | TS_1096 |
| OG0494 | COG0494,COG1328,NOG04940,NOG62754 | F,K,L,S | Anaerobic ribonucleoside-triphosphate reductase,NUDiX hydrolase,arsR family transcriptional regulator,germination protein ypeb | FS_0132,FS_0535,FS_2661, FS_2713 | TS_1098,TS_1147,TS_1323 |
| OG0495 | COG0495,COG1545 | J,S | Leucyl-trna synthetase,Nucleic-acid-binding protein containing a Zn-ribbon | FS_2663 | TS_1101 |
| OG0496 | COG0183 | I | Acetyl-coa acetyltransferase | FS_2664 | TS_1102 |
| OG0497 | COG0497,COG0551,NOG04974 | L,R | Releases the supercoiling and torsional tension of D which is introduced during the DNA replication and transcription, by transiently cleaving and rejoining one strand of the DNA duplex. Introduces a single-strand break via transesterification at a target site in duplex DNA. The scissile phosphodiester is attacked by the catalytic tyrosine of the enzyme, resulting in the formation of a DNA-(5’-phosphotyrosyl)-enzyme intermediate and the expulsion of a 3’-OH DNA strand. | - | - |
| OG0498 | COG0388,COG0498 | E,R | Nitrilase cyanide hydratase and apolipoprotein n-acyltransferase,threonine synthase | FS_2666 | TS_1104 |
| OG0500 | COG0500,COG0864 | K,Q | Transcriptional regulator,methyltransferase | FS_2678 | TS_1112 |
| OG0501 | COG0501,COG0540 | F,O | Protease HtpX homolog,aspartate transcarbamylase | FS_2679,FS_2680 | TS_1114 |
| OG0502 | COG0502,COG1781 | F,H | Catalyzes the conversion of dethiobiotin (DTB) to biotin by the insertion of a sulfur atom into dethiobiotin via a radical- based mechanism (By similarity),Involved in allosteric regulation of aspartate carbamoyltransferase (By similarity) | FS_1027,FS_2681 | TS_0705,TS_1115 |
| OG0503 | COG0503,COG1394 | C,F | Catalyzes a salvage reaction resulting in the formation of AMP, that is energically less costly than de novo synthesis (By similarity),Produces ATP from ADP in the presence of a proton gradient across the membrane (By similarity) | FS_0158 | TS_1553 |
| OG0504 | COG0504,COG1156 | C,F | Catalyzes the ATP-dependent amination of UTP to CTP with either L-glutamine or ammonia as the source of nitrogen (By similarity),Produces ATP from ADP in the presence of a proton gradient across the membrane. The V-type beta chain is a regulatory subunit (By similarity) | FS_2686 | TS_1120 |
| OG0505 | COG0505,COG1155 | C,F | Produces ATP from ADP in the presence of a proton gradient across the membrane. The V-type alpha chain is a catalytic subunit (By similarity),carbamoyl-phosphate synthetase glutamine chain | FS_2687 | TS_1121 |
| OG0506 | COG0506,COG2897 | E,P | Proline dehydrogenase,sulfurtransferase | FS_2692 | TS_1126 |
| OG0507 | COG0311,COG0507,COG0583,NOG05691 | H,K,L,U | Nephrosis 1, congenital, Finnish type (nephrin),Involved in the hydrolysis of glutamine to glutamate and ammonia. Channels an ammonia molecule to PdxS (By similarity),Transcriptional regulator,exodeoxyribonuclease v alpha | FS_0089 | TS_1624 |
| OG0508 | COG5608,NOG102046,NOG43207 | S | Late embryogenesis abundant protein,water Stress and Hypersensitive response | - | - |
| OG0509 | COG0509,COG1287,COG3279,NOG179830,NOG38268 | E,R,S,T | Oligosaccharyl transferase, STT3 subunit,Histidine kinase,The glycine cleavage system catalyzes the degradation of glycine. The H protein shuttles the methylamine group of glycine from the P protein to the T protein (By similarity),regulator,sh3 type 3 domain-containing protein | FS_2696 | - |
| OG0510 | COG0528 | F | Catalyzes the reversible phosphorylation of UMP to UDP (By similarity) | FS_2697 | TS_1132 |
| OG0511 | COG1078 | R | Metal Dependent Phosphohydrolase | FS_2698 | TS_1133 |
| OG0512 | COG0125,COG0512 | E,F | Phosphorylation of Dtmp to form Dtdp in both de novo and salvage pathways of Dttp synthesis (By similarity),anthranilate synthase | FS_2700 | TS_1134 |
| OG0513 | COG0120,COG0513 | G,L | ATP-dependent rna helicase,phosphoriboisomerase A | FS_2254 | TS_2032 |
| OG0514 | COG0514,COG0704 | L,P | Plays a role in the regulation of phosphate uptake,atp-dependent dna helicase | FS_2712 | TS_1148 |
| OG0515 | COG0515,COG1829 | H,T | Ghmp kinase,Serine Threonine protein kinase | - | TS_1149 |
| OG0516 | COG0516,COG1701 | F,H | Catalyzes the conversion of inosine 5’-phosphate (IMP) to xanthosine 5’-phosphate (XMP), the first committed and rate- limiting step in the de novo synthesis of guanine nucleotides, and therefore plays an important role in the regulation of cell growth (By similarity),pantothenate synthetase | - | TS_1150 |
| OG0517 | COG0413,COG0517 | H,R | Catalyzes the conversion of inosine 5’-phosphate (IMP) to xanthosine 5’-phosphate (XMP), the first committed and rate- limiting step in the de novo synthesis of guanine nucleotides, and therefore plays an important role in the regulation of cell growth (By similarity),Catalyzes the reversible reaction in which hydroxymethyl group from 5,10-methylenetetrahydrofolate is tranferred onto alpha-ketoisovalerate to form ketopantoate (By similarity) | - | TS_1152 |
| OG0518 | COG0006,COG0518,NOG05180 | E,F,U | Autophagy related,Catalyzes the synthesis of GMP from XMP (By similarity),peptidase M24 | - | TS_1185 |
| OG0519 | COG0519,COG1958 | F,K | Catalyzes the synthesis of GMP from XMP (By similarity),small nuclear ribonucleoprotein | FS_0959 | TS_2028 |
| OG0520 | COG0520,COG2450 | E,S | Protein of unknown function (DUF552),cysteine desulfurase | FS_0934 | TS_1188 |
| OG0521 | COG0638 | O | The proteasome is a multicatalytic proteinase complex which is characterized by its ability to cleave peptides with Arg, Phe, Tyr, Leu, and Glu adjacent to the leaving group at neutral or slightly basic Ph. The proteasome has an ATP-dependent proteolytic activity (By similarity) | FS_0717 | TS_1201 |
| OG0522 | COG0371,COG0522 | C,J | One of the primary Rrna binding proteins, it binds directly to 16S Rrna where it nucleates assembly of the body of the 30S subunit (By similarity),Dehydrogenase | FS_0716 | TS_1204 |
| OG0523 | COG1047 | O | Peptidylprolyl cistrans isomerase | FS_0524 | TS_1205 |
| OG0524 | COG0075,COG0524 | E,G | Aminotransferase,pfkb domain protein | FS_0521 | TS_1213 |
| OG0525 | COG0525,COG1056,NOG05252,NOG05258 | H,J,S | Component of the NOP7 complex, which is required for maturation of the 25S and 5.8S ribosomal RNAs and formation of the 60S ribosome (By similarity),amino acids such as threonine, to avoid such errors, it has a posttransfer editing activity that hydrolyzes mischarged Thr-Trna(Val) in a Trna-dependent manner (By similarity),Nicotinamide-nucleotide adenylyltransferase,fg-gap repeat protein | FS_1607 | TS_1215 |
| OG0526 | COG0526,COG1198,COG1996,COG4888,NOG00088 | K,L,O,R,S | DNA-dependent RNA polymerase catalyzes the transcription of DNA into RNA using the four ribonucleoside triphosphates as substrates,Primosomal protein n’,Thioredoxin,elongation factor 1 homolog | FS_0496 | - |
| OG0527 | COG0527,COG1402,NOG05274 | E,P,R | ATPase (type,Aspartokinase,K01470 creatinine amidohydrolase EC 3.5.2.10 | FS_0960 | TS_2027 |
| OG0528 | COG0528,COG1276,NOG69306 | F,P,S | Catalyzes the reversible phosphorylation of UMP to UDP (By similarity),copper resistance | FS_1610 | TS_1242 |
| OG0529 | COG0529,COG5608 | P,S | Catalyzes the synthesis of activated sulfate (By similarity),water Stress and Hypersensitive response | - | - |
| OG0530 | COG0530,COG0778,NOG05302 | C,P,V | RND family efflux transporter mfp subunit,calcium exchanger,nitroreductase | FS_1616 | TS_1250,TS_1799 |
| OG0531 | COG0531,COG0652,NOG05314 | E,O,S | PPIases accelerate the folding of proteins,amino acid | FS_1624 | TS_1466,TS_1806 |
| OG0532 | COG0532,COG0788 | F,J | One of the essential components for the initiation of protein synthesis. Protects formylmethionyl-Trna from spontaneous hydrolysis and promotes its binding to the 30S ribosomal subunits. Also involved in the hydrolysis of GTP during the formation of the 70S ribosomal complex (By similarity),formyltetrahydrofolate deformylase | FS_0961 | TS_2026 |
| OG0533 | COG0524,COG0533,NOG05332 | G,O,R | Laminin, gamma 1 (formerly LAMB2),Required for the formation of a threonylcarbamoyl group on adenosine at position 37 (t(6)A37) in tRNAs that read codons beginning with adenine (By similarity),pfkb domain protein | FS_1630 | TS_1812 |
| OG0534 | COG0534,NOG05341,NOG61007 | S,V | Mate efflux family protein,dUTPase | FS_1631 | TS_1813 |
| OG0535 | COG0477,COG0535 | G,R | Major facilitator Superfamily,radical SAM domain protein | FS_1635 | TS_1818 |
| OG0536 | COG0162 | J | Catalyzes the attachment of tyrosine to Trna(Tyr) in a two-step reaction tyrosine is first activated by ATP to form Tyr- AMP and then transferred to the acceptor end of Trna(Tyr) (By similarity) | FS_1638 | TS_1819 |
| OG0537 | COG0537,COG1109 | FG,G | Catalyzes the conversion of glucosamine-6-phosphate to glucosamine-1-phosphate (By similarity),histidine triad (Hit) protein | FS_0963 | TS_2025 |
| OG0538 | COG0538,COG1010 | C,H | Precorrin-3B C17-methyltransferase,isocitrate dehydrogenase (NADp) | FS_0589,FS_0692 | TS_1822 |
| OG0539 | COG0527,NOG05395 | E,R | C3 and PZP-like, alpha-2-macroglobulin domain containing 8,Aspartokinase | FS_2301 | TS_1825 |
| OG0540 | COG0540,COG0592,NOG05404 | F,L,S | DNA polymerase III is a complex, multichain enzyme responsible for most of the replicative synthesis in bacteria. This DNA polymerase also exhibits 3’ to 5’ exonuclease activity. The beta chain is required for initiation of replication once it is clamped onto D it slides freely (bidirectional and ATP- independent) along duplex DNA (By similarity),WD repeat domain 65,aspartate transcarbamylase | FS_2302 | TS_1827 |
| OG0541 | COG0541,COG1594 | K,U | DNA-dependent RNA polymerase catalyzes the transcription of DNA into RNA using the four ribonucleoside triphosphates as substrates (By similarity),Involved in targeting and insertion of nascent membrane proteins into the cytoplasmic membrane. Binds to the hydrophobic signal sequence of the ribosome-nascent chain (RNC) as it emerges from the ribosomes. The SRP-RNC complex is then targeted to the cytoplasmic membrane where it interacts with the SRP receptor FtsY | - | TS_1828 |
| OG0542 | COG0542,COG1761 | K,O | ATP-dependent CLP protease ATP-binding subunit,DNA-dependent RNA polymerase catalyzes the transcription of DNA into RNA using the four ribonucleoside triphosphates as substrates | FS_2304 | TS_1829 |
| OG0543 | COG0543,COG2890 | C,J | Methylates the class 1 translation termination release factors RF1 PrfA and RF2 PrfB on the glutamine residue of the universally conserved GGQ motif (By similarity),Responsible for channeling the electrons from the oxidation of dihydroorotate from the FMN redox center in the PyrD type B subunit to the ultimate electron acceptor NAD (By similarity) | FS_2305 | TS_1830 |
| OG0544 | NOG11588,NOG11925 | R,S | Capsid scaffolding protein,RNA ligase | FS_2306 | - |
| OG0545 | COG0545,COG2453 | O,T | Dual specificity protein phosphatase,Peptidyl-prolyl cis-trans isomerase | FS_2307 | TS_1832 |
| OG0546 | COG0546,COG1358 | J,R | Superfamily hydrolase, subfamily ia, variant,(ribosomal) protein | FS_0964,FS_1130 | TS_0203 |
| OG0547 | COG0520,COG0547 | E | Anthranilate phosphoribosyltransferase,cysteine desulfurase | FS_2017,FS_2311,FS_2724 | TS_0021,TS_0023,TS_0998, TS_1836 |
| OG0548 | COG0548,COG1145, NOG05480 | C,E,R | 4Fe-4S ferredoxin, iron-sulfur binding,methyltransferaseg kinase | FS_0315,FS_0552,FS_2308 | TS_1303,TS_1834,TS_2184 |
| OG0549 | COG0549,COG2053 | E,J | Ribosomal protein,carbamate kinase | FS_0965 | TS_2022 |
| OG0550 | COG0550,COG1234 | L,R | Releases the supercoiling and torsional tension of D which is introduced during the DNA replication and transcription, by transiently cleaving and rejoining one strand of the DNA duplex. Introduces a single-strand break via transesterification at a target site in duplex DNA. The scissile phosphodiester is attacked by the catalytic tyrosine of the enzyme, resulting in the formation of a DNA-(5’-phosphotyrosyl)-enzyme intermediate and the expulsion of a 3’-OH DNA strand. | - | TS_1880 |
| OG0551 | COG0551,COG1931 | L,S | Releases the supercoiling and torsional tension of D which is introduced during the DNA replication and transcription, by transiently cleaving and rejoining one strand of the DNA duplex. Introduces a single-strand break via transesterification at a target site in duplex DNA. The scissile phosphodiester is attacked by the catalytic tyrosine of the enzyme, resulting in the formation of a DNA-(5’-phosphotyrosyl)-enzyme intermediate and the expulsion of a 3’-OH DNA strand. The free DNA strand then undergoes passage around the unbroken strand, thus removing DNA supercoils. Finally, in the religation step, the DNA 3’-OH attacks the covalent intermediate to expel the active-site tyrosine and restore the DNA phosphodiester backbone (By similarity),UPF0201 protein | - | TS_1878 |
| OG0552 | COG0237,COG0552 | H,U | Catalyzes the phosphorylation of the 3’-hydroxyl group of dephosphocoenzyme A to form coenzyme A (By similarity),Involved in targeting and insertion of nascent membrane proteins into the cytoplasmic membrane. Acts as a receptor for the complex formed by the signal recognition particle (SRP) and the ribosome-nascent chain (RNC) | - | TS_1877 |
| OG0553 | COG0553,COG1514 | J,L | 2’-5’ rna ligase,helicase | FS_0253 | TS_1875 |
| OG0554 | COG1746 | J | Catalyzes the addition and repair of the essential 3’- terminal CCA sequence in tRNAs without using a nucleic acid template. Adds these three nucleotides in the order of C, C, and A to the Trna nucleotide-73, using CTP and ATP as substrates and producing inorganic pyrophosphate (By similarity) | FS_0252 | TS_1874 |
| OG0555 | COG2112 | T | Serine threonine protein kinase | FS_0250 | TS_1873 |
| OG0556 | COG0556,COG4221,NOG05569 | L,R,S | Damaged site, the DNA wraps around one UvrB monomer. DNA wrap is dependent on ATP binding by UvrB and probably causes local melting of the DNA helix, facilitating insertion of UvrB beta-hairpin between the DNA strands. Then UvrB probes one DNA strand for the presence of a lesion. If a lesion is found the UvrA subunits dissociate and the UvrB-DNA preincision complex is formed. This complex is subsequently bound by UvrC and the second UvrB is released. If no lesion is found, the DNA wraps around the other UvrB subunit that will check the other stand for damage (By similarity),PIWI domain protein,dehydrogenase | FS_0399 | TS_1865 |
| OG0557 | COG0105 | F | Major role in the synthesis of nucleoside triphosphates other than ATP. The ATP gamma phosphate is transferred to the NDP beta phosphate via a ping-pong mechanism, using a phosphorylated active-site intermediate (By similarity) | FS_0967 | TS_2020 |
| OG0558 | COG0558,COG1711 | I,S | CDP-diacylglycerol-glycerol-3-phosphate 3-phosphatidyltransferase | FS_0396 | TS_1863 |
| OG0559 | COG0174 | E | Glutamine synthetase | FS_0395,FS_2565 | TS_1862 |
| OG0560 | COG0112,COG0560,NOG05600 | E,S | Catalyzes the reversible interconversion of serine and glycine with tetrahydrofolate (THF) serving as the one-carbon carrier. This reaction serves as the major source of one-carbon groups required for the biosynthesis of purines, thymidylate, methionine, and other important biomolecules. Also exhibits THF- independent aldolase activity toward beta-hydroxyamino acids, producing glycine and aldehydes, via a retro-aldol mechanism (By similarity),phosphoserine phosphatase | FS_2572 | TS_1859 |
| OG0561 | COG0561,COG1646 | R | Prenyltransferase that catalyzes the transfer of the geranylgeranyl moiety of geranylgeranyl diphosphate (GGPP) to the C3 hydroxyl of sn-glycerol-1-phosphate (G1P). This reaction is the first ether-bond-formation step in the biosynthesis of archaeal membrane lipids (By similarity) | FS_0268 | TS_1857 |
| OG0562 | COG2199,COG5594 | S,T | DUF221 domain protein,Diguanylate cyclase | FS_0126,FS_1236 | TS_2150 |
| OG0563 | COG0640,NOG31363 | K | Transcriptional regulator, arsr family,Transcriptional regulator | - | - |
| OG0564 | COG0532 | J | One of the essential components for the initiation of protein synthesis. Protects formylmethionyl-Trna from spontaneous hydrolysis and promotes its binding to the 30S ribosomal subunits. Also involved in the hydrolysis of GTP during the formation of the 70S ribosomal complex. | FS_0968 | TS_2019 |
| OG0565 | NOG63585 | S |  | FS_0264 | TS_1854 |
| OG0566 | COG1474 | LO | Involved in regulation of DNA replication (By similarity) | FS_0262 | TS_1424 |
| OG0567 | COG0156,COG1311 | H,L | 8-Amino-7-oxononanoate synthase,DNA polymerase | FS_0379 | TS_1852 |
| OG0568 | COG2241 | H | Precorrin-6y C5,15-methyltransferase | FS_0801 | TS_1848 |
| OG0569 | COG0526,NOG05691 | O,U | Nephrosis 1, congenital, Finnish type (nephrin),Thioredoxin | FS_0969 | TS_2018 |
| OG0570 | COG2125 | J | 40s ribosomal protein s6 | FS_0494 | TS_1471 |
| OG0571 | COG1371,NOG05713 | S | Chaperone or modulator of proteins involved in DNA or RNA processing (By similarity),domain protein | FS_0970 | TS_2017 |
| OG0574 | COG0574,COG0586 | G,S | SNARE associated Golgi,pyruvate phosphate dikinase | FS_0199 | TS_0145,TS_0524,TS_0528, TS_0530 |
| OG0575 | COG0124,NOG05759 | J,R | Histidyl-trna synthetase,methyltransferase | FS_0971 | TS_2016 |
| OG0576 | COG0576,COG1976 | J,O | Participates actively in the response to hyperosmotic and heat shock by preventing the aggregation of stress-denatured proteins, in association with DnaK and GrpE. It is the nucleotide exchange factor for DnaK and may function as a thermosensor. Unfolded proteins bind initially to DnaJ,Binds to the 60S ribosomal subunit and prevents its association with the 40S ribosomal subunit to form the 80S initiation complex in the cytoplasm | FS_0973 | TS_2015 |
| OG0577 | COG0577,COG5257 | J,V | ABC transporter (permease),translation initiation factor | FS_0493 | TS_1470 |
| OG0578 | COG0470 | L | DNA polymerase III subunit delta’ | FS_0977 | TS_1775 |
| OG0579 | COG0579,NOG05794,NOG107639 | C,S,T | GINS complex protein,calcium calmodulin-dependent protein kinase,malate dehydrogenase (quinone) | - | - |
| OG0580 | COG0580,COG1241 | G,L | Channel that permits osmotically driven movement of water in both directions. It is involved in the osmoregulation and in the maintenance of cell turgor during volume expansion in rapidly growing cells. It mediates rapid entry or exit of water in response to abrupt changes in osmolarity (By similarity),dna replication licensing factor | FS_0980 | TS_1773 |
| OG0581 | COG0472,COG0581 | M,P | First step of the lipid cycle reactions in the biosynthesis of the cell wall peptidoglycan (By similarity),phosphate abc transporter | FS_0982 | TS_1770 |
| OG0582 | COG0110,COG0582 | L,R | Integrase,acetyltransferase | FS_0984 | TS_1767 |
| OG0583 | COG0583,COG2835 | K,S | Transcriptional regulator,UPF0434 protein | FS_0985 | TS_1766 |
| OG0584 | COG0115 | E | brancheD-chain amino acid aminotransferase | FS_0988 | TS_1763 |
| OG0585 | COG0119,COG0451,COG0585,COG1056,COG1057 | E,H,M,S | Catalyzes the condensation of the acetyl group of acetyl-CoA with 3-methyl-2-oxobutanoate (2-oxoisovalerate) to form 3-carboxy-3-hydroxy-4-methylpentanoate (2-isopropylmalate) (By similarity),Catalyzes the reversible adenylation of nicotinate mononucleotide (NaMN) to nicotinic acid adenine dinucleotide (NaAD) (By similarity)d-dependent epimerase dehydratase,Nicotinamide-nucleotide adenylyltransferase,Responsible for synthesis of pseudouridine from uracil- 13 in transfer RNAs (By similarity) | FS_0989 | TS_1762 |
| OG0586 | COG0586,COG1018 | C,S | Is involved in NO detoxification in an aerobic process, termed nitric oxide dioxygenase (NOD) reaction that utilizes O(2) and NAD(P)H to convert NO to nitrate, which protects the bacterium from various noxious nitrogen compounds. Therefore, plays a central role in the inducible response to nitrosative stress (By similarity),SNARE associated Golgi | FS_0990 | TS_1760 |
| OG0587 | COG2513 | G | Methylisocitrate lyase | FS_0992 | TS_1757 |
| OG0588 | COG1053 | C | Succinate DeHydrogenase | FS_0993 | TS_1755 |
| OG0589 | COG0589,NOG118664 | C,T | Succinate dehydrogenase/Fumarate reductase transmembrane subunit,Universal stress protein | FS_0994 | TS_1754 |
| OG0590 | COG2009 | C | Succinate dehydrogenase, cytochrome | FS_0995 | TS_1753 |
| OG0591 | COG0479,COG0591 | C,E | Succinate dehydrogenase,symporter | FS_0996 | TS_1752 |
| OG0592 | COG0592,COG2151,NOG05922 | L,S | DNA polymerase III is a complex, multichain enzyme responsible for most of the replicative synthesis in bacteria. This DNA polymerase also exhibits 3’ to 5’ exonuclease activity. The beta chain is required for initiation of replication once it is clamped onto D it slides freely (bidirectional and ATP- independent) along duplex DNA (By similarity),alpha-L-rhamnosidase,fes assembly suf system protein | FS_0997 | TS_1751 |
| OG0593 | COG0165,COG0593,NOG05936 | E,L,R | Actin binding LIM protein,argininosuccinate lyase,it binds specifically double-stranded DNA at a 9 bp consensus (dnaA box) 5’-TTATC CA A CA A-3’. DnaA binds to ATP and to acidic phospholipids (By similarity) | FS_0998 | TS_1750 |
| OG0594 | COG0483 | G | Inositol monophosphatase | FS_1753 | TS_1741 |
| OG0595 | COG0595,COG3937 | R,S | Metallo-Beta-Lactamase | FS_1752 | TS_1740 |
| OG0596 | COG0470 | L | DNA polymerase III subunit delta’ | FS_1751 | TS_1739 |
| OG0597 | COG0597,NOG170330,NOG18603 | MU,R,S | MIT (microtubule interacting and transport) domain,This protein specifically catalyzes the removal of signal peptides from prolipoproteins. | - | - |
| OG0598 | COG0439 | I | Acetyl-CoA carboxylase biotin carboxylase | FS_1745 | TS_1735 |
| OG0599 | COG0599,NOG102165 | S | Antioxidant protein with alkyl hydroperoxidase activity. Required for the reduction of the AhpC active site cysteine residues and for the regeneration of the AhpC enzyme activity (By similarity),Biotin lipoyl attachment | FS_1744 | TS_1734 |
| OG0600 | COG0600,COG1225 | O,P | Binding-protein-dependent transport systems inner membrane component,alkyl hydroperoxide reductase Thiol specific antioxidant Mal allergen | FS_1742 | TS_1733 |
| OG0601 | COG0601,COG0838,NOG06013,NOG06019 | C,O,P,R | NDH-1 shuttles electrons from NADH, via FMN and iron- sulfur (Fe-S) centers, to quinones in the respiratory chain,ABC transporter (permease),Calreticulin | FS_1741 | TS_1732 |
| OG0602 | COG0377,COG0602 | C,O | Catalyzes the conversion of 6-carboxy-5,6,7,8- tetrahydropterin (CPH4) to 7-carboxy-7-deazaguanine (CDG) (By similarity),NDH-1 shuttles electrons from NADH, via FMN and iron- sulfur (Fe-S) centers, to quinones in the respiratory chain. The immediate electron acceptor for the enzyme in this species is believed to be ubiquinone. Couples the redox reaction to proton translocation (for every two electrons transferred, four hydrogen ions are translocated across the cytoplasmic membrane), and thus conserves the redox energy in a proton gradient. | FS_1740 | TS_1731 |
| OG0603 | COG0603,COG0852 | C,R | NDH-1 shuttles electrons from NADH, via FMN and iron- sulfur (Fe-S) centers, to quinones in the respiratory chain. The immediate electron acceptor for the enzyme in this species is believed to be ubiquinone. Couples the redox reaction to proton translocation (for every two electrons transferred, four hydrogen ions are translocated across the cytoplasmic membrane), and thus conserves the redox energy in a proton gradient (By similarity),Catalyzes the ATP-dependent conversion of 7-carboxy-7- deazaguanine (CDG) to 7-cyano-7-deazaguanine (preQ(0)) (By similarity) | FS_1739 | TS_1730 |
| OG0604 | COG0604,COG1005 | C | NDH-1 shuttles electrons from NADH, via FMN and iron- sulfur (Fe-S) centers, to quinones in the respiratory chain. The immediate electron acceptor for the enzyme in this species is believed to be ubiquinone. Couples the redox reaction to proton translocation (for every two electrons transferred, four hydrogen ions are translocated across the cytoplasmic membrane), and thus conserves the redox energy in a proton gradient. This subunit may bind ubiquinone (By similarity),alcohol dehydrogenase | FS_1737 | TS_1728 |
| OG0605 | COG0605,COG1143 | C,P | NDH-1 shuttles electrons from NADH, via FMN and iron- sulfur (Fe-S) centers, to quinones in the respiratory chain. The immediate electron acceptor for the enzyme in this species is believed to be ubiquinone. Couples the redox reaction to proton translocation (for every two electrons transferred, four hydrogen ions are translocated across the cytoplasmic membrane), and thus conserves the redox energy in a proton gradient (By similarity),Destroys radicals which are normally produced within the cells and which are toxic to biological systems (By similarity) | FS_1735 | TS_1727 |
| OG0606 | COG0839 | C | NADH dehydrogenase subunit j | FS_1733 | TS_1726 |
| OG0607 | COG0607,COG0713 | C,P | NDH-1 shuttles electrons from NADH, via FMN and iron- sulfur (Fe-S) centers, to quinones in the respiratory chain. The immediate electron acceptor for the enzyme in this species is believed to be ubiquinone. Couples the redox reaction to proton translocation (for every two electrons transferred, four hydrogen ions are translocated across the cytoplasmic membrane), and thus conserves the redox energy in a proton gradient (By similarity),Rhodanese domain protein | FS_1732 | TS_1725 |
| OG0608 | COG0608,COG0651 | CP,L | Monovalent cation H antiporter subunit D,Single-stranded-DNA-specific exonuclease (RecJ) | FS_1730 | TS_1723 |
| OG0609 | COG1009 | C | subunit l | FS_0001,FS_1729 | TS_1722 |
| OG0610 | COG0610,COG1007 | C,V | NDH-1 shuttles electrons from NADH, via FMN and iron- sulfur (Fe-S) centers, to quinones in the respiratory chain. The immediate electron acceptor for the enzyme in this species is believed to be ubiquinone. Couples the redox reaction to proton translocation (for every two electrons transferred, four hydrogen ions are translocated across the cytoplasmic membrane), and thus conserves the redox energy in a proton gradient (By similarity),Type I site-specific deoxyribonuclease | FS_0002 | TS_1720 |
| OG0611 | COG0142,COG0611 | H | Catalyzes the ATP-dependent phosphorylation of thiamine- monophosphate (TMP) to form thiamine-pyrophosphate (TPP), the active form of vitamin B1 (By similarity),synthase | FS_0003 | TS_1717 |
| OG0612 | COG5424 | H | Ring cyclization and eight-electron oxidation of 3a-(2- amino-2-carboxyethyl)-4,5-dioxo-4,5,6,7,8,9-hexahydroquinoline- 7,9-dicarboxylic-acid to PQQ (By similarity) | FS_0004 | TS_1716 |
| OG0613 | COG0461,COG0613 | F,S | Catalyzes the transfer of a ribosyl phosphate group from 5-phosphoribose 1-diphosphate to orotate, leading to the formation of orotidine monophosphate (OMP) (By similarity),PHP domain protein | FS_0006 | TS_1714 |
| OG0614 | COG3875 | S | Domain of unknown function (DUF2088) | FS_0007 | TS_1712 |
| OG0615 | COG0456,COG0615 | IM,R | cytidylyltransferase | FS_0009 | TS_1710 |
| OG0616 | COG0249,NOG118207 | L,O | DNAj domain protein,that it carries out the mismatch recognition step. This protein has a weak ATPase activity (By similarity) | FS_2120 | TS_1704 |
| OG0617 | COG0036,COG0617,COG3959 | G,J | Catalyzes the addition and repair of the essential 3’- terminal CCA sequence in tRNAs without using a nucleic acid template. Adds these three nucleotides in the order of C, C, and A to the Trna nucleotide-73, using CTP and ATP as substrates and producing inorganic pyrophosphate,Transketolase,ribulose-phosphate 3-epimerase | FS_0016 | TS_1702 |
| OG0618 | COG0176 | G | Transaldolase is important for the balance of metabolites in the pentose-phosphate pathway (By similarity) | FS_0018 | TS_1697 |
| OG0619 | COG0619,COG1698, NOG06197 | P,S | Ragb susd domain-containing protein,Transmembrane (T) component of an energy-coupling factor (ECF) ABC-transporter complex. Unlike classic ABC transporters this ECF transporter provides the energy necessary to transport a number of different substrates (By similarity),UPF0147 protein | FS_0328 | TS_0149,TS_0306,TS_0418, TS_1312 |
| OG0620 | COG0179 | Q | Fumarylacetoacetate hydrolase | FS_0330 | - |
| OG0621 | COG0008,COG0621 | J | Catalyzes the methylthiolation of N6- (dimethylallyl)adenosine (i(6)A), leading to the formation of 2- methylthio-N6-(dimethylallyl)adenosine (ms(2)i(6)A) at position 37 in tRNAs that read codons beginning with uridine (By similarity),Catalyzes the attachment of glutamate to Trna(Glu) in a two-step reaction glutamate is first activated by ATP to form Glu-AMP and then transferred to the acceptor end of Trna(Glu) (By similarity) | FS_0331 | - |
| OG0622 | COG0142 | H | Synthase | FS_0332 | - |
| OG0623 | COG1608 | R | Aspartate glutamate uridylate kinase | FS_0334 | TS_2309 |
| OG0624 | COG0624,COG1577 | E,I | Catalyzes the hydrolysis of N-succinyl-L,L- diaminopimelic acid (SDAP), forming succinate and LL-2,6- diaminoheptanedioate (DAP), an intermediate involved in the bacterial biosynthesis of lysine and meso-diaminopimelic acid, an essential component of bacterial cell walls (By similarity),mevalonate kinase | FS_0335 | TS_2307 |
| OG0625 | COG0052 | J | 30S ribosomal protein S2 | FS_0337 | TS_2305 |
| OG0626 | COG0148,COG0626 | E,G | Catalyzes the reversible conversion of 2- phosphoglycerate into phosphoenolpyruvate. It is essential for the degradation of carbohydrates via glycolysis (By similarity),cystathionine | FS_0338 | TS_2304 |
| OG0627 | COG1644 | K | DNA-dependent RNA polymerase catalyzes the transcription of DNA into RNA using the four ribonucleoside triphosphates as substrates | FS_0339 | TS_2303 |
| OG0628 | COG0062 | G | Catalyzes the epimerization of the S- and R-forms of NAD(P)HX, a damaged form of NAD(P)H that is a result of enzymatic or heat-dependent hydration. This is a prerequisite for the S- specific NAD(P)H-hydrate dehydratase to allow the repair of both epimers of NAD(P)HX (By similarity) | FS_0340 | TS_2302 |
| OG0629 | COG0629,COG2038 | H,L | Catalyzes the synthesis of alpha-ribazole-5’-phosphate from nicotinate mononucleotide (NAMN) and 5,6- dimethylbenzimidazole (DMB) (By similarity),single-stranded DNA-binding protein | FS_0342 | TS_2300 |
| OG0630 | COG0522,COG0630 | J,U | One of the primary Rrna binding proteins, it binds directly to 16S Rrna where it nucleates assembly of the body of the 30S subunit (By similarity),type II secretion system protein | FS_0344 | TS_2298 |
| OG0631 | COG0099,COG0631 | J,T | Located at the top of the head of the 30S subunit, it contacts several helices of the 16S Rrna. In the 70S ribosome it contacts the 23S Rrna (bridge B1a) and protein L5 of the 50S subunit (bridge B1b), connecting the 2 subunits,Phosphatase | FS_0345 | TS_2297 |
| OG0633 | COG2138,NOG06330 | H,U | 5-Hydroxytryptamine (Serotonin) receptor,cobalamin (vitamin b12) biosynthesis cbix protein | FS_2024 | TS_1458 |
| OG0634 | COG0503,COG2247,NOG16463 | F,M,S | Catalyzes a salvage reaction resulting in the formation of AMP, that is energically less costly than de novo synthesis (By similarity),Cell wall binding repeat 2-containing protein,Ubiquitin-protein ligase | FS_0367 | - |
| OG0635 | COG0635,COG1032 | C,H | Coproporphyrinogen III oxidase,radical SAM domain protein | FS_0359,FS_0368 | TS_1536 |
| OG0636 | COG1691 | S | 1-(5-Phosphoribosyl)-5-amino-4-imidazole- carboxylate (AiR) carboxylase | FS_0357 | - |
| OG0637 | COG0039 | C | Catalyzes the reversible oxidation of malate to oxaloacetate (By similarity) | FS_0356 | - |
| OG0638 | COG0638,COG1641 | O,S | The proteasome is a multicatalytic proteinase complex which is characterized by its ability to cleave peptides with Arg, Phe, Tyr, Leu, and Glu adjacent to the leaving group at neutral or slightly basic Ph. The proteasome has an ATP-dependent proteolytic activity (By similarity),UPF0272 protein | FS_0354,FS_0632,FS_0717, FS_1060 | TS_0366,TS_0457,TS_0666, TS_1201 |
| OG0639 | COG0639,COG1606 | R,T | pp-loop domain protein,serine threonine-protein phosphatase | FS_0353 | - |
| OG0640 | COG0640,COG2012 | K | Transcriptional regulator, arsr family,DNA-dependent RNA polymerase catalyzes the transcription of DNA into RNA using the four ribonucleoside triphosphates as substrates | FS_0226,FS_0812,FS_1619, FS_1969,FS_2351,FS_2397, FS_2550 | TS_0026,TS_0657,TS_0664, TS_1447,TS_2055 |
| OG0641 | COG0086 | K | DNA-dependent RNA polymerase catalyzes the transcription of DNA into RNA using the four ribonucleoside triphosphates as substrates (By similarity) | FS_0814 | - |
| OG0642 | COG0642,COG1911 | J,T | (Ribosomal) protein,Histidine kinase | FS_0815 | - |
| OG0643 | COG0195,COG0643 | K,T | CheA signal transduction histidine kinase,Transcription elongation factor NusA | FS_0816 | - |
| OG0644 | COG0048,COG0644 | C,J | Interacts with and stabilizes bases of the 16S Rrna that are involved in Trna selection in the A site and with the Mrna backbone. Located at the interface of the 30S and 50S subunits, it traverses the body of the 30S subunit contacting proteins on the other side and probably holding the Rrna structure together. The combined cluster of proteins S8, S12 and S17 appears to hold together the shoulder and platform of the 30S subunit (By similarity),geranylgeranyl reductase | FS_0817 | - |
| OG0645 | COG0049,COG0645 | J,S | One of the primary Rrna binding proteins, it binds directly to 16S Rrna where it nucleates assembly of the head domain of the 30S subunit. Is located at the subunit interface close to the decoding center, probably blocks exit of the E-site Trna (By similarity),Aminoglycoside phosphotransferase | FS_0818 | - |
| OG0646 | COG0646,COG0827,COG1802,COG3903 | E,K,L | DNA Methylase,GntR Family Transcriptional Regulator,methionine synthase,transcriptional regulator | FS_0819 | - |
| OG0647 | COG0647,COG2072,NOG31616 | G,P,S | Hydrolase,Monooxygenase, Pfam:UPF0153 | FS_0820 | - |
| OG0648 | COG0644,COG0648 | C,L | Endonuclease IV plays a role in DNA repair. It cleaves phosphodiester bonds at apurinic or apyrimidinic sites (AP sites) to produce new 5’-ends that are base-free deoxyribose 5-phosphate residues. It preferentially attacks modified AP sites created by bleomycin and neocarzinostatin (By similarity),geranylgeranyl reductase | FS_0821 | TS_1889 |
| OG0649 | COG0649,COG1958 | C,K | NDH-1 shuttles electrons from NADH, via FMN and iron- sulfur (Fe-S) centers, to quinones in the respiratory chain. The immediate electron acceptor for the enzyme in this species is believed to be ubiquinone. Couples the redox reaction to proton translocation (for every two electrons transferred, four hydrogen ions are translocated across the cytoplasmic membrane), and thus conserves the redox energy in a proton gradient (By similarity),small nuclear ribonucleoprotein | FS_0060,FS_0959,FS_1386, FS_1738,FS_1740 | TS_1451,TS_1729,TS_1731, TS_2028 |
| OG0651 | COG0651,COG1201 | CP,L | Monovalent cation H antiporter subunit D,Dead DEAH box helicase | FS_0062,FS_0065,FS_0609, FS_1438,FS_1730 | TS_1449,TS_1719,TS_1723 |
| OG0652 | COG0652,COG1599 | L,O | PPIases accelerate the folding of proteins,replication | - | TS_1445 |
| OG0653 | NOG09900 | R | (ABC) transporter | FS_2210 | TS_1444 |
| OG0654 | COG0605 | P | Destroys radicals which are normally produced within the cells and which are toxic to biological systems (By similarity) | FS_0847 | TS_1913,TS_2103 |
| OG0655 | COG0655,NOG06555,NOG103385 | O,R,S | Molecular chaperone capable of stabilizing a range of proteins. Seems to fulfill an ATP-independent, HSP70-like function in archaeal de novo protein folding (By similarity)dph-dependent fmn reductase,integral membrane protein | FS_0848 | TS_1916 |
| OG0656 | COG0098,COG0656 | J,R | Located at the back of the 30S subunit body where it stabilizes the conformation of the head with respect to the body (By similarity),reductase | FS_2486 | TS_1922 |
| OG0657 | COG1841 | J | 50S ribosomal protein L30 | FS_2487 | TS_1923 |
| OG0658 | COG0200 | J | Binds to the 23S Rrna (By similarity) | FS_2488 | TS_1924 |
| OG0659 | COG0201 | U | The central subunit of the protein translocation channel SecYEG. Consists of two halves formed by TMs 1-5 and 6-10. These two domains form a lateral gate at the front which open onto the bilayer between TMs 2 and 7, and are clamped together by SecE at the back. The channel is closed by both a pore ring composed of hydrophobic SecY resides and a short helix (helix 2A) on the extracellular side of the membrane which forms a plug. The plug probably moves laterally to allow the channel to open. The ring and the pore may move independently (By similarity) | FS_2489 | TS_1925 |
| OG0660 | COG0842 | V | (ABC) transporter | FS_2209 | TS_1443 |
| OG0661 | COG0661,COG1102 | F,R | Required, probably indirectly, for the hydroxylation of 2-octaprenylphenol to 2-octaprenyl-6-hydroxy-phenol, the fourth step in ubiquinone biosynthesis (By similarity),Cytidine monophosphate kinase | FS_1757 | TS_1929 |
| OG0662 | COG0130,NOG06623 | J,S | (LipO)protein,Responsible for synthesis of pseudouridine from uracil- 55 in the psi GC loop of transfer RNAs (By similarity) | FS_1758 | TS_1930 |
| OG0663 | COG0337,COG0417,COG0663 | E,L,R | 3-Dehydroquinate synthase,DNA polymerase,Transferase | FS_1772 | - |
| OG0664 | COG0664,COG1052 | C,T | Transcriptional regulator, crp fnr family,Dehydrogenase | FS_1773,FS_2231 | TS_1936 |
| OG0665 | COG0665,COG0674,COG1014 | C,E | Catalyzes the last two steps in the biosynthesis of 5- methylaminomethyl-2-thiouridine (mnm(5)s(2)U) at the wobble position (U34) in Trna. Catalyzes the FAD-dependent demodification of cmnm(5)s(2)U34 to nm(5)s(2)U34, followed by the transfer of a methyl group from S-adenosyl-L-methionine to nm(5)s(2)U34, to form mnm(5)s(2)U34 (By similarity),Oxidoreductase required for the transfer of electrons from pyruvate to flavodoxin (By similarity),oxidoreductase | FS_1775,FS_1777 | TS_1938 |
| OG0666 | COG0666,COG1013 | C,R | Ankyrin Repeat,Oxidoreductase required for the transfer of electrons from pyruvate to flavodoxin (By similarity) | FS_1780 | TS_1940 |
| OG0667 | COG0394,COG0667 | C,T | Aldo keto reductase,PHOsphatase | FS_2730 | TS_1846 |
| OG0668 | COG0668,NOG98311 | M,S | Mechanosensitive ion channel | FS_1743 | - |
| OG0669 | COG0066 | E | Catalyzes the isomerization between 2-isopropylmalate and 3-isopropylmalate, via the formation of 2-isopropylmaleate (By similarity) | FS_2010 | TS_1948 |
| OG0670 | COG0065 | E | Catalyzes the isomerization between 2-isopropylmalate and 3-isopropylmalate, via the formation of 2-isopropylmaleate (By similarity) | FS_2007 | TS_1949 |
| OG0671 | COG0671,COG1817 | I,S | PHOsphatase,Protein of unknown function (DUF354) | FS_1139 | - |
| OG0672 | COG0066,NOG06726 | E,S | Catalyzes the isomerization between 2-isopropylmalate and 3-isopropylmalate, via the formation of 2-isopropylmaleate (By similarity),transmembrane and coiled-coil domains 7 | FS_2006 | TS_1950 |
| OG0673 | COG0103,COG0673,NOG06733 | C,J,R | 30S ribosomal protein S9,alcohol dehydrogenase,oxidoreductase | FS_2003 | TS_1953 |
| OG0674 | COG0102,COG0674 | C,J | This protein is one of the early assembly proteins of the 50S ribosomal subunit, although it is not seen to bind Rrna by itself. It is important during the early stages of 50S assembly (By similarity),Oxidoreductase required for the transfer of electrons from pyruvate to flavodoxin (By similarity) | FS_2002 | TS_1954 |
| OG0675 | COG0675,COG1727 | J,L | Ribosomal protein,Transposase | FS_2000 | TS_1955 |
| OG0676 | COG0202 | K | DNA-dependent RNA polymerase catalyzes the transcription of DNA into RNA using the four ribonucleoside triphosphates as substrates (By similarity) | FS_1999 | TS_1956 |
| OG0677 | COG0677,COG1097 | J,M | Dehydrogenase,Exosome complex | FS_1997 | TS_1958 |
| OG0678 | COG0689 | J | Phosphorolytic exoribonuclease that removes nucleotide residues following the —CCA terminus of Trna and adds nucleotides to the ends of RNA molecules by using nucleoside diphosphates as substrates (By similarity) | FS_1996 | TS_1960 |
| OG0679 | COG0677,COG0771,COG3127 | M,Q | ABC transporter (permease),Cell wall formation. Catalyzes the addition of glutamate to the nucleotide precursor UDP-N-acetylmuramoyl-L-alanine (UMA) (By similarity),Dehydrogenase | FS_1140 | - |
| OG0680 | COG2123 | J | Exosome complex | FS_1995 | TS_1961 |
| OG0681 | COG0681,COG1997 | J,U | Ribosomal protein,Signal peptidase i | FS_1994 | TS_1962 |
| OG0682 | COG1382 | O | Molecular chaperone capable of stabilizing a range of proteins. Seems to fulfill an ATP-independent, HSP70-like function in archaeal de novo protein folding (By similarity) | FS_1992 | TS_1964 |
| OG0683 | COG0683,COG1948,NOG06833 | E,L,S | Excision repair cross-complementing rodent repair deficiency, complementation group 4,ABC transporter substrate-binding protein,Retrotransposon protein | FS_1991 | TS_1965 |
| OG0684 | COG0061 | G | Catalyzes the phosphorylation of NAD to NADP. Utilizes ATP and other nucleoside triphosphates as well as inorganic polyphosphate as a source of phosphorus (By similarity) | FS_1508 | TS_1969 |
| OG0685 | COG0576,COG0685 | E,O | Participates actively in the response to hyperosmotic and heat shock by preventing the aggregation of stress-denatured proteins, in association with DnaK and GrpE. It is the nucleotide exchange factor for DnaK and may function as a thermosensor. Unfolded proteins bind initially to DnaJ,Methylenetetrahydrofolate reductase | FS_1141 | - |
| OG0687 | COG0687,COG1197,COG4458,NOG16972 | E,L,S | Virulence,extracellular solute-binding protein family 1,transcriptioN-repair coupling factor | FS_1520 | - |
| OG0688 | COG0197 | J | Binds 23S Rrna and is also seen to make contacts with the A and possibly P site tRNAs (By similarity) | FS_1525 | - |
| OG0689 | COG0689,COG1676 | J | Sites to release the intron. The products are an intron and two Trna half-molecules bearing 2’,3’ cyclic phosphate and 5’-OH termini. Recognizes a pseudosymmetric substrate in which 2 bulged loops of 3 bases are separated by a stem of 4 bp (By similarity),Phosphorolytic exoribonuclease that removes nucleotide residues following the —CCA terminus of Trna and adds nucleotides to the ends of RNA molecules by using nucleoside diphosphates as substrates (By similarity) | FS_1527,FS_1996,FS_2127 | TS_0468,TS_1960 |
| OG0690 | COG0484 | O | ATP binding to DnaK triggers the release of the substrate protein, thus completing the reaction cycle. Several rounds of ATP-dependent interactions between DnaJ, DnaK and GrpE are required for fully efficient folding. Also involved, together with DnaK and GrpE, in the DNA replication of plasmids through activation of initiation proteins (By similarity) | FS_1143 | TS_1454 |
| OG0691 | COG0102,COG1249,COG3711,NOG00891 | C,J,K,S | This protein is one of the early assembly proteins of the 50S ribosomal subunit, although it is not seen to bind Rrna by itself. It is important during the early stages of 50S assembly (By similarity),dihydrolipoyl dehydrogenase,transcriptional antiterminator | - | TS_1019 |
| OG0692 | COG0495,COG5043,NOG17565 | J,T,U | Anti-FecI sigma factor, FecR,Leucyl-trna synthetase,Vacuolar Protein | FS_0822 | TS_1890 |
| OG0694 | COG0520 | E | cysteine desulfurase | FS_2017 | TS_0997 |
| OG0695 | COG0822 | C | Suf system Fes assembly protein, NifU family | FS_2016 | TS_0996 |
| OG0696 | COG0126,COG0696,NOG06965 | G,T | Catalyzes the interconversion of 2-phosphoglycerate and 3-phosphoglycerate (By similarity),Serine Threonine protein kinase,phosphoglycerate kinase | - | - |
| OG0697 | COG0090,COG0697 | EG,J | One of the primary Rrna binding proteins. Required for association of the 30S and 50S subunits to form the 70S ribosome, for Trna binding and peptide bond formation. It has been suggested to have peptidyltransferase activity,membrane | FS_2250 | - |
| OG0698 | COG0373 | H | Catalyzes the NADPH-dependent reduction of glutamyl- Trna(Glu) to glutamate 1-semialdehyde (GSA) (By similarity) | FS_1417 | TS_0976 |
| OG0699 | NOG12559 | K | Transcriptional regulator AsnC family | FS_1415 | TS_0979 |
| OG0700 | COG0396 | O | FeS assembly ATPase SufC | FS_1427 | TS_0964 |
| OG0701 | COG1405 | K | Stabilizes TBP binding to an archaeal box-A promoter. Also responsible for recruiting RNA polymerase II to the pre- initiation complex (DNA-TBP-TFIIB) (By similarity) | FS_0636,FS_2415 | TS_0963 |
| OG0702 | COG0702,COG3277 | GM,J | Required for ribosome biogenesis. Part of a complex which catalyzes pseudouridylation of Rrna. This involves the isomerization of uridine such that the ribose is subsequently attached to C5, instead of the normal N1. Pseudouridine ( psi ) residues may serve to stabilize the conformation of rRNAs,epimerase dehydratase | FS_0637 | TS_0962 |
| OG0703 | COG1400 | U | Involved in targeting and insertion of nascent membrane proteins into the cytoplasmic membrane. Binds directly to 7S RNA and mediates binding of the 54 kDa subunit of the SRP (By similarity) | FS_0638 | TS_0961 |
| OG0704 | COG0704,COG2007 | J,P | 40S ribosomal protein S8,Plays a role in the regulation of phosphate uptake | FS_0639 | TS_0960 |
| OG0705 | COG0705,COG1386 | K,R | Participates in chromosomal partition during cell division. May act via the formation of a condensin-like complex containing Smc and ScpA that pull DNA away from mid-cell into both cell halves (By similarity),rhomboid family | FS_0640 | TS_0959 |
| OG0706 | COG0706,COG1354 | S,U | Required for the insertion and or proper folding and or complex formation of integral membrane proteins into the membrane. Involved in integration of membrane proteins that insert both dependently and independently of the Sec translocase complex, as well as at least some lipoproteins,Participates in chromosomal partition during cell division. May act via the formation of a condensin-like complex containing Smc and ScpB that pull DNA away from mid-cell into both cell halves (By similarity) | FS_0641 | TS_0957 |
| OG0707 | COG0231,COG0707 | J,M | Involved in peptide bond synthesis. Stimulates efficient translation and peptide-bond synthesis on native or reconstituted 70S ribosomes in vitro. Probably functions indirectly by altering the affinity of the ribosome for aminoacyl-Tr thus increasing their reactivity as acceptors for peptidyl transferase (By similarity),Cell wall formation. Catalyzes the transfer of a GlcNAc subunit on undecaprenyl-pyrophosphoryl-MurNAc-pentapeptide (lipid intermediate I) to form undecaprenyl-pyrophosphoryl-MurNAc- (pentapeptide)GlcNAc (lipid intermediate II) (By similarity) | FS_0645 | TS_0953 |
| OG0708 | COG0024,COG0661,COG1136 | J,R,V | Required, probably indirectly, for the hydroxylation of 2-octaprenylphenol to 2-octaprenyl-6-hydroxy-phenol, the fourth step in ubiquinone biosynthesis (By similarity),(ABC) transporter,Removes the N-terminal methionine from nascent proteins (By similarity) | FS_0651 | TS_0933 |
| OG0709 | COG1998 | J | 30S ribosomal protein S27Ae | FS_0653 | TS_0932 |
| OG0710 | COG0710,COG2004 | E,J | 40S ribosomal protein S24,Type I DHQase | FS_0654 | TS_0931 |
| OG0711 | COG0711,COG1547 | C,S | F_(1)_F_(0)_ ATP synthase produces ATP from ADP in the presence of a proton or sodium gradient. F-type ATPases consist of two structural domains, F_(1)_ containing the extramembraneous catalytic core and F_(0)_ containing the membrane proton channel, linked together by a central stalk and a peripheral stalk. During catalysis, ATP synthesis in the catalytic domain of F_(1)_ is coupled via a rotary mechanism of the central stalk subunits to proton translocation (By similarity),Domain of unknown function (DUF309) | FS_0655 | TS_0929 |
| OG0712 | COG3635 | G | Catalyzes the interconversion of 2-phosphoglycerate and 3-phosphoglycerate (By similarity) | FS_0656 | TS_0928 |
| OG0713 | COG0713,COG1085 | C,G | NDH-1 shuttles electrons from NADH, via FMN and iron- sulfur (Fe-S) centers, to quinones in the respiratory chain. The immediate electron acceptor for the enzyme in this species is believed to be ubiquinone. Couples the redox reaction to proton translocation (for every two electrons transferred, four hydrogen ions are translocated across the cytoplasmic membrane), and thus conserves the redox energy in a proton gradient (By similarity),galactose-1-phosphate uridylyltransferase | FS_0660 | TS_0924 |
| OG0714 | COG0714,COG2409 | R | ATPase associated with various cellular activities,MmpL domain protein | - | TS_0923 |
| OG0715 | COG0715,COG1208 | M,P | (ABC) transporter,nucleotidyl transferase | FS_0542,FS_0663,FS_1270, FS_2004 | TS_0921,TS_1316,TS_1951 |
| OG0716 | COG0287,COG0716 | C,E | Low-potential electron donor to a number of redox enzymes (By similarity),prephenate dehydrogenase | FS_0667 | TS_0915 |
| OG0717 | COG0436 | E | Aminotransferase | FS_0668 | TS_0914 |
| OG0718 | COG0082 | E | 5-Enolpyruvylshikimate-3-phosphate phospholyase | FS_0669 | TS_0913 |
| OG0719 | COG0128,COG0719 | E,O | 5-Enolpyruvylshikimate-3-phosphate synthase,Fe-S assembly protein | FS_0670 | TS_0910 |
| OG0720 | COG0720,COG1685 | E,H | Shikimate kinase,synthase | FS_0671 | TS_0909 |
| OG0721 | COG0710,COG0721 | E,J | Allows the formation of correctly charged Asn-Trna(Asn) or Gln-Trna(Gln) through the transamidation of misacylated Asp- Trna(Asn) or Glu-Trna(Gln) in organisms which lack either or both of asparaginyl-Trna or glutaminyl-Trna synthetases. The reaction takes place in the presence of glutamine and ATP through an activated phospho-Asp-Trna(Asn) or phospho-Glu-Trna(Gln) (By similarity),Type I DHQase | FS_0675 | TS_0904 |
| OG0722 | COG1465,NOG07227 | E,R | Catalyzes the oxidative deamination and cyclization of 2-amino-3,7-dideoxy-D-threo-hept-6-ulosonic acid (ADH) to yield 3- dehydroquinate (DHQ), which is fed into the canonical shikimic pathway of aromatic amino acid biosynthesis (By similarity),methyltransferase like 25 | FS_0681 | TS_0898 |
| OG0723 | COG0723,COG1830 | C,G | Component of the ubiquinol-cytochrome c reductase complex (complex III or cytochrome b-c1 complex), which is a respiratory chain that generates an electrochemical potential coupled to ATP synthesis (By similarity),Aldolase | FS_0683 | TS_0897 |
| OG0724 | COG1060 | H | Catalyzes the radical-mediated transfer of the hydroxybenzyl group from 4-hydroxyphenylpyruvate (HPP) to 5-amino- 6-ribitylamino-2,4(1H,3H)-pyrimidinedione to form 7,8-didemethyl- 8-hydroxy-5-deazariboflavin (FO) (By similarity) | FS_0685 | TS_0895 |
| OG0725 | COG0043,NOG07251 | H,S | Catalyzes the decarboxylation of 3-octaprenyl-4-hydroxy benzoate to 2-octaprenylphenol (By similarity),myomesin 3 | FS_0686 | TS_0894 |
| OG0726 | COG0726,COG1354 | G,S | Participates in chromosomal partition during cell division. May act via the formation of a condensin-like complex containing Smc and ScpB that pull DNA away from mid-cell into both cell halves (By similarity),polysaccharide deacetylase | FS_0689 | TS_0892 |
| OG0727 | COG0727,COG1225 | O,S | Fe-S-cluster oxidoreductase,alkyl hydroperoxide reductase Thiol specific antioxidant Mal allergen | FS_0605 | TS_0889 |
| OG0729 | COG1384 | J | lysyl-Trna synthetase | FS_0423 | TS_0841 |
| OG0730 | COG0730,COG1243 | BK,S | Histone acetyltransferase,Membrane | FS_0426 | TS_0840 |
| OG0731 | COG0150,COG0731 | C,F | Phosphoribosylaminoimidazole synthetase,radical SAM domain protein | FS_0427 | TS_0836 |
| OG0732 | COG0732,COG1060 | H,V | Catalyzes the radical-mediated transfer of the hydroxybenzyl group from 4-hydroxyphenylpyruvate (HPP) to 5-amino- 6-ribitylamino-2,4 (1H,3H)-pyrimidinedione to form 7,8-didemethyl- 8-hydroxy-5-deazariboflavin (FO) (By similarity), restriction modification system DNA specificity | FS_0433,FS_0685,FS_1560 | TS_0173,TS_0829,TS_0895 |
| OG0733 | COG1060 | H | Catalyzes the radical-mediated transfer of the hydroxybenzyl group from 4-hydroxyphenylpyruvate (HPP) to 5-amino- 6-ribitylamino-2,4 (1H,3H)-pyrimidinedione to form 7,8-didemethyl- 8-hydroxy-5-deazariboflavin (FO) | FS_0433,FS_0685,FS_1560 | TS_0173,TS_0829,TS_0895 |
| OG0734 | NOG09038 | M | Glycosyl transferase | FS_0446 | TS_0825 |
| OG0735 | COG0735,NOG96788 | K,S | Ferric uptake regulator, Fur family | - | - |
| OG0736 | COG2101 | K | General factor that plays a role in the activation of archaeal genes transcribed by RNA polymerase. Binds specifically to the TATA box promoter element which lies close to the position of transcription initiation (By similarity) | FS_0005,FS_1850 | TS_0187,TS_1715 |
| OG0737 | COG1321 | K | Iron (metal) dependent repressor, dtxr family | - | - |
| OG0738 | COG0024 | J | Removes the N-terminal methionine from nascent proteins (By similarity) | FS_0457,FS_1484 | TS_0816 |
| OG0739 | COG2042,NOG07399 | R,S | Methyltransferase, type 11,pre-Rrna processing protein involved in ribosome biogenesis (By similarity) | FS_0466 | TS_0807 |
| OG0740 | NOG74533 | S |  | - | - |
| OG0741 | COG3356 | S | Membrane | FS_0481 | TS_0791 |
| OG0742 | COG1645 | R | Sjogrens syndrome scleroderma autoantigen 1 | FS_0479 | TS_0793 |
| OG0743 | COG0023,NOG07430 | D,J | CAP-GLY domain containing linker protein,translation initiation factor | FS_0478 | TS_0794 |
| OG0744 | COG0744,COG1920,NOG07441 | M,S | Guanylyltransferase that catalyzes the activation of 2- phospho-L-lactate (LP) as (2S)-lactyl-2-diphospho-5’-guanosine (LPPG), via the condensation of LP with GTP. Is involved in the biosynthesis of coenzyme F420, a hydride carrier cofactor (By similarity),nuclease,penicillin-binding protein | FS_0475 | TS_0797 |
| OG0745 | COG0391,COG0745 | S,T | Catalyzes the transfer of the 2-phospholactate moiety from lactyl (2) diphospho-(5’)guanosine (LPPG) to 7,8-didemethyl- 8-hydroxy-5-deazariboflavin (FO) with the formation of the L- lactyl phosphodiester of 7,8-didemethyl-8-hydroxy-5- deazariboflavin (F420-0) and GMP (By similarity),regulatoR | FS_0474,FS_1340 | TS_0799 |
| OG0746 | COG0644 | C | Geranylgeranyl reductase | - | TS_1462 |
| OG0747 | COG2084 | I | Dehydrogenase | FS_2596 | TS_0505 |
| OG0748 | COG0834 | E | (ABC) transporter | FS_1236 | TS_2150 |
| OG0750 | COG0605,COG0750 | M,P | Destroys radicals which are normally produced within the cells and which are toxic to biological systems (By similarity),Membrane-associated zinc metalloprotease | FS_0008,FS_0847,FS_2616 | TS_1913,TS_2103 |
| OG0751 | NOG51816 | T | Sensor with hamp domain | FS_1181 | TS_0075 |
| OG0752 | COG1581 | K | Binds double-stranded DNA tightly but without sequence specificity. It is distributed uniformly and abundantly on the chromosome, suggesting a role in chromatin architecture. However, it does not significantly compact DNA. Binds Rrna and Mrna in vivo. May play a role in maintaining the structural and functional stability of R and, perhaps, ribosomes (By similarity) | FS_0677,FS_0991,FS_1180 | TS_0074,TS_0476,TS_0902, TS_1758 |
| OG0753 | COG1260,NOG07533 | I,U | Dolichyl-diphosphooligosaccharide—protein glycosyltransferase,synthase | FS_1172 | TS_0067 |
| OG0754 | COG4608,NOG07548 | E,S | (ABC) transporter,Fc fragment of IgG binding protein | FS_1169 | TS_0062 |
| OG0755 | COG0442 | J | Catalyzes the attachment of proline to Trna(Pro) in a two-step reaction proline is first activated by ATP to form Pro- AMP and then transferred to the acceptor end of Trna(Pro). As ProRS can inadvertently accommodate and process non-cognate amino acids such as alanine and cysteine, to avoid such errors it has two additional distinct editing activities against alanine. One activity is designated as ‘pretransfer’ editing and involves the Trna(Pro)-independent hydrolysis of activated Ala-AMP. The other activity is designated ‘posttransfer’ editing and involves deacylation of mischarged Ala-Trna(Pro). The misacylated Cys- Trna(Pro) is not edited by ProRS (By similarity) | FS_1245 | TS_0420 |
| OG0756 | NOG161344 | S |  | FS_1246 | TS_0421 |
| OG0757 | COG0560 | E | Phosphoserine phosphatase | FS_1247 | TS_0422 |
| OG0758 | COG1478 | S | Catalyzes the GTP-dependent successive addition of two or more gamma-linked L-glutamates to the L-lactyl phosphodiester of 7,8-didemethyl-8-hydroxy-5-deazariboflavin (F420-0) to form coenzyme F420-0-glutamyl-glutamate (F420-2) or polyglutamated F420 derivatives (By similarity) | FS_1248 | TS_0423 |
| OG0759 | COG0425 | O | Part of a sulfur-relay system required for 2-thiolation of 5-methylaminomethyl-2-thiouridine (mnm(5)s(2)U) at Trna wobble positions. Interacts with IscS and stimulates its activity. Then, accepts a sulfur from IscS and transfers it in turn to TusD (By similarity) | FS_1251 | TS_0426 |
| OG0760 | COG0492,COG0760 | O | Peptidyl-prolyl cis-trans isomerase,thioredoxin reductase | FS_0784,FS_1254 | TS_0428 |
| OG0761 | COG2897 | P | Sulfurtransferase | - | TS_0438 |
| OG0762 | NOG230510 | S | TPR | FS_0485 | TS_0439 |
| OG0763 | COG1163 | R | GTP-Binding protein | FS_0626 | TS_0444 |
| OG0764 | COG0561 | R | ,hydrolase | FS_0277 | TS_0449 |
| OG0765 | COG0018 | J | ArginyL-Trna synthetase | FS_0278 | TS_0450 |
| OG0766 | COG2519 | J | TRNA methyltransferase | FS_0376 | TS_0452 |
| OG0767 | COG0063 | G | Together with NAD(P)HX epimerase, which catalyzes the epimerization of the S- and R-forms, the enzyme allows the repair of both epimers of NAD(P)HX, a damaged form of NAD(P)H that is a result of enzymatic or heat-dependent hydration (By similarity) | FS_0377 | TS_0454 |
| OG0768 | COG0638 | O | The proteasome is a multicatalytic proteinase complex which is characterized by its ability to cleave peptides with Arg, Phe, Tyr, Leu, and Glu adjacent to the leaving group at neutral or slightly basic Ph. The proteasome has an ATP-dependent proteolytic activity (By similarity) | FS_0632 | TS_0457 |
| OG0769 | COG0769,COG2016 | J,M | Catalyzes the addition of meso-diaminopimelic acid to the nucleotide precursor UDP-N-acetylmuramoyl-L-alanyl-D-glutamate (UMAG) in the biosynthesis of bacterial cell-wall peptidoglycan (By similarity),Rna-binding protein | FS_0631 | TS_0458 |
| OG0770 | COG0460 | E | Homoserine dehydrogenase | FS_0630,FS_2269 | TS_0459 |
| OG0771 | COG0771,COG1351 | F,M | Catalyzes the formation of Dtmp and tetrahydrofolate from Dump and methylenetetrahydrofolate (By similarity),Cell wall formation. Catalyzes the addition of glutamate to the nucleotide precursor UDP-N-acetylmuramoyl-L-alanine (UMA) (By similarity) | FS_1721 | TS_0767 |
| OG0772 | COG0772,NOG29002 | D,S | Cell Division Protein | FS_1723 | TS_0765 |
| OG0773 | COG0258 | L | Structure-specific nuclease with 5’-flap endonuclease and 5’-3’ exonuclease activities involved in DNA replication and repair. During DNA replication, cleaves the 5’-overhanging flap structure that is generated by displacement synthesis when DNA polymerase encounters the 5’-end of a downstream Okazaki fragment. It enters the flap from the 5’-end and then tracks to cleave the flap base, leaving a nick for ligation. Also involved in the long patch base excision repair (LP-BER) pathway, by cleaving within the apurinic apyrimidinic (AP) site-terminated flap. | FS_1697 | TS_0774 |
| OG0774 | NOG13018 | S | LPXTG-motif cell wall anchor domain protein | - | - |
| OG0776 | COG0439 | I | Acetyl-CoA carboxylase biotin carboxylase | FS_0998,FS_1745 | TS_1735 |
| OG0777 | COG1173 | P | ABC transporter (Permease | FS_2755 | TS_0785 |
| OG0778 | COG0601,COG0778 | C,P | ABC transporter (permease),nitroreductase | FS_2754 | TS_0787 |
| OG0779 | COG2239,NOG120676 | P,S | Magnesium transporter | - | - |
| OG0780 | COG1060 | H | Catalyzes the radical-mediated transfer of the hydroxybenzyl group from 4-hydroxyphenylpyruvate (HPP) to 5-amino- 6-ribitylamino-2,4(1H,3H)-pyrimidinedione to form 7,8-didemethyl- 8-hydroxy-5-deazariboflavin (FO) (By similarity) | FS_1560 | TS_0173 |
| OG0781 | COG2107 | R | Periplasmic solute-binding protein | FS_1552 | TS_0171 |
| OG0782 | COG0480 | J | Catalyzes the GTP-dependent ribosomal translocation step during translation elongation. During this step, the ribosome changes from the pre-translocational (PRE) to the post- translocational (POST) state as the newly formed A-site-bound peptidyl-Trna and P-site-bound deacylated Trna move to the P and E sites, respectively. Catalyzes the coordinated movement of the two Trna molecules, the Mrna and conformational changes in the ribosome (By similarity) | FS_1444,FS_1550 | TS_0169 |
| OG0784 | COG0784,COG1759 | F,T | Catalyzes the ATP- and formate-dependent formylation of 5-aminoimidazole-4-carboxamide-1-beta-d-ribofuranosyl 5’- monophosphate (AICAR) to 5-formaminoimidazole-4-carboxamide-1- beta-d-ribofuranosyl 5’-monophosphate (FAICAR) in the absence of folates (By similarity),response regulator | FS_0053,FS_0189,FS_1234, FS_1236,FS_1710,FS_1974, FS_2387 | TS_0139,TS_0589,TS_0753,  TS_0757,TS_1032,TS_1659, TS_2133,TS_2150,TS_2154 |
| OG0785 | COG0163,COG0785,NOG07857 | H,O,R | 3-Octaprenyl-4-hydroxybenzoate carboxy-lyase,Cytochrome c biogenesis protein,methyltransferase | FS_0153,FS_0859,FS_1030, FS_1516 | TS_0698,TS_1559 |
| OG0786 | COG0805 | U | Part of the twin-arginine translocation (Tat) system that transports large folded proteins containing a characteristic twin-arginine motif in their signal peptide across membranes. Together with TatB, TatC is part of a receptor directly interacting with Tat signal peptides (By similarity) | FS_0187 | TS_0137 |
| OG0787 | COG3415,NOG115746,NOG50183 | L,S,T | LicD family,STYKc,transposase | FS_0186 | TS_0136 |
| OG0788 | COG0047,COG0788 | F | Formyltetrahydrofolate deformylase,phosphoribosylformylglycinamidine synthase | FS_0878,FS_0961,FS_2470 | TS_0569,TS_0650,TS_2026 |
| OG0789 | COG0046 | F | Phosphoribosylformylglycinamidine synthase | FS_0877,FS_0941,FS_1339 | TS_0651 |
| OG0790 | COG0034 | F | Glutamine phosphoribosylpyrophosphate amidotransferase | FS_1066 | TS_0653 |
| OG0792 | COG0152 | F | SAICAR synthetase | FS_1021,FS_1064 | TS_0663 |
| OG0793 | COG0640,COG0793 | K,M | Transcriptional regulator, arsr family,protease | FS_1062 | TS_0664 |
| OG0794 | COG0097 | J | This protein binds to the 23S Rr and is important in its secondary structure. It is located near the subunit interface in the base of the L7 L12 stalk, and near the Trna binding site of the peptidyltransferase center (By similarity) | FS_1041 | TS_0687 |
| OG0795 | COG0096,COG0795,NOG07952 | J,M,R | One of the primary Rrna binding proteins, it binds directly to 16S Rrna central domain where it helps coordinate assembly of the platform of the 30S subunit (By similarity),Permease yjgp yjgq family,glycosyl transferase group 1 | FS_1042 | TS_0686 |
| OG0796 | COG0094,COG0796 | J,M | This is 1 of the proteins that binds and probably mediates the attachment of the 5S RNA into the large ribosomal subunit, where it forms part of the central protuberance. In the 70S ribosome it contacts protein S13 of the 30S subunit (bridge B1b), connecting the 2 subunits,Provides the I-glutamate required for cell wall biosynthesis (By similarity) | FS_1044 | TS_0684 |
| OG0797 | COG1471 | J | (Ribosomal) protein | FS_1045 | TS_0682 |
| OG0798 | COG0198 | J | One of the proteins that surrounds the polypeptide exit tunnel on the outside of the subunit (By similarity) | - | TS_0681 |
| OG0799 | COG0093 | J | Binds to 23S Rrna. Forms part of two intersubunit bridges in the 70S ribosome (By similarity) | FS_1048 | TS_0680 |
| OG0800 | COG0186,NOG08008 | J,R | One of the primary Rrna binding proteins, it binds specifically to the 5’-end of 16S ribosomal,Lysine-specific histone demethylase | FS_1049 | TS_0679 |
| OG0801 | COG0255 | J | 50s ribosomal protein l29 | FS_1051 | TS_0677 |
| OG0802 | COG0092 | J | Binds the lower part of the 30S subunit head. Binds Mrna in the 70S ribosome, positioning it for translation (By similarity) | FS_1052 | TS_0676 |
| OG0803 | COG0091,COG0803 | J,P | The globular domain of the protein is located near the polypeptide exit tunnel on the outside of the subunit, while an extended beta-hairpin is found that lines the wall of the exit tunnel in the center of the 70S ribosome (By similarity),periplasmic solute binding protein | FS_1053 | TS_0675 |
| OG0804 | COG0185,COG0804 | E,J | Protein S19 forms a complex with S13 that binds strongly to the 16S ribosomal RNA (By similarity),Urea amidohydrolase subunit alpha | FS_1054 | TS_0674 |
| OG0805 | COG0089,COG0805 | J,U | Part of the twin-arginine translocation (Tat) system that transports large folded proteins containing a characteristic twin-arginine motif in their signal peptide across membranes. Together with TatB, TatC is part of a receptor directly interacting with Tat signal peptides (By similarity),One of the early assembly proteins it binds 23S Rrna. One of the proteins that surrounds the polypeptide exit tunnel on the outside of the ribosome. Forms the main docking site for trigger factor binding to the ribosome (By similarity) | FS_1055 | TS_0673 |
| OG0806 | COG0087 | J | One of the primary Rrna binding proteins, it binds directly near the 3’-end of the 23S Rr where it nucleates assembly of the 50S subunit (By similarity) | FS_1057 | TS_0669 |
| OG0807 | COG0638 | O | The proteasome is a multicatalytic proteinase complex which is characterized by its ability to cleave peptides with Arg, Phe, Tyr, Leu, and Glu adjacent to the leaving group at neutral or slightly basic Ph. The proteasome has an ATP-dependent proteolytic activity (By similarity) | FS_1060 | TS_0666 |
| OG0808 | COG2090 | S | Domain of unknown function (DUF371) | FS_1061 | TS_0665 |
| OG0809 | COG1960,COG3419 | I,NU | Pilus assembly protein tip-associated adhesin,acyl-CoA dehydrogenase | FS_1033 | TS_0695 |
| OG0810 | COG0526 | O | Thioredoxin | FS_0784,FS_0859,FS_0969, FS_1254,FS_1516,FS_2241, FS_2243,FS_2737,FS_2762 | TS_0428,TS_0698,TS_1652, TS_1975,TS_2018,TS_2044, TS_2332,TS_2349 |
| OG0811 | COG0785,COG0811 | O,U | Cytochrome c biogenesis protein,MotA TolQ exbB proton channel | FS_1030 | TS_0699 |
| OG0812 | COG0502 | H | Catalyzes the conversion of dethiobiotin (DTB) to biotin by the insertion of a sulfur atom into dethiobiotin via a radical- based mechanism (By similarity) | FS_1027 | TS_0705 |
| OG0813 | COG0161 | E | Catalyzes the transfer of the alpha-amino group from S- adenosyl-L-methionine (SAM) to 7-keto-8-aminopelargonic acid (KAPA) to form 7,8-diaminopelargonic acid (DAPA). It is the only animotransferase known to utilize SAM as an amino donor (By similarity) | FS_1025 | TS_0706 |
| OG0814 | COG0549,COG0795,COG0814,NOG78861 | E,R,S | Amino acid transporter,Permease yjgp yjgq family,carbamate kinase | FS_2484 | TS_1918 |
| OG0815 | COG3425 | I | Hydroxymethylglutaryl-coa synthase | FS_2172 | - |
| OG0816 | COG0498 | E | Threonine synthase | FS_0938,FS_1137,FS_2166 | - |
| OG0817 | COG0543 | C | Responsible for channeling the electrons from the oxidation of dihydroorotate from the FMN redox center in the PyrD type B subunit to the ultimate electron acceptor NAD( ) (By similarity) | FS_1944 | TS_1537 |
| OG0818 | COG0818,COG1591,COG2356,NOG02631,NOG09261 | J,L,M,O | Processing of precursor 1, ribonuclease P MRP subunit (S. cerevisiae),Diacylglycerol kinase,FAD-dependent oxidoreductase domain containing 2,Holliday junction resolvase,endonuclease I | FS_2067 | - |
| OG0819 | COG1545 | S | Nucleic-acid-binding protein containing a Zn-ribbon | FS_2056 | - |
| OG0820 | COG0820,COG2141 | C,J | Monooxygenase,Specifically methylates position 2 of adenine 2503 in 23S Rrna and position 2 of adenine 37 in tRNAs | FS_1269 | - |
| OG0821 | COG1208 | M | nucleotidyl transferase | FS_1270 | TS_0921 |

*The published *Thaumarchaeota* share 743 core genome gene families that was identified by Herbold et al. (17).

**Table S7. Genes implicated in carbon metabolisms in the FS and TS genomes.**

| **Gene** | **Product** | **EC no.** | **FS Locus** | **Best BLAST hit in NCBI nr (% identity)*** | **TS Locus** | **Best BLAST hit in NCBI nr (% identity)*** | **Comment** |
| --- | --- | --- | --- | --- | --- | --- | --- |
| **TCA cycle** | | | | | | | |
| ytsJ | NAD(H)-dependent malic enzyme | 1.1.1.38 | FS_1924 | Nitrosarchaeum limnium, EPA06504, 64% | TS_2197 | Planctomyces limnophilus DSM 3776, ADG67616, 62% |  |
| mdh | Malate dehydrogenase | 1.1.1.37 | FS_0356 | Nitrosopumilus maritimus, ABX12234, 79% | - | **-** |  |
| sdhA | succinate dehydrogenase/fumarate reductase, flavoprotein subunit | 1.3.99.1 | FS_0993 | Thaumarchaeota archaeon N4, CDI06292, 81% | TS_1775 | Thaumarchaeota archaeon N4, CDI06292, 81%/81% |  |
| sdhC | succinate dehydrogenase/fumarate reductase, subunit D | 1.3.99.1 | FS_0994 | Thaumarchaeota archaeon N4, CDI06291, 77% | TS_1774 | Thaumarchaeota archaeon MY2, gi\|757129328, 68%/68% |  |
| sdhD | succinate dehydrogenase/fumarate reductase | 1.3.99.1 | FS_0995 | Cenarchaeum symbiosum, ABK78317, 70% | TS_1753 | Thaumarchaeota archaeon MY2, gi\|757129327, 72%/72% |  |
| sdhB | Succinate dehydrogenase/fumarate reductase iron-sulfur protein | 1.3.99.1 | FS_0996 | Thaumarchaeota archaeon N4, CDI06289, 75% | TS_1752 | Nitrosarchaeum koreensis, EGP92990, 75%/Nitrosarchaeum koreensis MY1, EGP92990, 75% |  |
| sucD | succinyl-CoA synthetase subunit alpha | 6.2.1.5 | - | **-** | TS_0535 | Thaumarchaeota archaeon MY2, gi\|757132784, 72% |  |
| sucC | succinyl-CoA synthetase subunit beta | 6.2.1.5 | FS_2498 | Nitrosarchaeum limnium, EGG42212, 66% | TS_0538 | Thaumarchaeota archaeon MY2, gi\|757132782, 75% |  |
|  | 2-oxoglutarate ferredoxin oxidoreductase subunit alpha | 1.2.7.3 | FS_1776 | Thaumarchaeota archaeon MY2, gi\|757130770, 68% | TS_1938 | Thaumarchaeota archaeon MY2, gi\|757130770, 68% |  |
|  | 2-oxoglutarate ferredoxin oxidoreductase, beta subunit | 1.2.7.3 | FS_1780 | Nitrosopumilus sp. AR2, AFS82241, 76% | TS_1940 | Nitrosopumilus salaria, EIJ65086, 78% |  |
|  | isocitrate dehydrogenase | 1.1.1.42 | FS_2747 | Thaumarchaeota archaeon N4, CDI05831, 68% | TS_0050 | Nitrosarchaeum koreensis MY1, EGP93998, 74% |  |
| aco | aconitate hydratase | 4.2.1.3 | FS_0090 | Nitrosarchaeum limnium SFB1, EGG41053, 74% | TS_1947/1622 | Thaumarchaeota archaeon N4, gi\|851357069, 83%/Nitrosopumilus maritimus, ABX12317, 82%/Thaumarchaeota archaeon N4, CDI06166, 74% |  |
| gltA | 2-methylcitrate synthase/citrate synthase II | 2.3.3.1 | FS_0911 | Thaumarchaeota archaeon N4, CDI05857, 67% | TS_0615/0734 | Thaumarchaeota archaeon N4, CDI05857, 68%/68% |  |
| citE | citry-CoA lyase/citrate lyase subunit beta | 4.1.3.6 | FS_2653 | Thaumarchaeota archaeon MY2, gi\|757130112, 68% | TS_1083 | Nitrosarchaeum limnium, EGG41763, 65% |  |
| fumC | putative fumarate hydratase | 4.2.1.2 | FS_0530 | Nitrosopumilus maritimus, ABX13156, 75% | TS_1330 | Nitrosarchaeum limnium, EPA06357, 76% |  |
| **Gene** | **Product** | **EC no.** | **FS Locus** | **Best BLAST hit in NCBI nr (% identity)*** | **TS Locus** | **Best BLAST hit in NCBI nr (% identity)*** | **Comment** |
| **3-hydroxypropionate-4-hydroxybutyrate** | | | | | | | |
| accA/  pccA | Acetyl/propionyl-CoA carboxylase, subunit | 6.2.1.2/3 | FS_1774 | Nitrosarchaeum limnium, EGG41960, 56% | TS_1734 | Thaumarchaeota archaeon N4, CDI06274, 59% |  |
| accC/  pccC | Acetyl/propionyl-CoA carboxylase, subunit | 6.2.1.2/3 | FS_1745 | Nitrosarchaeum limnium, EPA04845, 74% | TS_1735 | Nitrosarchaeum limnium, EPA04845, 73% |  |
| accB/  pccB | Acetyl/propionyl-CoA carboxylase, subunit | 6.2.1.2/3 | FS_1747 | Thaumarchaeota archaeon MY2, gi\|757129321, 80% | TS_1736 | Thaumarchaeota archaeon MY2, gi\|757129321, 78% |  |
|  | 3-hydroxypropionyl-CoA synthetase (ADP-forming)/acyl-CoA synthetase | 6.2.1.36 | FS_2394/569 | Thaumarchaeota archaeon N4, CDI06027, 83%/Thaumarchaeota archaeon MY2, gi\|757129380, 83% | TS_1283/2050 | Thaumarchaeota archaeon N4, CDI06027, 82%/Thaumarchaeota archaeon N4, CDI05052, 78% |  |
|  | 3-Hydroxypropionyl-CoA dehydratase/enoyl-CoA hydratase/isomerase | 4.2.1.1.16 | FS_570/1478 | Thaumarchaeota archaeon MY2, gi\|757131246, 71%/Cenarchaeum symbiosum, ABK76811, 58% | TS_1281/1512 | Nitrosarchaeum limnium, EGG42023, 72%/Nitrososphaera gargensis, AFU59128, 52% |  |
|  | methylmalonyl-CoA epimerase | 5.4.99.1 | FS_1299/1563 | Thaumarchaeota archaeon N4, CDI05620, 59%/Nitrosarchaeum limnium, EPA05250, 51% | TS_518 | Nitrosopumilus maritimus, ABX12849, 59%, |  |
|  | methylmalonyl-CoA mutase large subunit | 5.4.99.2 | FS_238 | Nitrosarchaeum koreensis MY1, EGP93890, 65% | TS_517 | Thaumarchaeota archaeon MY2, gi\|757132382, 78% |  |
|  | methylmalonyl-CoA mutase, small subunit, C-terminus | 5.4.99.2 | - |  | TS_515 | Thaumarchaeota archaeon MY2, gi\|757132386, 76% |  |
|  | 4-Hydroxybutyryl-CoA synthetase (ADP-forming) | 6.2.1.36 | FS_2394 | Thaumarchaeota archaeon MY2, gi\|757129380, 83% | TS_2050 | Thaumarchaeota archaeon N4, CDI05052, 78% |  |
|  | 4-Hydroxybutyryl-CoA dehydratase | 4.2.1.1.20 | FS_0505 | Thaumarchaeota archaeon N4, CDI05065, 82% | TS_2048 | Cenarchaeum symbiosum, ABK78291, 78% |  |
|  | Crotonyl-CoA hydratase[(s)-3-hydroxybutyryl-CoA forming] | 4.2.1.55 | FS_570/1478 | Thaumarchaeota archaeon MY2, gi\|757131246, 71%/Cenarchaeum symbiosum, ABK76811, 58% | TS_1281/1512 | Nitrosarchaeum limnium, EGG42023, 72%/Nitrososphaera gargensis, AFU59128, 52% |  |
|  | (S)-3-hydroxybutyryl-CoA dehydrogenase (NAD) | 1.1.1.157 | FS_2106 | Nitrosopumilus maritimus, ABX12924, 74% | - |  |  |
|  | Acetoacetyl-CoA-β-ketothiolase | EC2.3.1.9 | FS_2055/2664 | Nitrosarchaeum limnium, EPA04635, 63%/Thaumarchaeota N4, CDI04930, 90% | TS_1102 | Thaumarchaeota archaeon N4, CDI05065, 82% |  |
| **Gene** | **Product** | **EC no.** | **FS Locus** | **Best BLAST hit in NCBI nr (% identity)*** | **TS Locus** | **Best BLAST hit in NCBI nr (% identity)*** | **Comment** |
| **Candidate genes for 3-hydroxypropionate-4-hydroxybutyrate** | | | | | | | |
| asd | aspartate-semialdehyde dehydrogenase | 1.2.1.11 | FS_2632 | Thaumarchaeota archaeon N4, CDI05072, 72% | TS_1062 | Thaumarchaeota archaeon N4, CDI05072, 74% |  |
| acsA-1 | acetyl-CoA synthetase | 6.2.1.1 | FS_1226 | Thaumarchaeota archaeon N4, CDI06289, 70% | TS_762 | Nitrosopumilus maritimus, ABX12597, 71% |  |
| gadD | succinate-semialdehyde dehydrogenase/aldehyde dehydrogenase | 1.2.1.16 | FS_0163 | Thaumarchaeota archaeon MY2, gi\|757129328, 70% | TS_0242/0129/0127 | Nitrosopumilus koreensis, AFS81602, 66%/Thaumarchaeota archaeon N4, CDI06710, 68% |  |
| **Gene** | **Product** | **EC no.** | **FS Locus** | **Best BLAST hit in NCBI nr (% identity)*** | **TS Locus** | **Best BLAST hit in NCBI nr (% identity)*** | **Comment** |
| **Alcohol dehydrogenase, EC 1.1.1-** | | | | | | | |
|  | Zn-dependent alcohol dehydrogenase | 1.1.1.1 | FS_0093/1096/2654/ 0325/1348/2344 | Nitrososphaera gargensis, AFU60030, 46%/Nitrososphaera gargensis, AFU57654, 54%/Thaumarchaeota archaeon MY2, gi\|757130110, 80%/Nitrososphaera gargensis, AFU59601, 61%/Thaumarchaeota archaeon MY2, gi\|757129412, 85%/N. limnium, EGG42319, 63% | TS_0040/0294/0886/1085/1620/2216 | Nitrosopumilus koreensis, AFS81170, 62%/Nitrosopumilus koreensis, AFS81560, 68%/Azospirillum amazonense, EGY00932, 72%/Thaumarchaeota archaeon MY2, gi\|757130110, 84% /Nitrososphaera gargensis, AFU60030, 47%/Nitrososphaera evergladensis, AIF83236, 72%. |  |
|  | Short-chain alcohol dehydrogenase | 1.1.1.1 | FS_0059/0580/1288/1485 | Nitrososphaera evergladensis, AIF85219 , 59%/Thaumarchaeota archaeon N4, CDI06046, 69%/Nitrosarchaeum koreensis MY1, EGP93391, 68% | TS_0463/0559/1270/1508/1865 | Nitrosarchaeum koreensis, EGP93965, 65%/Nitrosarchaeum limnium, EPA05989, 50%/Nitrosarchaeum limnium, EPA05989, 66%/Nitrosopumilus maritimus, ABX11944, 66%/Thaumarchaeota archaeon N4, CDI06623, 73% |  |
|  | Iron-containing alcohol dehydrogenase | 1.1.1.1 | FS_0919 | Thaumarchaeota archaeon MY2, gi\|757132703, 82% | TS_0723/0810 | Thaumarchaeota archaeon MY2, gi\|757132703, 79%/Nitrosopumilus salaria, EIJ66043, 33% |  |
| **Gene** | **Product** | **EC no.** | **FS Locus** | **Best BLAST hit in NCBI nr (% identity)*** | **TS Locus** | **Best BLAST hit in NCBI nr (% identity)*** | **Comment** |
| **Gluconeogenesis** | | | | | | | |
| pckA | PEP carboxykinase | 4.1.1.49 | FS_0843 | Thaumarchaeota archaeon N4, CDI05231, 74% | TS_1910 | Thaumarchaeota archaeon N4, CDI05231, 74% |  |
| ppdk | pyruvate, phosphate dikinase | 2.7.9.1/2 | - | - | TS_0524 | Thaumarchaeota archaeon MY1, EGP93886, 70% |  |
| eno | phosphopyruvate hydratase | 4.2.1.11 | FS_0338 | Thaumarchaeota archaeon MY1, EGP93049, 58% | TS_2304 | Nitrosopumilus sp. AR2,AFS82118, 74% |  |
| apgM | 2,3-bisphosphoglycerate-independent phosphoglycerate mutase | 5.4.2.1 | FS_0656 | Thaumarchaeota archaeon N4, CDI04874, 75% | TS_0928 | Thaumarchaeota archaeon N4, CDI04874, 75% |  |
| gpmB | putative 2,3-bisphosphoglycerate-dependent phosphoglycerate mutase | 5.4.2.1 | FS_0300 | Thaumarchaeota archaeon MY2, gi\|757129574, 56% | TS_2168 | Thaumarchaeota archaeon MY2, gi\|757129574, 58% |  |
| pgk | Phosphoglycerate kinase | 2.7.2.3 | FS_2014 | Nitrosarchaeum koreensis, EGP93226, 79% | TS_0994 | Thaumarchaeota archaeon MY1, EGP93226, 78% |  |
| gap | glyceraldehyde-3-phosphate dehydrogenase | 1.2.1.59 | FS_2169 | Thaumarchaeota archaeon N4, CDI05591, 60% | - | - |  |
| tpiA | triosephosphate isomerase | 5.3.1.1 | FS_0246 | Nitrosopumilus maritimus, ABX12846, 71% | TS_1147 | Nitrosopumilus maritimus, ABX12846, 69% |  |
|  | ignalin-2-dehydro-3-deoxyheptonate aldolase | 4.1.2.13 | FS_0386 | Thaumarchaeota archaeon N4, CDI04890, 78% | TS_897 | Thaumarchaeota archaeon MY2, gi\|757131174, 79% |  |
| fbp | fructose-1,6-bisphosphatase | 3.1.3.11 | FS_2180 | Nitrosopumilus koreensis AR1, AFS81038, 80% | - | - |  |
|  | phosphoglucomutase/phosphomannomutase subunit alpha/beta | 5.4.2.2 | FS_0110 | Nitrosarchaeum limnium, EGG42584, 69% | TS_1599 | Nitrosarchaeum limnium, EGG41373, 66% |  |
|  | Phosphoglucosamine mutase | 5.4.2.2 | FS_0963 | Thaumarchaeota archaeon MY2, gi\|757129355, 64% | TS_2025 | Thaumarchaeota archaeon MY2, gi\|757129355, 68% |  |
| mdh | lactate/Malate dehydrogenase | 1.1.1.37 | FS_0356 | Nitrosopumilus maritimus SCM1, ABX12234, 79% | - | - |  |
| pgi | Glucose-6-phosphate isomerase | 5.3.1.9 | - | - | TS_1396 | Marine Group I thaumarchaeote SCGC RSA3, KFM19940, 60% |  |
| **Gene** | **Product** | **EC no.** | **FS Locus** | **Best BLAST hit in NCBI nr (% identity)*** | **TS Locus** | **Best BLAST hit in NCBI nr (% identity)*** | **Comment** |
| **Putative oxidative pentose-phosphate pathway** | | | | | | | |
|  | putative coenzyme F420-dependent glucose-6-phosphate dehydrogenase | 1.1.98.2 | FS_0920 | Thaumarchaeota archaeon N4, CDI05865, 77% | TS_0721 | Thaumarchaeota archaeon N4, CDI05865, 75% | might substitute function of Glucose-6-phosphate dehydrogenase (EC 1.1.1.49) |
|  | Zn-dependent hydrolase of the beta-lactamase fold |  | FS_2557 | Thaumarchaeota archaeon N4, CDI06541, 75% | TS_1467 | Nitrososphaera gargensis, KER05842, 53% | might substitute function of 6-phosphogluconolactonase (EC 3.1.1.31) |
| gnd | 6-phosphogluconate dehydrogenase | 1.1.1.44 | FS_1917 | Nitrosopumilus salaria, gi\|495574284, 53% | TS_0004, TS_2215 | Thaumarchaeota archaeon N4, CDI05638, 65%/Thaumarchaeota archaeon N4, CDI06700, 48% |  |
| **Gene** | **Product** | **EC no.** | **FS Locus** | **Best BLAST hit in NCBI nr (% identity)*** | **TS Locus** | **Best BLAST hit in NCBI nr (% identity)*** | **Comment** |
| **Nonoxidative pentose-phosphate pathway** | | | | | | | |
| rpiA | ribose-5-phosphate isomerase | EC 5.3.1.6 | FS_2254 | Thaumarchaeota archaeon N4, CDI05100, 67% | TS_2032 | Thaumarchaeota archaeon N4, CDI05100, 63% |  |
| tktA+rpe | ribulose-phosphate 3-epimerase protein with N-terminal transketolase domain | 5.1.3.1. and 2.2.1.1 | FS_0016 | uncultured marine crenarchaeote, ABZ08016, 75% | TS_1702 | uncultured marine thaumarchaeote, AIF17072, 76% |  |
| tktB | transketolase C-terminal subunit | 2.2.1.1 | FS_0017 | Nitrosarchaeum limnium SFB1, EGG41933, 76% | TS_1698 | uncultured marine thaumarchaeote, AIF10572, 80% |  |
| tal | transaldolase | 2.2.1.2 | FS_0018 | Thaumarchaeota archaeon N4, CDI06214, 79% | TS_1697 | Thaumarchaeota archaeon N4, CDI06214, 82% |  |
| prs | ribose-phosphate pyrophosphokinase | 2.7.6.1 | FS_1644 | Nitrosarchaeum koreensis MY1, EGP94624, 59% | TS_2198 | Nitrosarchaeum koreensis MY1, EGP94624, 64% |  |
| **Gene** | **Product** | **EC no.** | **FS Locus** | **Best BLAST hit in NCBI nr (% identity)*** | **TS Locus** | **Best BLAST hit in NCBI nr (% identity)*** | **Comment** |
| **Sugar isomerases** | | | | | | | |
|  | hypothetical protein with conserved Cupin 2 barrel domain | 5.3.1.8 | FS_2214 | Thaumarchaeota archaeon N4, CDI05890, 69% | TS_1038 | Thaumarchaeota archaeon N4, CDI05890, 47% |  |
|  | alpha-D-phosphohexomutase | 5.4.2- | FS_0110, FS_0963 | Nitrosopumilus sp. AR2, gi\|407465443, 62%/Nitrosopumilus maritimus SCM1, ABX12120, 62% | TS_1599, TS_2025 | Nitrosarchaeum limnium SFB1, EGG41373, 66%/uncultured marine thaumarchaeote, AIE96950, 58% |  |
|  | putative phosphosugar isomerase |  | FS_343,FS_2150 | Thaumarchaeota N4, CDI05181, 72%/66% | TS_2299 | Thaumarchaeota N4, KFM17180, 66% |  |
|  | mannose-1-phosphate guanyltransferase | 2.7.7.13 | FS_663 | Nitrosarchaeum koreensis MY1, EGP93294, 55% | TS_0921 | Nitrosopumilus salaria BD31, EIJ65724, 70% |  |
| **Gene** | **Product** | **EC no.** | **FS Locus** | **Best BLAST hit in NCBI nr (% identity)*** | **TS Locus** | **Best BLAST hit in NCBI nr (% identity)*** | **Comment** |
| **Mannosylglycerate synthase and HAD-like dehalogenases** | | | | | | | |
| ttuD | putative hydroxypyruvate reductase | 1.1.1.81 | FS_0916 | Thaumarchaeota archaeon N4, CDI05862, 52% | TS_611, TS_0730 | Thaumarchaeota archaeon N4, CDI05862, 52% |  |
|  | putative phosphoserine phosphatase, HAD-like hydrolase family | 3.1.3.3 | FS_1247 | Nitrosopumilus maritimus SCM1, ABX12562, 76% | TS_0422 | Nitrosarchaeum koreensis MY1, EGP93424, 74% |  |
| **Gene** | **Product** | **EC no.** | **FS Locus** | **Best BLAST hit in NCBI nr (% identity)*** | **TS Locus** | **Best BLAST hit in NCBI nr (% identity)*** | **Comment** |
| **Archaeal Lipid biosynthesis: Synthesis of glycerol phosphate backbone** | | | | | | | |
| egsA | sn-glycerol-1-phosphate dehydrogenase | 1.1.1.261 | FS_0716 | uncultured marine crenarchaeote, ABZ09691, 61% | TS_1204 | uncultured marine thaumarchaeote, AIF19836, 67% |  |

* Best non-*Nitrosotalea* hit.

**Table S8. Presence of horizontally acquried genes in the ‘*Nitrosotalea*-specific core’ gene set identified by Herbold et al*.* (17) in the FS and TS genomes.**

| **Genbank accession/ Locus ID in *Ca.* N. devanaterra Nd1** | **Predicted function** | **FS** | **TS** |
| --- | --- | --- | --- |
| CUR51883.1 | Divalent heavy-metal cation transporter (zinc permease?) | FS_1971 | TS_0401 |
| CUR52062.1 | Na^+^/H^+^ solute symporter | FS_1852 | TS_0186 |
| CUR52158.1 | acpD \| FMN-dependent NADH-azoreductase | FS_0921 | TS_0562 |
| CUR51850.1 | mntH \| NRAMP family Mn^2+^/Fe^2+^ transporter | FS_1307 | TS_0954 |
| CUR52192.1 | Coiled-coil motif protein | FS_1372 | TS_0727 |
| CUR51294.1 | FKBP-type peptidyl-prolyl cis-trans isomerase | FS_0657 | TS_0927 |
| CUR52193.1 | Putative phage protein | FS_1373 | TS_0606, TS_0726 |
| CUR51439.1 | Unknown (pentapeptide repeat containing protein) | FS_0437 | TS_0396 |

**Table S9. Specific gene sets in the FS and TS genomes but absent in the non-acidophilic thaumarchaeotal genomes.**

| **Genome Specific Genes** | | **Cluster of Orthologous Genes (COGs, 4.0)** | | | **Best BLAST hit in NCBI nr**  **(% identity)*** | **Predicted function** |  |
| --- | --- | --- | --- | --- | --- | --- | --- |
| **FS** | **TS** | **Class** | **Class functions** | **Cluster** |  |  |  |
| FS_0039 | TS_1676 | Unassigned |  |  | uncultured marine thaumarchaeote KM3_78_D03, \|AIF17601, 32% | hypothetical protein |  |
| FS_0044 | TS_1671 | Unassigned |  |  | planctomycete KSU-1, gi\|494426153, 32% | hypothetical protein |  |
| FS_2297 | TS_1998 | Unassigned |  |  | Mollicutes bacterium HR2, KFZ27346, 39% | HNH endonuclease |  |
| FS_2448 | TS_0937 | Unassigned |  |  | zeta proteobacterium SCGC AB-604-B04, gi\|517091700, 37% | hypothetical protein |  |
| FS_2537 | TS_1353 | Unassigned |  |  | Cyanothece sp. PCC 7822, gi\|503085825, 33% | sodium:proton antiporter |  |
| FS_2683 | TS_1117 | Unassigned |  |  | uncultured marine crenarchaeote HF4000_ANIW137N13, ABZ07626, 52% | putative Vacuolar (H+)-ATPase G subunit |  |
| FS_2691 | TS_1125 | Unassigned |  |  | uncultured marine thaumarchaeote KM3_191_E02, AIF06356, 44% | hypothetical protein |  |
| FS_0179 | TS_2061 | Unassigned |  |  | Marine Group I thaumarchaeote SCGC AAA799-D11, KFM16653, 38% | Ig family protein |  |
| FS_0180 | TS_2062 | Unassigned |  |  | Marine Group I thaumarchaeote SCGC AAA799-D11, KFM16652, 44% | MG2 domain protein |  |
| FS_0182 | TS_2064 | Unassigned |  |  | Marine Group I thaumarchaeote SCGC AAA799-D11, KFM16652, 36% | MG2 domain protein |  |
| FS_0183 | TS_0402,TS_0319, TS_1280 | Unassigned |  |  | Streptomyces auratus, gi\|493656763, 30% | outer membrane adhesin-like protein |  |
| FS_0194 | TS_1192 | Unassigned |  |  | Microcoleus sp. PCC 7113, gi\|504994098\|, 37% | arabinose efflux permease family protein |  |
| FS_0375 | TS_1627 | Unassigned |  |  | - | Exported protein of unknown function |  |
| FS_0408 | TS_0257 | Unassigned |  |  | - | Coiled-coil motif protein |  |
| FS_0437 | TS_0396 | Unassigned |  |  | Kamptonema formosum, gi\|518316762, 41% | hypothetical protein |  |
| FS_0438 | TS_399 | Unassigned |  |  | Nitrolancea hollandica, gi\|495753724, 60% | multicopper oxidase |  |
| FS_0454 | TS_0817 | Unassigned |  |  | uncultured marine thaumarchaeote SAT1000_09_A04, AIF22256, 55% | hypothetical protein |  |
| FS_0657 | TS_0927 | Unassigned |  |  | Candidatus Methanoperedens nitroreducens, KCZ71978, 48% | FKBP-type peptidyl-prolyl cis-trans isomerase |  |
| FS_0734 | TS_0260 | Unassigned |  |  | - | Chromosome segregation ATPase-like protein |  |
| FS_0772 | TS_0159 | Unassigned |  |  | - | Exported protein of unknown function |  |
| FS_0879 | TS_0649, TS_0648 | Unassigned |  |  | Synechococcus sp. NKBG15041c, gi\|639206599, 38% | hypothetical protein |  |
| FS_0895 | TS_0869 | Unassigned |  |  | Marine Group I thaumarchaeote SCGC AAA799-B03, KFM22236, 41% | putative membrane-bound metal-dependent hydrolase protein |  |
| FS_0921 | TS_0562 | Unassigned |  |  | uncultured bacterium, EKD52264, 55% | acyl carrier protein phosphodiesterase |  |
| FS_1183 | TS_0078 | Unassigned |  |  | candidate division YNPFFA, gi\|516977678, 35% | hypothetical protein |  |
| FS_1307 | TS_0954 | Unassigned |  |  | Serratia plymuthica, gi\|493366053, 38% | manganese transporter |  |
| FS_1372 | TS_0727, TS_0607 | Unassigned |  |  | Thermoplasmatales archaeon A-plasma, gi\|546152856, 41% | hypothetical protein |  |
| FS_1373 | TS_0606, TS_0726 | [S] | Function unknown | COG4260 | Thermoplasmatales archaeon Gpl, gi\|546147137, 55% | hypothetical protein |  |
| FS_1374 | TS_0725, TS_0828 | Unassigned |  |  | Thermoplasmatales archaeon A-plasma, gi\|546152858, 43% | hypothetical protein |  |
| FS_1476 | TS_1514 | Unassigned |  |  | Lactobacillus, gi\|545606736, 24% | putative sugar uptake protein |  |
| FS_1581 | TS_0184 | Unassigned |  |  | Candidatus Methylomirabilis oxyfera, gi\|506225137, 41% | hypothetical protein |  |
| FS_1664 | TS_1221 | Unassigned |  |  | Leptospira inadai, gi\|498098888, 40% | hypothetical protein |  |
| FS_1665 | TS_1221 | Unassigned |  |  | Leptospira licerasiae, gi\|495865824, 38% | hypothetical protein |  |
| FS_1699 | TS_0775 | Unassigned |  |  | - | Protein of unknown function |  |
| FS_1707 | TS_0152 | Unassigned |  |  | Methanothermobacter marburgensis, gi\|503061404, 44% | tetratricopeptide repeat domain-containing protein |  |
| FS_1836 | TS_0333, TS_0333 | Unassigned |  |  | Gloeocapsa sp. PCC 7428, gi\|505000455, 47% | HAD-superfamily hydrolase, subfamily IA, variant |  |
| FS_1852 | TS_0186 | Unassigned |  |  | Sulfolobus islandicus, gi\|504324792, 49% | sodium:solute symporter |  |
| FS_1859 | TS_0147 | Unassigned |  |  | Sulfolobus islandicus, gi\|502115633, 48% | sugar ABC transporter permease |  |
| FS_1875 | TS_1161 | Unassigned |  |  | Marine Group I thaumarchaeote SCGC AAA799-E16, KER07120, 49% | Demethylmenaquinone methyltransferase protein |  |
| FS_1926 | TS_1647 | Unassigned |  |  | Nitrosococcus oceani, KFI21071, 86% | hypothetical protein |  |
| FS_1971 | TS_0401 | Unassigned |  |  | unclassified Crenarchaeota , gi\|516670120, 38% | Divalent heavy-metal cation transporter |  |
| FS_2212 | TS_0821 | Unassigned |  |  | Clostridium ljungdahlii, gi\|503004797, 57% | hypothetical protein |  |
| FS_2195 | TS_1403 | Unassigned |  |  | Candidatus Thiomargarita nelsonii, KHD08768, 33% | hypothetical protein |  |
| FS_2197 | TS_1160 | Unassigned |  |  | Thermoplasmatales archaeon I-plasma, gi\|546149619, 62% | hypothetical protein |  |
| FS_2316 | TS_0377 | Unassigned |  |  | Candidatus Parvarchaeum acidophilus ARMAN-5, gi\|290559060, 41% | sodium/hydrogen exchanger |  |
| FS_2322 | TS_2094 | Unassigned |  |  | Sediminibacterium sp. OR43, gi\|652379024, 49% | hypothetical protein |  |
| FS_0723 | TS_2026 | Unassigned |  |  | Marine Group I thaumarchaeote SCGC AAA799-P11, KFM20130, 40% | hypothetical protein |  |
| FS_1071 | TS_2026 | Unassigned |  |  | Marine Group I thaumarchaeote SCGC AAA799-P11, KFM20130, 42% | hypothetical protein |  |
| FS_0045 | - | Unassigned |  |  | uncultured marine crenarchaeote HF4000_ANIW97P9,ABZ07131, 35% | putative copper-binding protein |  |
| FS_0066 | - | Unassigned |  |  | Archaeoglobus profundus,gi\|502705910, 37% | hypothetical protein |  |
| FS_0196 | - | [S] | Function unknown | COG4705 | Bacillus cereus, gi\|488128921, 69% | membrane protein |  |
| FS_0140 | - | Unassigned |  |  | Nitrosococcus oceani C-27,gi\|672875692, 70% | hypothetical protein |  |
| FS_1103 | - | Unassigned |  |  | Leeuwenhoekiella sp. MAR_2009_132, gi\|670480575, 33% | sugar phosphate isomerase |  |
| FS_1106 | - | Unassigned |  |  | Synechococcus sp. PCC 7335, gi\|493502965, 36% | PKD domain protein |  |
| FS_1107 | - | Unassigned |  |  | Sphaerobacter thermophilus, gi\|502636563, 33% | glucose sorbosone dehydrogenase |  |
| FS_1354 | - | Unassigned |  |  | Methanosaeta thermophila, gi\|500015922, 44% | TPR repeat-containing protein |  |
| FS_1396 | - | [S] | Function unknown | COG4705 | Streptomyces cellulosae, gi\|664132890, 64% | membrane protein |  |
| FS_1960 | - | Unassigned |  |  | uncultured crenarchaeote, gi\|42557725, 40% | UDP-3-O-(3-hydroxymyristoyl) glucosamin N-acyltransferase |  |
| FS_2424 | - | Unassigned |  |  | Ilumatobacter coccineus, gi\|505256210, 35% | hypothetical protein |  |
| FS_2426 | - | Unassigned |  |  | Candidatus Solibacter usitatus, gi\|500007767, 38% | NHL repeat-containing protein |  |
| FS_2553 | - | Unassigned |  |  | uncultured crenarchaeote, gi\|82697889, 43% | putative copper binding protein |  |
| FS_2580 | - | Unassigned |  |  | Marine Group I thaumarchaeote SCGC AAA799-P11, KFM20130, 40% | hypothetical protein |  |
| FS_0203 | - | Unassigned |  |  | uncultured marine crenarchaeote HF4000_ANIW93J19, gi\|167042257, 61% | putative CorA-like Mg2+ transporter protein |  |
| FS_0210 | - | Unassigned |  |  | Stigmatella aurantiaca, gi\|488693029, 31% | carbohydrate-binding protein |  |
| FS_0727 | - | Unassigned |  |  | Marine Group I thaumarchaeote SCGC AAA799-E16, gi\|662556783, 47% | hypothetical protein |  |
| FS_0888 | - | Unassigned |  |  | Vulcanisaeta moutnovskia, gi\|503370964, 37% | hypothetical protein |  |
| FS_1296 | - | Unassigned |  |  | uncultured marine thaumarchaeote KM3_87_H02, gi\|663529876, 70% | hypothetical protein |  |
| FS_1346 | - | [S] | Function unknown | COG3042 | Cupriavidus taiwanensis, gi\|648472556, 53% | hemolysin |  |
| FS_1350 | - | Unassigned |  |  | uncultured marine thaumarchaeote SAT1000_15_H02, gi\|663533533, 27% | hypothetical protein |  |
| FS_1406 | - | Unassigned |  |  | Candidatus Micrarchaeum acidiphilum ARMAN-2, gi\|255513312, 33% | hypothetical protein |  |
| FS_1521 | - | Unassigned |  |  | Methanosarcina barkeri, gi\|499624718, 40% | hypothetical protein |  |
| FS_1645 | - | Unassigned |  |  | candidate division WWE3 bacterium RAAC2_WWE3_1, gi\|563772607, 47% | hypothetical protein |  |
| FS_1617 | - | Unassigned |  |  | Frankia sp. CN3, gi\|494780496, 51% | hypothetical protein |  |
| FS_1677 | - | Unassigned |  |  | bacterium JKG1, gi\|649577200, 33% | hypothetical protein |  |
| FS_1679 | - | Unassigned |  |  | Arenitalea lutea, gi\|518217636, 30% | hypothetical protein |  |
| FS_1765 | - | Unassigned |  |  | Haloferax elongans, gi\|495597514, 35% | hypothetical protein |  |
| FS_1846 | - | Unassigned |  |  | Leptospira biflexa, gi\|501452615, 30% | lyas |  |
| FS_1856 | - | Unassigned |  |  | uncultured Acidilobus sp. CIS, gi\|557424065, 46% | Protein of unknown function (DUF3311) |  |
| FS_1881 | - | Unassigned |  |  | Methanococcus aeolicus, gi\|500684901, 25% | hypothetical protein |  |
| FS_1883 | - | Unassigned |  |  | Candidatus Methanoperedens nitroreducens, gi\|630828850, 45% | hypothetical protein |  |
| FS_1884 | - | Unassigned |  |  | Oscillatoria sp. PCC 10802, gi\|516325343, 59% | hypothetical protein |  |
| FS_1885 | - | Unassigned |  |  | Burkholderia acidipaludis, gi\|654281935, 45% | hypothetical protein |  |
| FS_1900 | - | Unassigned |  |  | Ktedonobacter racemifer, gi\|495194024, 64% | phospholipase C |  |
| FS_1985 | - | Unassigned |  |  | Leptolyngbya boryana, gi\|515855544, 30% | hypothetical protein |  |
| FS_1986 | - | Unassigned |  |  | Acidobacteriaceae bacterium URHE0068, gi\|651320806, 31% | hypothetical protein |  |
| FS_2035 | - | Unassigned |  |  | Methanothermobacter marburgensis, gi\|503061404, 45% | tetratricopeptide repeat domain-containing protein |  |
| FS_2052 | - | Unassigned |  |  | Streptomyces aureofaciens, gi\|703119160, 34% | hypothetical protei |  |
| FS_2099 | - | Unassigned |  |  | Nitrosococcus oceani, gi\|672879806, 73% | hypothetical protei |  |
| FS_2185 | - | Unassigned |  |  | Thermococcus sp. ES1, gi\|573025152, 44% | Hypothetical protein |  |
| FS_2202 | - | Unassigned |  |  | Candidatus Methanoperedens nitroreducens, gi\|630829403, 43% | cytotoxic translational repressor of toxin-antitoxin stability system |  |
| FS_2315 | - | Unassigned |  |  | Algoriphagus machipongonensis, gi\|495473407, 40% | hypothetical protein |  |
| FS_2348 | - | Unassigned |  |  | uncultured Desulfobacterium sp., gi\|308270959, 47% | Probable formate transporter 1 |  |
| FS_2374 | - | Unassigned |  |  | Bacillus, gi\|489242753, 45% | DNA-binding protein |  |
| FS_2375 | - | Unassigned |  |  | Mesorhizobium, gi\|665828191, 46% | hypothetical protein |  |
| FS_2381 | - | Unassigned |  |  | Sphaerochaeta globosa, gi\|503373380, 34% | hypothetical protein |  |
| FS_2521 | - | Unassigned |  |  | Marinitoga piezophila, gi\|504062523, 58% | hypothetical protein |  |
| FS_2527 | - | Unassigned |  |  | Mycobacterium sp. 141, gi\|648665168, 40% | thioredoxin |  |
| FS_2533 | - | Unassigned |  |  | Amycolatopsis benzoatilytica, gi\|522147468, 58% | hypothetical protein |  |
| FS_2534 | - | Unassigned |  |  | Clostridium pasteurianum, gi\|505427925, 38% | putative cell wall binding protein |  |
| FS_2566 | - | [S] | Function unknown | COG5649 | Rhizobium mongolense, gi\|550966818, 75% | hypothetical protein |  |
| FS_2581 | - | Unassigned |  |  | uncultured marine thaumarchaeote KM3_15_E05, gi\|663512294, 42% | tripartite motif-containing protein 71 (TRIM71 |  |
| FS_2584 | - | Unassigned |  |  | Synechococcus sp. PCC 6312, gi\|504938356, 25% | RHS repeat-associated core domain-containing protein |  |
| FS_2598 | - | Unassigned |  |  | Ignavibacterium album, gi\|504373043, 35% | glucose/sorbosone dehydrogenase |  |
| FS_2603 | - | Unassigned |  |  | Thermoplasmatales archaeon E-plasma, gi\|546150982, 45% | hypothetical protein |  |
| FS_2605 | - | Unassigned |  |  | Parcubacteria bacterium RAAC4_OD1_1, gi\|564799671, 33% | hypothetical protein |  |
| FS_2610 | - | Unassigned |  |  | Microcoleus vaginatus, gi\|493684815, 41% | pentapeptide repeat protein |  |
| FS_0245 | - | Unassigned |  |  | Helcococcus sueciensis, gi\|653097469, 51% | mechanosensitive ion channel protein MscL |  |
| FS_0271 | - | Unassigned |  |  | Pseudomonas putida S610, gi\|558522688, 29% | hypothetical protein EDP1_4128 |  |
| FS_0273 | - | Unassigned |  |  | Ralstonia solanacearum, gi\|655479406, 62% | hypothetical protein |  |
| FS_0387 | - | [S] | Function unknown | COG3832 | Sphingobacterium sp. 21, gi\|503429575, 77% | ATPase |  |
| FS_0611 | - | Unassigned |  |  | Marine Group I thaumarchaeote SCGC AAA799-P11, gi\|675347103, 40% | hypothetical protein |  |
| FS_0616 | - | Unassigned |  |  | Arthrobacter sp. MA-N2, gi\|654812118, 51% | phosphodiesterase |  |
| FS_0713 | - | Unassigned |  |  | Candidatus Parvarchaeum acidophilus ARMAN-5, gi\|290559048, 32% | hypothetical protein |  |
| FS_0794 | - | Unassigned |  |  | Streptomyces viridochromogenes, gi\|490102377, 33% | putative Nonribosomal peptide synthetase |  |
| FS_0804 | - | Unassigned |  |  | Myxococcus stipitatus, gi\|505160040, 60% | transposas |  |
| FS_0856 | - | Unassigned |  |  | Desulfotomaculum alcoholivorax, gi\|653115375, 34% | UbiD family decarboxylase |  |
| FS_0857 | - | Unassigned |  |  | Acidiphilium multivorum, gi\|503406384, 72% | transposase |  |
| FS_0867 | - | Unassigned |  |  | Thermoplasmatales archaeon SCGC AB-540-F20, gi\|495879484, 34% | PQQ-like domain-containing protein |  |
| FS_0953 | - | Unassigned |  |  | Alcanivorax sp. 19-m-6, gi\|686989300, 31% | hypothetical protein |  |
| FS_1832 | - | Unassigned |  |  | Octadecabacter arcticus, gi\|505309711, 65% | hypothetical protein |  |
| FS_1069 | - | Unassigned |  |  | Alcanivorax sp. 19-m-6, gi\|686989300, 35% | hypothetical protein |  |
| FS_1268 | - | Unassigned |  |  | Methylobacterium sp. WSM2598, gi\|517074682, 57% | hypothetical protein |  |
| FS_1271 | - | Unassigned |  |  | Methyloferula ignalin, gi\|651600660, 51% | hemolysin D |  |
| FS_1394 | - | Unassigned |  |  | Nitrososphaera phage Pro-Nvie1, gi\|339272035, 45% | putative terminase, large subunit |  |
| FS_1537 | - | Unassigned |  |  | uncultured marine thaumarchaeote KM3_179_B04, gi\|663514566, 49% | hypothetical protein |  |
| FS_1548 | - | Unassigned |  |  | Desulfosporosinus youngiae, gi\|495062618, 52% | putative low-complexity protein |  |
| FS_1605 | - | Unassigned |  |  | Azospirillum sp. CAG:239, gi\|547786027, 51% | putative uncharacterized protein |  |
| FS_2456 | - | Unassigned |  |  | - | Membrane protein of unknown function |  |
| - | TS_0010 | Unassigned |  |  | uncultured marine thaumarchaeote SAT1000_44_H06, AIF25086, 58% | hypothetical protein |  |
| - | TS_0150 | Unassigned |  |  | Acidobacterium capsulatum, gi\|225872173, 32% | nitrate ABC transporter ATP-binding protein |  |
| - | TS_0162 | Unassigned |  |  | Desulfosporosinus sp. OT, EGW37019, 58% | GNAT family acetyltransferase |  |
| - | TS_0290 | Unassigned |  |  | Haloferax larsenii, ELZ84533, 34% | hypothetical protein |  |
| - | TS_0312 | Unassigned |  |  | Thermoplasmatales archaeon I-plasma, EQB65868, 47% | multidrug resistance protein |  |
| - | TS_0378 | Unassigned |  |  | uncultured bacterium, gi\|406994743, 33% | hypothetical protein |  |
| - | TS_0387 | Unassigned |  |  | Nitrosococcus oceani C-27, gi\|672875692, 47% | hypothetical protein |  |
| - | TS_0395 | Unassigned |  |  | Alicyclobacillus macrosporangiidus, gi\|657611444, 53% | GCN5 family acetyltransferase |  |
| - | TS_0408 | Unassigned |  |  | Thermoplasmatales archaeon I-plasma, EQB65868, 47% | multidrug resistance protein |  |
| - | TS_0551 | Unassigned |  |  | Caulobacter crescentus CB15, gi\|221233261, 22% | hypothetical protein |  |
| - | TS_0552 | Unassigned |  |  | Pelosinus, EIW17851, 46% | hypothetical protein |  |
| - | TS_0571 | Unassigned |  |  | Rhodopirellula sallentina, EMI51897, 30% | hypothetical protein |  |
| - | TS_0582 | Unassigned |  |  | [Burkholderia pyrrocinia, gi\|674793949, 47% | peptidase S8/S53 subtilisin kexin sedolisin |  |
| - | TS_0583 | Unassigned |  |  | Aciduliprofundum boonei, EDY35511, 51% | peptidase S53 |  |
| - | TS_0585 | Unassigned |  |  | Aciduliprofundum sp. MAR08-339, gi\|432328632, 58% | putative protease |  |
| - | TS_0691 | Unassigned |  |  | candidate division YNPFFA, gi\|516977387, 43% | hypothetical protei |  |
| - | TS_0864 | Unassigned |  |  | Sulfolobales archaeon AZ1, gi\|583846877, 28% | hypothetical protein |  |
| - | TS_0946 | Unassigned |  |  | Oscillochloris trichoides, EFO79114.1, 36% | cytochrome C biogenesis protein |  |
| - | TS_1035 | Unassigned |  |  | Burkholderia pyrrocinia, gi\|51590238, 44% | peptidase S8/S53 subtilisin kexin sedolisin |  |
| - | TS_1174 | Unassigned |  |  | Butyrivibrio sp. AE3004, gi\|657209607, 45% | hypothetical protein |  |
| - | TS_1181 | Unassigned |  |  | Ferroplasma sp. Type II, EQB72915, 39% | hypothetical protein |  |
| - | TS_1264 | Unassigned |  |  | Prolixibacter bellariivorans, gi\|653563185, 25% | hypothetical protein |  |
| - | TS_1265 | Unassigned |  |  | Mycobacterium smegmatis, gi\|433647791, 34% | lysyl-Trna synthetase (class II) |  |
| - | TS_1364 | Unassigned |  |  | Desulfococcus multivorans, gb\|EPR43038, 29% | Base plate protein |  |
| - | TS_1370 | Unassigned |  |  | Ophiocordyceps sinensis CO18, gi\|531864340, 30% | virulence-associated lipoprotein |  |
| - | TS_1375 | Unassigned |  |  | Streptomyces afghaniensis, gb\|EPJ37174, 30% | hypothetical protein |  |
| - | TS_1379 | Unassigned |  |  | Cylindrospermum stagnale, gi\|434405340, 41% | hypothetical protei |  |
| - | TS_1384 | Unassigned |  |  | Paracoccus versutus, gi\|694206387, 34% | hypothetical protei |  |
| - | TS_1385 | Unassigned |  |  | Desulfococcus multivorans, EPR43046, 46% | hypothetical protein |  |
| - | TS_1386 | Unassigned |  |  | Desulfococcus multivorans, EPR43047, 31% | hypothetical protein |  |
| - | TS_1388 | Unassigned |  |  | uncultured archaeon, gi\|268325569, 66% | conserved hypothetical protein containing PAAR motifs |  |
| - | TS_1389 | Unassigned |  |  | Streptomyces, gi\|662101538, 43% | hypothetical protein |  |
| - | TS_1392 | Unassigned |  |  | Rivularia sp. PCC 7116, gi\|427738595, 28% | hypothetical protein |  |
| - | TS_1394 | Unassigned |  |  | Methanobacterium formicicum, EKF85749, 51% | putative glycosyltransferase |  |
| - | TS_1398 | Unassigned |  |  | Cyanothece sp. PCC 7822, gi\|307592357, 29% | YD repeat-containing protein |  |
| - | TS_1406 | Unassigned |  |  | Sulfolobales archaeon Acd1, gi\|519043902, 37% | hypothetical protein |  |
| - | TS_1422 | Unassigned |  |  | Sulfolobus acidocaldarius, gi\|568164343, 37% | DNA-binding protein |  |
| - | TS_1423 | Unassigned |  |  | Hyperthermus butylicus, gi\|124028348, 49% | hypothetical protein |  |
| - | TS_1561 | Unassigned |  |  | uncultured marine thaumarchaeote KM3_67_B07, AIF14357, 87% | DNA topoisomerase VI subunit A (top6A) |  |
| - | TS_1609 | Unassigned |  |  | Methanocaldococcus sp. FS406-22, gi\|289192965, 34% | hypothetical protein |  |
| - | TS_1776 | Unassigned |  |  | Bacillus cereus, EJQ71287, 58% | hypothetical protein |  |
| - | TS_1777 | Unassigned |  |  | Bacillus gaemokensis, gi\|727216303, 51% | 40-residue YVTN family beta-propeller, partia |  |
| - | TS_1977 | Unassigned |  |  | Methanoplanus petrolearius, gi\|307352284, 53% | heat shock protein HtpX |  |
| - | TS_2322 | Unassigned |  |  | Haloferax, ELZ75351, 35% | transcription regulato |  |
| - | TS_2002 | Unassigned |  |  | Streptomyces sp. FxanaC1, gi\|648477857, 43% | hypothetical protein |  |
| - | TS_2007 | Unassigned |  |  | Ferroplasma sp. Type II, EQB73621, 34% | transferase |  |
| - | TS_2041 | Unassigned |  |  | Aciduliprofundum sp. MAR08-339, gi\|432328632, 58% | putative protease |  |
| - | TS_2080 | Unassigned |  |  | Sinorhizobium meliloti, gi\|433615256, 42% | hypothetical protein |  |
| - | TS_2213 | Unassigned |  |  | Candidatus Parvarchaeum acidiphilum ARMAN-4, EGD71829, 47% | hypothetical protein |  |
| - | TS_2247 | Unassigned |  |  | Marine Group I thaumarchaeote SCGC AAA799-O18, KFM15242, 56% | hypothetical protein |  |
| - | TS_2278 | Unassigned |  |  | Desulfovibrio sp. U5L, EIG54785, 34% | PAS domain S-box |  |
| - | TS_2286 | Unassigned |  |  | uncultured organism, gi\|452077386, 27% | nucleic acid binding protein |  |
| - | TS_2287 | Unassigned |  |  | uncultured organism, gi\|452077385, 60% | protein belonging to Uncharacterized protein family |  |
| - | TS_2326 | Unassigned |  |  | Desulfobulbus propionicus, gi\|320354510, 29% | multi-sensor hybrid histidine kinase |  |
| - | TS_2347,TS_2330 | [K] | Transcription | COG3905 | Pectobacterium carotovorum, KFX02072, 64% | CopG family transcripitonal regulator |  |
| - | TS_2331,TS_2348 | [S] | Function unknown | COG3668 | Ferrovum myxofaciens, gi\|671638165, 54% | plasmid stabilization protein |  |
| - | TS_2332,TS_2349 | [O] | Posttranslational modification, protein turnover, chaperones | COG0526 | Geopsychrobacter electrodiphilus, gi\|522166820, 77% | thioredoxin |  |
| - | TS_2341 | Unassigned |  |  | Wuchereria bancrofti, EJW81824, 58% | plasmid encoded RepA protein |  |
| - | TS_2343 | Unassigned |  |  | Thiomonas sp. CB2, gi\|668347182, 53% | putative ParA-like protei |  |
| - | TS_2346 | Unassigned |  |  | Schlesneria paludicola, gi\|498272295, 53% | resolvase |  |
| - | TS_2353 | Unassigned |  |  | Zooshikella ganghwensis, gi\|654069122, 58% | hypothetical protein |  |

*Best non-*Nitrosotalea* hit. Genes in red were proposed to be involved in acidophily. The yellow highlighted genes were consistent with the ‘*Ca.* N. devanaterra-specific genes proposed to be involved in acidophily’ by Herbold et al*.* (17) and Lehtovirta-Morley et al*.* (50).

**Table S10. Transporters in the FS and TS genomes.**

| **Gene** | **Product** | **FS** | **TS** | **PFAM** | **Explanation** | **Presence in neutrophilic thaumarchaeotal genomes** |
| --- | --- | --- | --- | --- | --- | --- |
| **1.A.1. Voltage-gated ion channel (VIC) superfamily** | | | | | | |
|  | putative ion channel | _ | TS_0300 | PF00520 | Members of the VIC Superfamily can be voltage insensitive. Most of these proteins are K^+^, Na^+^ or Ca^2+^ channels. | SCM1-1, AR2-1, AR1-1, Ngar-1 |
| **1.A.8. Major intrinsic protein (MIP) family** | | | | | | |
|  | major intrinsic protein | FS_1762, FS_0604, FS_1314, FS_1496 | TS_0888, TS_1235 | PF00230 | Channels in the MIP family transport water, organics (e.g. glycerol), urea, NH_3_, CO_2_, H_2_O_2_ and ions. | BG20-2, SBF1-2, MY1-2, SCM1-2, AR2-2, BD31-1, AR1-2, CENSYa-2, N4-2, Ngar-2, SR1-3 |
| **1.A.11. Ammonia channel transporter (Amt) family** | | | | | | |
| amt1 | ammonia channel | FS_0042 | TS_1674 | PF00909 | Channels in the Amt family transport NH_3_, NH_4_^+^,CO_2_, methylammonium | BG20-1, SBF1-1, MY1-1, SCM1-1, AR2-1, BD31-1, AR1-1, CENSYa-1, N4-1, Ngar-1, SR1-2 |
| amt2 | ammonia channel | FS_0760, FS_1983 | **_** |  |  | BG20-1, SBF1-1, MY1-1, SCM1-1, AR2-1, BD31-1, AR1-1, N4-1, Ngar-1, SR1-1 |
| **1.A.13. The Epithelial Chloride Channel (E-ClC) Family** | | | | | | |
|  | Chloride channel protein | _ | _ | PF00092 | Channels in the E-ClC family transport Cl^-^ | BG20-1, SCM1-1, AR2-1, AR1-1, CENSYa-1, SR1-1 |
| **1.A.16. The Formate-Nitrite Transporter (FNT) Family** | | | | | | |
|  | Formate/nitrite transporter | FS_2348 | _ | PF01226 | Proteins of the FNT family probably function in the transport of the structurally related compounds, formate and nitrite. | Ngar-1 |
| **1.A.22. Large Conductance Mechanosensitive Ion Channel (MscL) Family** | | | | | | |
| mscL | large-conductance mechanosensitive ion channel | FS_0245 | TS_1338 | PF01741 | The MscL channel of *E. coli* favor the non specific efflux of ions in reponse to hypo-osmotic shock. This transporter has a preference for cations over anions. | BG20-1, SBF1-1, MY1-1, Ngar-1, SR1-1 |
| **1.A.23. Small Conductance Mechanosensitive Ion Channel (MscS) Family** | | | | | | |
| mscS2 | putative small-conductance mechanosensitive ion channel | _ | - | PF00924 | The MscS channel of E. coli favor the efflux of ions in reponse to hypo-osmotic shock. | BG20-1, SFB1-1, MY1-1, SCM1-4, AR2-4, BD31-2, AR1-3, N4-1, Ngar-2, SR1-3 |
| **1.A.28. Urea Transporter (UT) Family** | | | | | | |
|  | putative urea transporter | _ | TS_1744 | PF03253 | Channels of the UT family facilitate the transport of urea in mammals and bacteria. Up to now, this family of transporter wasn’t known in archaea. | Ngar-1, SR1-1 |
| **1.A.35. CorA Metal Ion Transporter (MIT) Family** | | | | | | |
|  | putative CorA ion transporter | _ | _ | PF01544 | Characterized transporters of the MIT family favor the uptake and efflux of divalent cations (e.g. Mg^2+^, Co^2+^, Ni^2+^, Zn^2+^, Cd^2+^). | BG20-2, SFB1-2, MY1-1, SCM1-2, AR2-2, BD31-1, AR1-2, CENSYa-1, N4-1, Ngar-2, SR1-2 |
| **2.A.1.2. The drug:H^+^ antiporter (12 spanner) (DHA1) family** | | | | | | |
|  | major facilitator superfamily transporter | FS_0577 | _ | SSF103473 | Similarity searches suggest this protein belong to the Drug:H+ Antiporter-1 (12 Spanner) (DHA2) Family (2.A.1.2). This family is involved in the efflux of quinidine, and is important in maintaining physiological levels of K^+^ in the cell under K^+^-limited growth. | BG20-2, SFB1-1, MY1-3, SCM1-1, AR2-1, BD31-1, AR1-2, CENSYa-2, N4-1, Ngar-1, SR1-2 |
| **2.A.1.3. The drug:H^+^ antiporter (14 spanner) (DHA2) family** | | | | | | |
|  | major facilitator superfamily transporter | FS_0417, FS_2358, FS_2358, FS_1159* | TS_0312, TS_0421, TS_0874, TS_1229 | SSF103473 | Similarity searches suggest this protein belong to the Drug:H+ Antiporter-2 (14 Spanner) (DHA2) Family (2.A.1.3). This family is involved in the efflux of antibiotics but also in the efflux of siderophores and ignaling compounds and in the uptake of basic amino acids and nucleobases for example. | BG20-1, SFB1-1, MY1-1, BD31-1, Ngar-2, SR1-2 |
| **2.A.1.30. The Putative Abietane Diterpenoid Transporter (ADT) Family** | | | | | | |
| DitE | Putative abietane uptake permease | FS_0194 | TS_1192 | SSF103473 | Putative abietane uptake permease (in gene cluster for degradation of abietane diterpenoids). | - |
| **2.A.1.59. unidentified major facilitator-10 (UMF10) family** | | | | | | |
|  | MFS permease | _ | _ | SSF103473 | Unidentified Major Facilitator-10 (UMF10) Family (mostly from Archaea but some from bacteria) | Ngar-1 |
| **2.A.1.63. The unidentified major Facilitator-12 (UMF12) family** | | | | | | |
|  | MFS carrier | FS_2595 | TS_1176 | SSF103473 | The Unidentified Major Facilitator-12 (UMF12) Family | BG20-2, SFB1-1, BD31-1, N4-1, SR1-1 |
| **2.A.3. The amino acid-polyamine-organoca on (APC) superfamily** | | | | | | |
|  | Putative amino acid transporter | FS_0517 | TS_0660, TS_0805 | PF13520 | Transporters of the APC family are involved in the transport of various amino acids, polyamine (e.g. putrescine, spermidine, cadaverine) but also ethanolamine, methylamine, thiamine or choline | BG20-2, SFB1-4,MY1-1, SCM1-2, AR2-2, BD31-2, AR1-2, CENSYa-1, N4-4, Ngar-4, SR-6 |
| **2.A.4. The cation diffusion facilitator (CDF) family** | | | | | | |
|  | cation diffusion facilitator family transporter | FS_1583 | TS_1872,TS_1915 | PF01545 | Transporters of the CDF family are involved in the efflux of Zn2+, Co2+, Cd2+, Fe2+ and Hg2+ and in the transport of Cu2+, Ni2+ and Mn2+. | BG20-2, BFG1-2, MY1-2, SCM1-3, AR2-1, BD31-4, AR1-1, N4-1, Ngar-4, SR1-5 |
| **2.A.5. The zinc (Zn^2+^)-iron (Fe^2+^) permease (ZIP) family** | | | | | | |
|  | putative metal cation transporter | FS_1971 | TS_0401 | PF02535 | Transporters of the ZIP family are involved in the transport of divalent cations, mainly Zn^2+^ and Fe^2+^ but also Co^2+^, Mn^2+^, Cd^2+^, Pb^2+^, Hg^2+^. | SFB1-1, MY1-1, SCM1-1, AR2-1, AR1-1, SR1-1 |
| **2.A.7. The drug/Metabolite transporter (DMT) superfamily** | | | | | | |
|  | drug/metabolite transporter superfamily protein | FS_0304, FS_0846, FS_1476, FS_2195 | TS_0826, TS_1403, TS_1514, TS_1912, TS_1852 | PF00892 | Transporter of the DMT Superfamily are involved in the uptake/efflux of various drugs and metabolites. | BG20-4, SFB1-4, MY1-5, SCM1-5, AR2-3, BD31-4, AR1-2, CENSYa-3, N4-4, Ngar-3, SR1-3 |
| **2.A.19.2 The Ca^2+^ : cation antiporter (CaCA) family** | | | | | | |
|  | calcium/proton antiporter | _ | TS_0832 | PF01699 | Similarity searches suggest this protein belong to the transporter cluster 2.A.19.2. Protein in this cluster are Ca2+/heavy metals (Mn2+, Zn2+, Cd2+, Mg2+, Co2+) :H+ exchangers. | Ngar-1 |
| **2.A.19.5 The Ca^2+^ : cation antiporter (CaCA) family** | | | | | | |
|  | calcium/cation antiporter family protein | FS_0430 | _ | PF01699 | Similarity searches suggest this proteinbelong to the transporter cluster 2.A.19.5 of Na+:Ca2+ exchanger. | BG20-1, SFB1-1, SCM1-1, AR2-1, AR1-1, CENSYa-1, N4-1, Ngar-1, SR1-1 |
| **2.A.20. The inorganic phosphate transporter (Pit) family** | | | | | | |
|  | phosphate transporter protein | FS_1909 | TS_0058 | PF01384 | Genes in N. limnea and N. koreensis are in the size range of PIT transporter (354-681 aa) and are probably functional. | BG20-1, SFB1-1, MY1-1, AR2-1, AR1-1, N4-1, Ngar-1, SR1-1 |
| **2.A.21.4.1. The H^+^/solute symporter** | | | | | | |
|  | The H+/solute symporter | FS_1852 | TS_0186 | PF00474 | The monocarboxylate uptake (H+ symport?) permease, MctP (transports lactate (Km = 4.4 Μm), pyruvate (Km = 3.8), propionate, butyrate (butanoic acid), α-hydroxybutyrate, L- and D-alanine (Km = 0.5 Mm), and possibly cysteine and histidine) | - |
| **2.A.21.6.3 Urea active transporter** | | | | | | |
|  | Urea active transporter | _ | _ | PF00474 | Similarity searches suggest this protein beolong to the transporter cluster 2.A.21.6 of urea, polyamines and antimicrobial peptide uptake systems. | AR2-1, CENSYa-1, Ngar-1, SR1-1 |
| **2.A.21.8. Sodium : solute symporter,** | | | | | | |
|  | The Sodium : solute symporter | _ | _ | PF00474 | High affinity neuronal choline:Na+ symporter, CHT1 (chloride-dependent). |  |
| **2.A.36. Na^+^/H^+^ antiporter, The Monovalent Cation:Proton Antiporter-1 (CPA1) Family** | | | | | | |
|  | Na+/H+ antiporter | FS_2537, FS_2316 | TS_0377, TS_1353 | PF00999 | All members of CPA1 catalyze Na^+^:H^+^ exchange. Their primary physiological functions may be in (1) cytoplasmic Ph regulation, extruding the H^+^ generated during metabolism, and (2) salt tolerance (in plants), due to Na+ uptake into vacuoles. Bacterial homologues are also Na^+^:H^+^ antiporters, but some also catalyze Li^+^:H^+^ antiport or Ca^2+^:H^+^ antiport under some conditions. | BG20-1, SFB1-1, SCM1-1, AR2-1, BD31-1, AR1-1, CENYa-1 |
| **2.A.37. The Monovalent Cation:Proton Antiporter-2 (CPA2) Family** | | | | | | |
|  | K+/H+ antiport | _ | TS_0562 | PF00999 | The CPA2 family is a moderately large from bacteria, archaea and eukaryotes. Among the functionally well-characterized members of the family are (1) the KefB/KefC K+ efflux proteins of E. coli which may be capable of catalyzing both K+/H+ antiport and K+ uniport, depending on conditions, (2) the Na+/H+ antiporter of Enterococcus hirae and (3) the K+/H+ antiporter of S. cerevisiae. It has been proposed that under normal physiological conditions, these proteins may function by essentially the same mechanism | BG20-4, SFB1-3, MY1-5, SCM1-3, AR2-4, BD31-3, AR1-4, CENYa-2, N4-3, Ngar-9 |
| **2.A.38. The K^+^ Transporter (Trk) Family** | | | | | | |
|  | K+ Transporter | FS_2512 | _ | PF02386 | Similarity searches suggest this protein belong to the transporter cluster 2.A.38 of K+ uptake transporters. | BG20-3, SFB1-4, MY1-1, SCM1-3, AR2-3, BD31-4, AR1-2, N4-4, Ngar-4, SR1-2 |
| **2.A.39. The nucleobase:Cation Symporter-1 (NCS1) Family** | | | | | | |
| cytX | hydroxymethylpyrimidine transporter | _ | _ | PF02133 | Similarity searches suggest this protein beolong to the transporter cluster 2.A.39.1. | Ngar-1, SR1-2 |
| **2.A.45. The arsenite-antimonite (ArsB) Efflux family.** | | | | | | |
|  | Arsenite-Antimonite efflux pumps | _ | TS_1637 | PF03600 | These pumps actively expel both arsenite and antimonite | BG20-1, MY1-1, N4-1, Ngar-1, SR1-2 |
| **2.A.50. The Glycerol Uptake (GUP) Family** | | | | | | |
|  | Glycerol uptake porter | FS_0214 | _ | PF03062 | glycerol has been reported to be actively taken up via two electrogenic H+ symporters | BD31-1 |
| **2.A.52. Ni^2+^ - Co^2+^ Transporter (NiCoT) Family** | | | | | | |
|  | High affinity nickel transport protein | _ | TS_0362/0219, TS_0362/0219, TS_0338/0208, TS_0943 | PF03824 | Transporters of the NiCoT family catalyse the uptake of Ni2+ and Co2+ but also the efflux of Ca2+ and Ni2+. Some of these transporters are involved in the incorporation of nickel in urease enzymes. | SR1-1 |
| **2.A.53. The Sulfate Permease (SulP) Family** | | | | | | |
|  | Sulfate Permease | _ | _ | PF00581 | Many function by SO42-:H+ symport, but SO42-:HCO3-, or more generally, anion:anion antiport has been reported for several homologues | SFB1-1, MY1-1, SCM1-1, N4-1 |
| **2.A.55. The Metal Ion (Mn^2+^-iron) Transporter (Nramp) Family** | | | | | | |
|  | Mn^2+^/Fe^2+^ transporter | FS_1307 | TS_0954 | PF07690 | The members of Nramp family transport several heavy metals including Mn^2+^, Cu^2+^, Cd^2+^ and Co^2+^ | SCM1-1, AR2-1, BD31-1, AR1-1, N4-1, SR1-1 |
| **2.A.59. Arsenical Resistance-3 (ACR3) Family** | | | | | | |
|  | arsenical resistance-3 family protein | _ | TS_0516 | PF01758 | Only two representatives of the ACR3 family have been characterized and shown to be arsenite/antimonite efflux systems. This family includes several clusters of phylogenetically distinct protein which have not yet been characterized. | N4-1 |
| **2.A.66 Multidrug/Oligosaccharidyl-lipid/Polysaccharide (MOP) Flippase Superfamily** | | | | | | |
|  | putative polysaccharide transporter | FS_1093 | _ | PF13440 | Similarity searches suggest this protein belong to the Polysaccharide Transport (PST) Family 2.A.66. | CENYa-1, Ngar-1 |
| **2.A.83. The Na^+^-dependent bicarbonate transporter (SBT) family** | | | | | | |
|  | Na+-dependent Bicarbonate Transporter | _ | _ | PF05982 | Na+-dependent bicarbonate (HCO3-) uptake system in Cyanobacteria that is induced when the CO2 concentration is low. | BG20-1, SFB1-2, SCM1-1, AR2-1, AR1-1, N4-1 |
| **2.A.89. Vacuolar Iron Transporter (VIT) Family** | | | | | | |
|  | vacuolar iron transporter family protein | FS_0358 | _ | PF01988 | Transporters of the VIT family are involved in the transport of Fe2+ and Mn2+. | BG20-1, SFB1-1, MY1-1, AR2-1, BD31-3, AR1-1, N4-1, Ngar-1, SR1-2 |
| **2.A.102. The putative 4-toluene sulfonate uptake permease (TSUP) family** | | | | | | |
|  | putative sulfonate transporter | FS_2273 | TS_0461 | PF01925 | Transporters of the TSUP family are involved in the uptake and efflux of organo-sulfur compounds such as sulfonates but also sulfite and sulfate. | BG20-1, SFB1-1, MY1-1, SCM1-1, AR2-1, BD31-1, AR1-2, N4-1, Ngar-2, SR1-1 |
| **2.A.108. The iron/Lead transporter (ILT) family** | | | | | | |
|  | iron/Lead transporter | FS_0439, FS_0529 | TS_0399, TS_2255 | PF03239 | The iron/Lead transporter catalyze the uptake of iron and lead ions. | BG20-4, SFB1-5, MY1-3, SCM1-4, AR2-7, BD31-3, AR1-6, CENSYa-1, N4-3, Ngar-9, SR1-7 |
| **2.A.109. The tellurium Ion tesistance (TerC) family** | | | | | | |
|  | Tellurium Ion transporter | _ | _ | PF03741 | The TerC family (Pfam 03741) includes the E. coli TerC protein which has been implicated in tellurium resistance. It is hypothesized to catalyze efflux of tellurium ions | BG20-1, SFB1-1, MY1-1, BD31-1, N4-1, Ngar-3, SR1-2 |
| **2.A.113. The nickel/cobalt transporter (NicO) family** | | | | | | |
|  | nickel/cobalt transporter | _ | _ | PF03824 | The members of the nickel/cobalt transporter (NicO) family catalyze efflux of tellurium ions | BG20-1, SFB1-1, MY1-1, SCM1-1, AR2-1. BD31-1, N4-1, Ngar-1 |
| **3.A.1.5. The peptide/Opine/Nickel uptake transporter (PepT) family.** | | | | | | |
|  | Peptide/opine/nickel ABC uptake transporter, permease protein | FS_0483,FS_1165, FS_1923,FS_1169, FS_2574, FS_2755 | TS_0060, TS_0062, TS_0785, TS_0787, TS_0788, TS_0789, TS0964, TS_1707 | PF00528 | Similarity searches indicate that this transport system belongs to the Peptide/Opine/Nickel Uptake Transporter (PepT) Family (3.A.1.5). This family of transporters also include sugar, glutathione, EDTA, and antibiotic uptake systems. | BG20-4, SFB1-5, MY1-5, SCM1-5, AR2-5, BD31-5, AR1-5, CENSYa-5, N4-5, Ngar-6, SR1-5 |
| **3.A.1.7. The phosphate uptake transporter (PhoT) family.** | | | | | | |
|  | ABC uptake transporter | FS_1913, FS_1305, FS_2408, FS_2410, FS_2411, FS_2412, FS_2413 | TS_1218, TS_1220, TS_1223, TS_1224 | PF00528 | Phosphate porter, PhoSPstABC. Serves as both a transporter and a sensor for transcriptional activation of the pho regulon in the presence of low external phosphate. The unphosphorylated EIIA^Ntr^ protein of the PTS (TC# 4.A) activates PhoR, the senor kinase that phosphorylates the response regulator, PhoB, that activates the phoregulon. | SFB1-4, MY1-4, SCM1-4, CENSYa-4, N4-5, Ngar-8, SR1-5 |
| **3.A.1.9. The phosphonate uptake transporter (PhnT) family** | | | | | | |
|  | ABC efflux transporter, ester porter | _ | _ | PF00528 | Phosphonate/organophosphate ester porter (broad specificity). | SCM1-3, AR2-2, CENSYa-2, N4-3 |
| **3.A.1.15. The manganese/Zinc/Iron chelate uptake transporter (MZT) family.** | | | | | | |
|  | ABC uptake transporter, substrate-binding/permease protein | FS_2673, FS_2674, FS_2675, FS_2676, FS_0500,FS_0501, FS_0502 | TS_1109, TS_1110, TS_1111 | PF00950 | Similarity searches suggest this transport system belongs to the Manganese/Zinc/Iron Chelate Uptake Transporter (MZT) Family (3.A.1.15). This family of transporter is involved in the uptake of Mn2+, Zn2+, Fe2+ and, to a lower extent, Cu2+. | BG20-5, SFB1-3, MY1-3, SCM1-9, AR2-3, BD31-3, AR1-4, CENSYa-3, N4-4, Ngar-9, SR1-3 |
| **3.A.1.17. The taurine uptake transporter ( TauT) family** | | | | | | |
|  | ABC uptake transporter, substrate-binding protein | FS_0542, FS_0544 | TS_1314 | PF00528 | The Taurine Uptake Transporter (TauT) Family (3.A.1.17). | BG20-2, SFB1-2, MY1-2, SCM1-2, AR2-1, BD31-1, AR1-2, CENSYa-2, N4-2, Ngar-3, SR1-4 |
| **3.A.1.105. The drug exporter-1 (DrugE1) family** | | | | | | |
|  | ABC efflux transporter, permease protein | FS_1317, FS_1318 | TS_0310, TS_0311, TS_0408, TS_0409, TS_1443 | PF01061 | Similarity searches suggest these two transport systems belong to the Drug Exporter-1 (DrugE1) Family 3.A.1.105 which mainly includes drug/antibiotic efflux systems. | BG20-3, SFB1-3, MY1-3, SCM1-3, AR2-2, AR1-2, CENSYa-1, N4-4, Ngar-2, SR1-5 |
| **3.A.1.122. The macrolide exporter (MacB) family** | | | | | | |
|  | ABC efflux transporter, ATP-binding protein | FS_2708, FS_1436, FS_1891, FS_1893 | TS_0304, TS_2093, TS_0306 , TS_1108, TS_1144, TS_1145, TS_2093 | PF02687 | Similarity searches suggest this transport system belongs to the Macrolide Exporter (MacB) Family 3.A.1.122 which includes macrolides, enterotoxin, antimicrobial peptide and heme efflux systems. | BG20-2, SFB1-2, MY1-2, SCM1-1, AR2-2, BD31-2, AR1-1, CENSYa-2, N4-1, Ngar-4, SR1-5 |
| **3.A.3. The P-type ATPase (P- ATPase) superfamily (3.A.3.5. family)** | | | | | | |
| CopA | Copper-transporting ATPase | FS_1718, FS_2113 | TS_0660, TS_2072 | PF00122 | The copper resistance ATPase protein, CopA of *Bacillus subtilis*. | BG20-3. SFB1-1, AR2-1, AR1-1, N4-1, Ngar-3, SR1-6 |
| **3.A.3. The P-type ATPase (P- ATPase) superfamily (3.A.3.7. family)** | | | | | | |
| KdpABC | Potassium-transporting ATPase | FS_1588, FS_1590, FS_1001 | _ | PF00122 | High affinity potassium uptake ATPase, KdpABC. Regulated by direct interaction of the IIA^Ntr^ protein with the sensor kinase/response regulator, KdpDE. | SR1-2 |
| **3.A.3. The P-type ATPase (P- ATPase) superfamily (3.A.3.25. family)** | | | | | | |
|  | Cadmium-transporting ATPase | _ | _ | PF00122 | Functionally uncharacterized P-type ATPase family 25. | BG20-2, SFB1-1 |
| **3.A.10. The H^+^, Na^+^-translocating pyrophosphatase (M^+^-Ppase) Family** | | | | | | |
|  | proton-translocating pyrophosphatase family protein | _ | TS_0114 | PF03030 | Transporters of the H^+^-Ppase Superfamily are involved in the translocation of H^+^, Na^+^ and K^+^ ions. | BG20-1, SFB1-1, MY1-1, SCM1-1, AR2-1, BD31-1, AR1-1, CENSYa-1, N4-1, Ngar-1 |
| **9.A.40. HlyC/CorC (HCC) Family of Putative Transporters** | | | | | | |
|  | HlyC/CorC-like protein | FS_0893 | _ | PF03471 | Proteins of the HCC family include hemolysins and homologues of the Co^2+^-resistance protein CorC of *Salmonella typhimurium*. CorC is thought to be an auxiliary protein of the CorA channel (Metal Ion Transporter (MIT) Family: 1.A.35). CorC might enable CorA to function as a Co^2+^ efflux system. *N. gargensis* encodes 2 homologues of CorA. | BG20-1, SFB1-1, MY1-1, SCM1-2, AR2-2, BD31-1, AR1-1, CENSYa-1, N4-1, Ngar-1 |
| **9.A.58. The sweet; PQ-loop; Saliva; MtN3 (sweet) family** | | | | | | |
|  | putative magnesium transporter-C family protein | _ | _ | PF03083/PF04193 | The transport function of proteins of the MgtC family has not yet been established. Experimental work with *Salmonella typhimurium* and *Mycobacterium tuberculosis* suggests a function in Mg^2+^ active transport. | BG20-1, SFB1-1, MY1-1, SCM1-1, AR2-1, BD31-1, N4-1, Ngar-2, SR1-1 |
| **9.B.20. Putative Mg2+ Transporter-C (MgtC) Family** | | | | | | |
|  | putative magnesium transporter-C family protein | _ | _ | PF02308 | The transport function of proteins of the MgtC family has not yet been established. Experimental work with *Salmonella typhimurium* and *Mycobacterium tuberculosis* suggests a function in Mg^2+^ active transport. | BG20-1, SFB1-1, AR2-1, N4-1, Ngar-1, SR1-2 |
| **9.B.27. DedA or YdjX-Z (DedA) Family** | | | | | | |
|  | DedA family protein | FS_2165, FS_0199 | TS_0145, TS_1350 | PF09335 | Protein of the DedA family might be involved in the transport of selenite in bacteria and some fungal homologues might be involved in oxalate efflux. Protein of this family are also related to the SNARE-associated Golgi proteins. The three genes in *N. gargensis* are only distantly related to each other (id <30%) and only the RNAR_00489 gene shows clear sequence similarity with DedA family proteins in the Transporter Classification Database. | BG20-1, SFB1-1, MY1-1, SCM1-1, AR2-1, BD31-1, AR1-2, CENSYa-1, N4-1, Ngar-1, SR1-2 |
| **9.B.45. Arg/Asp/Asp (RDD) Family** | | | | | | |
|  | **RDD family protein** | **_** | **_** | **PF06271** | The transport function and putative ligands of the RDD family has not yet been establsihed. | SFB1-1, MY1-1, AR2-1, Ngar-2, SR1-2 |
| **9.B.62. Copper Resistance (CopD) Family** | | | | | | |
|  | copper resistance (CopD) family protein | _ | _ | PF04234/PF05425 | This protein contain both the CopC and CopD domains of Copper resistance proteins as well as an SMP-30/Gluconolactonase/LRE-like domain in the long (522aa) extracellular C-terminal region. The mechanism of copper resistance is not known. | MY1-1 |

Transporter families were classified according the the transporter classification database (TCDB) (85), while the corresponding protein families are identified by matching PFAM (86) and SUPERFAMILY (87) domains. Yellow represents the pores and channels, green represents electrochemical-potential-driven transporters, orange reprents primary active transporters, pink represents incompletely characterized transport systems. BG20, *Ca.* Nitrosarchaeum limnium BG20; SFB1, *Ca.* Nitrosarchaeum limnium SFB1; MY1, *Nitrosarchaeum koreense* MY1; SCM1, *Nitrosopumilus maritimus* SCM1; AR2, *Ca.* Nitrosopumilus sediminis AR2; BD31, *Ca.* Nitrosopumilus salaria BD31; AR1, *Ca.* Nitrosopumilus koreense AR1; CENSYa, *Cenarchaeum symbiosum* A; N4, *Ca.* Nitrosotenuis uzonensis N4; Ngar, *Ca.* Nitrososphaera gargensis Ga9.2; SR1, *Ca.* Nitrososphaera evergladensis SR1. *Genes in red indicate the genes propsed to be related with acidic adaptation.

**Table S11. Genes encoding proteins of complex I, II, III, IV and V in the respiration chain of the FS and TS genomes.**

| **Gene** | **Product** | **EC no.** | **FS Locus** | **Best BLAST hit in NCBI nr (% identity)*** | | **TS locus** | | **Best BLAST hit in NCBI nr (% identity)*** | | **Comment** |
| --- | --- | --- | --- | --- | --- | --- | --- | --- | --- | --- |
| **11 subunit version of Complex I: type I NADH dehydrogenase (nuoEFG absent)** | | | | | | | | | | |
| nuoA | NADH-quinone oxidoreductase, subunit A | EC 1.6.99.5 | FS_1741 | Nitrosopumilus maritimus, ABX12172, 74% | | | TS_1732 | Nitrosarchaeum koreensis, EGP93005, 76% | |  |
| nuoB | NADH-quinone oxidoreductase, subunit B | EC 1.6.99.5 | FS_1740 | Nitrosopumilus maritimus, ABX12173, 86% | | | TS_1731 | Nitrosarchaeum koreensis, EGP93006, 69% | |  |
| nuoC | NADH-quinone oxidoreductase, subunit C | EC 1.6.99.5 | FS_1739 | Thaumarchaeota archaeon N4, CDI06270, 73% | | | TS_1730 | Thaumarchaeota archaeon N4, CDI06270, 74% | |  |
| nuoD | NADH-quinone oxidoreductase, subunit D | EC 1.6.99.5 | FS_1738 | Thaumarchaeota archaeon N4, CDI06269, 82% | | | TS_1729 | Thaumarchaeota archaeon N4, CDI06269, 84% | |  |
| nuoH | NADH-quinone oxidoreductase, subunit H | EC 1.6.99.5 | FS_1737 | Nitrosopumilus koreensis AR1, gi\|407045406, 80% | | | TS_1728 | Nitrosopumilus koreensis, AFS80159, 68% | |  |
| nuoI | NADH-quinone oxidoreductase, subunit I | EC 1.6.99.5 | FS_1735 | Cenarchaeum symbiosum, ABK78287, 85% | | | TS_1727 | Cenarchaeum symbiosum, ABK78287, 85%, | |  |
| nuoJ | NADH-quinone oxidoreductase, subunit J | EC 1.6.99.5 | FS_1734 | Thaumarchaeota archaeon N4, CDI06266, 72% | | | TS_1726 | Nitrosarchaeum koreensis, EGP93011, 60% | |  |
| nuoK | NADH-quinone oxidoreductase, subunit K | EC 1.6.99.5 | FS_1732 | Nitrosarchaeum limnium BG20, EPA05552, 86% | | | TS_1725 | Nitrosarchaeum limnium, EPA05552, 55% | |  |
| nuoM | NADH-quinone oxidoreductase, subunit M | EC 1.6.99.5 | FS_1730 | Thaumarchaeota archaeon MY2, gi\|757129309, 66% | | | TS_1723 | Nitrosarchaeum koreensis, EGP93013, 56% | |  |
| nuoL | NADH-quinone oxidoreductase, subunit L | EC 1.6.99.5 | FS_0001 | Thaumarchaeota archaeon N4, CDI06263, 78% | | | TS_1722 | Cenarchaeum symbiosum, ABK78291, 78% | |  |
| nuoN | NADH-quinone oxidoreductase, subunit N | EC 1.6.99.5 | FS_0002 | Cenarchaeum symbiosum, ABK78292, 85% | | | TS_1720 | Thaumarchaeota archaeon MY2, gi\|757129307, 74% | |  |
| **Gene** | **Product** | **EC no.** | **FS Locus** | **Best BLAST hit in NCBI nr (% identity)*** | | | **TS locus** | **Best BLAST hit in NCBI nr (% identity)*** | | **Comment** |
| **Complex II** | | | | | | | | | | |
| sdhA | succinate dehydrogenase flavoprotein subunit/fumarate reductase | EC 1.3.99.1 | FS_0993 | Thaumarchaeota archaeon N4, CDI06292, 81% | TS_1775 | | | Thaumarchaeota archaeon N4, CDI06292, 81%/81% | cytochrome subunit complex II | |
| (sdhC) | putative succinate dehydrogenase/fumarate reductase | (EC 1.3.99.1) | FS_0996 | Thaumarchaeota archaeon N4, CDI06289, 75% | TS_1774 | | | Thaumarchaeota archaeon MY2, gi\|757129328, 68%/68% | cytochrome subunit complex II | |
| (sdhD) | putative succinate dehydrogenase/fumarate reductase | (EC 1.3.99.1) | FS_0944 | Thaumarchaeota archaeon N4, CDI06291, 77% | TS_1753 | | | Nitrosarchaeum limnium, EPA04844, 68%/68%, | cytochrome subunit complex II | |
| sdhB | FeS-center protein of succinate dehydrogenase/fumarate reductase | EC 1.3.99.1 | FS_0995 | Nitrosopumilus maritimus, ABX12156, 75% | TS_1752 | | | Nitrosarchaeum koreensis, EGP92990, 75%/75% | cytochrome subunit complex II | |
| **Gene** | **Product** | **EC no.** | **FS Locus** | **Best BLAST hit in NCBI nr (% identity)*** | **TS locus** | | | **Best BLAST hit in NCBI nr (% identity)*** | **Comment** | |
| **Complex III: 1.10.2.2** | | | | | | | | | | |
| QcrB | putative cytochrome b/b6 domain |  | FS_0308 | Thaumarchaeota archaeon N4, CDI06303, 80% | TS_2177 | | | Thaumarchaeota archaeon N4, CDI06303, 75% |  | |
| QcrA | rieske [2Fe-2S] iron-sulphur domain protein |  | FS_0306/2136/2478/0363/1012 | Nitrosarchaeum koreensis, EGP94410, 76%/40%/58%/62%/43% | TS_2177 | | | Thaumarchaeota archaeon N4, CDI06304, 74% |  | |
| (NirD) | Ferredoxin subunits of nitrite reductase and ring-hydroxylating dioxygenases |  | FS_1762 | Thaumarchaeota archaeon MY2, gi\|757129377, 62% | TS_0500/0999/2209 | | | Thaumarchaeota archaeon N4, CDI05760, 76%/Nitrosopumilus koreensis AR1, AFS80408, 67%/Nitrososphaera gargensis, AFU57642, 33% |  | |
| **Gene** | **Product** | **EC no.** | **FS Locus** | **Best BLAST hit in NCBI nr (% identity)*** | **TS locus** | | | **Best BLAST hit in NCBI nr (% identity)*** | **Comment** | |
| **Complex IV** | | | | | | | | | | |
| CtaA | cytochrome oxidase assembly protein |  | FS_2325 | Thaumarchaeota archaeon N4, CDI06438, 66% | TS_2254 | | | Thaumarchaeota archaeon MY2, gi\|757129436, 59% |  | |
| NosZ | blue (type1) copper domain-containing protein | (EC 1.9.3.1) | FS_2326 | Thaumarchaeota archaeon N4, CDI06439, 54% | TS_2255 | | | Thaumarchaeota archaeon N4, CDI06439, 54% |  | |
| CyoB | Cytochrome c oxidase subunit I | EC 1.9.3.1 | FS_2328 | Nitrosarchaeum limnium, EPA05490, 69% | TS_2256 | | | Nitrosarchaeum koreensi, EGP92915, 71% |  | |
| CyoA | cytochrome c oxidase subunit II |  | FS_2329 | Nitrosarchaeum limnium, EGG41795, 67%, | TS_2257 | | | Nitrosarchaeum limnium, EGG41795, 65%, |  | |
|  | heme transporter CcmC |  | FS_2330 | Nitrosopumilus maritimus, ABX12078, 48% | TS_2258 | | | Thaumarchaeota archaeon N4, CDI06442, 71% |  | |
| **Gene** | **Product** | **EC no.** | **FS Locus** | **Best BLAST hit in NCBI nr (% identity)*** | **TS locus** | | | **Best BLAST hit in NCBI nr (% identity)*** | **Comment** | |
| **Complex V: V-type ATPase** | | | | | | | | | | |
| atpI | archaeal A1A0-type ATP synthase, subunit I | EC 3.6.3.14 | FS_2682 | Thermoplasma volcanium GSS1, gi\|499218772, 38% | TS_1116 | | | Thermoplasma volcanium GSS1, gi\|13540887, 39% |  | |
| AtpH | archaeal A1A0-type ATP synthase, subunit H | EC 3.6.3.14 | FS_2683 | Thermoplasma volcanium GSS1, BAB59196, 27% | TS_1117 | | | Thermoplasma volcanium GSS1, BAB59196, 28% | atpH is absent in neutrophilic Thaumarchaeota | |
| AtpD | archaeal A1A0-type ATP synthase, subunit D | EC 3.6.3.14 | FS_2685 | Thermoplasma volcanium GSS1, gi\|499218770, 46% | TS_1119 | | | Thermoplasma volcanium GSS1, gi\|13540885, 48% |  | |
| atpB | archaeal A1A0-type ATP synthase, subunit B | EC 3.6.3.14 | FS_2686 | Thermoplasma volcanium GSS1, gi\|499218769, 67% | TS_1120 | | | Thermoplasma volcanium GSS1, gi\|499218769, 70% |  | |
| atpA | archaeal A1A0-type ATP synthase, subunit A | EC 3.6.3.14 | FS_2687 | Thermoplasma acidophilum DSM 1728, gi\|499202891, 67% | TS_1121 | | | Thermoplasma volcanium GSS1, gi\|13540883, 69% |  | |
| atpF | archaeal A1A0-type ATP synthase, subunit F | EC 3.6.3.14 | FS_2688 | Thermoplasma volcanium GSS1, gi\|499218767, 41% | TS_1122 | | | Micrarchaeum acidiphilum ARMAN-2, EET90350, 44% |  | |
| atpC | archaeal A1A0-type ATP synthase, subunit C | EC 3.6.3.14 | FS_2689 | Thermoplasma acidophilum DSM 1728, gi\|499202889, 34% | TS_1123 | | | Thermoplasma acidophilum DSM 1728, gi\|16081188, 36% |  | |
| atpE | archaeal A1A0-type ATP synthase, subunit E | EC 3.6.3.14 | FS_2690 | Thermoplasmatales archaeon, EMR75141, 30% | TS_1124 | | | Thermoplasma volcanium GSS1, BAB59190, 30% |  | |
| atpK | archaeal A1A0-type ATP synthase, subunit K | EC 3.6.3.14 | FS_2691 | Picrophilus torridus DSM 9790, gi\|499490655, 94% | TS_1125 | | | Micrarchaeum acidiphilum ARMAN-2, EET90353, 90% |  | |

*Best non-*Nitrosotalea* hit, words in red indicate the best non-thaumarchaeotal genes hit.

**Table S12. Homologous and non-homologous components of the A-, V- and F-type ATPases.**

| A-type ATPase Acidophilic Thaumarchaeota | V-type ATPase Prokaryotes (*Enterococcus hirae*) | V-type ATPase Eukaryotes (*Saccharomyces cerevisiae*) | A-type ATPase Neutrophilic Thaumarchaeota | F-type ATPase (*Escherichia coli*) |
| --- | --- | --- | --- | --- |
| *Hydrophilic catalytic A_1_/V_1_/F_1_ domain* | | | | |
| A-subunit; *atpA* gene | A-subunit; *ntpA* gene | A-subunit; *VMA1* gene | A-subunit; *atpA* gene | β-subunit; *atpD* gene |
| B-subunit; *atpB* gene | B-subunit; *ntpB* gene | B-subunit; *VMA2* gene | B-subunit; *atpB* gene | α-subunit; *atpA* gene |
| *Central stalk* | | | | |
| D-subunit; *atpD* gene | D-subunit; *ntpD* gene | D-subunit; *VMA8* gene | D-subunit; *atpD* gene | **-** |
| - | **-** | **-** | **-** | γ-subunit; *atpG* gene |
| F-subunit; *atpF* gene | F-subunit; *ntpG* gene | F-subunit; *VMA7* gene | F-subunit; *atpF* gene | **-** |
| - | **-** | **-** | **-** | ε-subunit; *atpC* gene |
| C-subunit; *atpC* gene | C-subunit; *ntpC* gene | d-subunit; *VMA6* gene | C-subunit; *atpC* gene | **-** |
| *Membrane-embedded A_0_/V_0_/F_0_ domain* | | | | |
| K-subunit; a*tpK* gene | K-subunit; *ntpK* gene | c-, c'- and c''- subunits; *VMA3*, *VMA11* and *VMA16* genes | K-subunit; a*tpK* gene | c-subunit; *atpE* gene |
| I-subunit; a*tpI* gene | I-subunit; *ntpI* gene | ɑ-subunit; *VPH* and *STV1* genes | I-subunit; *atpI* gene | **-** |
| - | **-** | **-** | **-** | ɑ-subunit; *atpB* gene |
| *Peripheral stalk* | | | | |
| (EH)_2_* | (EG)_2_ | (EG)_3_ | ? | (b_2_δ_1_)_1_ |
| E-subunit; *atpE* gene | E-subunit; *ntpE* gene | E-subunit; *VMA4* gene | E-subunit; *atpE* gene | **-** |
| H-subunit; *atpH* gene | G-subunit; *ntpF* gene | G-subunit; *VMA10* gene | **-** | **-** |
| - | **-** | **-** | **-** | b-subunit; *atpF* gene |
| - | **-** | **-** | **-** | δ-subunit; *atpH* gene |

This table is based in part on the results of previous studies by (55, 63, 69). Orthologous subunits are shown in the same row of the table; unrelated but functionally analogous subunits are shown in adjacent rows shaded in the same color. Dashes indicate the absence of the respective proteins. *subunits in the brackets indicate the subunit stoichiometry of peripheral stalks, and the numbers adjacent to the brace indicate numbers of peripheral stalks in A-, V- or F-type ATPase. ?indicate the structure of peripheral stalk has not been solved.

**Table S13. The sequence identity of A-type *atp* operons and flanking genes among hadopelagic marine, neutrophilic estuarine/coastal marine, WCA and neutrophilic terrestiral group of AOA.**

| **Hadopelagic Marine** | **Sequence identity (%)** | **Concatenated 8 subunits of A-ATPases** | **Subunits of A-ATPases** | | | | | | | | **Gene flanking A-ATPase operons** | |
| --- | --- | --- | --- | --- | --- | --- | --- | --- | --- | --- | --- | --- |
|  |  |  | **A** | **B** | **C** | **D** | **E** | **F** | **I** | **K** | **Sulfurtransferase** | **PyrI** |
| **Neutrophilic estuarine/**  **coastal marine** | Nitrosopumilus maritimus SCM1 | 85.0-85.1 | 88.2-88.8* | 92.5-92.9 | 71.2-71.7 | 86.9-86.9 | 82.2-82.2 | 77.7-79.3 | 56.1-56.3 | 89.2-89.2 | 68.3-69.6 | 91.2-91.2 |
|  | Ca. Nitrosopumilus koreense AR1 | 86.0-86.1 | 89.2-89.4 | 92.5-92.9 | 77.0-77.4 | 86.9-86.9 | 84.6-84.6 | 81.0-83.6 | 57.4-57.7 | 90.0-91.7 | 67.7-68.3 | 91.2-91.2 |
|  | Ca. Nitrosopumilus piranensis D3C | 85.9-86.0 | 88.6-88.8 | 92.5-92.9 | 76.6-77.0 | 88.1-88.1 | 84.6-84.6 | 81.0-83.6 | 58.0-58.5 | 90.0-91.7 | 69.0-70.3 | 91.2-91.2 |
|  | Ca. Nitrosopumilus salaria BD31 | 84.1-84.3 | 88.0-88.2 | 93.3-93.8 | 75.4-75.8 | 84.4-84.4 | 79.4-79.4 | 79.6-81.0 | 54.7-55.0 | 90.0-91.7 | 69.0-70.3 | 89.4-89.4 |
|  | Ca. Nitrosopumilus adriaticus NF5 | 84.3-84.4 | 88.6-88.6 | 93.8-94.2 | 73.7-73.7 | 83.1-83.1 | 77.8-77.8 | 87.2-88.7 | 53.6-54.1 | 88.2-88.2 | 70.9-71.5 | 86.8-86.8 |
|  | Ca. Nitrosopumilus sediminis AR2 | 83.9-84.0 | 87.8-88.0 | 92.9-93.3 | 75.0-75.6 | 85.0-85.0 | 73.3-73.3 | 79.3-81.0 | 56.3-56.6. | 90.0-91.7 | 71.2-73.4 | 86.8-86.8 |
|  | Ca. Nitrosomarinus catalina SPOT01 | 83.1-83.2 | 85.7-85.9 | 92.5-92.9 | 75.0-75.8 | 86.3-86.3 | 66.4-66.4 | 81.0-82.6 | 58.8-59.0 | 88.2-88.2 | 71.2-73.4 | 79.4-79.4 |
|  | Ca. Nitrosopumilus sp. MED740 | 82.2-82.9 | 85.7-85.9 | 92.5-92.9 | 75.0-75.8 | 86.3-86.3 | 66.4-66.4 | 81.0-82.6 | 57.4-57.7 | 88.2-88.2 | 73.4-74.7 | 79.4-79.4 |
| **Neutrophilic estuarine/**  **terrestrial** | Ca. Nitrosarchaeum limnium SFB1 | 83.8-83.9 | 86.3-86.5 | 94.2-94.6 | 75.0-76.2 | 88.1-88.1 | 70.5-70.5 | 79.3-81.0 | 58.0-58.2 | 88.2-90.0 | 69.6-70.3 | 89.5-89.5 |
|  | Ca. Nitrosarchaeum limnium BG20 | 83.7-83.8 | 85.7-85.9 | 94.6-95.0 | 79.3-80.1 | 88.1-88.1 | 70.5-70.5 | 81.0-82.6 | 57.2-57.4 | 88.2-90.0 | 73.4-74.7 | 89.5-89.5 |
|  | Nitrosarchaeum koreense MY1 | 83.5-83.6 | 85.4-85.7 | 94.2-94.6 | 78.9-79.7 | 90.5-90.5 | 60.5-60.5 | 76.0-77.7 | 57.4-57.7 | 88.2-90.0 | 69.6-70.9 | 86.8-86.8 |
| **WCA** | Ca. Nitrosopelagicus brevis CN25 | 76.0-76.2 | 83.3-83.5 | 86.2-87.1 | 43.7-44.3 | 79.6-79.6 | 52.6-52.6 | 59.5-61.4 | 51.1-51.6 | 84.6-86.4 | 49.7-50.5 | 68.3-68.3 |
| **Neutrophilic**  **terrestrial** | Ca. Nitrosotenuis uzonensis N4 | 77.9-78.0 | 84.6-84.8 | 86.2-87.1 | 60.1-60.6 | 79.3-79.3 | 58.9-58.9 | 53.3-55.4 | 54.4-55.0 | 84.6-84.6 | 58.7-59.5 | 78.5-78.5 |
|  | Ca. Nitrosotenuis chungbukensis MY2 | 76.9-77.0 | 83.7-83.9 | 86.6-87.1 | 58.2-58.7 | 79.3-79.3 | 50.9-50.9 | 48.9-51.1 | 56.9-57.4 | 84.6-84.6 | 63.0-63.6 | 77.5-77.5 |

*indicates the amino acid sequence identity between the subunit A of *Nitrosopumilus maritimus* SCM1 and those of hadopelagic marine group in Fig 2.

**>The synthesized DNA sequence of the V-type *atp* operon (8623bp)**

ATGATGGTTCTGAACAAACGTTCTAAACTGTCTATTCTGGTTATTGCTGCTGGTCTGACCATTGCTCTGAGCACTTTCGTGAGTACCACCCCGGCGTTTGCGGCTGCGGATTCCAGCACCACATCGGGCCTGACCAAACCGCTGCTGGCGATCGCGGCGGCGATCGCAATCGCGGGCGGCCTTATTGGCACCGGCAACGCGCAGCAGGGCATCGGCGCGGCGGGTATGGGCATCATCGCGGAAAAACCGGAAAAATTCGGTCAGGTTCTGTTCTTCTTCGTTATCCCGGAAACCCTGTGGATCATCGGCTTCGTTCTGGGCATCATCCTGCTGCTGGGCATCCTGTAATGGTTTTCAGTAATGAGCATCGAAACCTTCATCCAGGAAATCGAAACCCGTAAACGCAAAGAAATCGAAGACCTGGAAAAAGACCTGCAGGAAAGCAAAAGCCGTCTGCAGGCGGAAATGAACAACACCCTGAAAGAAATCCAGGAACGTTTCAGCACCGAGGCGAAAGTGAAAAGCGAACGCGAACAGGCGCGTATCATCGAAGCGTCTAAACTGCAGGCGAAGAAAATCATGTTCGACGCGATCAACGCGAACATGCAGAGCGCGTTCACCATGATCCAGAAGGAAATCAAAAACTACACCAACAGCCCGCAGTACAAGAAAAGCCTGGAGACCATGGTGAGCAACAGCAAGAAAAAACTGGGCCAGAACATCATCGTGCACTGCCGTGAGGAAGACAAAAGCATCCTGAAAGAACTGGGCGTGACCACCAGCAAAAGCATCAAAACCCTGGGCGGCATCATCGCGGAAAACAAAGAGGGCACCCGCGAACTGGACCTGACCTTCGAGGAACTGCTGCGTACCAACGAAGACCAGGTTAAAAGCTTCCTGAGCGAAAAAATGTAATGCCGACCAGCCAGTACGCGAGCAGCTTCGGCCGTCTGCAGGCGATCAGCCTGAACCTGCTGAGCAAAGAAGTTATGCAGAACCTGATGAAAGCGAAAGATGAAGTTGACATGGTTAAAGCGCTGGAATCAACCTGGTACAAACCGGAGATCGAAAAAGCGGCGTCTATCTTCAAAGAAAGCGAACTGCTGGAAGTTGCGCTGAACCGTCACCTGGTTTACATCAACAAAACCGCGCTGGAAGCGACGCCGTTTAACGGCAAAAGCGCGATCCGTGCGTACCTGAGCAAATGGGATATCTACAACATCGAACTGATCCTGAGCGCCAAAAGCATGGGTCGCCCGATCAGCGAAACCGAAAGCTTCCTGGTTTCGTCCCGTAACGTGCCGGCAGGTATCTCTGCGGGCAACATCAGCCACGATGAAATGAAAATCATCCTGAGCCAGACCGGCGTTGATGGTGTTGTGAACCAGCTGGTGAAATACAACTACGGTACCATCCTGATGCAACACCTGGAAACCTACCAGAATAACGGCAACCTGGGTCCGATGATGAGCGCGCTGCAGACCTTCTACTACCGTAACCTGCTGGAAAGCCTGAAATTCTTCCAGGGCGATGAAGGTCTGATCCGTGACTTCATCCGCGCAGAAATCGATAAAAAGAACGTTTTGTCGCTGCTGAAAGCAAAAGAATCCGATCTGGATAAAGAAATCGTGAGCAAACACCTGATCGAAGGCGGCAAAATGACCAAAAACGAACTGCTGGATGTATACAACGCGAAAGATGTTTCCGAAATCGTGGGTCGTGTTGAAAACCGTTTCATGCTGGTGAACGCTCTGGCGCAGTACAAAAAATCCAACTCCCTGATCGACTTCGAAGTCGCGCTGGATAAATTCATCAACAGCGAATACGTTAAAAAACTGAAAAACATCGCGCTGAGCATCGGCACCATCTTCTACTTCATCATCAACACCGAACACGAACGTGAAAACATCAAACGTATCGCGTACGGTAAACGCTATAACCTGAGCGCTGACTACATTAATAGCTTACTGCTGATTGAATGAACATGAGCCAGCAGAAATCTAACACCACCGGTCGTATAGCCGTTGTTGGTGAACGTGAACTGGCAATCGGCTACAACCTGCTGGGCATCGAAGATACCTTCATCACCTCTGGTGAAGAAGCGAGCAAAATCATCCAGGATTTGTTCTCTTCTGGTAACTACTCTCTGATCATCATCAGCGATATCGTTCGTAGCAGCCTGCCGGCGATCTTCCGTAAAAAGATCGAAGCGAGCATCGAACCGCTGGTTATCTTCATGCCGGCGCTGGAAGGTAACATCCAGGAAGAAAGCATCAGCGTTCTGGCGAAACGTGTTCTGGGCATCAGCATCCCGAGCAGCTAATGATGATAAATGGCTGATGGTATCATCTCCCGTGTTAGCGGCCCGGTTGTTATTGCGAGCGGTCTGGAAGGCGCGCAGATGTTCGATGTGGTTCGTATCGGTGATATGGGTCTGGTGGGCGAAATTATTCGCATTGAAGGTAACAAAGCGACCGTTCAGGTGTACGAAGACACCACCGGTCTGCGTCCGGGCGAAAAAGTAATTAACACCAAACGCCCGCTGTCTATGCAGCTGGGTCCGGGTCTGCTGACCAGCATTTATGATGGCATTCAGCGTCCGCTTGATGTTCTGCGTGAACAGTCTGGTGATTTTATCAGCCGTGGTAAAGTGATTCCGGCGCTGGATCAGACCAAAAAATGGGAATTTGTTCCGATTAAGAAAAAAGGCGACCATGTTAGCCCAGGTGAAATCATCGGTGAGGTTCAGGAAACCCCGCTGATTATGCACAAAATCATGATCCCGTACAATGTGAAAGGCACCCTGACCGACATCTCCGAAGGTAAATATACCGTTAACGATATCGTGGCAAGCGTGCAGAACGGCACCAAAGCCGATATCGGTCTGTCCTCTTGGTGGACCGTGCGTACTCCTCGTCCGGTGCTGCGTAAACTGGCTCCGGAAGAACCGCTGCTGACTGGTCAGCGTGTCCTGGACACCTTCTTCCCGGTTGCGAAAGGCGGCACCGCCGCGATTCCGGGCCCGTTTGGTTCCGGTAAAACCGTTACCCAGCAGCAGCTGGCCAAATGGGCTGATTCCAACGTTATCGTTTATGTGGGTTGTGGCGAACGCGGTAACGAAATGACCGAAGTGCTGACCACCTTTCCGAAACTGGAAGATCCGAAATCTAAACGTCCGCTGATGGAACGCACCATCCTGGTGGCCAACACCTCTAACATGCCTGTAGCAGCGCGCGAAGCTTCTATTTATACCGGTATCACCATGGGCGAATACTACCGTGACATGGGCTACGGTGTTGCGCTGATGGCAGATTCCACCAGCCGCTGGGCGGAAGCGCTGCGTGAAATTAGCGGTCGCCTGGAAGAAATGCCGGGCGAAGAAGGCTACCCGGCGTATCTGGGCCGCCGTCTGGCAGAATTCTACGAACGTGGCGGCAAAGCGGTTGTTATTAGCCCGGAAGAACGTGTGGGCTCTCTGACTCTGGTTGGCGCAGTTTCCCCGCCGGGCGGTGACTTCTCTGAACCGGTGTCCCAGAACACTCTGCGTGTTACCCGTGTTTTCTGGGCTTTAGACGCGAGCCTGGCATCTCGCCGCCACTTCCCGTCTATTAACTGGCTGACCTCCTATAGCCTGTACGCTGATGGCATGGGTGACTGGTATAAAAACAACGTGGCAGCCTCCTGGATTCAGAGCCGCAAGGAAGCTCTGGAAATCCTGCAGAAAGAAAGTGAACTGCAGGAAATTGTCCAGCTGGTTGGCTACGACGCGCTGCCGGAACCGGAAAAAGGTGTGCTGGACACTGCTCGTTCCATCCGCGAAGATTACCTGCAGCAGAGCGCTTATGACGATGTTGACACCTATACCTCTATTCGTAAACAGTTCCTGATGCTGAGCACCATTCTGGAATTCGGCAAAATGGAAGCCGATGCCATCAAAAAAGGCATCACCTCTGTTAAAGTTGGCCAGCTGGAAAGCCGTAAAATGATCAGCAAAATTAAATGGACCAAAGAAGATCAGGTTGAACAGCTGGTCAAAGACACCAAATCTAAAATGCAGCAGGAATTTTCTAGCCTGTCTATGGAAGCGTCTCGTTAATGAGCGGCATCGCGTTCAAAACCCTGAGCAAAATCGCGGGCCCGCTGATGTTCGTTGAAGGCGTTGAAAACGCAGCGTACGGTGAAATGGTTGAAATTAAACTGCAGAACGGTGAACGTCGTCAGGGCCAGGTTCTGGATACCCGCCAGGGTCTGGCAGTTGTTCAGGTTTTCGGCGCTACCCTGGGTCTGAACATCGGTGATACCTCCGTTAAATTCCTGGGTGAAACCGCTCAGCTGGCAGTTAGCGATGAAATGTTGGGTCGTGTTTTTGATGGTCTGGGTAACCCGCGTGATAACGGCCCGAAAATCGTTAGCAAAACCAAAGTGGATCTGGTTGGTAGCGGCATCAACCCGTACAGCCGTGAAGAACCGTCTGAATTTATTCAGACCGGTATGTCTAACATTGACGGTATGAACACCCTGGTTCGTGGCCAGAAACTGCCGATCTTCAGCGGCGCGGGCCTGCCTCACAACCTGCTGGCGGCGCAGATCGCACGTCAGGCGAAAGTTCTGGGTGGCTCTGAAAACTTCTCCGTTGTGTTCGCGGCGATGGGCATCACCTCGGAAGAAGCGAACTTCTTCGTTAAACAGTTCGAAGAAAGCGGCGCTCTGGGTCGTACCGCGCTGTTCCTGAATCTGTCTTCTGATCCGTCTATGGAACGTCTGCTGACCCCTCGCCTGGCTCTGACTACCGCAGAATACCTGGCTTATGAACGTGACATGCATGTTCTGGTGATTATGACTGATATGACCAACTATTGTGAAGCTCTGCGTGAAATTTCTGCAGCACGTGAAGAAGTTCCGGGTCGTCGTGGCTATCCAGGCTATATGTACACCGATCTGAGTTCCCTGTATGAACGTGCCGGCAAAATTAAAGGTCGTAACGGCAGCGTGACCCAGATTCCGATTCTGACCATGCCGGCTGATGATATCACCCATCCGATCCCTGATCTGACTGGTTACATCACCGAAGGTCAGATCGTGATGAGCCGTGACCTGCACCGTGGTGATATTCACCCGCCGGTTGATGTTCTGACCTCTCTGAGCCGCCTGATGAACCAGGGCATTGGTAAAGGTAGCACCCGTGAAGATCATCGTTCTCTGGCAGATCAGCTGTACTCTTCTTACGCACAGGGTAAAGATGCACGTTCTTTAGCTGCGATCGTTGGTGAAGAAGCACTGAGCGATCTGGATCGTAAATTCATGAAAGTTGCGAACGATTTCGAACGTAAATTCGTTAACCAGGGCATGGATGAAAACCGTAGCATCGAACAGACCCTGGATATCGGTTGGGAACTGCTGTCTGAACTGTCTGATTCTGAACTGAACCGTATCAAACCGGAATTCATTGAAAAATACCGTAAACGTAGCGGTACCGGTGCGTAAaaATGTTGCCGTCCGTTATCAACATCCGTCCGACCCGTCTGGAATACATCCGTACCAAACGTCGTATCATCGTTGCGAAGAAAGGACTGAAACTGCTGAAACTGAAACGTCAGGCGCTGATCCTGGAATTCTTCAACACCTCTAAAACCGTGGCGAGCCTGCGTAGCGGCCTGCAGATCGAACTGGTTAAAGGCTACCAGGCGATCCGCATGGCGGAAATGCTGGCGGGCGCGATGCGTCTGGAAAACGAAGCGATGAAAATCCCGCAGCTGAACAAACTGCAGATCACCCCGAAAAACGTTATGGGCGTGCGTATCCCGAAAATCGAAGGTGGCAAAAGCGATCAGGTGATCACCGAACACCTGCTGGAACTGCCGCCGTCTATCAACGAAGCGATCAAAGCGTTCCGTAACGTTCACAAAATGGTTCTGGATGTTGCGGAAAAAGAAACCACCCTGCGTAAACTGCTGCTGGAAATCGAAAAAACCAAGCGTAAAAGCAACGCGATCGAAAACGTTTTTATCCCGCGCCTGCAGGCGGCGATCAAATTCATCATTTTTCGTCTCGATGAAATGGAACGTGACACCTTCATGATGCTGAAAACCGTTAAACGTAAAATGGGTGAGCGTGAACAGGAAAGCCTGAAACTGAAAGAAAAAGAAATCCTGGCGTAATTTTGCAAATCAACAACAGAAAAGATTTTTTATGATAATTAGAGCATTCAAGCTAGGTAATATTCTTGCTACTGTAGCCTTGGCTCTCTATGCTGGAAAGCACTTTTTTGGAGTTTAAAATGTCGACCAGCGAAAAATATGTGCACAGCCTCAAACAGATCAAAGAAATCGAAGACCGCAGCCAGAAAGAAATCGACGAACAGAAAAAACGTGTTGCGGAAGAACTGCGCAACTTCGAAACCTACGCGATCCAGAGCATCACCGCGGCGAAAGCGGACGGCGAAAAACTGATCGAATCCTCCATCGACCAGGCGCGCAAAAAAGCGCACGCGGAAACCGAGAAAATCATCGAAGACGCGAAAAACAAAGCGAAAACCGTTTCCAGCCGCATCGACAGCCAGACCGTGAAAGAAATCATCGACATCCTGCTGAAAGAAGTGTAAAATATGTTGGTTCTGAAACCGGTTCCGATGGGCCGTATCGCTGTGCTGGGCCTGCGTAAAGAAAAACAGATCGTTGTTTCTATTCTGCACGATCTGCACGTGGTGCAGCTGGAATCTCTGTCCAAAGATGTTGCGACCCTGGTTCGTAACGAACGTGACAACGAAACCTTCCGTCAGGTTTCTGATCAACTGCTGCGTATGAAAGCGCTGAAAACCGTTCTGCCGCCAATCGAATCTACCCAGTGCCAGCGCTTCACCAGCATCGAACAGATCATCCAGGCTGCCAAAAGCATCGATATCGACTCCAACGTTGCGACCCTGGAAAAAGAAAAAGAACATCTGCTGACCCAGCTGAAAGAAACCGAGAACAACATCAAACTGGTTGAAGAATTCAGCTTCTTCCCGGAAGATTTCAACGTTCTGCAGCTGAAAATGGCACATTCTTATTTCGGCCGTCTGGACAGCAAAAACTACACCGCATTCAAAAAGACCCTGGATGTCAACTCTCAGGATGTATTCGTGTATCCGAAAGAAGGCAAAGATACCACTAACATTGTGCTGATTACCTTCCCGAACTTTCCACCGCATGCGCTGGCGACCGTTGTTCAGGAATATGACGTGAAACTGGAAGCGGTGCCGAAACTGGACGGCAAAGCGGATCAGCTGATTCAGTCCCTGAAAGCGAAACACGCGGATATCTCCCACAAACTGAAAGAAGTTGAACGCCAACTGGGCGAAATCTCTAAAAGCCACTATATCAACATTGTATGTATCGAAGAACAGCTGGAAATCGAAAACAAAAAACTGGAAGTTGTTGATAACCTGGGTGTGACTAGCGACTCCTTCGCCCTGGAAGGTTGGATTCCGAAATCTAAAATTGATCAGCTGAAAAACGTATTCGCGAACAACACCAAAGGTACCATGCTGTTCGAACTGGAAACCGACGAACATCCGCCGACCCAGTTTGACAACCCGAAACGTTTTAAACTGTTTGAAGCATTCATTCGTTTCTACAGCCTGCCGGAAGGTCGTGAATTCGATCCGACCCTGATTTTTGGCCTGCTGTTCCCGATCTTTTATGGCATGATGGTTGGTGACGCTGGTTACGGTCTGGTTATCCTGCTGGTTTGCCGTTGGGTTATTCGTCGCATCGATGGCGGCAAAAAGGACTTCAACATCATGCCGGGTATGCTGCGCAAATTCGCGCTGAACATCCTGAAACGTCGTCAGATGGTGAAACTGGCTAAAGCTATGACTCCGGGCGCGATCATCGCCATCGGTCTGGGCTTCGTGTTTAACCTGTACTTCGGCTTCCACCTGAACGGCTACCTGTTCTCTTACCTGAACTCTACCTTCGGTCTGCACCTGCCGGCTGATGGTGCGCTGTTCAACCCGATTACCTCCCTGCGTAAACTGCTGCTGATCAGCGGCTATATCGGTCTGGGCATGGTGACCTTTGGTCTGATCCTGGGCGTTCTGAACAGCCTGCGTGAAGGCCTGAAAAAACACGCCATCGGTAAAATCGGTTGGCTGCTGTTTGGTTGGGGCGTTGTTCTGTTTGGCCTGGCCCTGATGCATCACCAGCACGTGAACCCGGTGCACTCTGCTCAGGGTGCTGCATACATCGGTCTGATGATTGGTGGCGTGGCGCTGATGTTCATCGGTGAAGGTCCGCGCGCGATCATGGAACTGCCGAGCATTGTTAGCCACATCCTGTCCTACACCCGTATCATCGGTATCCTGCTGGCGAGCGTTATCCTGGCAGACGTTATTGACTTCATTTTCATCAAAACCCTGCACCACTCCATCCCGTACATCATTCTGGGCACCATTATTCTGTTCATCGGTCACATCTTCAACATCGTAATCGGCGTGTTCGAACCGGGCATCCAGGGTGCGCGCCTGATCTACGTTGAATTCTTCTCTAAATTCTACCATGGTAACGGCCGCGCGTTCCGCCCGTTCGGCAGCGCGCGTAAATTCACCTATGATCAGTATGCTATGCAGACCGAAAAGAAATAA
